# Are the results of open randomised controlled trials comparing antipsychotic drugs in schizophrenia biased? Exploratory meta- and subgroup analysis

**DOI:** 10.1038/s41537-024-00442-8

**Published:** 2024-02-15

**Authors:** Stefan Leucht, Spyridon Siafis, Johannes Schneider-Thoma, Aran Tajika, Josef Priller, John M. Davis, Toshi A. Furukawa

**Affiliations:** 1 Department of Psychiatry and Psychotherapy, Technical University of Munich, School of Medicine and Health Klinikum rechts der Isar, Ismaningerstr. 22, 81675 Munich, Germany; 2 German Center for Mental Health (DZPG), Munich, Germany; 3 Department of Health Promotion and Human Behavior, Graduate School of Medicine / School of Public Health, Kyoto University, Kyoto, Japan; 4 Psychiatric Institute, University of Illinois at Chicago (mc 912), 1601 W. Taylor St., Chicago, IL 60612 USA; 5 Johns Hopkins University, Baltimore, MD USA

**Keywords:** Schizophrenia, Psychosis

## Abstract

A recent meta-epidemiological study did not reveal major differences between the results of blinded and open randomised-controlled trials (RCTs). Fewer patients may consent to double-blind RCTs than to open RCTs, compromising generalisability, making this question very important. However, the issue has not been addressed in schizophrenia. We used a database of randomised, acute-phase antipsychotic drug trials. Whenever at least one open and one blinded RCT was available for a comparison of two drugs, we contrasted the results by random-effects meta-analysis with subgroup tests. The primary outcome was overall symptoms as measured by the Positive and Negative Syndrome Scale, supplemented by seven secondary efficacy and side-effect outcomes. We also examined whether open RCTs were biased in favour of more recently introduced antipsychotics, less efficacious or more prone to side-effects antipsychotics, and pharmaceutical sponsors. 183 RCTs (155 blinded and 28 open) with 34715 participants comparing two active drugs were available. The results did not suggest general differences between open and blinded RCTs, which examined two active drugs. Only 12 out of 122 subgroup tests had a *p*-value below 0.1, four below 0.05, and if a Bonferroni correction for multiple tests had been applied, only one would have been significant. There were some exceptions which, however, did not always confirm the originally hypothesized direction of bias. Due to the relatively small number of open RCTs, our analysis is exploratory, but this fundamental question should be given more scientific attention. Currently, open RCTs should be excluded from meta-analyses, at least in sensitivity analyses.

## Introduction

A recent meta-epidemiological study across various medical fields did not reveal major differences between the results of blinded and open RCTs^1^. These findings were in contrast to the results of a frequently quoted study published in the 1990s, which found that insufficient blinding was the second most important measurable factor after allocation concealment to produce bias^2^. The question is very important for various reasons: first, blinding is difficult and expensive to do. Second, trial quality assessment tools, such as the risk of bias tool of the Cochrane collaboration^3^, include the effectiveness of masking as an indicator of bias. If blinding did ultimately not turn out to be associated with bias, these tools would need to be revised, and the results of open studies would need to be judged differently. Third, it is likely that patients are less willing to consent to trials in which they do not know their actual treatment. Therefore, the representativeness of the trial population may be reduced.

However, this question has not been analysed in antipsychotic drug trials in schizophrenia. We filled this gap by pairwise meta-analysis and subgroup tests of randomised, acute-phase antipsychotic drug trials in schizophrenia.

Our hypothesis was that open RCTs find different effect sizes between two antipsychotics as blinded RCTs, and the direction of this difference usually is in favour of less efficacious and/or more side-effect prone antipsychotics, more recently licenced antipsychotics and in favour of the pharmaceutical company sponsoring a trial. The aspect of “recency” addresses the phenomenon of “novelty bias”, i.e. that there is a general trend that more recent drugs do better in RCTs than older ones, e.g. due to optimism bias^4^. Moreover, if a pharmaceutical company designs a study, it wants to show that its drug is better and that this leads to rater bias, which should be more pronounced in open RCTs. Finally, any such differences should be less frequent in objective outcomes, including weight gain and prolactin, than in subjective outcomes, in particular rating scale-based efficacy.

## Material and methods

### Search

We used the 402 RCTs with 53 463 participants included in a previous systematic review of our team^5^. All studies had been selected, their outcomes extracted, and the risk of bias judged in duplicate by at least two reviewers (see Huhn et al.^5^ for details). We included all studies regardless of their origin and language. Only studies from mainland China were excluded because quality concerns have been raised, and this situation has not changed in recent years^6–10^.

### Participants

People with schizophrenia or related disorders (schizoaffective disorder and schizophreniform disorder), irrespective of gender, race and the diagnostic criteria used. We excluded studies which were restricted to patient subgroups such as children and adolescents, first-episode patients, patients with predominant/prominent negative symptoms, people with co-morbid substance use, treatment-resistant patients, and elderly people. These patient subgroups were excluded to have a more homogeneous study sample. Thus, studies in ‘general’ patients remained as they are usually included in registrational studies. They can be best described as typically chronic adults between 18-65 with acute exacerbations of positive symptoms^5,11^. We also excluded studies in stable patients (relapse prevention studies^12^).

### Interventions

We included all second-generation antipsychotics (SGAs) available in Europe or the US, placebo and a selection of first-generation antipsychotics (FGAs) (benperidol, chlorpromazine, clopenthixol, flupenthixol, fluphenazine, haloperidol, levomepromazine, loxapine, molindone, penfluridol, perazine, perphenazine, pimozide, sulpiride, thioridazine, thiothixene, trifluoperazine, zuclopenthixol) guided by a survey among 50 international schizophrenia experts^13^. We excluded intramuscular formulations because they are primarily used for emergency use (short-acting) or relapse prevention (long-acting). We included all flexible-dose studies since these allow the investigators to titrate to the optimum dose for the individual patient. In fixed-dose studies, we included a target to maximum doses according to the “International-Consensus-Study-on-Antipsychotic-Dose”^14^. If studies used several eligible doses, we averaged the results of the individual arms using appropriate formulas^3^.

### Outcomes

The primary outcome was the change in overall symptoms of schizophrenia as measured by the Positive-and-Negative-Syndrome-Scale (PANSS)^15^ or the Brief-Psychiatric-Rating-Scale (BPRS)^16^. Secondary outcomes were the change in positive and negative symptoms, both measured with published rating scales, all-cause discontinuation and the following side-effects: use of antiparkinson drugs as a measure of extrapyramidal side-effects, sedation, weight gain, prolactin levels and QTc prolongation.

### Study design

We included open, single (rater) and double-blind (patients, raters and treating teams, usually achieved by identical capsules) RCTs of a duration between 3 weeks^17^ and 3 months following Cochrane Schizophrenia Group definitions on short-term results^18^. We excluded studies with a high risk of bias in sequence generation according to the Cochrane Collaboration´s risk of bias tool version 1^3^.

### Statistical analysis

Whenever at least one blinded and at least one open RCT was available for a comparison of two drugs, we conducted pairwise, random-effects, inverse variance meta-analyses and random-effects subgroup tests in R statistical software v 4.3.1 using the package ‘meta’ v.6.5-0^19^. The effect size index for continuous outcomes was the standardised mean difference (SMD, Hedges’s *g*) for rating scale results, the mean difference (MD) for weight gain and prolactin, and the odds ratio for dichotomous outcomes. We performed four analyses:

1. In the primary analysis, we compared the effect sizes of blinded and open RCTs of each comparison (e.g. haloperidol versus risperidone or aripiprazole versus olanzapine) by subgroup tests.
2. For continuous outcomes, we subtracted the SMDs/MDs of the open RCTs from those of the blinded RCTs. For dichotomous outcomes, we calculated ratios of odds ratios. We meta-analysed these differences/ratios to obtain an overall effect of blinded versus open RCTs. These analyses require a determination of which drug is intervention and control. Two sets of meta-analyses were performed:
  - The more recently licensed antipsychotic was considered to be the intervention, and the older antipsychotic was the comparator. The assumption was that the more recent drug would be the biased one in open RCTs (“novelty bias”^4^).
  - The drug which was less efficacious or produced more of a given side-effect in Huhn et al.^5^ was the intervention. The assumption was that open trials would be biased towards the worst antipsychotic.
3. In pharmaceutical-sponsored RCTs, we designated the sponsored drug as the intervention and non-sponsored drugs as comparators. We then meta-analysed the effect sizes and compared blinded and open RCTs via subgroup test. The assumption was that the open RCTs would be biased in favour of the pharmaceutical sponsors.

In sensitivity analyses of the primary outcome, we used a fixed-effects model, and we excluded single-blind studies. As the statistical power of subgroup tests is limited, alpha was set at *p* < 0.1 for this purpose.

## Results

The PRISMA flow chart of the search is presented in eFig. 1, information on individual studies and risk of bias are presented in eTables1 and 2. Of the 402 RCTs with 53,463 participants, 183 RCTs with 34715 participants were available for at least one subgroup test comparing the results of 144 double-blind (patient, rater and treatment team blind), 11 single-blind (rater blind) and 28 open RCTs. According to a summary of study characteristics presented in Table 1, patients in blinded trials had an approximately 4 years higher median age, a median 28 patients higher sample size and a median 6 years earlier publication year. The other characteristics were not statistically significant.

**Table 1:** Characteristics of included blinded and open RCTs

	Blinded RCTs		Open RCTs		*p*-Value*
	Median; IQR	*N*	Median; IQR	*N*	
Percentage male	64%; 48.5, 254.5	128	60%; 39.25, 97.5	28	0.74
Median mean age	36.94; 34.45, 39.57	140	33.3; 28.65, 36.21	26	<0.001
Study duration (weeks)	6; 6, 8	154	6; 6, 12	28	0.06
PANSS total score baseline	94.83; 90.35, 98.8	125	89.81; 79.44, 99.47	19	0.13
Olanzapine dose equivalent	17.11; 14.93, 21.24	152	16.1; 14.37, 17.55	27	0.06
Sample size	88; 48.5, 254.5	155	60; 39.25, 97.5	28	0.012
Publication year	2001; 1993, 2007	155	2007; 2004, 2011	28	<0.001

^*^Mann–Whitney test. *RCT* randomised controlled trial, *N* number of studies with data, *PANSS* positive and negative syndrome scale, *IQR* interquartile range.

### Overall symptoms (primary outcome), positive and negative symptoms

Results of the 17 subgroup tests comparing the results of blinded and open RCTs, which could be made for the primary outcome “overall symptoms”, are presented in eFigure 2. None was statistically significant (all *p*-values > 0.1).

**Fig. 1:**
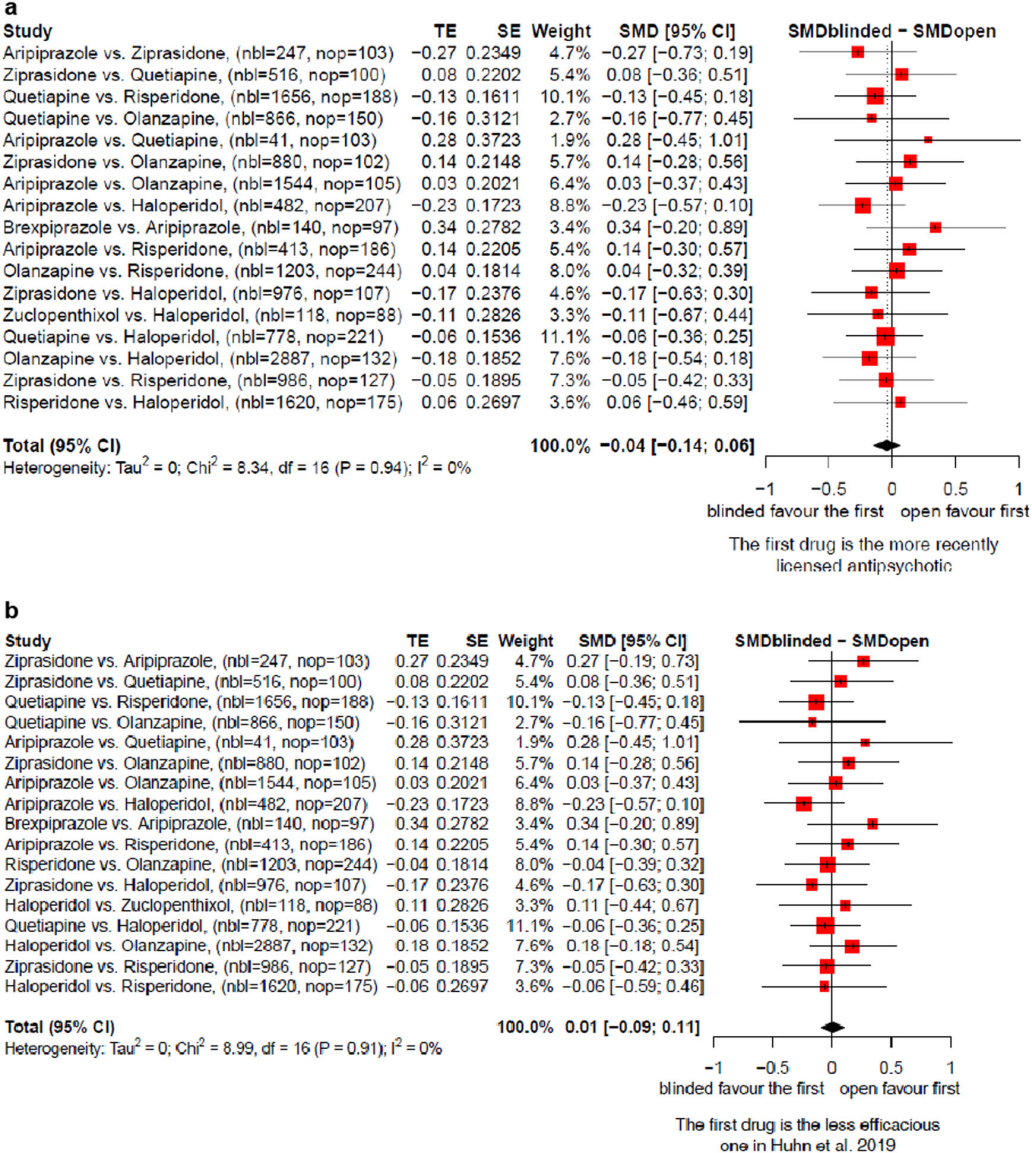
Overall efficacy, blinded versus open RCTs. **a** The more recently licensed antipsychotic is the intervention. **b** The less efficacious antipsychotic is the intervention.

On average, there was no clear difference between the open and the blinded effect sizes neither when the more recent drug was considered the intervention (SMD −0.04 [−0.14; 0.06], Fig. 1a), nor when the drug which was less efficacious in^5^ was considered the intervention (SMD 0.01 [−0.09; 0.11], Fig. 1b).

**Fig. 2:**
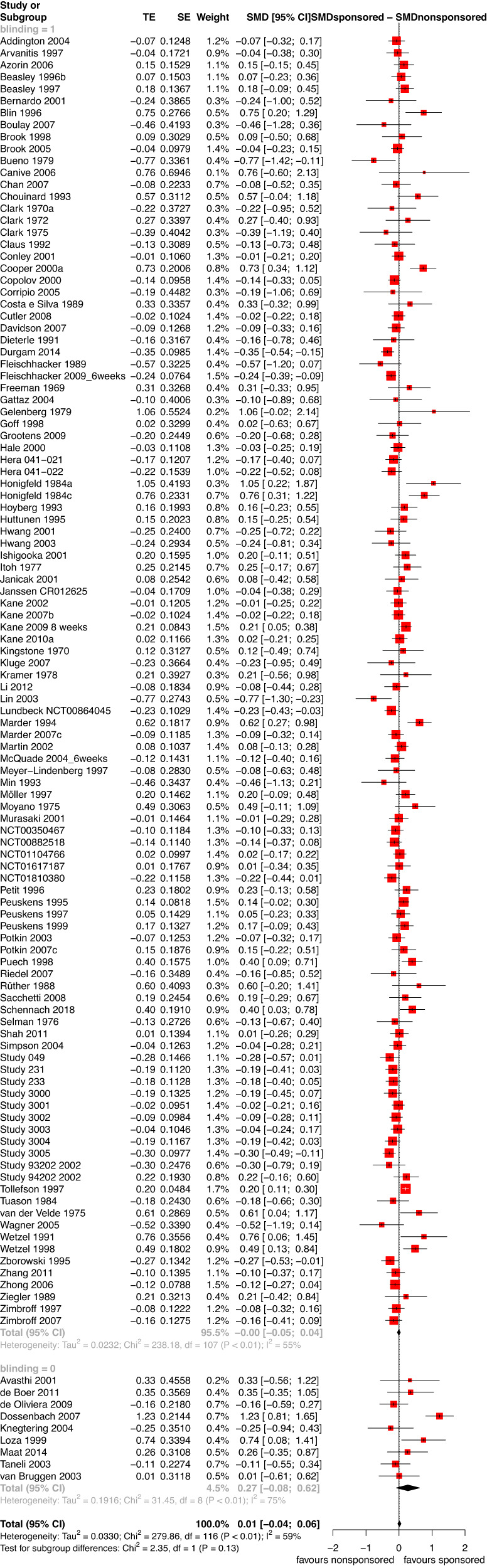
Overall efficacy of sponsored versus non-sponsored antipsychotics in blinded and open RCTs.

Neither in blinded RCTs (average SMDsponsored minus SMDnon-sponsored 0.00 [−0.05;0.04]) nor in open RCTs were the drugs of the pharmaceutical companies sponsoring the trials favoured (average SMDsponsored minus SMDnon-sponsored 0.27 [−0.08;0.62]). The subgroup difference between blinded and non-blinded trials was also not significant (*p* = 0.13, Fig. 2). It was significant, however, when a fixed effects model was used (eFig. 3d). Other sensitivity analyses did not change the results to an important degree (eFigs. 3 and 4).

Based on fewer available comparisons than for overall symptoms, there were no clear differences between blinded and non-blinded RCTs in terms of positive and negative symptoms (8 subgroup tests each, eFigs. 5a and 6a), except that in terms of negative symptoms *both* blinded, (difference in SMDs 0.05 [0.01; 0.10]), and open trials (difference in SMDs 0.21 [0.01; 0.41]), favoured the drugs of the trial sponsors, but with no clear difference in magnitude of the effect between blinded and open trials (test for subgroup differences: Chi2 = 2.25, d*f* = 1 (*p* = 0.13), eFig. 6d).

### All-cause discontinuation

Of 16 subgroup tests, 2 had *p*-values below 0.1. In the comparison of haloperidol versus risperidone, blinded trials favoured risperidone (17 RCTs, 1718 participants, OR 1.36 [1.08;1.71]), while open RCTs tended to favour haloperidol (4 RCTs, 262 participants, OR 0.61 [0.28;1.34]; test for subgroup differences: Chi2 = 3.66, d*f* = 1 (*p* = 0.06), eFig. 7a). In the comparison of quetiapine and haloperidol, blinded trials favoured quetiapine (3 RCTs, 802 participants, OR 0.72 [0.53; 0.96], while open RCTs tended to favour haloperidol (3 RCTs, 237 participants, OR 1.44 [0.70; 2.96]; test for subgroup differences: Chi2 = 3.07, d*f* = 1, = 0.08, eFig. 7a).

On average, blinded trials favoured the more recently licensed antipsychotic (mean OR/OR 0.76 [0.61; 0.96], eFig. 7b). Neither blinded nor open RCTs on average favoured the sponsored drugs (eFig. 7d).

### Use of antiparkinson medication at least once

Out of 13 subgroup tests, 5 had *p*-values below 0.1 (aripiprazole versus haloperidol, *p* = 0.02, aripiprazole versus risperidone, *p* = 0.05, haloperidol versus olanzapine, *p* = 0.04, quetiapine versus olanzapine, *p* = 0.08, ziprasidone versus risperidone, *p* = 0.07, eFig. 8a). These subgroup differences were largely driven by a single open RCT, which compared several antipsychotics (aripiprazole, haloperidol, quetiapine, olanzapine, risperidone and ziprasidone)^20^. The direction of the effect was the same in blinded and open RCTs, but differences in EPS were more pronounced in open RCTs.

Many of these findings were on second-generation antipsychotics versus haloperidol. As a consequence, open trials as a group favoured the more recently introduced antipsychotic (Fig. 8b), while blinded RCTs favoured the drugs with more EPS, according to Huhn et al.^5^ (Fig. 8c).

Concerning pharmaceutical company sponsoring, blinded RCTs (OR of ORs 1.83 [1.43; 2.34]) but not open RCTs favoured sponsored drugs. The subgroup test between blinded and open was not significant (Chi2 = 0.37, d*f* = 1 (*p* = 0.55).

### Sedation

Of 4 subgroup tests, one had a *p*-value < 0.1. Olanzapine produced less sedation than risperidone in one open RCT (OR 5.00 [1.16; 21.50]), while there was no clear difference in 3 blinded RCTs (OR 0.33 [0.02; 4.75], test for subgroup differences: Chi2 = 3.08, d*f* = 1, *p* = 0.08, eFig. 9a).

There were no other effects of blinding; see eFig. 9b–d.

### Weight gain

Only one out of 6 subgroup effects showed an effect of blinding (eFig. 10a). In haloperidol versus risperidone, 4 blinded trials favoured haloperidol (mean difference −1.04 kg [−1.58; −0.50]), while a single, small open RCT (Nam et al.^21^, 40 participants) showed a trend of risperidone being associated with less weight gain than haloperidol (1.74 kg [−0.62; 4.10], test for subgroup differences: Chi2 = 5.07, d*f* = 1, *p* = 0.02).

There were no other clear effects of blinding; see eFig. 10b–d.

### Prolactin increase

Of 6 subgroup tests, one was statistically significant. The superiority of haloperidol compared to risperidone was more pronounced in one open than in 4 blinded RCTs (difference in MD 63.7 [22.1; 105.3]; test for subgroup differences: Chi2 = 9.00, d*f* = 1 (*p* < 0.01), but the direction of the effect was the same (eFig. 11a).

There was no difference between blinded and open when the more recent drug or when the drug which produced more weight gain in Huhn et al. ^5^ was considered the intervention (eFig. 11b, c).

Both blinded 8.51 [0.14;16.87] and open RCTs 4.94 [33.75;56.14] favoured sponsored drugs, but this effect was more pronounced in the two open RCTs (Test for subgroup differences: Chi2 = 26.12, d*f* = 1; *p* < 0.01; eFig. 11d).

### QTc prolongation

There were not enough studies to allow a meaningful analysis.

## Discussion

In a recent meta-epidemiological study of Cochrane reviews, we did not find that the results of open randomised antipsychotic drug trials in schizophrenia are systematically different from those of blinded ones^1^. However, there were some exceptions to the rule. These results have implications for the design of antipsychotic drug trials and for meta-analyses.

First, the findings of each outcome must be discussed. We did not find clear evidence that overall efficacy would be biased by open RCTs. This finding is important because it usually is the primary outcome, most data were available for this outcome, and because it is a subjective, scale-rated outcome which could be biased more easily than objective outcomes such as laboratory measures or weight gain^3^. Based on fewer studies, the same was found for positive and negative symptoms. The exception was the use of a fixed- instead of a random-effects model in which open RCTs favoured sponsored drugs. However, usually, random-effects models are preferred in situations where heterogeneity is expected^3^.

In terms of all-cause discontinuation, *blinded* trials favoured risperidone and quetiapine over haloperidol, while in open RCTs, there was an opposite trend. In line with this finding, *blinded* RCTs overall favoured the more recently licensed antipsychotics, but there was no effect of pharmaceutical sponsorship. We would have expected opposite results, i.e. that the *open* RCTs favour the more recent second-generation antipsychotics. Thus, at least the effect of lack of masking was “conservative” in that it did not bias the results in favour of more recent and, thus, usually more expensive drugs.

We examined the use of antiparkinson medication at least once as a proxy for extrapyramidal side effects. Compared to blinded RCTs, open RCTs several times favoured drugs with less extrapyramidal side-effects (e.g. aripiprazole over haloperidol and aripiprazole over risperidone). This may be said to be expected because, in open trials, it may be easier for doctors and patients to refrain from using antiparkinsonian drugs when they know that they are on newer drugs. Thus, a lack of masking bias may have biased the results. It should be noted, however, that many of the findings with *p*-values < 0.1 were driven by an open RCT which compared four antipsychotics: aripiprazole, haloperidol, olanzapine, risperidone and ziprasidone^20^.

In sedation, olanzapine was favoured compared to risperidone in open RCTs, while there was no difference in the blinded trials. As olanzapine has a strong anti-histaminergic component^22^, it should be more sedating than risperidone. Thus, there might be a lack of binding bias, but it was based on a single, small, open RCT (43 participants)^23^. Sedation is a very important but understudied outcome in antipsychotic drug trials. Sedation is usually only assessed as an adverse event in RCTs. Rating scales should be used to make the assessment of this very important side effect more objective.

Finally, in weight gain, a single, small (*n* = 40), open RCT showed a non-significant trend in favour of risperidone^21^, while 4 blinded studies showed the expected statistically significant superiority of haloperidol. In prolactin increase, the expected superiority of haloperidol compared to risperidone was more pronounced in open RCTs. Moreover, both blinded and open RCTs favoured sponsored drugs, but more so open RCTs. These findings are difficult to explain because objective outcomes, such as weight gain and prolactin, are less prone to unblinding bias than subjective outcomes. The only explanation can be that treatment is changed as a consequence of a lack of blinding. For example, if prolactin increase was known in open RCTs, the investigators might have reduced the doses in flexible-dose trials.

The results are in contrast to a meta-analysis of our group in which open RCTs clearly exaggerated the superiority of second-generation antipsychotics compared to first-generation compounds^24^. The most extreme outcome was negative symptoms, where the average effect size comparing second-generation and first-generation antipsychotics was 0.2 in blinded trials but 0.5 in open RCTs, a more than 2-fold difference. However, most of the open RCTs in this previous meta-analysis came from mainland China, which has been shown to be often biased^6–10^. Thus, it is not clear whether the lack of blinding was the actual problem or the poorer quality of studies from mainland Chinese in general. For example, inappropriate randomisation methods, which are an even more essential methodological component than blinding, are frequently used in Chinese studies^6–10^. But the authors do not report their methods exactly and—probably in part due to language problems—when contacted in our experience, they usually do not answer. In a meta-epidemiological study Panagiotou et al. ^25^ found that the effect sizes in less developed are higher than those in developed countries. In our experience, they are sometimes unplausibly high. This effect has been shown not only for studies from mainland China but also Iran^26^ and India^6^. It does, of course, not mean that all studies emerging from such countries are methodologically poor and that all studies from more experienced countries are good^27^, but problems must be expected more frequently in the former.

Our analysis has some limitations. First, while the currently largest meta-epidemiological study on the effects of blinding quoted above^1^ did not reveal bias, systematic reviews of such meta-epidemiological studies (“meta-meta-meta-analyses”) suggest that overall, the field is mixed with some studies finding and others not finding important effects of blinding^28–32^. The reasons for these discrepancies are unclear but may include the disease area studied, definitions of blinding, the number of available studies and meta-analyses, and methodological approaches.

Second, not a single comparison with placebo was available (there were some three-arm studies which had a placebo arm, but there was no corresponding open RCT with a placebo arm). In trials with placebo or no-treatment as comparators, the effects of blinding could be more important. In a related analysis of placebo-controlled antipsychotic drug trials, we assessed how often there are unblinding side effects^33^. In only 4 placebo-controlled trials, blinding was tested, and in all 4 of them, side-effects unblinded treatment. In a similar analysis of 154 placebo-controlled antidepressants, blinding success was assessed in only 16 (10%) trials, but patients and assessors were unlikely to judge treatment allocation^34^, and two analyses did not find that the occurrence of adverse events and, consequently, potential unblinding increased antidepressant-placebo differences^35,36^. In contrast, in a study not included in Lin et al.^34^, adolescents with depression were more likely than chance to guess the allocation to fluoxetine or placebo^37^. A Cochrane review found smaller antidepressant versus placebo effect sizes in RCTs using active placebos^38^. Testing for blinding should be done more frequently. In head-to-head trials, it is more difficult because side effects between drugs overlap. For this reason, we did not address this question in the current analysis. Moreover, in our experience, the effects of blinding are extremely rarely tested in head-to-head trials. However, an alternative explanation of our findings is that blinding of antipsychotics does not work well, so ultimately, blinded and non-blinded trials have the same findings.

Third, we emphasise that we did not compare study results based on the Risk of Bias tool version 1 (RoB 1)^3^ or version 2 (RoB 2)^39^, but based on their descriptions as open, single-blind (blinded raters) and double-blind (aiming at participants, carers and raters by using identical capsules). The reason was that both RoB 1 and RoB 2 asked the reviewer to make a judgement of the success of blinding, which is often speculative. For example, a study comparing olanzapine with ariprazole may be prone to unblinding because the drugs have quite different side effects. In contrast, a study comparing olanzapine with quetiapine may not because they have similar side effects. If we had chosen such an approach, the results might have been different but more speculative because side-effect differences are gradual.

Fourth, although we started out from 402 RCTs, for most comparisons, only a few trials were available, reducing statistical power. The reason was that we always needed at least one blinded and one non-blinded trial for each comparison. The major factor was the small number of open RCTs because there were only 28 open RCTs (with a median 28 lower number of participants) compared to 155 blinded RCTs with useable data). Participants in open trials were approximately 4 years younger, which may impact patients’s experience with an antipsychotic, and they had a 5 points lower PANSS total score (not-significant). Open trials had a median 4 years after publication year, which was associated with effect sizes in placebo-controlled trials^40^. The IQRs of the study duration were different, which could potentially impact discontinuation, efficacy, and side effects, and there may be other differences in terms of geography, trial population, and acuity of symptoms. Individual-patient data meta-analyses could give answers as to how these factors may interact and impact the differences between open and blinded RCTs.

Fifth, we used a relatively high alpha (*p*-value of 0.1) to indicate significant differences because subgroup tests are not very sensitive. Nevertheless, there could be an issue of multiple testing and false positives. Indeed, if *p* < 0.05 were used as a threshold, only 4 subgroup tests would be significant, 2 were below 0.01, and if a Bonferroni correction had been applied—which is not generally recommended for systematic reviews^8^—only one would have been below the adjusted significance level 0.0008 (0.1 divided by 122 subgroup tests).

Sixth, we assumed that trial results could be biased in favour of newer medications or those from pharmaceutical companies, but the individual investigators make the ratings, and these have a much smaller if any, conflict of interest.

In summary, we found no clear evidence that lack of blinding systematically biased the results of RCTs comparing antipsychotic drugs head-to-head, in particular in terms of their primary outcome overall symptoms of schizophrenia. However, there were some “outliers” where open RCTs showed clearly larger effects than blinded ones. Moreover, due to the limitations summarised above we consider our analysis exploratory and would currently not recommend practice changes. The analysis should be repeated once significantly more studies are available. Double-blind studies are methodologically more rigorous, but they are more expensive; they are not an option for all patients and thus reduce generalisability. It is possible that blinding attempts are not effective for antipsychotics. Open trials reflect better real-world settings, thus giving more information on effectiveness. Currently, designers of meta-analyses of antipsychotic drug trials can decide to exclude or include open RCTs. For example, in an area where few trials are available, open RCTs with good randomisation and allocation concealment methods may be included. However, even in such cases, open RCTs might be excluded in a sensitivity analysis. Overall, the inclusion of trials from less-developed countries such as China, Iran or India may produce more bias than the lack of double-blinding^6,10,25^. Given that approximately 30% of RCTs in schizophrenia currently come from China^41^, their impact on meta-analyses is more important than the relatively few open RCTs.

### Supplementary information

eAppendix

## Data Availability

We are planning to update this work. Thus, we do not plan to share the database used for the statistical analyses. Please contact the corresponding author if you would like to see any data that are not included in the Article or the appendix.
