# Supplementary material for: Are the results of open randomised controlled trials comparing antipsychotic drugs in schizophrenia biased? Exploratory meta- and subgroup analysis
Source: Schizophrenia (Heidelb). 2024 Feb 15;10(1):17. doi: 10.1038/s41537-024-00442-8 (PMC10866997; doi:10.1038/s41537-024-00442-8)

Content of eAppendix

- Prisma checklist
- eFigure 1 Prisma diagram
- eTable 1 Included studies table
- eTable 2 Risk of bias
- eFigure 2 Primary outcome
- eFigure 3 Sensitivity analysis of the primary outcome – fixed effects model
- eFigure 4 Sensitivity analysis of the primary outcome – single-blind studies excluded
- eFigure 5 Positive symptoms
- eFigure 6 Negative symptoms
- eFigure 7 All-cause discontinuation
- eFigure 8 Antiparkinson medication
- eFigure 9 Sedation
- eFigure 10 Weight gain
- eFigure 11 Prolactin

eAppendix

PRISMA checklist

PRISMA Checklist

Section/topic	#	Checklist item	Reported on page #
**TITLE**			
Title	1	Identify the report as a systematic review, meta-analysis, or both.	1
**ABSTRACT**			
Structured summary	2	Provide a structured summary including, as applicable: background; objectives; data sources; study eligibility criteria, participants, and interventions; study appraisal and synthesis methods; results; limitations; conclusions and implications of key findings; systematic review registration number.	3
**INTRODUCTION**			
Rationale	3	Describe the rationale for the review in the context of what is already known.	4
Objectives	4	Provide an explicit statement of questions being addressed with reference to participants, interventions, comparisons, outcomes, and study design (PICOS).	4+method
**METHODS**			
Protocol and registration	5	Indicate if a review protocol exists, if and where it can be accessed (e.g., Web address), and, if available, provide registration information including registration number.	
Eligibility criteria	6	Specify study characteristics (e.g., PICOS, length of follow-up) and report characteristics (e.g., years considered, language, publication status) used as criteria for eligibility, giving rationale.	5-7
Information sources	7	Describe all information sources (e.g., databases with dates of coverage, contact with study authors to identify additional studies) in the search and date last searched.	5
Search	8	Present full electronic search strategy for at least one database, including any limits used, such that it could be repeated.	5
Study selection	9	State the process for selecting studies (i.e., screening, eligibility, included in systematic review, and, if applicable, included in the meta-analysis).	5
Data collection process	10	Describe method of data extraction from reports (e.g., piloted forms, independently, in duplicate) and any processes for obtaining and confirming data from investigators.	5
Data items	11	List and define all variables for which data were sought (e.g., PICOS, funding sources) and any assumptions and simplifications made.	6
Risk of bias in individual studies	12	Describe methods used for assessing risk of bias of individual studies (including specification of whether this was done at the study or outcome level), and how this information is to be used in any data synthesis.	7
Summary measures	13	State the principal summary measures (e.g., risk ratio, difference in means).	7
Synthesis of results	14	Describe the methods of handling data and combining results of studies, if done, including measures of consistency (e.g., I^2) for each meta-analysis.	7

Section/topic	#	Checklist item	Reported on page #
Risk of bias across studies	15	Specify any assessment of risk of bias that may affect the cumulative evidence (e.g., publication bias, selective reporting within studies).	7
Additional analyses	16	Describe methods of additional analyses (e.g., sensitivity or subgroup analyses, meta-regression), if done, indicating which were pre-specified.	8
**RESULTS**			
Study selection	17	Give numbers of studies screened, assessed for eligibility, and included in the review, with reasons for exclusions at each stage, ideally with a flow diagram.	10 and PRISMA diagram and included and excluded studies table in supplement
Study characteristics	18	For each study, present characteristics for which data were extracted (e.g., study size, PICOS, follow-up period) and provide the citations.	Included studies table in supplement
Risk of bias within studies	19	Present data on risk of bias of each study and, if available, any outcome level assessment (see item 12).	8 and Risk of bias summary in supplement
Results of individual studies	20	For all outcomes considered (benefits or harms), present, for each study: (a) simple summary data for each intervention group (b) effect estimates and confidence intervals, ideally with a forest plot.	Figures 1 and 2, and eFigures
Synthesis of results	21	Present results of each meta-analysis done, including confidence intervals and measures of consistency.	Figures 1 and 2, and eFigures
Risk of bias across studies	22	Present results of any assessment of risk of bias across studies (see Item 15).	8 and risk of bias graph in summary

Additional analysis	23	Give results of additional analyses, if done (e.g., sensitivity or subgroup analyses, meta-regression [see Item 16]).	9 and supplement
**DISCUSSION**			
Summary of evidence	24	Summarize the main findings including the strength of evidence for each main outcome; consider their relevance to key groups (e.g., healthcare providers, users, and policy makers).	12
Limitations	25	Discuss limitations at study and outcome level (e.g., risk of bias), and at review-level (e.g., incomplete retrieval of identified research, reporting bias).	14-15
Conclusions	26	Provide a general interpretation of the results in the context of other evidence, and implications for future research.	12-16
**FUNDING**			
Funding	27	Describe sources of funding for the systematic review and other support (e.g., supply of data); role of funders for the systematic review.	16

From: Moher D, Liberati A, Tetzlaff J, Altman DG, The PRISMA Group (2009). Preferred Reporting Items for Systematic Reviews and Meta-Analyses: The PRISMA Statement. PLoS Med 6(6): e1000097. doi:10.1371/journal.pmed1000097

For more information, visit: **www.prisma-statement.org**.

eFigure 1

PRISMA diagram

eFigure1

PRISMA diagram of the search

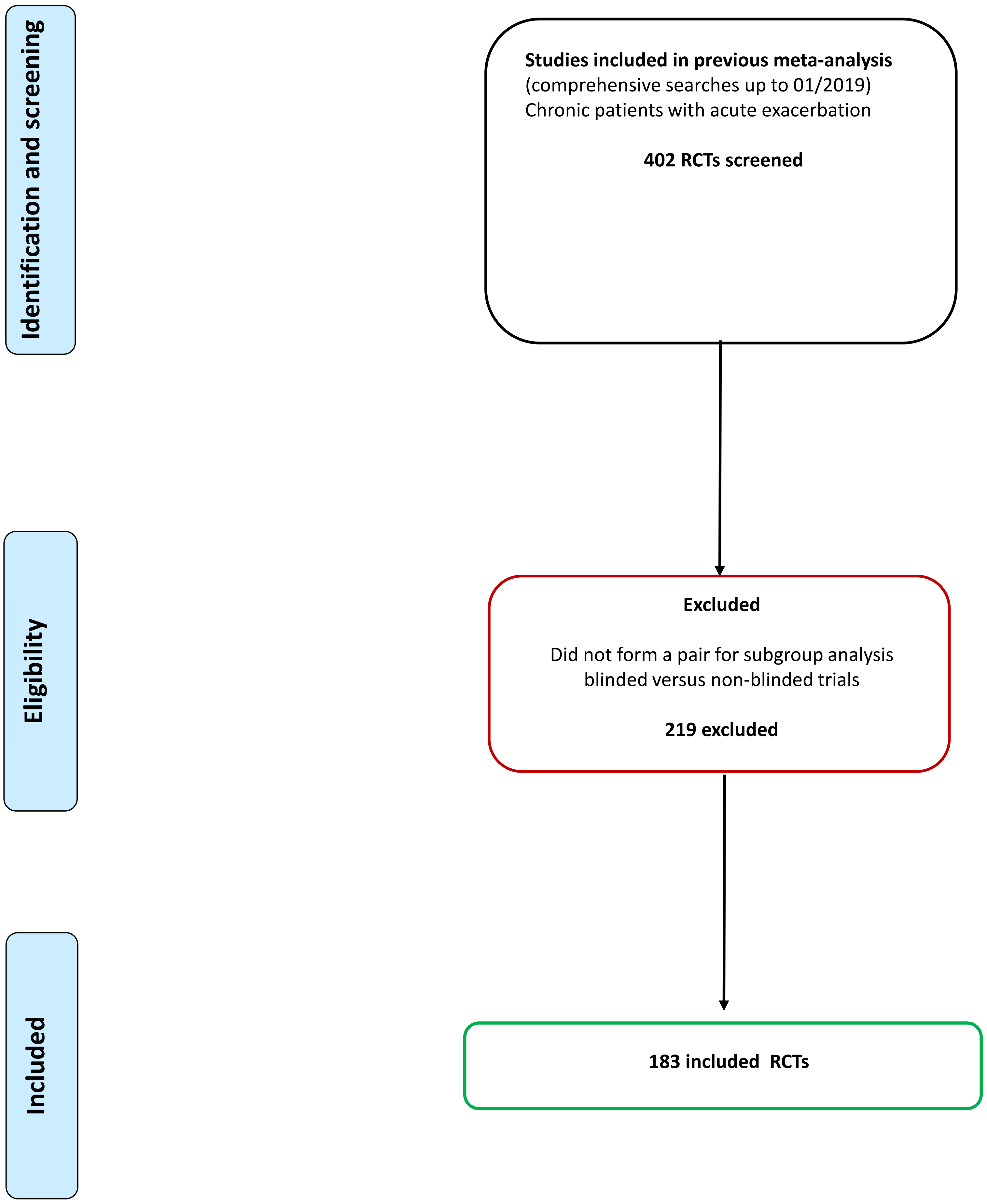

Reference

1. Huhn, M., Nikolakopoulou, A., Schneider-Thoma, J., Krause, M., Samara, M., Peter, N., Arndt, T., Bäckers, L., Rothe, P., Cipriani, A., Davis, J., Salanti, G., & Leucht, S. (2019). Comparative efficacy and tolerability of 32 oral antipsychotics for the acute treatment of adults with multi-episode schizophrenia: a systematic review and network meta-analysis. *Lancet (London, England)*, *394*(10202), 939–951

eTable 1

Characteristics of included studies

eTable1: Characterists of included studies

Study	Drug	N	Mean dose	Mean age	Year	Duration	Blinding type	Diagnostic criteria	Diagnostic term
A1281046[1]	Risperidone	29	5	-	2005	12	double-blind	DSM-III-R	schizophrenia, schizoaffective and schizophreniform disorder
	Ziprasidone	29	120	-					
A1281050[2]	Haloperidol	122	12,4	-	2004	6	single-blind	DSM-IV	schizophrenia or schizoaffective disorder
	Ziprasidone	130	108,1	-					
Abdolahian 2008[3]	Haloperidol	30	12,5	-	2008	8	double-blind	DSM-IV	chronic schizophrenia
	Risperidone	35	6	-					
	Tiotixene	28	31,8	34					
Addington 2004[4]	Risperidone	147	7,4	33,89	2004	8	double-blind	DSM-III-R	acute exacerbation of schizophrenia or schizoaffective disorder
	Ziprasidone	149	114,2	35,1					
Allan 1998[5]	Haloperidol	10	10	56	1998	6	double-blind	Clinical diagnosis	schizophrenia
	Olanzapine	13	20	50					
Arvanitis 1997[6]	Haloperidol	52	12	37	1997	6	double-blind	DSM-III-R	acute exacerbation of chronic or subchronic schizophrenia
	Placebo	51	0	36					
	Quetiapine	105	677	36,94					
Atmaca 2002[7]	Haloperidol<br>Risperidone	17<br>18	10(10-10)<br>600(600-600)	29.4<br>27.6	2002	6	open	DSM-IV	Schizophrenia
Avasthi 2001[8]	Haloperidol<br>Olanzapine	10<br>17	12.5 (5-20)<br>12.5 (5-20)	n.i.	2001	12	open	DSM-IV	Schizophrenia
Azorin 2006[9]	Risperidone	89	6,6	35,37	2006	12	double-blind	DSM-IV	schizophrenia of the paranoid, disorganized, catatonic, or undifferentiated type
	Sertindole	98	16,2	35,6					
	Placebo	50	0	36					
Beasley 1996b[10]	Haloperidol	69	16,4	36	1996	6	double-blind	DSM-III-R	schizophrenia with an acute exacerbation
	Olanzapine	133	14,04	36,5					
	Placebo	68	0	35					
Beasley 1997[11]	Haloperidol	81	17,6	36	1997	6	double-blind	DSM-III-R	

Study	Drug	N	Mean dose	Mean age	Year	Duration	Blinding type	Diagnostic criteria	Diagnostic term
	Olanzapine	175	13,88	36,51					schizophrenia with acute exacerbation
Bernardo 2001[12]	Haloperidol	13	10	29,9	2001	4	double-blind	DSM-IV	schizophrenia or schizophreniform disorder, acute psychosis
	Olanzapine	14	10	26,9					
Blin 1996[13]	Haloperidol	20	7,6	33,9	1996	4	double-blind	DSM-III-R	acute exacerbation of schizophrenia
	Levomepromazine	21	100	34,2					
	Risperidone	21	7,4	34,8					
Borison 1992[14]	Haloperidol	53	15	38,4	1992	6	double-blind	DSM-III-R	acute exacerbation schizophrenia
	Placebo	54	0	40,3					
	Risperidone	53	7,8	39,7					
Boulay 2007[15]	Haloperidol	13	9,32	34,73	2007	8	double-blind	DSM-IV	schizophrenia or schizoaffective disorder
	Olanzapine	14	12	32,86					
Brook 1998[16]	Haloperidol	20	10	28,8	1998	4	double-blind	Clinical diagnosis	schizophrenia or schizophreniform disorder
	Zuclopenthixol	24	25	32,3					
Brook 2005[17]	Haloperidol	138	11,5	34,6	2005	6	single-blind	DSM-IV	schizophrenia or schizoaffective disorder, acute exacerbation
	Ziprasidone	429	116	34					
Bueno 1979[18]	Haloperidol	20	5,4	29,21	1979	6	double-blind	Clinical diagnosis	schizophrenia
	Loxapine	20	58,3	28,42					
Canive 2006[19]	Olanzapine	5	15	41,7	2006	8	double-blind	DSM-IV	schizophrenia
	Risperidone	4	6	43					
Cavallaro 2001[20]	Haloperidol	16	6,5	23,2	2001	6	double-blind	DSM-III-R	subchronic schizophrenia
	Risperidone	17	6,25	25,4					
Ceskova 1993[21]	Haloperidol	31	6,4	38,7	1993	8	double-blind	ICD-9	acute schizophrenia or schizoaffective psychosis
	Risperidone	31	6	33					
Chan 2007[22]	Aripiprazole	49	15	35,2	2007	4	double-blind	DSM-IV	schizophrenia or schizoaffective disorder with an acute relapse
	Risperidone	34	6	35,1					
Chen 2018	Olanzapine	53	n.i.(10-20)	35.6	2018	12	Open	DSM-IV	schizophrenia

Study	Drug	N	Mean dose	Mean age	Year	Duration	Blinding type	Diagnostic criteria	Diagnostic term
	Paliperidone	30	n.i.(6-12)	36.2					
	Risperidone	28	n.i.(4-6)	35					
Chouinard 1993[23]	Haloperidol	21	20	37	1993	8	double-blind	DSM-III-R	chronic schizophrenia
	Placebo	22	0	37					
	Risperidone	22	6	37					
Chung 2000[24, 25]	Haloperidol	15	10.7 (9-15)	30.1	2000	6	open	DSM-IV	schizophrenia, schizophreniform disorder, schizoaffective disorder, delusional disorder, brief psychotic disorder
	Risperidone	18	4.8 (n.i.-n.i.)	31.3					
Chung 2012[25]	Olanzapine	60	16.6 (n.i.-n.i.)	34.9	2012	12	open	DSM-IV-TR	schizophrenia
	Paliperidone	30	7.6 (n.i.-n.i.)	36.3					
	Risperidone	30	4.6 (n.i.-n.i.)	35.5					
Citrome 2015[26]	Aripiprazole	33	18.2 (10-20)	42.1	2016	12	open	DSM-IV-TR	schizophrenia
	Brexpiprazole	64	3.6 (1-4)	41.2					
Clark 1970a[27]	Chlorpromazine	15	684	38,9	1970	12	double-blind	Clinical diagnosis	chronic schizophrenia
	Molindone	15	68,2	42,5					
	Placebo	14	0	37,4					
Clark 1972[28]	Chlorpromazine	19	816,71	41,8	1972	12	double-blind	Clinical diagnosis	chronic schizophrenia
	Loxapine	18	80,62	41,2					
	Placebo	18	0	39,9					
Clark 1975[29]	Loxapine	15	71	35,7	1975	4	double-blind	Clinical diagnosis	chronic schizophrenia
	Placebo	13	0	36,9					
	Trifluoperazine	15	36	41,4					
Claus 1992[30]	Haloperidol	22	10,3	39	1992	12	double-blind	DSM-III-R	schizophrenia with chronic course
	Risperidone	22	12	37,4					
Conley 2001[31]	Olanzapine	189	13,1	38,9	2001	8	double-blind	DSM-IV	schizophrenia or schizoaffective disorder
	Risperidone	188	4,7	41					
Cooper 2000a[32]	Chlorpromazine	53	532,08	41	2000	8	double-blind	DSM-III-R	

Study	Drug	N	Mean dose	Mean age	Year	Duration	Blinding type	Diagnostic criteria	Diagnostic term
	Placebo	53	0	36,3					acute episode of schizophrenia or acute exacerbation of (sub-) chronic schizophrenia
	Zotepine	53	240,57	39,6					
Copolov 2000[33]	Haloperidol	227	8	37	2000	6	double-blind	DSM-III-R	acute exacerbation of chronic or subchronic schizophrenia
	Quetiapine	221	455	37					
Corripio 2005[34]	Haloperidol	10	12,5	36	2005	-	single-blind	DSM-IV	schizophreniform disorders or schizophrenia with acute psychosis exacerbation
	Ziprasidone	10	100	30,7					
Costa e Silva 1989[35]	Amisulpride	20	850	35,2	1989	3	double-blind	ICD-9	schizophrenia
	Haloperidol	20	28	35,5					
Cutler 2008[36]	Iloperidone	303	24	39,5	2008	4	double-blind	DSM-IV	schizophrenia
	Placebo	152	0	40,7					
	Ziprasidone	151	160	40					
Davidson 2007[37]	Olanzapine	128	10	36,5	2007	6	double-blind	DSM-IV	schizophrenia acute episode
	Paliperidone	125	9	36,2					
	Placebo	123	0	37,3					
Dieterle 1991[38]	Perazine	20	350	31,1	1991	4	double-blind	ICD-9	schizophrenia or schizoaffective disorder
	Zotepine	20	240	35,8					
Dossenbach 2007[39]	Chlorpromazine	40	433.1 (200-800)	33,4	2007	6	open	DSM-IV	schizophrenia
	Olanzapine	83	14.9 (5-20)	30,9					
Duggan 2005[40]	Haloperidol	51	10.8 (1.5-20)	n.i.	2005	6	open	DSM-IV	schizophrenia, schizophreniform and schizoaffective disorder
	Olanzapine	53	12.5 (5-20)	n.i.					
Durgam 2014[41]	Cariprazine	438	3,0	36,564 38356	2014	6	double-blind	DSM-IV-TR	acute exacerbation of schizophrenia
	Placebo	151	0	36					
	Risperidone	140	4	36,5					
Durgam 2015[42]	Aripiprazole	152	10	39,3	2015	6	double-blind	DSM-IV-TR	schizophrenia
	Cariprazine	312	4,51	38,252 24359					

Study	Drug	N	Mean dose	Mean age	Year	Duration	Blinding type	Diagnostic criteria	Diagnostic term
	Placebo	153	0	38,2					
Ehrlich 2012[43]	Olanzapine	20	14.9 (7.5-30)	32.1	2012	12	open	DSM-IV	Schizophrenia or schizoaffective (DSM-IV)
	Ziprasidone	20	102.6 (60-160)	30.6					
Fakra 2008[44]	Haloperidol	15	8 (n.i.-n.i.)	37.8	2008	4	open	DSM-IV	Schizophrenia or schizoaffective disorder
	Risperidone	15	5.8 (n.i.-n.i.)	33.9					
Fleischhacker 1989[45]	Haloperidol	20	14,5	32,5	1989	6	double-blind	DSM-III	paranoid schizophrenia
	Zotepine	20	309	34,7					
Fleischhacker 2009[46]	Aripiprazole	355	23	35,9	2009	52	double-blind	DSM-IV	schizophrenia, acute relapse
	Olanzapine	348	15,4	37,3					
Freeman 1969[47]	Molindone	20	80	43	1969	8	double-blind	Clinical diagnosis	schizophrenia
	Trifluoperazine	20	40	42					
Gattaz 2004[48]	Flupentixol	13	12,5	33,3	2004	4	double-blind	DSM-IV	schizophrenia
	Olanzapine	15	12,5	31,5					
Gelenberg 1979[49]	Chlorpromazine	8	606	29,6	1979	4	double-blind	DSM-II	schizophrenia
	Clozapine	7	279	30,8					
Gerlach 1975[50]	Clozapine	4	225	49,75	1975	3	double-blind	Clinical diagnosis	schizophrenia
	Haloperidol	4	9	49,75					
Ghaleiha 2011[51]	Clozapine	17	300	37,12	2011	8	double-blind	DSM-IV-TR	chronic schizophrenia
	Haloperidol	17	15	33					
	Risperidone	17	6	32,06					
Goff 1998[52]	Haloperidol	17	15	35,5	1998	4	double-blind	DSM-III-R	chronic or subchronic schizophrenia or schizoaffective disorder
	Ziprasidone	20	160	41,7					
Gowardman 1973[53]	Haloperidol	10	10,15	52,2	1973	13	double-blind	Clinical diagnosis	schizophrenia
	Pimozide	10	5,1	50,2					
Guirguis 1977[54]	Chlorpromazine	28	525	34,7	1977	7	double-blind	Clinical diagnosis	acute schizophrenia
	Clozapine	22	262,5	41,5					

Study	Drug	N	Mean dose	Mean age	Year	Duration	Blinding type	Diagnostic criteria	Diagnostic term
Hale 2000[55]	Haloperidol	125	10	36,5	2000	8	double-blind	DSM-III-R	schizophrenia
	Sertindole	255	18	34,75					
Harnryd 1984[56]	Chlorpromazine	25	400	27	1984	8	double-blind	Clinical diagnosis	schizophrenia
	Sulpiride	25	800	27					
Hatta 2009[57]	Aripiprazole	22	23,6	42,1	2009	8	single-blind	ICD-10	schizophrenia, acute schizophrenia-like psychotic disorder, schizoaffective disorder
	Olanzapine	17	17,4	39,8					
	Quetiapine	20	579	39,8					
	Risperidone	21	7,2	41,1					
Hatta 2013[58]	Olanzapine	22	23	39,2	2013	8	single-blind	DSM-IV-TR	schizophrenia, schizophreniform disorder, schizoaffective disorder
	Risperidone	20	6,9	34,5					
Heikkila 1981[59]	Haloperidol	33	10	44,3	1981	12	double-blind	Clinical diagnosis	schizophrenia or other psychosis
	Zuclopenthixol	30	40	41					
Heikkilae 1992[60]	Haloperidol	23	10,3	38	1992	8	double-blind	ICD-9	acute schizophrenia, exacerbation of chronic schizophrenia, paranoid states, reactive paranoid psychosis
	Zuclopenthixol	26	33,5	35					
Hera 041-021[61]	Asenapine	208	14,9	40,79	2009	6	double-blind	DSM-IV	acute exacerbation of schizophrenia
	Olanzapine	103	15	39,7					
	Placebo	106	0	39,5					
Hera 041-022[62]	Asenapine	91	15	44	2009	6	double-blind	DSM-IV	acute exacerbation of schizophrenia
	Olanzapine	93	15	41,6					
	Placebo	93	0	41,9					
Hirayasu 2010[63]	Olanzapine	47	10	46,2	2007	6	double-blind	DSM-IV	schizophrenia with acute symptoms
	Paliperidone	136	6	44					
	Placebo	138	0	46,2					
Honigfeld 1984c[64]	Clozapine	39	397	-	1984	5,7	double-blind	Clinical diagnosis	schizophrenia
	Haloperidol	40	7,6	-					
Honigfeld 1984d[64]	Chlorpromazine	113	360	34	1984	5,7	double-blind	ICD-9	schizophrenia

Study	Drug	N	Mean dose	Mean age	Year	Duration	Blinding type	Diagnostic criteria	Diagnostic term
	Clozapine	110	310	34					
Hoyberg 1993[65]	Perphenazine	52	28	35	1993	8	double-blind	DSM-III-R	chronic schizophrenia with acute exacerbation
	Risperidone	55	8,5	38					
Huttunen 1995[66]	Risperidone	48	8	34	1995	6	double-blind	DSM-III-R	acute exacerbation of schizophrenia or schizophreniform disorder
	Zuclopenthixol	50	38	38					
Hwang 2001[67]	Haloperidol	35	9	33,7	2001	6	double-blind	ICD-10	schizophrenia
	Zotepine	35	150	34,2					
Hwang 2003[68]	Amisulpride	23	630	36,3	2003	6	double-blind	DSM-IV	schizophrenia
	Risperidone	25	6,88	34,1					
Hwang 2012[69]	Aripiprazole	49	15	-	2012	4	double-blind	DSM-IV	schizophrenia or schizoaffective disorder
	Risperidone	34	6	-					
Ingole 2009[70]	Olanzapine	30	10	25.3	2009	12	open	DSM-IV	schizophrenia or schizoaffective disorder
	Risperidone	30	6	26.6					
Ishigooka 2001[70]	Haloperidol	89	7,36	42,9	2001	8	double-blind	ICD-10	schizophrenia
	Olanzapine	93	10,31	42,9					
Itil 1971[71]	Molindone	30	85	28,5	1971	12	double-blind	Clinical diagnosis	schizophrenia
	Trifluoperazine	30	85	27,8					
Itoh 1977[72]	Clozapine	47	287,5	-	1977	12	double-blind	Clinical diagnosis	schizophrenia
	Haloperidol	41	8,625	-					
Janicak 2001[73]	Haloperidol	32	10,8	42	2001	6	double-blind	DSM-IV	schizoaffective disorder
	Risperidone	30	5,5	43					
Jindal 2013[74]	Aripiprazole	30	12,5	-	2013	6	double-blind	ICD-10	schizophrenia
	Olanzapine	30	11,01	-					
Kane 2002[75]	Aripiprazole	204	22,5	38,55	2002	4	double-blind	DSM-IV	schizophrenia or schizoaffective disorder, acute relapse
	Haloperidol	104	10	38,9					
	Placebo	106	0	38,5					
Kane 2007b[76]	Olanzapine	128	10	36,3	2007	6	double-blind	DSM-IV	acute episode of schizophrenia
	Paliperidone	375	9,1	37,14					

Study	Drug	N	Mean dose	Mean age	Year	Duration	Blinding type	Diagnostic criteria	Diagnostic term
	Placebo	127	0	37,9					
Kane 2009[77]	Aripiprazole	285	18,85	38,2	2009	28	double-blind	DSM-IV-TR	schizophrenia
	Olanzapine	281	16,37	39,3					
Kane 2010a[78]	Asenapine	220	14,9	38,5	2010	6	double-blind	DSM-IV-TR	schizophrenia acute exacerbation
	Haloperidol	115	8	38,5					
	Placebo	123	0	38,5					
Kingstone 1970[79]	Chlorpromazine	21	435 (150-1800)	30.8	1978	3	double-blind	Clinical diagnosis	acute psychotic patients (clinical diagnosis)
	Clopenthixol	20	122 (75-600)	31					
Kluge 2007[80]	Clozapine	15	266,7	36,7	2007	6	double-blind	DSM-IV	schizophrenia, schizophreniform or schizoaffective disorder
	Olanzapine	15	21,2	32,8					
Knegtering 2004[81]	Quetiapine	25	580 (200-1200)	26.5	2004	6	open	DSM-IV	schizophrenia, schizophreniform or schizoaffective disorder
	Risperidone	26	3.2 (1-6)	25.2					
Knegtering 2006[82]	Olanzapine	25	9.4 (5-15)	27.2	2006	6	open	DSM-IV	schizophrenia, schizophreniform or schizoaffective disorder
	Risperidone	21	3.4 (2-6)	26					
Kramer 1978[83]	Loxapine	29	78	30,4	1978	4	double-blind	DSM-II	schizophrenia
	Thioridazine	27	526	32,9					
Kwon 2012[84]	Olanzapine	196	15	34,9	2010	12	double-blind	DSM-IV-TR	chronic schizophrenia
	Sertindole	198	16	34,4					
Lahti 2009[85]	Haloperidol	14	10,4	38,3	2009	6	double-blind	DSM-IV	schizophrenia
	Olanzapine	18	15,9	36,1					
Lamure 2003[86]	Haloperidol	45	n.i.	38.8	2003	6	open	DSM-III-R	schizophrenia
	Zuclopenthixol	43	n.i.	38.6					
Landbloom 2016[87]	Asenapine	113	10	39,1	2015	6	double-blind	DSM-IV-TR	schizophrenia of paranoid, disorganized, or undifferentiated subtype
	Olanzapine	46	15	40,8					
	Placebo	103	0	41,4					

Study	Drug	N	Mean dose	Mean age	Year	Duration	Blinding type	Diagnostic criteria	Diagnostic term
Li 2012[88]	Quetiapine	60	630,47	35,4	2012	6	single-blind	DSM-IV	schizophrenia
	Risperidone	59	3,57	35,4					
Li 2017[89]	Chlorpromazine	192	450	32	2012	6	double-blind	Clinical diagnosis	schizophrenia
	Quetiapine	196	600	32,9					
Lieberman 2005[90]	Olanzapine	336	20,1	40,8	2005	78	double-blind	DSM-IV	schizophrenia
	Perphenazine	261	20,8	40					
	Quetiapine	337	543,4	40,9					
	Risperidone	341	3,9	40,6					
	Ziprasidone	185	112,8	40,1					
Lin 2003[91]	Clozapine	24	377,1	-	2003	12	single-blind	DSM-IV	schizophrenia
	Zotepine	35	397,1	-					
Liu 2000[92]	Haloperidol	28	-	35,1	2000	12	double-blind	DSM-III-R	schizophrenic disorders
	Risperidone	28	-	32,7					
Loebel 2013[93]	Lurasidone	246	119,35	37,04	2010	6	double-blind	DSM-IV-TR	acute exacerbation of schizophrenia
	Placebo	122	0	37,4					
	Quetiapine	120	600	37,4					
Loza 2006[94]	Olanzapine	39	12,5	-	2006	8	double-blind	Clinical diagnosis	schizophrenia
	Risperidone	40	5	-					
Lublin 1991[95]	Haloperidol	7	6,2	-	1991	3	single-blind	Clinical diagnosis	schizophrenia
	Zuclopenthixol	8	16,5	-					
McCue 2006[96]	Aripiprazole	63	21.8 (n.i.-n.i.)	40.5	2006	3	open	DSM-IV	schizophrenia, schizoaffective disorder or schizophreniform disorde
	Haloperidol	61	16 (n.i.-n.i.)	35.7					
	Olanzapine	58	19.1 (n.i.-n.i.)	33.8					
	Quetiapine	62	652.5 (n.i.-n.i.)	39					
	Risperidone	65	5.2 (n.i.-n.i.)	38.6					
	Ziprasidone	59	151.2 (n.i.-n.i.)	38.3					

Study	Drug	N	Mean dose	Mean age	Year	Duration	Blinding type	Diagnostic criteria	Diagnostic term
Maat 2014[97]	Aripiprazole	38	17 (7.5-30)	26.4	2014	8	open	DSM-IV-TR	schizophrenia
	Risperidone	42	3.5 (1-6)	24.8					
Marder 1994[98]	Haloperidol	66	20	38	1994	8	double-blind	DSM-III-R	schizophrenia
	Placebo	66	0	37,1					
	Risperidone	64	6	37,5					
Marder 2007c[99]	Olanzapine	110	10	40,5	2007	6	double-blind	DSM-IV	acute exacerbation of schizophrenia
	Paliperidone	224	9	41,3					
	Placebo	110	0	42,3					
McQuade 2004_6weeks[100]	Aripiprazole	156	25,1	38,6	2004	6	double-blind	DSM-IV	schizophrenia, acute relapse
	Olanzapine	161	16,5	38,2					
Meltzer 2011[101]	Lurasidone	239	79,8	37,8	2010	6	double-blind	DSM-IV	acute exacerbation of schizophernia
	Olanzapine	123	15	38,3					
	Placebo	116	0	37					
Mesotten 1991[102]	Haloperidol	32	11	40	1991	8	double-blind	DSM-III	schizophrenia, other serious psychotic disorders
	Risperidone	28	11	40					
Meyer-Lindenberg 1997[103]	Clozapine	25	300	33,2	1997	6	double-blind	DSM-III-R	schizophrenia
	Zotepine	25	300	33,7					
Min 1993[104]	Haloperidol	19	8,9	34,1	1993	8	double-blind	DSM-III-R	chronic schizophrenic disorder
	Risperidone	16	7,5	34,1					
Mirabzadeh 2014[105]	Haloperidol	29	15 (15-15)	37.4	2014	8	open	DSM-IV-TR	schizophrenic disorder
	Risperidone	37	6 (6-6)	37.4					
Möller 1997[106]	Amisulpride	95	700	36	1997	6	double-blind	DSM-III-R	chronic or subchronic schizophrenia
	Haloperidol	96	17,5	35					
Moosavi 2015[107]	Quetiapine	45	498	35,5	2015	4	double-blind	DSM-IV-R	schizophrenia or schizophreniform disorder
	Risperidone	45	5,6	34,3					
Mori 2004[108]	Olanzapine	20	16,5	59,9	2004	8	double-blind	DSM-IV	schizophrenia

Study	Drug	N	Mean dose	Mean age	Year	Dura tion	Blinding type	Diagnostic criteria	Diagnostic term
	Quetiapine	20	432,5	59,9					
	Risperidone	19	7,37	59,9					
Mortimer 2004[109]	Amisulpride	189	504	38,2	2004	26	double-blind	DSM-IV	schizophrenia or schizophreniform disorder
	Olanzapine	188	13	37,4					
Moyano 1975[110]	Loxapine	25	50	45,5	1975	12	double-blind	Clinical diagnosis	schizophrenia
	Trifluoperazine	24	30	48,5					
Murasaki 1993[111]	Haloperidol	95	5,8	40	1993	8	double-blind	ICD-9, DSM-III-R	schizophrenia
	Risperidone	97	6,1	44					
Murasaki 2001[112]	Haloperidol	97	6,7	44,1	2001	8	double-blind	ICD-10	schizophrenia
	Quetiapine	100	226	45,8					
NCT00350467[113]	Olanzapine	145	10	-	2008	6	double-blind	DSM-IV	schizophrenia
	Paliperidone	141	7,5	-					
NCT00905307[114]	Aripiprazole	50	15	40,8	2015	6	double-blind	DSM-IV-TR	schizophrenia
	Brexpiprazole	90	2,5	37,4					
	Placebo	95	0	38,8					
NCT01810380[115]	Brexpiprazole	150	-	39,68	2016	6	double-blind	DSM-IV-TR	schizophrenia
	Placebo	163	-	40,85					
	Quetiapine	154	-	41,12					
Nam 2004[116]	Clozapine	20	290 (n.i.-n.i.)	38.9	2004	12	open	DSM-IV	schizophrenia
	Haloperidol	20	17 (n.i.-n.i.)	37.9					
Nishizono 1994[117]	Chlorpromazine	52	225	-	1994	4	double-blind	-	schizophrenia
	Haloperidol	57	10,5	-					
	Zotepine	60	225	-					
Nistico 1974[118]	Penfluridol	20	40	-	1974	6	double-blind	Clinical diagnosis	hospitalized chronic schizophrenics
	Placebo	20	0	-					
Ozguven 2004[119]	Olanzapine	15	23	38,8	2004	6	single-blind	DSM-IV	schizophrenia, schizoaffective disorder, schizophreniform disorder
	Quetiapine	19	826,67	33,9					

Study	Drug	N	Mean dose	Mean age	Year	Dura tion	Blinding type	Diagnostic criteria	Diagnostic term
Petit 1996[120]	Haloperidol	63	15,7	36	1996	8	double-blind	DSM-III-R	acute exacerbation of schizophrenia
	Zotepine	63	241,5	38,6					
Peuskens 1995[121]	Haloperidol	226	10	38,1	1995	8	double-blind	DSM-III-R	chronic schizophrenia
	Risperidone	457	6,01	37,848					
Peuskens 1997[122]	Chlorpromazine	100	384	34	1997	6	double-blind	DSM-III-R	acute exacerbation of chronic/subchronic schizophrenia, or schizophreniform disorder
	Quetiapine	101	407	32					
Peuskens 1999[123]	Amisulpride	115	800	36	1999	8	double-blind	DSM-IV	acute exacerbation of schizophrenia
	Risperidone	113	8	37					
Potkin 2003[124]	Aripiprazole	202	25	39,15	2003	4	double-blind	DSM-IV	schizophrenia or schizoaffective disorder
	Placebo	103	0	38,8					
	Risperidone	99	6	38,6					
Potkin 2007c[125]	Asenapine	60	10	38	2007	6	double-blind	DSM-IV	acute exacerbation of schizophrenia
	Placebo	62	0	42					
	Risperidone	60	6	43					
Potkin 2008a[126]	Haloperidol	124	15	39,1	2008	6	double-blind	DSM-IV	acute exacerbation of schizophrenia
	Iloperidone	124	12	40,1					
	Placebo	127	0	39,3					
Potkin 2008b[126]	Iloperidone	154	14,58	39,3	2008	6	double-blind	DSM-IV	schizophrenia and schizoaffective disorder
	Placebo	156	0	38,8					
	Risperidone	153	7,02	37,5					
Potkin 2008c[126]	Iloperidone	389	18	38,3	2008	6	double-blind	DSM-IV	acute exacerbation of schizophrenia and schizoaffective disorder
	Placebo	160	0	39					
	Risperidone	157	7,09	39,8					
Potkin 2015[127]	Haloperidol	73	10	40	2010	6	double-blind	DSM-IV	acute exacerbation of schizophrenia
	Lurasidone	140	60,3	42,1					
	Placebo	72	0	41					
	Olanzapine	34	12,4	-	2004	12	double-blind	DSM-IV	

Study	Drug	N	Mean dose	Mean age	Year	Duration	Blinding type	Diagnostic criteria	Diagnostic term
Protocol ZIP-NY-97-019[128]	Ziprasidone	33	105,7	-					schizophrenia or schizoaffective disorder
Puech 1998[129]	Amisulpride	129	600	36,15	1998	4	double-blind	DSM-III-R	chronic or subchronic schizophrenia with acute exacerbation
	Haloperidol	64	16	36,6					
Rickels 1978[130]	Chlorpromazine	40	926	30,4	1978	3	double-blind	Clinical diagnosis	schizophrenic
	Tiotixene	39	44,3	31,3					
Riedel 2007[131]	Olanzapine	26	15,82	34,47	2007	8	double-blind	DSM-IV	schizophrenia, acute episode
	Quetiapine	26	586,86	36,69					
Rifkin 1984[132]	Chlorpromazine	33	1288	26,3	1984	4	double-blind	Clinical diagnosis	schizophrenia
	Loxapine	31	128,6	26,8					
Rosenheck 2003_6 weeks[133]	Haloperidol	150	11,2	46,2	2003	6	double-blind	DSM-IV	schizophrenia, schizoaffective disorder
	Olanzapine	159	11,4	46,8					
Rüther 1988[134]	Amisulpride	15	482	36,5	1988	4	double-blind	ICD-9	schizophrenia (295,1-295,7)
	Perazine	15	476	34,4					
Sacchetti 2008[135, 136]	Olanzapine	25	15,1	35	2008	8	single-blind	DSM-IV-TR	schizophrenia
	Quetiapine	25	590	39					
	Risperidone	25	5,1	43					
Safa 2008[137]	Olanzapine	32	17,5	32,6	2008	12	double-blind	DSM-IV-TR	schizophrenia or schizoaffective disorder
	Risperidone	31	9	32,6					
Schennach 2018[138]	Haloperidol	58	-	36,96	2018	4	double-blind	DSM-IV	schizophrenia
	Ziprasidone	54	-	36,96					
See 1999[139]	Haloperidol	10	n.i. (15-30)	37.7	1999	5	double-blind	DSM-IV	schizophrenia
	Risperidone	10	n.i. (4-6)	33.5					
Selman 1976[140]	Haloperidol	29	8,8	32,8	1976	12	double-blind	Clinical diagnosis	acute exacerbation of chronic schizophrenia
	Loxapine	29	110	31,8					
	Placebo	29	0	34,2					
Sergi 2007[141]	Haloperidol	20	8	50	2007	8	double-blind	DSM-IV	

Study	Drug	N	Mean dose	Mean age	Year	Duration	Blinding type	Diagnostic criteria	Diagnostic term
	Olanzapine	40	15	49,2					schizophrenia, schizoaffective disorder
	Risperidone	40	4	48,2					
Shah 2011[142]	Olanzapine	105	12,5	34,27	2011	6	double-blind	DSM-IV	schizophrenia
	Paliperidone	109	7,5	33,37					
Simpson 2004[143]	Olanzapine	133	11,3	37,6	2004	6	double-blind	DSM-IV	schizophrenia or schizoaffective disorder
	Ziprasidone	136	129,9	37,7					
Sonmez 2009[144]	Risperidone	11	6 (4-8)	34.8	2009	6	open	DSM-IV-TR	schizophrenia or schizoaffective disorder
	Ziprasidone	11	120 (80-160)	34.5					
Study 115 2000[145]	Haloperidol	85	15	38,8	2000	6	double-blind	DSM-III-R	(sub-)chronic schizophrenia
	Placebo	83	0	38,9					
	Ziprasidone	164	161,9512195	38,95					
Study 128-301 1997[146]	Haloperidol	238	15	-	1997	12	double-blind	DSM-III-R	schizophrenia or schizoaffective disorder
	Ziprasidone	243	120	-					
Study 3001[147]	Haloperidol	146	11,9	37,3	2009	6	double-blind	DSM-IV	schizophrenia or schizoaffective disorder
	Iloperidone	454	11,4	37,3					
Study 3002[147]	Haloperidol	137	14	33,7	2009	6	double-blind	DSM-IV	schizophrenia or schizoaffective disorder
	Iloperidone	420	12,9	33,7					
Study 3003[147]	Haloperidol	122	14,5	36,1	2009	6	double-blind	DSM-IV	schizophrenia or schizoaffective disorder
	Iloperidone	365	13,3	36,1					
Study 93202 2002[148]	Aripiprazole	34	30	33	2002	4	double-blind	DSM-III-R	schizophrenia acute relapse
	Haloperidol	34	20	38,8					
	Placebo	35	0	37,9					
Study 94202 2002[149]	Aripiprazole	61	30	38,8	2002	4	double-blind	DSM-IV	schizophrenia acute relapse
	Haloperidol	63	10	38,91					
	Placebo	64	0	38,02					
Svestka 1990[150]	Haloperidol	18	11	33	1990	8	double-blind	ICD-9	schizophrenia or schizoaffective disorder
	Risperidone	18	11	37					

Study	Drug	N	Mean dose	Mean age	Year	Duration	Blinding type	Diagnostic criteria	Diagnostic term
Svestka 2003a[151]	Olanzapine	20	19,5	39,5	2003	6	double-blind	ICD-10	acute schizophrenia or schizoaffective disorder
	Quetiapine	22	677,3	32,4					
Svestka 2005[152]	Olanzapine	24	-	-	2005	6	double-blind	ICD-10	acute schizophrenia or schizoaffective disorder
	Ziprasidone	24	-	-					
Tamrakar 2006[153]	Haloperidol	44	15 (10-20)	28.7	2006	6	open	ICD-10	schizophrenia
	Risperidone	33	5 (4-6)	27.3					
Taneli 2003[154]	Haloperidol	21	10 (5-20)	35.7	2003	12	open	DSM-IV	exacerbation of schizophrenia of chronic or subchronic schizophrenia
	Quetiapine	9	487 (50-750)	36.8					
Tollefson 1997[155]	Haloperidol	660	11,8	38,3	1997	6	double-blind	DSM-III-R	schizophrenia, schizophreniform disorder, schizoaffective disorder
	Olanzapine	1336	13,2	38,7					
Tuason 1984[156]	Chlorpromazine	34	570	35,5	1984	4	double-blind	Clinical diagnosis	schizophrenia, paranoid type
	Loxapine	34	59	34,4					
Tybura 2014[157]	Olanzapine	72	15 (10-20)	34.7	2014	12	open	ICD-10	schizophrenia
	Perazine	60	450 (300-600)	36					
	Ziprasidone	59	140 (120-160)	36.8					
Wagner 2005[158]	Amisulpride	26	511,1	38,3	2005	8	double-blind	DSM-IV/ICD-10	schizophrenia
	Olanzapine	26	15	34,3					
Weston 1973[159]	Haloperidol	42	5,3	49,21	1973	12	double-blind	Clinical diagnosis	schizophrenia
	Thioridazine	44	330	48,98					
Wetzel 1991[160]	Perazine	21	500	37,8	1991	4	double-blind	ICD-9	schizophrenia
	Zotepine	20	250	41,6					
Wetzel 1998[161]	Amisulpride	70	956	35	1998	6	double-blind	DSM-III-R	schizophrenia; paranoid or undifferentiated
	Flupentixol	62	22,6	33					
Yamashita 2004[162]	Olanzapine	20	16.5 (2.5-20)	56.2	2004	8	open	DSM-IV	schizophrenia
	Quetiapine	28	432.5 (50-750)	61					

Study	Drug	N	Mean dose	Mean age	Year	Dura tion	Blinding type	Diagnostic criteria	Diagnostic term
	Risperidone	20	7.4 (1-12)	62.8					
Yen 2004[163]	Haloperidol	20	11,2	34	2004	12	single-blind	DSM-III-R	schizophrenia
	Risperidone	21	4,4	32,9					
Zborowski 1995[164]	Haloperidol	115	16	39	1995	8	double-blind	DSM-III-R/DSM-IV	schizophrenia
	Placebo	116	0	38					
	Sertindole	117	20	38					
Zhang 2011[165]	Risperidone	121	3,8	34,8	2011	6	double-blind	Chinese Classification of Mental Disorders	schizophrenia
	Ziprasidone	118	118,5	34,7					
Zhong 2006[166]	Quetiapine	338	525	40,2	2006	8	double-blind	DSM-IV	schizophrenia
	Risperidone	335	5,2	39,6					
Ziegler 1989[167]	Amisulpride	20	525	32	1989	4	double-blind	ICD-9	Schizophrenia
	Haloperidol	20	12,25	39					
Zimbroff 1997[168]	Haloperidol	137	12,09	39,44	1997	8	double-blind	DSM-III-R/DSM-IV	schizophrenia
	Placebo	73	0	38,7					
	Sertindole	144	15,78	38,02					
Zimbroff 2007[169]	Aripiprazole	129	20,9	39,8	2007	4	double-blind	DSM-IV	schizophrenia or schizoaffective disorder
	Ziprasidone	127	149	40,8					
De Boer 2011[170]	Aripiprazole	23	12.6 (7.5-30)	29.2	2011	8	open	DSM-IV	schizophrenia or related psychiatric disorders
	Risperidone	21	3.2 (1-6)	29					
De Oliveira 2009[171]	Aripiprazole	66	19.4 (15-30)	34.5	2009	8	open	DSM-IV-TR	schizophrenia or schizoaffective disorder
	Haloperidol	33	10.1 (10-15)	34.2					
Van Bruggen 2003[172]	Olanzapine	18	15.6 (5-30)	21	2003	8	open	DSM-IV	first or second episode schizophrenia
	Risperidone	26	4.4 (1-8)	20.6					
	Loxapine	26	102.5 (100-150)	27.3	1975	8	double-blind		schizophrenia, acute exacerbation

Study	Drug	N	Mean dose	Mean age	Year	Duration	Blinding type	Diagnostic criteria	Diagnostic term
Van der Velde 1975[173]	Placebo	28	-	25.6				Clinical diagnosis	
	Tiotixene	28	50 (40-60)	27.6					

Study= name of study (usually first author and year of publication); Drug= interventions compared in the trial; N= Number of participants randomized; Mean dose= mean dose in milligram per day; Mean age= mean age in years, Year= year of publication; Duration= trial duration in weeks; Diagnostic criteria= diagnositic criteria used to diagnose schizophrenia and related disorders; Diagnostic term= diagnoses included in study and specific criteria defining subgroups when appropriate

References

1. A1281046 S. Study report on study A1281046. *Pfizer, data on file.* 2005.
2. A1281050 S. Study report on study A1281050. *Pfizer, data on file.* 2004.
3. Abdolahian E, Mohareri F, Bordbar MRF. Haloperidol versus risperidone. A comparison of beneficial effect on cognitive function of patients with chronic schizophrenia. *Iranian Journal of Psychiatry and Behavioral Sciences.* 2008;2(1):14–20.
4. Addington DE, Pantelis C, Dineen M, Benattia I, Romano SJ. Efficacy and tolerability of ziprasidone versus risperidone in patients with acute exacerbation of schizophrenia or schizoaffective disorder. an 8-week, double-blind, multicenter trial. *J Clin Psychiatry.* 2004;65(12):1624–1633.
5. Allan ER, Sison CE, Alpert M, Connolly B, Crichton J. The relationship between negative symptoms of schizophrenia and extrapyramidal side effects with haloperidol and olanzapine. *Psychopharmacol Bull.* 1998;34(1):71–74.
6. Arvanitis LA, Miller BG. Multiple fixed doses of "Seroquel" (quetiapine) in patients with acute exacerbation of schizophrenia. a comparison with haloperidol and placebo. The Seroquel Trial 13 Study Group. *Biol Psychiatry.* 1997;42(4):233–246.
7. Atmaca M, Kuloglu M, Unal A, Tezcan AE. The comparison of efficacy of quetiapine and haloperidol in a group of schizophrenic patients with comorbid depressive disorder. Anadolu Psikiyatri Dergisi (Anatolian Journal of Psychiatry). 2002;3(1):14–19.
8. Avasthi A, Kulhara P, Kakkar N. Olanzapine in the treatment of schizophrenia. an open label comparative clinical trial from north India. *Indian J Psychiatry.* 2001;43(3):257–263.
9. Azorin JM, Strub N, Loft H. A double-blind, controlled study of sertindole versus risperidone in the treatment of moderate-to-severe schizophrenia. *Int Clin Psychopharmacol.* 2006;21(1):49–56.
10. Beasley, C. M., Jr., Tollefson G, Tran P, Satterlee W, Sanger T, Hamilton S. Olanzapine versus placebo and haloperidol. acute phase results of the North American double-blind olanzapine trial. *Neuropsychopharmacology.* 1996;14(2):111–123.
11. Beasley, C. M., Jr., Hamilton SH, Crawford AM, et al. Olanzapine versus haloperidol. acute phase results of the international double-blind olanzapine trial. *Eur Neuropsychopharmacol.* 1997;7(2):125–137.
12. Bernardo M, Parellada E, Lomena F, et al. Double-blind olanzapine vs. haloperidol D2 dopamine receptor blockade in schizophrenic patients. a baseline-endpoint. *Psychiatry Res.* 2001;107(2):87–97.
13. Blin O, Azorin JM, Bouhours P. Antipsychotic and anxiolytic properties of risperidone, haloperidol, and methotrimeprazine in schizophrenic patients. *J Clin Psychopharmacol.* 1996;16(1):38–44.
14. Borison RL, Pathiraja AP, Diamond BI, Meibach RC. Risperidone. clinical safety and efficacy in schizophrenia. *Psychopharmacol Bull.* 1992;28(2):213–218.
15. Boulay LJ, Labelle A, Bourget D, et al. Dissociating medication effects from learning and practice effects in a neurocognitive study of schizophrenia. Olanzapine versus haloperidol. *Cogn.* 2007;12(4):322–338.
16. Brook S, Berk M, Selemani S, Kolloori J, Nzo I. A randomized controlled double blind study of zuclopenthixol acetate compared to haloperidol in acute psychosis. *Hum.* 1998;13(1):17–20. doi:10.1002/%28SICI%291099-1077%28199801%2913:1%3C17::AID-HUP937%3E3.0.CO;2-G.

17. Brook S, Walden J, Benattia I, Siu CO, Romano SJ. Ziprasidone and haloperidol in the treatment of acute exacerbation of schizophrenia and schizoaffective disorder. comparison of intramuscular and oral formulations in a 6-week, randomized, blinded-assessment study. *Psychopharmacology (Berl).* 2005;178(4):514–523.
18. Bueno JR. A double-blind comparative clinical trial with loxapine succinate and haloperidol in the treatment of schizophrenia. [Portuguese] Ensaio Clinico Comparativo E Duplo-Cego Com Succinato De Loxapina E Haloperidol No Tratamento Da Esquizofrenia. *Folha Medica.* 1979;78(1):47–52.
19. Canive JM, Miller GA, Irwin JG, et al. Efficacy of olanzapine and risperidone in schizophrenia. a randomized double-blind crossover design. *Psychopharmacol Bull.* 2006;39(1):105–116.
20. Cavallaro R, Mistretta P, Cocchi F, Manzato M, Smeraldi E. Differential efficacy of risperidone versus haloperidol in psychopathological subtypes of subchronic schizophrenia. *Hum.* 2001;16(6):439–448. doi:10.1002/hup.322.
21. Ceskova E, Svestka J. Double-blind comparison of risperidone and haloperidol in schizophrenic and schizoaffective psychoses. *Pharmacopsychiatry.* 1993;26(4):121–124.
22. Chan HY, Lin WW, Lin SK, et al. Efficacy and safety of aripiprazole in the acute treatment of schizophrenia in Chinese patients with risperidone as an active control. a randomized trial. *J Clin Psychiatry.* 2007;68(1):29–36.
23. Chouinard G, Jones B, Remington G, et al. A Canadian multicenter placebo-controlled study of fixed doses of risperidone and haloperidol in the treatment of chronic schizophrenic patients.[Erratum appears in J Clin Psychopharmacol 1993 Apr;13(2). 149]. *J Clin Psychopharmacol.* 1993;13(1):25–40.
24. Chung YC, Park KH, Kim DJ, Park KY. Prolactin Response to the Administration of Risperidone and haloperidol in Patients with Schizophrenia and Other Psychotic Disorder. Korean Journal of Psychopharmacology. 2000;11(4):343–349.
25. Chung TS, Lung FW. Different impacts of aquaporin 4 and MAOA allele variation among olanzapine, risperidone, and paliperidone in schizophrenia. *J Clin Psychopharmacol.* 2012;32(3):394–397. doi:10.1097/JCP.0b013e31825370f4.
26. Citrome L, Ota A, Nagamizu K, Perry P, Weiller E, Baker RA. The effect of brexpiprazole (OPC-34712) and aripiprazole in adult patients with acute schizophrenia. results from a randomized, exploratory study. *Int Clin Psychopharmacol.* 2016;31(4):192–201. doi:10.1097/YIC.0000000000000123.
27. Clark ML, Huber WK, Sakata K, Fowles DC, Serafetinides EA. Molindone in chronic schizophrenia. *Clin Pharmacol Ther.* 1970;11(5):680–688.
28. Clark ML, Huber WK, Sullivan J, Wood F, Costiloe JP. Evaluation of loxapine succinate in chronic schizophrenia. *Dis Nerv Syst.* 1972;33(12):783–791.
29. Clark ML, Paredes A, Costiloe JP, Wood F, Barrett A. Loxapine in newly admitted chronic schizophrenic patients. *J Clin Pharmacol.* 1975;15(4 Pt 1):286–294.
30. Claus A, Bollen J, Cuyper H de, et al. Risperidone versus haloperidol in the treatment of chronic schizophrenic inpatients. a multicentre double-blind comparative study. *Acta Psychiatr Scand.* 1992;85(4):295–305.
31. Conley RR, Mahmoud R. A randomized double-blind study of risperidone and olanzapine in the treatment of schizophrenia or schizoaffective disorder.[Erratum appears in Am J Psychiatry 2001 Oct;158(10). 1759]. *Am J Psychiatry.* 2001;158(5):765–774.

32. Cooper SJ, Tweed J, Raniwalla J, Butler A, Welch C. A placebo-controlled comparison of zotepine versus chlorpromazine in patients with acute exacerbation of schizophrenia. *Acta Psychiatr Scand.* 2000;101(3):218–225.
33. Copolov DL, Link CG, Kowalcyk B. A multicentre, double-blind, randomized comparison of quetiapine (ICI 204,636, 'Seroquel') and haloperidol in schizophrenia.[Erratum appears in Psychol Med 2000 Jul;30(4). 991]. *Psychol Med.* 2000;30(1):95–105.
34. Corripio I, Catafau AM, Perez V, et al. Striatal dopaminergic D2 receptor occupancy and clinical efficacy in psychosis exacerbation. a 123I-IBZM study with ziprasidone and haloperidol.[Erratum appears in Prog Neuropsychopharmacol Biol Psychiatry. 2006 Aug 30;30(6):1186]. *Prog Neuropsychopharmacol Biol Psychiatry.* 2005;29(1):91–96.
35. Costa-e-Silva JA. A comparative double-blind trial of amisulpride versus haloperidol in the treatment of acute psychotic disorders. [French] Etude Comparative En Double-Insu Amisulpride Versus Haloperidol Dans Le Traitement Des Etats Psychotiques Aigus. *Annales de Psychiatrie.* 1990;5(1):71–78.
36. Cutler AJ, Kalali AH, Weiden PJ, Hamilton J, Wolfgang CD. Four-week, double-blind, placebo- and ziprasidone-controlled trial of iloperidone in patients with acute exacerbations of schizophrenia. *J Clin Psychopharmacol.* 2008;28(2 Suppl 1):S20-8. doi:10.1097/JCP.0b013e318169d4ce.
37. Davidson M, Emsley R, Kramer M, et al. Efficacy, safety and early response of paliperidone extended-release tablets (paliperidone ER): results of a 6-week, randomized, placebo-controlled study.[Erratum appears in Schizophr Res. 2007 Nov;96(1-3):273-4]. *Schizophr Res.* 2007;93(1-3):117–130.
38. Dieterle DM, Miller-Spahn F, Ackenheil M. Efficacy and tolerance of zotepine in a double-blind comparison with perazin in schizophrenics. [German] Wirksamkeit Und Vertraglichkeit Von Zotepin Im Doppelblindvergleich Mit Perazin Bei Schizophrenen Patienten. *Fortschritte der Neurologie Psychiatrie.* 1991;59(SUPPL. 1):18–22.
39. Dossenbach M, Treuer T, Kryzhanovskaya L, et al. Olanzapine versus chlorpromazine in the treatment of schizophrenia. a pooled analysis of four 6-week, randomized, open-label studies in the Middle East and North Africa. *J Clin Psychopharmacol.* 2007;27(4):329–337.
40. Duggan L, Fenton M, Rathbone J, Dardennes R, El-Dosoky A, Indran S. Olanzapine for schizophrenia. *Cochrane Database Syst Rev.* 2005(2):CD001359.
41. Durgam S, Starace A, Li D, et al. An evaluation of the safety and efficacy of cariprazine in patients with acute exacerbation of schizophrenia. a phase II, randomized clinical trial. *Schizophr Res.* 2014;152(2-3):450–457. doi:10.1016/j.schres.2013.11.041.
42. Durgam S, Cutler AJ, Lu K, et al. Cariprazine in acute exacerbation of schizophrenia. a fixed-dose, phase 3, randomized, double-blind, placebo- and active-controlled trial. *J Clin Psychiatry.* 2015;76(12):e1574-82. doi:10.4088/JCP.15m09997.
43. Ehrlich S, Leopold K, Merle JV, et al. Trajectories of agouti-related protein and leptin levels during antipsychotic-associated weight gain in patients with schizophrenia. *J Clin Psychopharmacol.* 2012;32(6):767–772. doi:10.1097/JCP.0b013e318270e5c5.
44. Fakra E, Khalfa S, Da Fonseca D, et al. Effect of risperidone versus haloperidol on emotional responding in schizophrenic patients. *Psychopharmacology (Berl).* 2008;200(2):261–272. doi:10.1007/s00213-008-1203-y.

45. Fleischhacker WW, Barnas C, Stuppack CH, Unterweger B, Miller C, Hinterhuber H. Zotepine vs. haloperidol in paranoid schizophrenia. a double-blind trial. *Psychopharmacol Bull.* 1989;25(1):97–100.
46. Fleischhacker WW, McQuade RD, Marcus RN, Archibald D, Swanink R, Carson WH. A double-blind, randomized comparative study of aripiprazole and olanzapine in patients with schizophrenia. *Biol Psychiatry.* 2009;65(6):510–517. doi:10.1016/j.biopsych.2008.07.033.
47. Freeman H, Frederick AN. Comparison of trifluoperazine and molindone in chronic schizophrenic patients. *Curr Ther Res Clin Exp.* 1969;11(11):670–676.
48. Gattaz WF, Diehl A, Geuppert MS, et al. Olanzapine versus flupenthixol in the treatment of inpatients with schizophrenia. a randomized double-blind trial. *Pharmacopsychiatry.* 2004;37(6):279–285.
49. Gelenberg AJ, Doller JC. Clozapine versus chlorpromazine for the treatment of schizophrenia. preliminary results from a double-blind study. *J Clin Psychiatry.* 1979;40(5):238–240.
50. Gerlach J, Thorsen K, Fog R. Extrapyramidal reactions and amine metabolites in cerebrospinal fluid during haloperidol and clozapine treatment of schizophrenic patients. *Psychopharmacologia.* 1975;40(4):341–350.
51. Ghaleiha A, Honarbakhsh N, Boroumand MA, et al. Correlation of adenosinergic activity with superior efficacy of clozapine for treatment of chronic schizophrenia. a double blind randomised trial. *Hum.* 2011;26(2):120–124. doi:10.1002/hup.1176.
52. Goff DC, Posever T, Herz L, et al. An exploratory haloperidol-controlled dose-finding study of ziprasidone in hospitalized patients with schizophrenia or schizoaffective disorder. *J Clin Psychopharmacol.* 1998;18(4):296–304.
53. Gowardman M, Barrer B, Brown RA. Pimozide (R6238) in chronic schizophrenia. double blind trial. *N Z Med J.* 1973;78(504):487–491.
54. Guirguis E, Voineskos G, Gray J, Schlieman E. Clozapine (Leponex) vs chlorpromazine (Largactil) in acute schizophrenia: (A double-blind controlled study). *Current Therapeutic Research.* 1977;21(5):707–719.
55. Hale A, Azorin JM, Kasper S, et al. Sertindole improves both the positive and negative symptoms of schizophrenia. Results of a phase III trial. *Int.* 2000;4(1):55–62. doi:10.1080/13651500050518406.
56. Harnryd C, Bjerkenstedt L, Bjork K, et al. Clinical evaluation of sulpiride in schizophrenic patients--a double-blind comparison with chlorpromazine. *Acta Psychiatr Scand Suppl.* 1984;311:7–30.
57. Hatta K, Sato K, Hamakawa H, et al. Effectiveness of second-generation antipsychotics with acute-phase schizophrenia. *Schizophr Res.* 2009;113(1):49–55. doi:10.1016/j.schres.2009.05.030.
58. Hatta K, Takebayashi H, Sudo Y, et al. The possibility that requiring high-dose olanzapine cannot be explained by pharmacokinetics in the treatment of acute-phase schizophrenia. *Psychiatry Res.* 2013;210(2):396–401. doi:10.1016/j.psychres.2013.07.005.
59. Heikkila L, Laitinen J, Vartiainen H. Cis(Z)-clopenthixol and haloperidol in chronic schizophrenic patients--a double-blind clinical multicentre investigation. *Acta Psychiatr Scand Suppl.* 1981;294:30–38.
60. Heikkila L, Eliander H, Vartiainen H, Turunen M, Pedersen V. Zuclopenthixol and haloperidol in patients with acute psychotic states. A double-blind, multi-centre study. *Curr Med Res Opin.* 1992;12(9):594–603.

61. 041-021 SH. A multicenter, randomized, double-blind, fixed-dose, 6-week trial of the efficacy and safety of asenapine compared with placebo using olanzapine postive control in subjects with an acute exacerbation of schizophrenia. *Center for drug evaluation and research.Application number 22-117.Medical review(s).http://www.fga.gov.* 2009.
62. 041-022 SH. A multicenter, randomized, double-blind, flexible-dose, 6-week trial of the efficacy and safety of asenapine compared with placebo using olanzapine postive control in subjects with an acute exacerbation of schizophrenia. *Center for drug evaluation and research.Application number 22-117.Medical review(s).http://www.fga.gov.* 2009.
63. Hirayasu Y, Tomioka M, Iizumi M, Kikuchi H. A double-blind, placebo-controlled, comparative study of paliperidone extended release (ER) tablets in patients with schizophrenia. *Jpn.J.Clin.Psychopharmacol.* 2010;13:2077–2103.
64. Honigfeld G, Patin J, Singer J. Clozapine. Antipsychotic activity in treatment-resistant schizophrenics. *Adv Ther.* 1984;1(2):77–97.
65. Hoyberg OJ, Fensbo C, Remvig J, Lingjaerde O, Sloth-Nielsen M, Salvesen I. Risperidone versus perphenazine in the treatment of chronic schizophrenic patients with acute exacerbations. *Acta Psychiatr Scand.* 1993;88(6):395–402.
66. Huttunen MO, Piepponen T, Rantanen H, Larmo I, Nyholm R, Raitasuo V. Risperidone versus zuclopenthixol in the treatment of acute schizophrenic episodes. a double-blind parallel-group trial. *Acta Psychiatr Scand.* 1995;91(4):271–277.
67. Hwang TJ, Lin SK, Lin HN. Efficacy and safety of zotepine for the treatment of Taiwanese schizophrenic patients. a double-blind comparison with haloperidol. *J Formos Med Assoc.* 2001;100(12):811–816.
68. Hwang TJ, Lee SM, Sun HJ, et al. Amisulpride versus risperidone in the treatment of schizophrenic patients. a double-blind pilot study in Taiwan. *J Formos Med Assoc.* 2003;102(1):30–36.
69. Hwang TJ, Chan HY, Lin WW, et al. Aripiprazole versus risperidone in the treatment of acutely relapsed patients with schizophrenia in Taiwan: a randomized controlled trial. *Eur.Neuropsychopharmacol.* 2012;15:S498.
70. Ishigooka J, Inada T, Miura S. Olanzapine versus haloperidol in the treatment of patients with chronic schizophrenia. results of the Japan multicenter, double-blind olanzapine trial. *Psychiatry Clin Neurosci.* 2001;55(4):403–414.
71. Itil TM, Polvan N, Ucok A, Eper E, Guven F, Hsu W. Comparison of the clinical and electroencephalographical effects of molindone and trifluoperazine in acute schizophrenic patients. *Behav Neuropsychiatry.* 1971;3(5):25–32.
72. Itoh H, Miura S, Yagi G, Sakurai S, Ohtsuka N. Some methodological considerations for the clinical evaluation of neuroleptics--comparative effects of clozapine and haloperidol on schizophrenics. *Folia Psychiatr Neurol Jpn.* 1977;31(1):17–24.
73. Janicak PG, Keck, P. E., Jr., Davis JM, et al. A double-blind, randomized, prospective evaluation of the efficacy and safety of risperidone versus haloperidol in the treatment of schizoaffective disorder. *J Clin Psychopharmacol.* 2001;21(4):360–368.
74. Jindal KC, Singh GP, Munjal V. Aripiprazole versus olanzapine in the treatment of schizophrenia. a clinical study from India. *Int.* 2013;17(1):21–29. doi:10.3109/13651501.2011.653376.

75. Kane JM, Carson WH, Saha AR, et al. Efficacy and safety of aripiprazole and haloperidol versus placebo in patients with schizophrenia and schizoaffective disorder. *J Clin Psychiatry.* 2002;63(9):763–771.
76. Kane J, Canas F, Kramer M, et al. Treatment of schizophrenia with paliperidone extended-release tablets. a 6-week placebo-controlled trial. *Schizophr Res.* 2007;90(1-3):147–161.
77. Kane JM, Osuntokun O, Kryzhanovskaya LA, et al. A 28-week, randomized, double-blind study of olanzapine versus aripiprazole in the treatment of schizophrenia. *J Clin Psychiatry.* 2009;70(4):572–581.
78. Kane JM, Cohen M, Zhao J, Alphs L, Panagides J. Efficacy and safety of asenapine in a placebo- and haloperidol-controlled trial in patients with acute exacerbation of schizophrenia. *J Clin Psychopharmacol.* 2010;30(2):106–115. doi:10.1097/JCP.0b013e3181d35d6b.
79. Kingstone E, Kolivakis T, Kossatz I. Double blind study of clopenthixol and chlorpromazine in acute hospitalized schizophrenics. *Int Z Klin Pharmakol Ther Toxikol.* 1970;3(1):41–45.
80. Kluge M, Schuld A, Himmerich H, et al. Clozapine and olanzapine are associated with food craving and binge eating. results from a randomized double-blind study. *J Clin Psychopharmacol.* 2007;27(6):662–666.
81. Knegtering R, Castelein S, Bous H, et al. A randomized open-label study of the impact of quetiapine versus risperidone on sexual functioning. *J Clin Psychopharmacol.* 2004;24(1):56–61.
82. Knegtering H, Boks M, Blijd C, Castelein S, Van den Bosch, R. J., Wiersma D. A randomized open-label comparison of the impact of olanzapine versus risperidone on sexual functioning. *J Sex Marital Ther.* 2006;32(4):315–326.
83. Kramer M, Roth T, Salis PJ. Relative efficacy and safety of loxapine succinate (Loxitane) and thioridazine hydrochloride (Mellaril) in the treatment of acute schizophrenia. *Current Therapeutic Research - Clinical and Experimental.* 1978;23(5 II):619–631.
84. Kwon JS, Mittoux A, Hwang JY, Ong A, Cai Zj, Su TP. The efficacy and safety of 12 weeks of treatment with sertindole or olanzapine in patients with chronic schizophrenia who did not respond successfully to their previous treatments. a randomized, double-blind, parallel-group, flexible-dose study. *Int Clin Psychopharmacol.* 2012;27(6):326–335.
85. Lahti AC, Weiler MA, Holcomb HH, Tamminga CA, Cropsey KL. Modulation of limbic circuitry predicts treatment response to antipsychotic medication. a functional imaging study in schizophrenia. *Neuropsychopharmacology.* 2009;34(13):2675–2690. doi:10.1038/npp.2009.94.
86. Lamure M, Toumi M, Chabannes JP, Dansette GY, Benyaya J, Hansen K. Zuclopenthixol versus haloperidol. An observational randomised pharmacoeconomic evaluation of patients with chronic schizophrenia exhibiting acute psychosis. *International Journal of Psychiatry in Clinical Practice.* 2003;7(3):177–185. doi:10.1080/13651500310000906.
87. Landbloom R, Mackle M, Wu X, et al. Asenapine for the treatment of adults with an acute exacerbation of schizophrenia. results from a randomized, double-blind, fixed-dose, placebo-controlled trial with olanzapine as an active control. *CNS spectrums.* 2016:1–9. doi:10.1017/s1092852916000377.
88. Li Y, Li H, Liu Y, Yan X, Yue Y, Qian M. Comparison of quetiapine and risperidone in Chinese Han patients with schizophrenia. results of a single-blind, randomized study. *Curr Med Res Opin.* 2012;28(10):1725–1732. doi:10.1185/03007995.2012.728524.

89. Li H, Shen Y, Wang G, et al. A 6-week, multicenter, double-blind, double-dummy, chlorpromazine-controlled non-inferiorityrandomized phase iiitrial to evaluate the efficacy and safety of quetiapine fumarate (SEROQUEL) extended-release (XR) in the treatment of patients with schizophrenia and acute episodes. *Psychiatry Res.* 2018;259:117–124. doi:10.1016/j.psychres.2017.07.006.
90. Lieberman JA, Stroup TS, McEvoy JP, et al. Effectiveness of antipsychotic drugs in patients with chronic schizophrenia.[Erratum appears in N Engl J Med. 2010 Sep 9;363(11). 1092-3]. *N Engl J Med.* 2005;353(12):1209–1223.
91. Lin CC, Bai YM, Chen JY, et al. Switching from clozapine to zotepine in schizophrenic patients. A randomized single-blind controlled Study. *European Neuropsychopharmacology.* 2003;13(Supplement 4):S318-S319. doi:10.1016/s0924-977x(03)92042-6.
92. Liu SK, Chen WJ, Chang CJ, Lin HN. Effects of atypical neuroleptics on sustained attention deficits in schizophrenia. a trial of risperidone versus haloperidol. *Neuropsychopharmacology.* 2000;22(3):311–319.
93. Loebel A, Cucchiaro J, Sarma K, et al. Efficacy and safety of lurasidone 80 mg/day and 160 mg/day in the treatment of schizophrenia. a randomized, double-blind, placebo- and active-controlled trial. *Schizophr Res.* 2013;145(1-3):101–109. doi:10.1016/j.schres.2013.01.009.
94. Loza B, Czernikiewicz A, Roszkowska A, Szulc A. Atypical antipsychotics. The prosocial capacity. Double-blind, randomized, prospective study of olanzapine and risperidone treatment of schizophrenia: Cognitive, awareness and quality of life report. *Int J Neuropsychopharmcol.* 2006;9(Suppl. 1):S271.
95. Lublin H, Gerlach J, Hagert U, et al. Zuclopenthixol, a combined dopamine D1/D2 antagonist, versus haloperidol, a dopamine D2 antagonist, in tardive dyskinesia. *Eur Neuropsychopharmacol.* 1991;1(4):541–548.
96. McCue RE, Waheed R, Urcuyo L, et al. Comparative effectiveness of second-generation antipsychotics and haloperidol in acute schizophrenia. *Br J Psychiatry.* 2006;189:433–440.
97. Maat A, Cahn W, Gijsman HJ, Hovens JE, Kahn RS, Aleman A. Open, randomized trial of the effects of aripiprazole versus risperidone on social cognition in schizophrenia. *Eur Neuropsychopharmacol.* 2014;24(4):575–584. doi:10.1016/j.euroneuro.2013.12.009.
98. Marder SR, Meibach RC. Risperidone in the treatment of schizophrenia. *Am J Psychiatry.* 1994;151(6):825–835.
99. Marder SR, Kramer M, Ford L, et al. Efficacy and safety of paliperidone extended-release tablets. results of a 6-week, randomized, placebo-controlled study. *Biol Psychiatry.* 2007;62(12):1363–1370.
100. McQuade RD, Stock E, Marcus R, et al. A comparison of weight change during treatment with olanzapine or aripiprazole. results from a randomized, double-blind study. *J Clin Psychiatry.* 2004;65 Suppl 18:47–56.
101. Meltzer HY, Cucchiaro J, Silva R, et al. Lurasidone in the treatment of schizophrenia. a randomized, double-blind, placebo- and olanzapine-controlled study. *Am J Psychiatry.* 2011;168(9):957–967. doi:10.1176/appi.ajp.2011.10060907.
102. Mesotten F. Risperidone versus haloperidol in the treatment of chronic psychotic patients. A multicentre double-blind study. *unpublished clinical report.* 1991.
103. Meyer-Lindenberg A, Gruppe H, Bauer U, Lis S, Krieger S, Gallhofer B. Improvement of cognitive function in schizophrenic patients receiving clozapine or zotepine. results from a double-blind study. *Pharmacopsychiatry.* 1997;30(2):35–42.

104. Min SK, Rhee CS, Kim CE, Kang DY. Risperidone versus haloperidol in the treatment of chronic schizophrenic patients. a parallel group double-blind comparative trial. *Yonsei Med J.* 1993;34(2):179–190.
105. Mirabzadeh A, Kimiaghalam P, Fadai F, Samiei M, Daneshmand R. The therapeutic effectiveness of risperidone on negative symptoms of schizophrenia in comparison with haloperidol. a randomized clinical trial. *Iran.* 2014;5(3):212–217.
106. Moeller HJ, Boyer P, Rein W, Eich FX, Group AS. Treatment of schizophrenic patients with acute exacerbations. A double-blind comparison of amisulpride and haloperidol. *Pharmacopsychiatry.* 1997;30(5):199.
107. Moosavi SM, Ahmadi M, Mojtahedi D, Yazdani J, M BM. Comparison of Quetiapine and Risperidone in Treatment of Acute Psychosis. A Double-Blind, Randomized-Controlled Study. *Glob J Health Sci.* 2015;7(5):41952. doi:10.5539/gjhs.v7n5p359.
108. Mori K, Nagao M, Yamashita H, Morinobu S, Yamawaki S. Effect of switching to atypical antipsychotics on memory in patients with chronic schizophrenia. *Prog Neuropsychopharmacol Biol Psychiatry.* 2004;28(4):659–665.
109. Mortimer A, Martin S, Loo H, Peuskens J, Group SS. A double-blind, randomized comparative trial of amisulpride versus olanzapine for 6 months in the treatment of schizophrenia. *Int Clin Psychopharmacol.* 2004;19(2):63–69.
110. Moyano CZ. A double blind comparison of Loxitane(TM) loxapine succinate and trifluoperazine hydrochloride in chronic schizophrenic patients. *Disease of the Nervous System.* 1975;36(6):301–304.
111. Mitsukuni M, Sadanori M, Itaru Y, et al. Efficacy of a New Antipsychotic, Risperidone, on Schizophrenia. A comparative double-blind study with haloperidol. Rinsho Hyoka (Clinical Evaluation). 1993;21(2):221–259.
112. Murasaki M, Koyama T, Yagi G, Ushijima S, Kamijima K. Efficacy and tolerability of quetiapine compared with haloperidol in patients with schizophrenia. International Journal of Neuropsychopharmacology (Abstracts of the XXIInd CINP Congress, Brussels, Belgium, July 9-13, 2000). 2000;3(Suppl 1):S150.
113. NCT00350467. A Randomized, Active-controlled, Double-blind, Parallel-Goup Study of the Efficacy and Safety of Extended Release(ER) Paliperidone in the Treatment of Schizophrenia. http://clinicaltrials.gov/show/NCT00350467</web_address>. 2006.
114. NCT00905307. Study to Evaluate the Efficacy, Safety, and Tolerability of Oral OPC-34712 and Aripiprazole for Treatment of Acute Schizophrenia. http://clinicaltrials.gov/show/NCT00905307</web_address>. 2009.
115. NCT01810380. Brexpiprazole in Patients With Acute Schizophrenia. *https://ClinicalTrials.gov/show/NCT01810380.* 2013.
116. Nam CW, Yang BH, Lee JN. The Influences of Risperidone and Clozapine on Body Weight and Glucose Level in Patients with Chronic Schizophrenia. Korean Journal of Biological Psychiatry. 2004;11(2):127–135.
117. Nishizono M. A comparative trial zotepine, chlorpromazine and haloperidol in schizophrenic patients. *Neuropsychopharmacology.* 1994;10 (suppl.):30S.
118. Nistico G, Ragozzine D, Marano V. A comparative study of penfluridol and flupentixol in the treatment of chronic schizophrenia. *J Clin Pharmacol.* 1974;14(8):476–482.

119. Ozguven HD, Baskak B, Oner O, Atbasoglu C. Metabolic effects of olanzapine and quetiapine. A six-week randomized, single blind, controlled study. *Open Neuropsychopharmacology Journal.* 2011;4(1):10–17. doi:10.2174/1876523801104010010.
120. Petit M, Raniwalla J, Tweed J, Leutenegger E, Dollfus S, Kelly F. A comparison of an atypical and typical antipsychotic, zotepine versus haloperidol in patients with acute exacerbation of schizophrenia. a parallel-group double-blind trial. *Psychopharmacol Bull.* 1996;32(1):81–87.
121. Peuskens J. Risperidone in the treatment of patients with chronic schizophrenia. a multi-national, multi-centre, double-blind, parallel-group study versus haloperidol. Risperidone Study Group. *Br J Psychiatry.* 1995;166(6):712-26; discussion 727-33.
122. Peuskens J, Link CG. A comparison of quetiapine and chlorpromazine in the treatment of schizophrenia. *Acta Psychiatr Scand.* 1997;96(4):265–273.
123. Peuskens J, Bech P, Moller HJ, Bale R, Fleurot O, Rein W. Amisulpride vs. risperidone in the treatment of acute exacerbations of schizophrenia. Amisulpride study group. *Psychiatry Res.* 1999;88(2):107–117.
124. Potkin SG, Saha AR, Kujawa MJ, et al. Aripiprazole, an antipsychotic with a novel mechanism of action, and risperidone vs placebo in patients with schizophrenia and schizoaffective disorder. *Arch Gen Psychiatry.* 2003;60(7):681–690.
125. Potkin SG, Cohen M, Panagides J. Efficacy and tolerability of asenapine in acute schizophrenia. a placebo- and risperidone-controlled trial. *J Clin Psychiatry.* 2007;68(10):1492–1500.
126. Potkin SG, Litman RE, Torres R, Wolfgang CD. Efficacy of iloperidone in the treatment of schizophrenia. initial phase 3 studies. *J Clin Psychopharmacol.* 2008;28(2 Suppl 1):S4-11. doi:10.1097/JCP.0b013e3181692787.
127. Potkin SG, Kimura T, Guarino J. A 6-week, double-blind, placebo- and haloperidol-controlled, phase II study of lurasidone in patients with acute schizophrenia.[Erratum appears in Ther Adv Psychopharmacol. 2015 Dec;5(6). 369; PMID: 26836398]. *Ther.* 2015;5(6):322–331. doi:10.1177/2045125315606027.
128. ZIP-NY-97-019 S. Study report on study ZIP-NY-97-019. *Pfizer, data on file.* 2004.
129. Puech A, Fleurot O, Rein W. Amisulpride, and atypical antipsychotic, in the treatment of acute episodes of schizophrenia. a dose-ranging study vs. haloperidol. The Amisulpride Study Group. *Acta Psychiatr Scand.* 1998;98(1):65–72.
130. Rickels K, Byrdy H, Valentine J, Postel W, Norstad N, Downing R. Double-blind trial of thiothixene and chlorpromazine in acute schizophrenia. *Int Pharmacopsychiatry.* 1978;13(1):50–57.
131. Riedel M, Muller N, Spellmann I, et al. Efficacy of olanzapine versus quetiapine on cognitive dysfunctions in patients with an acute episode of schizophrenia. *Eur Arch Psychiatry Clin Neurosci.* 2007;257(7):402–412.
132. Rifkin A, Rieder E, Sarantakos S, Saraf K, Kane J. Is loxapine more effective than chlorpromazine in paranoid schizophrenia? *Am J Psychiatry.* 1984;141(11):1411–1413.
133. Rosenheck R, Perlick D, Bingham S, et al. Effectiveness and cost of olanzapine and haloperidol in the treatment of schizophrenia. a randomized controlled trial. *Jama.* 2003;290(20):2693–2702.
134. Rüther E, J. B. Therapievergleich von Aminosultoprid (DAN 2163) und Perazin bei schizophrenen Patienten. In: H. H, Hippius H, R. T, eds. *Therapie mit Neuroleptika - Perazin*. Stuttgart New York: Georg Thieme Verlag; 1988:65–70.

135. Curtis VA, Katsafouros K, Moller HJ, Medori R, Sacchetti E. Long-acting risperidone improves negative symptoms in stable psychotic patients. *J Psychopharmacol.* 2008;22(3):254–261. doi:10.1177/0269881107082119.
136. Sacchetti E, Valsecchi P, Parrinello G, Group Q. A randomized, flexible-dose, quasi-naturalistic comparison of quetiapine, risperidone, and olanzapine in the short-term treatment of schizophrenia. the QUERISOLA trial. *Schizophr Res.* 2008;98(1-3):55–65.
137. Safa M, Sadr S, Delfan B, Saki M, Javad Tarrahi M. Metabolic effects of olanzapine and risperidone in patients with psychotic disorders. *Int.* 2008;12(4):299–302. doi:10.1080/13651500802155337.
138. Schennach R, Riedel M, Spellmann I, et al. Comparing Schizophrenia Patients With a Predicted High/Low Risk of Nonresponse Receiving Treatment with Ziprasidone and Haloperidol: A Randomized-Controlled Study. *Pharmacopsychiatry.* 2019;52(4):180–185. doi:10.1055/a-0669-9461.
139. See RE, Fido AA, Maurice M, Ibrahim MM, Salama GM. Risperidone-induced increase of plasma norepinephrine is not correlated with symptom improvement in chronic schizophrenia. *Biol Psychiatry.* 1999;45(12):1653–1656.
140. Selman FB, McClure RF, Helwig H. Loxapine succinate. a double-blind comparison with haloperidol and placebo in acute schizophrenics. *Curr Ther Res Clin Exp.* 1976;19(6):645–652.
141. Sergi MJ, Green MF, Widmark C, et al. Social cognition [corrected] and neurocognition. effects of risperidone, olanzapine, and haloperidol.[Erratum appears in Am J Psychiatry. 2007 Nov;164(11):1766]. *Am J Psychiatry.* 2007;164(10):1585–1592.
142. Shah S, Joshi D. Tolerability and efficacy of paliperidone ER compared to olanzapine in the treatment of schizophrenia. A randomized, double-blind, multicentric trial. *Ind.* 2011;20(1):25–31. doi:10.4103/0972-6748.98411.
143. Simpson GM, Glick ID, Weiden PJ, Romano SJ, Siu CO. Randomized, controlled, double-blind multicenter comparison of the efficacy and tolerability of ziprasidone and olanzapine in acutely ill inpatients with schizophrenia or schizoaffective disorder. *Am J Psychiatry.* 2004;161(10):1837–1847.
144. Sonmez B, Vardar E, Altun GD, Abay E, Bedel D. Ziprasidone versus risperidone. Comparison of clinical efficacy and cardiac, extrapyramidal, and metabolic side effects in patients with acute exacerbation of schizophrenia and schizoaffective disorders. *Klinik Psikofarmakoloji Bulteni / Bulletin of Clinical Psychopharmacology.* 2009;19(2):101–112.
145. 2000 S1. Center for drug evaluation and research approval package for application number 20-825. Medical review. *http://www.fda.gov.* 2000.
146. 128-301 S. Study report of study 128-301. *Pfizer, data on file.* 1997.
147. Feeney J, Wolfgang C, Polymeropoulos M, Baroldi P, Hamilton J. The comparative efficacy of iloperidone and haloperidol across four short-term controlled trials: (Poster nr1-026). *Presented at the 162nd Annual Meeting of the American Psychiatric Association, San Francisco, 2009.* 2009:Poster nr1-026.
148. 2002 S9. Center for drug evaluation and research. Application number 21-436. Medical review(s). *http://www.fda.gov.* 2002.
149. 2002 S9. Center for drug evaluation and research. Application number 21-436. Medical review(s). *http://www.fda.gov.* 2002.
150. Svestka J, Ceskova E, Rysanek R, Obrovska V. Double-blind clinical comparison of riperidon and haloperidol in acute schizophrenic and schizoaffective psychoses. *Act Nerv Super (Praha).* 1990;32(3):237–238.

151. Svestka J, Synek O, Zourkova A. A double-blind comparison of olanzapine and quetiapine in treatment of acute exacerbations of schizophrenic or schizoaffective disorders. *Eur Neuropsychopharmacol.* 2003;13(Supplement 4):S292. doi:10.1016/s0924-977x(03)91984-5.
152. Svestka J, Synek O, Zourkova A. A double-blind comparison of olanzapine and ziprasidone in treatment of acute exacerbations of schizophrenic or schizoaffective disorders. *Psychiatr.Danub.* 2005.
153. Tamrakar SM, Nepal MK, Koirala NR, Sharma VD, Gurung CK, Adhikari SR. An open, randomized, comparative study of efficacy and safety of risperidone and haloperidol in schizophrenia. *Kathmandu Univ.* 2006;4(2):152–160.
154. Taneli B, Alptekin K, Bilici M, et al. Comparison of efficacy and tolerability of quetiapine and haloperidol in acute exacerbation of chronic or subchronic schizophrenia. *Eur.Neuropsychopharmacol.* 2003;13:S287-S287.
155. Tollefson GD, Beasley, C. M., Jr., Tran PV, et al. Olanzapine versus haloperidol in the treatment of schizophrenia and schizoaffective and schizophreniform disorders. results of an international collaborative trial. *Am J Psychiatry.* 1997;154(4):457–465.
156. Tuason VB, Escobar JI, Garvey M, Schiele B. LOXAPINE VS. CHLORPROMAZINE IN PARANOID SCHIZOPHRENIA A DOUBLE-BLIND STUDY. *J Clin Psychiatry.* 1984;45(4):158–163.
157. Tybura P, Trzesniowska-Drukala B, Bienkowski P, et al. Pharmacogenetics of adverse events in schizophrenia treatment. comparison study of ziprasidone, olanzapine and perazine. *Psychiatry Res.* 2014;219(2):261–267. doi:10.1016/j.psychres.2014.05.039.
158. Wagner M, Quednow BB, Westheide J, Schlaepfer TE, Maier W, Kuhn KU. Cognitive improvement in schizophrenic patients does not require a serotonergic mechanism. randomized controlled trial of olanzapine vs amisulpride. *Neuropsychopharmacology.* 2005;30(2):381–390.
159. Weston MJ, Bentley R, Unwin A, Morris M, Harper MA. A comparative trial of haloperidol and thioridazine. management of chronic schizophrenia. *Aust N Z J Psychiatry.* 1973;7(1):52–57.
160. Wetzel H, Bardeleben U von, Holsboer F, Benkert O. [Zotepine versus perazine in patients with paranoid schizophrenia. a double-blind controlled trial of its effectiveness]. *Fortschr Neurol Psychiatr.* 1991;59 Suppl 1:23–29.
161. Wetzel H, Grunder G, Hillert A, et al. Amisulpride versus flupentixol in schizophrenia with predominantly positive symptomatology -- a double-blind controlled study comparing a selective D2-like antagonist to a mixed D1-/D2-like antagonist. The Amisulpride Study Group. *Psychopharmacology (Berl).* 1998;137(3):223–232.
162. Yamashita H, Mori K, Nagao M, Okamoto Y, Morinobu S, Yamawaki S. Effects of changing from typical to atypical antipsychotic drugs on subjective sleep quality in patients with schizophrenia in a Japanese population. *J Clin Psychiatry.* 2004;65(11):1525–1530.
163. Yen YC, Lung FW, Chong MY. Adverse effects of risperidone and haloperidol treatment in schizophrenia. *Prog Neuropsychopharmacol Biol Psychiatry.* 2004;28(2):285–290.
164. Zborowski J, Schmitz P, Staser J, et al. Efficacy and safety of sertindole in a trial of schizophrenic patients. *Biol.Psychiatry.* 1995;37:661–662.
165. Zhang H, Li H, Shu L, et al. Double-blind comparison of ziprasidone and risperidone in the treatment of Chinese patients with acute exacerbation of schizophrenia. *Neuropsychiatr.* 2011;7:77–85. doi:10.2147/NDT.S16664.

166. Zhong KX, Sweitzer DE, Hamer RM, Lieberman JA. Comparison of quetiapine and risperidone in the treatment of schizophrenia. A randomized, double-blind, flexible-dose, 8-week study. *J Clin Psychiatry.* 2006;67(7):1093–1103.
167. Ziegler B. Study of the efficacy of a substituted benzamide amisulpride, versus haloperidol, in productive schizophrenia. *Amisulpride*. Paris: Expansion scientifique francaise; 1989:73–81.
168. Zimbroff DL, Kane JM, Tamminga CA, et al. Controlled, dose-response study of sertindole and haloperidol in the treatment of schizophrenia. Sertindole Study Group. *Am J Psychiatry.* 1997;154(6):782–791.
169. Zimbroff D, Warrington L, Loebel A, Yang R, Siu C. Comparison of ziprasidone and aripiprazole in acutely ill patients with schizophrenia or schizoaffective disorder. a randomized, double-blind, 4-week study. *Int Clin Psychopharmacol.* 2007;22(6):363–370.
170. Boer MK de, Wiersma D, Bous J, et al. A randomized open-label comparison of the impact of aripiprazole versus risperidone on sexual functioning (RAS study). *J Clin Psychopharmacol.* 2011;31(4):523–525. doi:10.1097/JCP.0b013e318222bb29.
171. Oliveira IR de, Elkis H, Gattaz WF, et al. Aripiprazole for patients with schizophrenia and schizoaffective disorder. an open-label, randomized, study versus haloperidol. *CNS Spectr.* 2009;14(2):93–102.
172. van Bruggen J, Tijssen J, Dingemans P, Gersons B, Linszen D. Symptom response and side-effects of olanzapine and risperidone in young adults with recent onset schizophrenia. *Int Clin Psychopharmacol.* 2003;18(6):341–346.
173. van der Velde, C. D., Kiltie H. Effectiveness of loxapine succinate in acute schizophrenia. a comparative study with thiothixene. *Curr Ther Res Clin Exp.* 1975;17(1):1–12.

eTable 2

a) Risk of Bias Table
b) Risk of Bias Summary

eTable 2a: Risk of bias table

Study	Random sequence generation	Allocation concealment	Blinding of participants and personal	Blinding of outcome assessment	Incomplete outcome data	Selective reporting	Other sources of bias		Overall risk of bias
A1281046	Low risk	Unclear risk	Low risk	Unclear risk	Unclear risk	Unclear risk	Unclear risk		Moderate
A1281050	Unclear risk	Unclear risk	Unclear risk	Unclear risk	Unclear risk	Unclear risk	Unclear risk		Moderate
Abdolahian 2008	Unclear risk	Unclear risk	Unclear risk	Unclear risk	Unclear risk	Low risk	Low risk		Moderate
Addington 2004	Low risk	Low risk	Unclear risk	Unclear risk	Low risk	Low risk	Low risk		Low
Allan 1998	Unclear risk	Unclear risk	Unclear risk	Unclear risk	Unclear risk	Unclear risk	Low risk		Moderate
Arvanitis 1997	Unclear risk	Unclear risk	Unclear risk	Unclear risk	Low risk	Low risk	Low risk		Moderate
Atmaca 2002	Unclear risk	Unclear risk	Unclear risk	Unclear risk	Low risk	High risk	Low risk		Moderate
Avasthi 2001	Unclear risk	Unclear risk	High risk	High risk	Low risk	Low risk	Low risk		High
Azorin 2006	Unclear risk	Unclear risk	Unclear risk	Unclear risk	Low risk	Low risk	High risk		Moderate
Beasley 1996b	Low risk	Low risk	Unclear risk	Unclear risk	Low risk	Low risk	Low risk		Low
Beasley 1997	Low risk	Low risk	Low risk	Low risk	Low risk	Low risk	Low risk		Low
Bernardo 2001	Unclear risk	Unclear risk	Unclear risk	Unclear risk	Unclear risk	Unclear risk	Low risk		Moderate
Blin 1996	Unclear risk	Unclear risk	Low risk	Low risk	Low risk	Low risk	Low risk		Low
Borison 1992	Low risk	Unclear risk	Low risk	Low risk	Low risk	Low risk	High risk		Moderate
Boulay 2007	Unclear risk	Unclear risk	Unclear risk	Unclear risk	Low risk	High risk	Low risk		Moderate
Brook 1998	Unclear risk	Unclear risk	Unclear risk	Low risk	High risk	Low risk	Low risk		Moderate
Brook 2005	Low risk	Low risk	High risk	Unclear risk	Low risk	Low risk	Low risk		Moderate
Bueno 1979	Unclear risk	Unclear risk	Low risk	Low risk	Unclear risk	High risk	Low risk		Moderate
Canive 2006	Low risk	Unclear risk	Unclear risk	Unclear risk	High risk	Low risk	Low risk		Moderate
Cavallaro 2001	Unclear risk	Low risk	Low risk	Low risk	High risk	High risk	Low risk		High
Ceskova 1993	Unclear risk	Unclear risk	Unclear risk	Unclear risk	Unclear risk	Low risk	Low risk		Moderate
Chan 2007	Low risk	Low risk	Low risk	Low risk	Low risk	Low risk	Low risk		Low
Chen 2018	Unclear risk	Unclear risk	High risk	High risk	High risk	Low risk	Low risk		High
Chiu 1976	Unclear risk	Unclear risk	Low risk	Low risk	High risk	High risk	High risk		High
Chouinard 1993	Unclear risk	Unclear risk	Low risk	Low risk	Low risk	Low risk	Low risk		Low
Chung 2000	Unclear risk	Unclear risk	Unclear risk	Unclear risk	Unclear risk	Unclear risk	Unclear risk		Moderate

Chung 2012	Unclear risk	Unclear risk	High risk	High risk	High risk	High risk	Low risk		High
Citrome 2015	Unclear risk	Low risk	High risk	High risk	Low risk	Low risk	Low risk		High
Clark 1970a	Unclear risk	Unclear risk	Low risk	Low risk	Low risk	Low risk	Low risk		Low
Clark 1972	Unclear risk	Unclear risk	Low risk	Low risk	High risk	Low risk	Low risk		Moderate
Clark 1975	Low risk	Unclear risk	Low risk	Low risk	Low risk	Low risk	Low risk		Low
Claus 1992	Unclear risk	Unclear risk	High risk	High risk	Low risk	Low risk	Low risk		High
Conley 2001	Unclear risk	Unclear risk	Unclear risk	Unclear risk	Low risk	Low risk	Low risk		Moderate
Cooper 2000a	Unclear risk	Unclear risk	Low risk	Low risk	Low risk	Low risk	Low risk		Low
Copolov 2000	Unclear risk	Unclear risk	Unclear risk	Unclear risk	Low risk	Low risk	Low risk		Moderate
Corripio 2005	Unclear risk	Unclear risk	High risk	Unclear risk	Low risk	High risk	Low risk		High
Costa e Silva 1989	Low risk	Low risk	Low risk	Low risk	High risk	Unclear risk	High risk		High
Cutler 2008	Low risk	Low risk	Low risk	Low risk	High risk	Low risk	Low risk		Moderate
Davidson 2007	Low risk	Low risk	Low risk	Low risk	Low risk	Low risk	Low risk		Low
Dieterle 1991	Unclear risk	Unclear risk	Low risk	Low risk	Unclear risk	Low risk	Low risk		Low
Dossenbach 2007	Unclear risk	Unclear risk	High risk	High risk	Low risk	High risk	Low risk		High
Duggan 2005	Low risk	Unclear risk	High risk	High risk	Unclear risk	Unclear risk	Low risk		High
Durgam 2014	Unclear risk	Unclear risk	Unclear risk	Unclear risk	Low risk	Low risk	Low risk		Moderate
Durgam 2015	Unclear risk	Unclear risk	Unclear risk	Unclear risk	Low risk	Low risk	Low risk		Moderate
Ehrlich 2012	Unclear risk	Unclear risk	High risk	High risk	High risk	High risk	Low risk		High
Fakra 2008	Unclear risk	Unclear risk	High risk	High risk	High risk	Low risk	Low risk		High
Fleischhacker 1989	Low risk	Low risk	Low risk	Low risk	High risk	High risk	Low risk		High
Fleischhacker 2009_6weeks	Low risk	Low risk	Low risk	Low risk	Low risk	Low risk	Low risk		Low
Freeman 1969	Unclear risk	Unclear risk	Low risk	Low risk	High risk	Low risk	Low risk		Moderate
Gattaz 2004	Unclear risk	Unclear risk	Unclear risk	Unclear risk	Low risk	Low risk	Low risk		Moderate
Gelenberg 1979	Unclear risk	Unclear risk	Unclear risk	Unclear risk	High risk	Low risk	High risk		High
Gerlach 1975	Unclear risk	Unclear risk	Low risk	Low risk	Unclear risk	Low risk	Low risk		Low
Ghaleiha 2011	Low risk	Unclear risk	Unclear risk	Unclear risk	High risk	High risk	Low risk		High
Goff 1998	Unclear risk	Unclear risk	Unclear risk	Unclear risk	Low risk	Low risk	Low risk		Moderate
Gowardman 1973	Unclear risk	Low risk	Low risk	Low risk	Low risk	Low risk	Low risk		Low
Grootens 2009	Unclear risk	Unclear risk	Low risk	Low risk	Unclear risk	Low risk	Low risk		Low

Guirguis 1977	Unclear risk	Low risk	Low risk	Low risk	High risk	High risk	High risk		High
Hale 2000	Low risk	Low risk	Low risk	Low risk	Low risk	Low risk	Low risk		Low
Harnryd 1984	Unclear risk	Unclear risk	Low risk	Low risk	Unclear risk	Low risk	Low risk		Low
Hatta 2009	Low risk	Low risk	Low risk	High risk	Low risk	Low risk	High risk		High
Hatta 2013	Low risk	Low risk	High risk	Unclear risk	Low risk	Low risk	Low risk		Moderate
Heikkila 1981	Unclear risk	Unclear risk	Unclear risk	Unclear risk	High risk	Low risk	Low risk		Moderate
Heikkilae 1992	Unclear risk	Unclear risk	Low risk	Low risk	Low risk	Low risk	Low risk		Low
Hera 041-021	Unclear risk	Unclear risk	Low risk	Unclear risk	Unclear risk	Unclear risk	Unclear risk		Moderate
Hera 041-022	Unclear risk	Unclear risk	Low risk	Unclear risk	Low risk	Unclear risk	Unclear risk		Moderate
Hirayasu 2010	Unclear risk	Unclear risk	Low risk	Low risk	Unclear risk	Unclear risk	Low risk		Moderate
Honigfeld 1984a	Unclear risk	Unclear risk	Low risk	Low risk	Unclear risk	Unclear risk	Low risk		Moderate
Honigfeld 1984c	Unclear risk	Unclear risk	Unclear risk	Unclear risk	Unclear risk	Unclear risk	Low risk		Moderate
Honigfeld 1984d	Unclear risk	Unclear risk	Low risk	Low risk	Unclear risk	High risk	Low risk		Moderate
Hoyberg 1993	Unclear risk	Unclear risk	Low risk	Low risk	Low risk	Low risk	Low risk		Low
Huttunen 1995	Unclear risk	Unclear risk	Unclear risk	Unclear risk	Low risk	High risk	Low risk		Moderate
Hwang 2001	Unclear risk	Unclear risk	Low risk	Low risk	Low risk	Low risk	Low risk		Low
Hwang 2003	Unclear risk	Unclear risk	Low risk	Low risk	Low risk	Low risk	Low risk		Low
Hwang 2012	Unclear risk	Unclear risk	Unclear risk	Unclear risk	Unclear risk	Unclear risk	Unclear risk		Moderate
Ingole 2009	Unclear risk	Unclear risk	High risk	High risk	Low risk	Low risk	Low risk		High
Ishigooka 2001	Unclear risk	Unclear risk	Unclear risk	Unclear risk	Low risk	Low risk	Low risk		Moderate
Itil 1971	Unclear risk	Unclear risk	Unclear risk	Unclear risk	Low risk	Low risk	Low risk		Moderate
Itoh 1977	Unclear risk	Unclear risk	Unclear risk	Unclear risk	Low risk	High risk	Low risk		Moderate
Janicak 2001	Unclear risk	Unclear risk	Low risk	Low risk	Low risk	Low risk	Low risk		Low
Jindal 2013	Low risk	Unclear risk	Low risk	Low risk	High risk	Low risk	Low risk		Moderate
Kane 2002	Unclear risk	Unclear risk	Unclear risk	Unclear risk	Low risk	Low risk	Low risk		Moderate
Kane 2007b	Low risk	Low risk	Low risk	Low risk	Low risk	Low risk	Low risk		Low
Kane 2009_8 weeks	Unclear risk	Unclear risk	Unclear risk	Unclear risk	Low risk	Low risk	Low risk		Moderate
Kane 2010a	Unclear risk	Unclear risk	Low risk	Low risk	Unclear risk	Low risk	Low risk		Low
Kingstone 1970	Unclear risk	Unclear risk	Low risk	Low risk	Unclear risk	Low risk	Low risk		Low
Kluge 2007	Low risk	Low risk	Low risk	Low risk	Low risk	Low risk	Low risk		Low

Knegtering 2004	Unclear risk	Unclear risk	High risk	High risk	High risk	High risk	Unclear risk		High
Knegtering 2006	Unclear risk	Unclear risk	High risk	High risk	Low risk	High risk	Low risk		High
Kramer 1978	Unclear risk	Unclear risk	Low risk	Low risk	High risk	Low risk	Low risk		Moderate
Kwon 2012	Unclear risk	Unclear risk	Low risk	Low risk	Low risk	Low risk	Low risk		Low
Lahti 2009	Unclear risk	Unclear risk	Low risk	Low risk	High risk	Low risk	Low risk		Moderate
Lamure 2003	Unclear risk	Unclear risk	High risk	High risk	High risk	Low risk	Low risk		High
Landbloom 2016	Low risk	Unclear risk	Low risk	Low risk	Low risk	Low risk	Low risk		Low
Li 2012	Low risk	Unclear risk	High risk	Low risk	Low risk	Low risk	Low risk		Moderate
Li 2017	Unclear risk	Unclear risk	Unclear risk	Unclear risk	Low risk	Low risk	High risk		Moderate
Lieberman 2005_12weeks	Unclear risk	Unclear risk	Low risk	Low risk	Low risk	Low risk	Low risk		Low
Lin 2003	Unclear risk	Unclear risk	High risk	Unclear risk	Low risk	Low risk	High risk		High
Liu 2000	Low risk	Unclear risk	Unclear risk	Unclear risk	High risk	High risk	Low risk		High
Loebel 2013	Low risk	Low risk	Low risk	Low risk	Low risk	Low risk	Low risk		Low
Loza 1999	Low risk	Unclear risk	High risk	High risk	Low risk	Low risk	Low risk		High
Lublin 1991	Unclear risk	Unclear risk	Low risk	Unclear risk	Unclear risk	Unclear risk	Low risk		Moderate
Maat 2014	Unclear risk	Unclear risk	High risk	High risk	High risk	High risk	Low risk		High
Marder 1994	Low risk	Unclear risk	Low risk	Low risk	Low risk	Low risk	Low risk		Low
Marder 2007c	Low risk	Low risk	Low risk	Low risk	Low risk	Low risk	Low risk		Low
McCue2006	Low risk	Unclear risk	High risk	High risk	Low risk	Low risk	Low risk		High
McQuade 2004_6weeks	Unclear risk	Unclear risk	Unclear risk	Unclear risk	High risk	Low risk	High risk		High
Meltzer 2011	Low risk	Low risk	Low risk	Low risk	Low risk	Low risk	Low risk		Low
Mesotten 1991	Unclear risk	Unclear risk	Low risk	Low risk	Unclear risk	Unclear risk	Unclear risk		Moderate
Meyer-Lindenberg 1997	Low risk	Low risk	Low risk	Low risk	Low risk	Low risk	Low risk		Low
Min 1993	Low risk	Low risk	Low risk	Low risk	Low risk	High risk	Low risk		Moderate
Mirabzadeh 2014	Unclear risk	Unclear risk	Unclear risk	Unclear risk	Unclear risk	Low risk	High risk		Moderate
Moosavi 2015	Low risk	Unclear risk	Unclear risk	Unclear risk	High risk	Low risk	Low risk		Moderate
Mori 2004	Unclear risk	Unclear risk	Unclear risk	Unclear risk	High risk	Low risk	Low risk		Moderate
Mortimer 2004_6 weeks	Low risk	Low risk	Low risk	Low risk	Low risk	Low risk	Low risk		Low
Moyano 1975	Unclear risk	Unclear risk	Low risk	Low risk	High risk	Low risk	Low risk		Moderate
Murasaki 1993	Unclear risk	Unclear risk	Unclear risk	Unclear risk	Unclear risk	Low risk	Low risk		Moderate

Murasaki 2001	Unclear risk	Unclear risk	Unclear risk	Unclear risk	Unclear risk	Low risk	Low risk		Moderate
Möller 1997	Low risk	Low risk	Low risk	Low risk	Low risk	Low risk	Low risk		Low
NCT00350467	Unclear risk	Unclear risk	Low risk	Low risk	Unclear risk	Low risk	Low risk		Low
NCT00905307	Low risk	Low risk	Low risk	Low risk	Low risk	Low risk	Low risk		Low
NCT01810380	Unclear risk	Unclear risk	Unclear risk	Unclear risk	Low risk	Low risk	Low risk		Moderate
Nam 2004	Unclear risk	Unclear risk	High risk	Unclear risk	Unclear risk	Unclear risk	Unclear risk		Moderate
Nishizono 1994	Unclear risk	Low risk	Unclear risk	Unclear risk	Unclear risk	Unclear risk	Unclear risk		Moderate
Ozguven 2004	Low risk	Low risk	High risk	Unclear risk	Unclear risk	High risk	Unclear risk		High
Petit 1996	Unclear risk	Unclear risk	Low risk	Low risk	Low risk	Low risk	Low risk		Low
Peuskens 1995	Low risk	Low risk	Low risk	Low risk	Low risk	Low risk	Low risk		Low
Peuskens 1997	Low risk	Low risk	High risk	High risk	Low risk	Low risk	Low risk		High
Peuskens 1999	Low risk	Low risk	Low risk	Low risk	Low risk	Low risk	Low risk		Low
Potkin 2003	Unclear risk	Unclear risk	Unclear risk	Unclear risk	Low risk	High risk	Low risk		Moderate
Potkin 2007c	Unclear risk	Unclear risk	Low risk	Low risk	Low risk	Low risk	Low risk		Low
Potkin 2008a	Low risk	Low risk	Low risk	Low risk	Low risk	Low risk	Low risk		Low
Potkin 2008b	Low risk	Low risk	Low risk	Low risk	Low risk	Low risk	Low risk		Low
Potkin 2008c	Low risk	Low risk	Low risk	Low risk	Low risk	Low risk	Low risk		Low
Potkin 2015	Low risk	Low risk	Low risk	Low risk	Low risk	Low risk	Low risk		Low
Protocol ZIP-NY-97-019	Unclear risk	Unclear risk	Unclear risk	Unclear risk	Unclear risk	Unclear risk	Unclear risk		Moderate
Puech 1998	Low risk	Low risk	Low risk	Low risk	Low risk	Low risk	Low risk		Low
Rickels 1978	Unclear risk	Unclear risk	Low risk	Low risk	Unclear risk	High risk	Low risk		Moderate
Riedel 2007	Unclear risk	Unclear risk	Unclear risk	Unclear risk	High risk	Low risk	Low risk		Moderate
Rifkin 1984	Unclear risk	Unclear risk	Low risk	Low risk	High risk	Unclear risk	Low risk		Moderate
Rosenheck 2003_6 weeks	Low risk	Low risk	Low risk	Low risk	Unclear risk	Low risk	High risk		Moderate
Rüther 1988	Low risk	Low risk	Low risk	Low risk	Unclear risk	Low risk	Low risk		Low
Sacchetti 2008	Low risk	Unclear risk	High risk	Unclear risk	High risk	Unclear risk	Low risk		High
Safa 2008	Unclear risk	Unclear risk	Unclear risk	Unclear risk	High risk	Low risk	Low risk		Moderate
Sarai 1987	Unclear risk	Low risk	Unclear risk	Unclear risk	Unclear risk	High risk	Low risk		Moderate
Schennach 2018	Unclear risk	Unclear risk	Unclear risk	Unclear risk	Low risk	Low risk	Low risk		Moderate
See 1999	Unclear risk	Unclear risk	Unclear risk	Unclear risk	Unclear risk	High risk	Low risk		Moderate

Selman 1976	Unclear risk	Unclear risk	Low risk	Low risk	Low risk	Low risk	Low risk		Low
Sergi 2007	Low risk	Unclear risk	Unclear risk	Unclear risk	High risk	High risk	Low risk		High
Shah 2011	Unclear risk	Unclear risk	Low risk	Low risk	Low risk	High risk	Unclear risk		Moderate
Simpson 2004	Low risk	Low risk	Low risk	Low risk	Low risk	High risk	Low risk		Moderate
Sonmez 2009	Unclear risk	Unclear risk	High risk	High risk	High risk	Unclear risk	Unclear risk		High
Study 115 2000	Low risk	Unclear risk	Unclear risk	Unclear risk	Low risk	Unclear risk	Low risk		Moderate
Study 128-301 1997	Unclear risk	Unclear risk	Unclear risk	Unclear risk	Unclear risk	Unclear risk	Unclear risk		Moderate
Study 3001	Unclear risk	Unclear risk	Unclear risk	Unclear risk	Unclear risk	Unclear risk	Low risk		Moderate
Study 3002	Unclear risk	Unclear risk	Unclear risk	Unclear risk	Unclear risk	Unclear risk	Unclear risk		Moderate
Study 3003	Unclear risk	Unclear risk	Unclear risk	Unclear risk	Unclear risk	Unclear risk	Unclear risk		Moderate
Study 93202 2002	Unclear risk	Unclear risk	Unclear risk	Unclear risk	Low risk	Low risk	Low risk		Moderate
Study 94202 2002	Unclear risk	Unclear risk	Low risk	Low risk	Low risk	Low risk	Low risk		Low
Svestka 1990	Unclear risk	Unclear risk	Unclear risk	Unclear risk	Unclear risk	Unclear risk	Low risk		Moderate
Svestka 2003a	Low risk	Low risk	Low risk	Low risk	Unclear risk	Low risk	Low risk		Low
Svestka 2005	Low risk	Low risk	Low risk	Low risk	Unclear risk	Low risk	Low risk		Low
Tamrakar 2006	Unclear risk	Unclear risk	High risk	High risk	High risk	Low risk	Low risk		High
Taneli 2003	Unclear risk	Unclear risk	High risk	High risk	Low risk	Unclear risk	Unclear risk		High
Tollefson 1997	Low risk	Low risk	Unclear risk	Unclear risk	High risk	Low risk	Low risk		Moderate
Tuason 1984	Unclear risk	Unclear risk	Low risk	Low risk	Unclear risk	Low risk	Low risk		Low
Tybura 2014	Low risk	Low risk	High risk	Low risk	High risk	Unclear risk	Low risk		High
Wagner 2005	Low risk	Low risk	Unclear risk	Unclear risk	Low risk	Low risk	Low risk		Low
Weston 1973	Unclear risk	Unclear risk	Low risk	Low risk	High risk	Low risk	Low risk		Moderate
Wetzel 1991	Unclear risk	Unclear risk	Unclear risk	Unclear risk	High risk	Low risk	Low risk		Moderate
Wetzel 1998	Low risk	Low risk	Low risk	Low risk	Low risk	Low risk	High risk		Moderate
Yamashita 2004	Unclear risk	Unclear risk	Unclear risk	Unclear risk	Low risk	Low risk	Low risk		Moderate
Yen 2004	Unclear risk	Unclear risk	High risk	Unclear risk	Low risk	Low risk	Low risk		Moderate
Zborowski 1995	Low risk	Low risk	Low risk	Low risk	Low risk	Low risk	Low risk		Low
Zhang 2011	Low risk	Low risk	Low risk	Low risk	Low risk	Low risk	Low risk		Low
Zhong 2006	Low risk	Low risk	Low risk	Low risk	Low risk	Low risk	Low risk		Low
Ziegler 1989	Low risk	Low risk	Unclear risk	Unclear risk	High risk	Low risk	Low risk		Moderate

Zimbroff 1997	Low risk	Low risk	Unclear risk	Unclear risk	Low risk	High risk	Low risk		Moderate
Zimbroff 2007	Low risk	Low risk	Low risk	Low risk	Low risk	Low risk	Low risk		Low
de Boer 2011	Unclear risk	Unclear risk	High risk	High risk	High risk	Unclear risk	Low risk		High
de Oliviera 2009	Unclear risk	Unclear risk	High risk	High risk	Low risk	Low risk	Low risk		High
van Bruggen 2003	Unclear risk	Unclear risk	High risk	High risk	High risk	Low risk	Low risk		High
van der Velde 1975	Unclear risk	Unclear risk	Low risk	Low risk	Low risk	Unclear risk	Low risk		Low

eTable 2b: Risk of bias summary

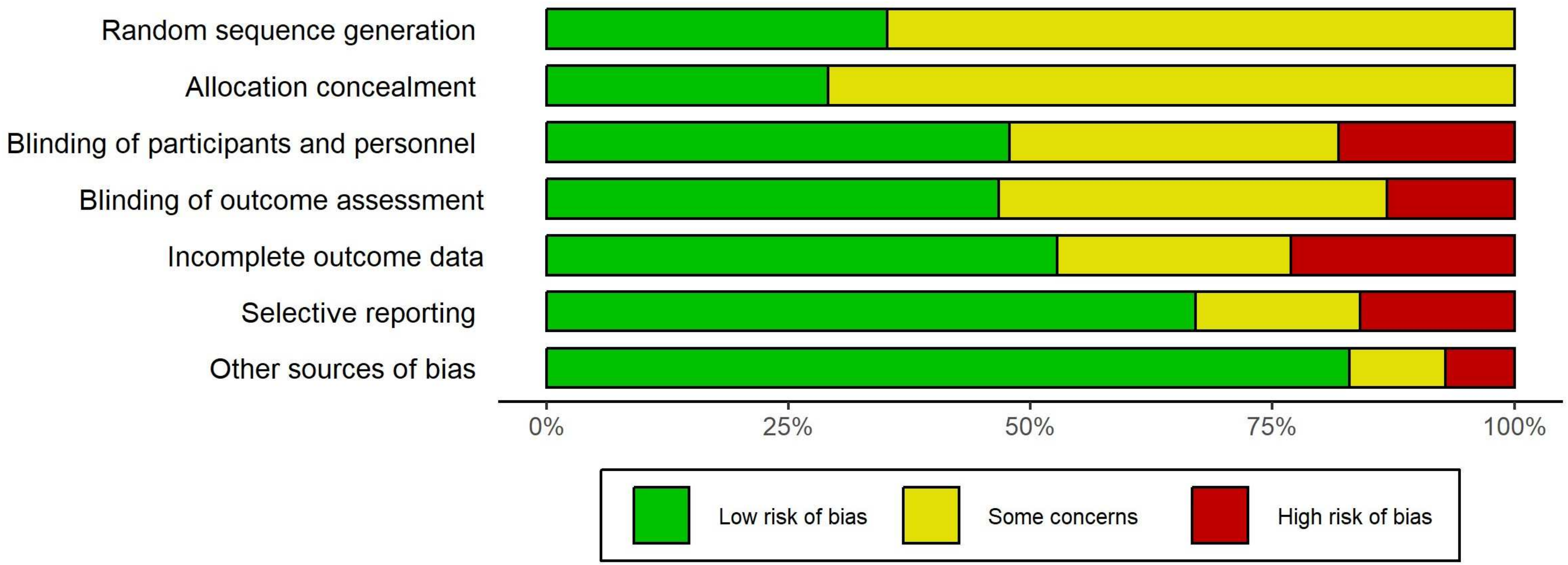

eFigure 2

Primary outcome Overall symptoms

Results of individual comparisons

eFigure 2 overall symptoms (primary outcome) individual comparisons

Study or Subgroup	first drug Mean	first drug SD	first drug Total	second drug Mean	second drug SD	second drug Total	Weight	SMD [95% CI]
**blinding = 0**								
de Oliviera 2009	69.70	22.9000	66	66.40	15.4000	31	14.1%	0.16 [−0.27; 0.58]
McCue2006	−12.90	11.8240	53	−16.40	11.8240	57	17.8%	0.29 [−0.08; 0.67]
**Total (95% CI)**			**119**			**88**	**31.9%**	**0.23 [−0.05; 0.52]**
Heterogeneity: $Tau^2 = 0$; $Chi^2 = 0.22$, df = 1 (P = 0.64); $I^2 = 0\%$								
**blinding = 1**								
Kane 2002	−13.44	24.4583	204	−13.80	24.4583	104	39.3%	0.01 [−0.22; 0.25]
Study 93202 2002	−11.10	15.7265	33	−15.80	15.7265	33	11.1%	0.30 [−0.19; 0.78]
Study 94202 2002	−15.90	25.2612	54	−10.40	25.2612	54	17.6%	−0.22 [−0.59; 0.16]
**Total (95% CI)**			**291**			**191**	**68.1%**	**0.00 [−0.18; 0.19]**
Heterogeneity: $Tau^2 = < 0.0001$; $Chi^2 = 2.69$, df = 2 (P = 0.26); $I^2 = 26\%$								
**Total (95% CI)**			**410**			**279**	**100.0%**	**0.08 [−0.09; 0.24]**

Heterogeneity: $Tau^2 = 0.0040$; $Chi^2 = 4.76$, df = 4 (P = 0.31); $I^2 = 16\%$

Test for subgroup differences: $Chi^2 = 1.85$, df = 1 (P = 0.17)

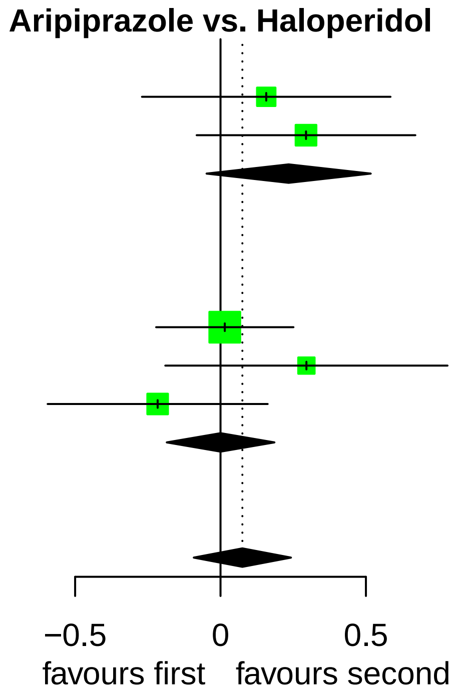

Study or Subgroup	first drug Mean	SD	Total	second drug Mean	SD	Total	Weight	SMD [95% CI]
**blinding = 1**								
Fleischhacker 2009_6weeks	−24.60	20.4892	347	−29.50	20.4892	344	41.9%	0.24 [0.09; 0.39]
Hatta 2009	−18.40	26.1940	21	−33.40	26.1940	17	2.2%	0.56 [−0.09; 1.21]
Jindal 2013	−45.31	11.9400	26	−40.93	5.4000	27	3.1%	−0.47 [−1.02; 0.08]
Kane 2009 8 weeks	−22.20	22.3000	285	−26.80	21.1000	281	34.4%	0.21 [0.05; 0.38]
McQuade 2004_6weeks	−28.21	20.4892	95	−30.71	20.4892	101	11.9%	0.12 [−0.16; 0.40]
**Total (95% CI)**			**774**			**770**	**93.6%**	**0.20 [0.10; 0.30]**
Heterogeneity: Tau^2 = < 0.0001; Chi^2 = 7.5, df = 4 (P = 0.11); I^2 = 47%								
**blinding = 0**								
McCue2006	−12.90	11.8240	53	−14.90	11.8240	52	6.4%	0.17 [−0.22; 0.55]
**Total (95% CI)**			**53**			**52**	**6.4%**	**0.17 [−0.22; 0.55]**
Heterogeneity: not applicable								
**Total (95% CI)**			**827**			**822**	**100.0%**	**0.20 [0.10; 0.29]**

Heterogeneity: Tau^2 < 0.0001; Chi^2 = 7.52, df = 5 (P = 0.18); I^2 = 34%

Test for subgroup differences: Chi^2 = 0.02, df = 1 (P = 0.88)

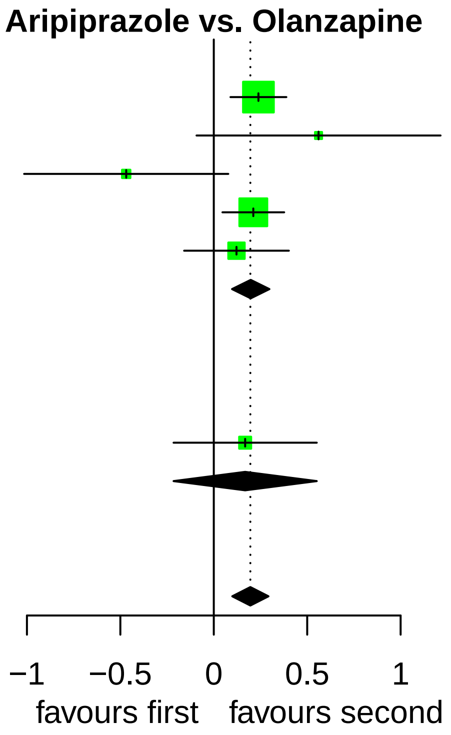

Study or Subgroup	first drug Mean	SD	Total	second drug Mean	SD	Total	Weight	SMD [95% CI]
**blinding = 1**								
Hatta 2009	−18.40	26.1940	21	−28.90	26.1940	20	28.1%	0.39 [−0.23; 1.01]
**Total (95% CI)**			**21**			**20**	**28.1%**	**0.39 [−0.23; 1.01]**
Heterogeneity: not applicable								
**blinding = 0**								
McCue2006	−12.90	11.8240	53	−14.20	11.8240	50	71.9%	0.11 [−0.28; 0.50]
**Total (95% CI)**			**53**			**50**	**71.9%**	**0.11 [−0.28; 0.50]**
Heterogeneity: not applicable								
**Total (95% CI)**			**74**			**70**	**100.0%**	**0.19 [−0.14; 0.52]**

Heterogeneity: $Tau^2 = 0$; $Chi^2 = 0.58$, df = 1 (P = 0.45); $I^2 = 0\%$

Test for subgroup differences: $Chi^2 = 0.58$, df = 1 (P = 0.45)

Aripiprazole vs. Quetiapine

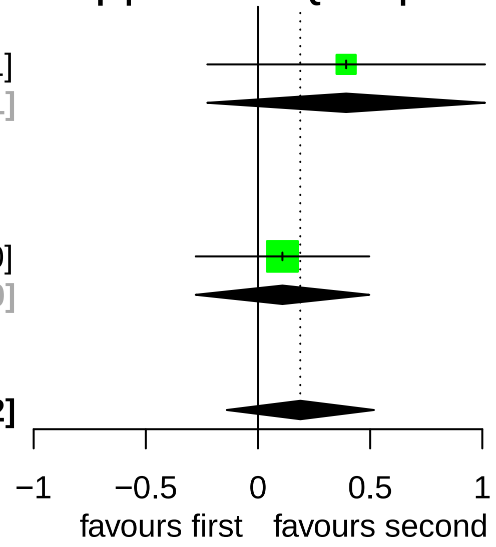

Study or Subgroup	first drug Mean	SD	Total	second drug Mean	SD	Total	Weight	SMD [95% CI]
**blinding = 1**								
Chan 2007	−19.60	18.1000	49	−21.10	17.1000	34	14.4%	0.08 [−0.35; 0.52]
Hatta 2009	−18.40	26.1940	21	−24.70	26.1940	20	7.3%	0.24 [−0.38; 0.85]
Potkin 2003	−14.20	21.1274	194	−15.70	21.1274	95	45.7%	0.07 [−0.17; 0.32]
**Total (95% CI)**			**264**			**149**	**67.4%**	**0.09 [−0.11; 0.29]**
Heterogeneity: $Tau^2 = 0$; $Chi^2 = 0.24$, df = 2 (P = 0.89); $I^2 = 0\%$								
**blinding = 0**								
de Boer 2011	−8.10	17.2000	17	−2.90	11.6000	15	5.6%	−0.34 [−1.04; 0.36]
Maat 2014	−17.24	15.8900	17	−12.85	17.5800	27	7.4%	−0.25 [−0.86; 0.36]
McCue2006	−12.90	11.8240	53	−15.40	11.8240	57	19.6%	0.21 [−0.17; 0.59]
**Total (95% CI)**			**87**			**99**	**32.6%**	**−0.04 [−0.43; 0.34]**
Heterogeneity: $Tau^2 = 0.0401$; $Chi^2 = 2.78$, df = 2 (P = 0.25); $I^2 = 28\%$								
**Total (95% CI)**			**351**			**248**	**100.0%**	**0.06 [−0.10; 0.23]**

Heterogeneity: $Tau^2 = 0$; $Chi^2 = 3.23$, df = 5 (P = 0.66); $I^2 = 0\%$

Test for subgroup differences: $Chi^2 = 0.38$, df = 1 (P = 0.54)

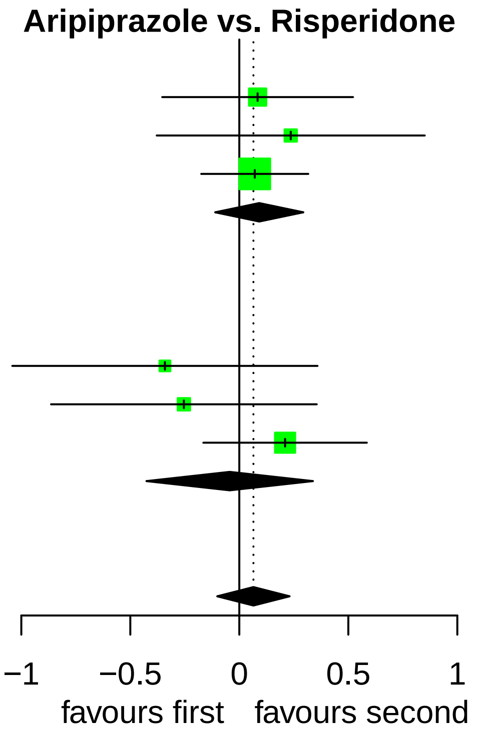

Study or Subgroup	first drug Mean	SD	Total	second drug Mean	SD	Total	Weight	SMD [95% CI]
**blinding = 0**								
Citrome 2015	−22.90	13.6000	64	−19.40	13.7900	33	43.6%	−0.25 [−0.68; 0.17]
**Total (95% CI)**			**64**			**33**	**43.6%**	**−0.25 [−0.68; 0.17]**
Heterogeneity: not applicable								
**blinding = 1**								
NCT00905307	−16.19	19.8009	90	−17.98	19.8009	50	56.4%	0.09 [−0.26; 0.44]
**Total (95% CI)**			**90**			**50**	**56.4%**	**0.09 [−0.26; 0.44]**
Heterogeneity: not applicable								
**Total (95% CI)**			**154**			**83**	**100.0%**	**−0.06 [−0.39; 0.27]**

Heterogeneity: $Tau^2 = 0.0205$; $Chi^2 = 1.53$, df = 1 (P = 0.22); $I^2 = 35\%$

Test for subgroup differences: $Chi^2 = 1.53$, df = 1 (P = 0.22)

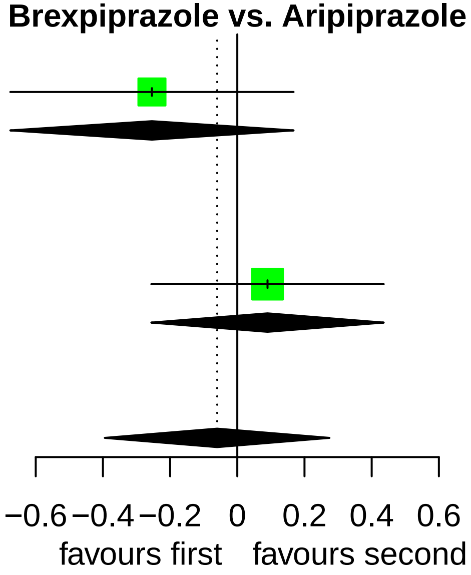

Study or Subgroup	first drug Mean	SD	Total	second drug Mean	SD	Total	Weight	SMD [95% CI]
**blinding = 1**								
Allan 1998	85.30	14.6000	10	83.40	26.2000	13	1.7%	0.08 [−0.74; 0.91]
Beasley 1996b	−12.90	15.7977	68	−13.93	15.7977	127	10.9%	0.06 [−0.23; 0.36]
Beasley 1997	−20.00	25.9000	79	−24.72	26.6400	168	12.6%	0.18 [−0.09; 0.45]
Bernardo 2001	62.70	20.7000	13	68.00	23.3000	14	2.0%	−0.23 [−0.99; 0.53]
Boulay 2007	56.10	12.9700	10	62.00	12.8400	14	1.7%	−0.44 [−1.26; 0.38]
Ishigooka 2001	−7.94	21.8500	78	−11.84	17.4200	80	9.9%	0.20 [−0.12; 0.51]
Lahti 2009	33.20	8.1000	12	33.80	6.9000	17	2.1%	−0.08 [−0.82; 0.66]
Rosenheck 2003w6	79.00	15.8000	115	79.90	15.5000	121	13.5%	−0.06 [−0.31; 0.20]
Tollefson 1997	−13.40	20.6000	636	−17.70	21.8000	1312	36.9%	0.20 [0.11; 0.30]
**Total (95% CI)**			**1021**			**1866**	**91.3%**	**0.12 [0.01; 0.23]**
Heterogeneity: $Tau^2 = 0.0044$; $Chi^2 = 7.44$, df = 8 (P = 0.49); $I^2 = 0\%$								
**blinding = 0**								
Avasthi 2001	12.57	13.3900	7	9.50	7.0600	16	1.5%	0.32 [−0.58; 1.21]
McCue2006	−16.40	11.8240	57	−14.90	11.8240	52	7.3%	−0.13 [−0.50; 0.25]
**Total (95% CI)**			**64**			**68**	**8.7%**	**−0.06 [−0.41; 0.29]**
Heterogeneity: $Tau^2 = 0$; $Chi^2 = 0.8$, df = 1 (P = 0.37); $I^2 = 0\%$								
**Total (95% CI)**			**1085**			**1934**	**100.0%**	**0.10 [−0.01; 0.21]**

Heterogeneity: $Tau^2 = 0.0061$; $Chi^2 = 9.59$, df = 10 (P = 0.48); $I^2 = 0\%$
Test for subgroup differences: $Chi^2 = 0.93$, df = 1 (P = 0.34)

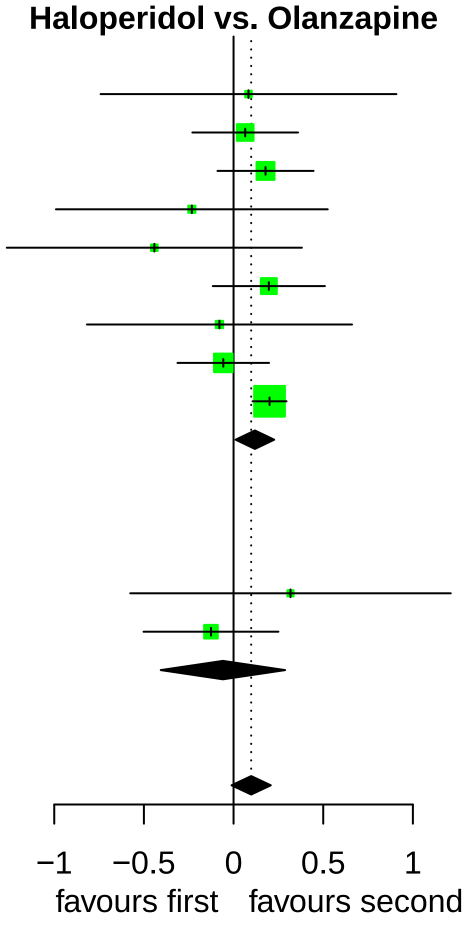

Study or Subgroup	first drug Mean	first drug SD	first drug Total	second drug Mean	second drug SD	second drug Total	Weight	SMD [95% CI]
**blinding = 1**								
Abdolahian 2008	86.10	17.0000	30	71.30	17.0000	35	5.5%	0.86 [0.35; 1.37]
Blin 1996	−26.60	27.1611	20	−44.70	27.1611	21	4.4%	0.65 [0.02; 1.28]
Borison 1992	−9.00	16.5414	53	−11.60	16.5414	53	6.9%	0.16 [−0.23; 0.54]
Ceskova 1993	28.58	6.4600	31	32.48	10.2400	31	5.5%	−0.45 [−0.95; 0.05]
Chouinard 1993	−9.30	25.5028	21	−25.70	25.5028	22	4.6%	0.63 [0.02; 1.25]
Claus 1992	74.30	20.1600	21	76.90	20.1600	21	4.6%	−0.13 [−0.73; 0.48]
Ghaleiha 2011	−37.87	29.3861	16	−43.62	29.3861	16	3.9%	0.19 [−0.50; 0.89]
Janicak 2001	−14.00	27.7400	32	−16.00	21.4200	30	5.6%	0.08 [−0.42; 0.58]
Liu 2000	−31.60	20.6000	19	−24.70	15.7000	19	4.3%	−0.37 [−1.01; 0.27]
Marder 1994	−4.10	20.3186	64	−16.10	20.3186	63	7.2%	0.59 [0.23; 0.94]
Min 1993	−21.90	9.3300	19	−17.10	11.6700	16	4.1%	−0.45 [−1.12; 0.23]
Murasaki 1993	20.30	12.2000	95	21.20	13.9000	97	8.0%	−0.07 [−0.35; 0.21]
Peuskens 1995	−15.00	21.8000	223	−18.25	23.5080	455	9.3%	0.14 [−0.02; 0.30]
See 1999	67.55	11.4500	10	59.95	9.6400	10	2.8%	0.69 [−0.22; 1.60]
Svestka 1990	20.70	11.3431	18	24.70	11.3431	18	4.2%	−0.34 [−1.00; 0.31]
Yen 2004	−24.80	18.5000	20	−29.80	15.7000	21	4.5%	0.29 [−0.33; 0.90]
**Total (95% CI)**			**692**			**928**	**85.5%**	**0.15 [−0.05; 0.35]**
Heterogeneity: $Tau^2 = 0.0906$; $Chi^2 = 35.9$, df = 15 ($P < 0.01$); $I^2 = 58\%$								
**blinding = 0**								
Fakra 2008	57.27	12.9200	14	54.70	9.5000	11	3.3%	0.22 [−0.58; 1.01]
McCue2006	−16.40	11.8240	57	−15.40	11.8240	57	7.0%	−0.08 [−0.45; 0.28]
Tamrakar 2006	−43.17	12.6400	18	−52.11	12.2000	18	4.1%	0.70 [0.03; 1.38]
**Total (95% CI)**			**89**			**86**	**14.5%**	**0.22 [−0.27; 0.71]**
Heterogeneity: $Tau^2 = 0.0976$; $Chi^2 = 4.11$, df = 2 ($P = 0.13$); $I^2 = 51\%$								
**Total (95% CI)**			**781**			**1014**	**100.0%**	**0.16 [−0.02; 0.34]**

Heterogeneity: $Tau^2 = 0.0804$; $Chi^2 = 40.05$, df = 18 ($P < 0.01$); $I^2 = 55\%$

Test for subgroup differences: $Chi^2 = 0.06$, df = 1 ($P = 0.81$)

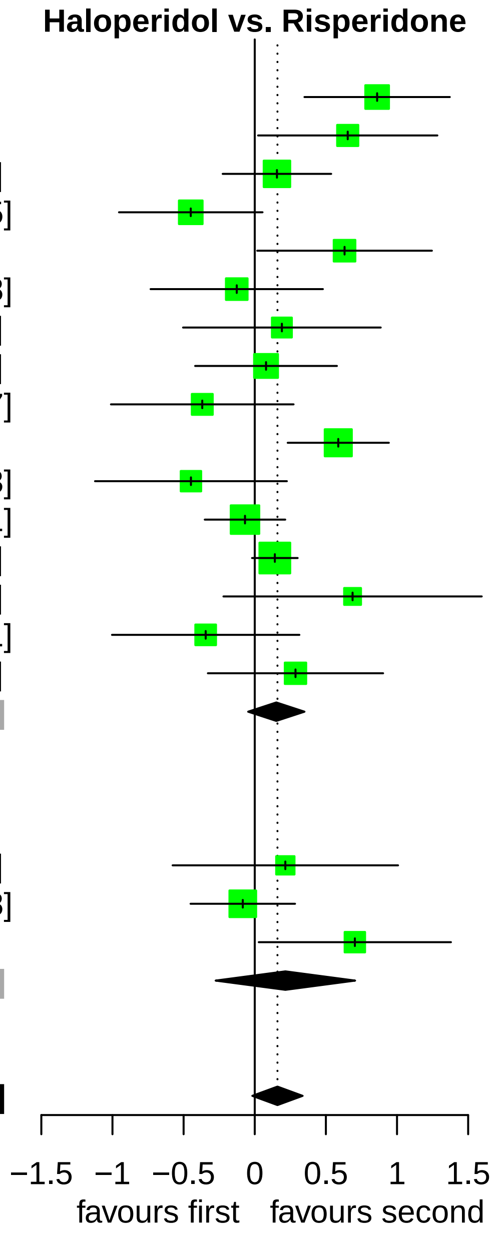

Study or Subgroup	first drug Mean	first drug SD	first drug Total	second drug Mean	second drug SD	second drug Total	Weight	SMD [95% CI]
blinding = 1								
Brook 1998	−5.50	6.0300	20	−6.04	6.0300	24	21.3%	0.09 [−0.51; 0.68]
Heikkila 1981	26.64	8.2555	19	22.98	8.2555	17	17.1%	0.43 [−0.23; 1.10]
Heikkilae 1992	10.30	7.9341	18	11.60	8.5329	20	18.5%	−0.15 [−0.79; 0.48]
Total (95% CI)			57			61	56.9%	0.11 [−0.25; 0.48]
Heterogeneity: Tau^2 = 0; Chi^2 = 1.58, df = 2 (P = 0.45); I^2 = 0%								
blinding = 0								
Lamure 2003	21.70	13.6100	45	21.70	13.6100	43	43.1%	0.00 [−0.42; 0.42]
Total (95% CI)			45			43	43.1%	0.00 [−0.42; 0.42]
Heterogeneity: not applicable								
**Total (95% CI)**			**102**			**104**	**100.0%**	**0.06 [−0.21; 0.34]**

Heterogeneity: Tau^2 = 0; Chi^2 = 1.74, df = 3 (P = 0.63); I^2 = 0%

Test for subgroup differences: Chi^2 = 0.16, df = 1 (P = 0.69)

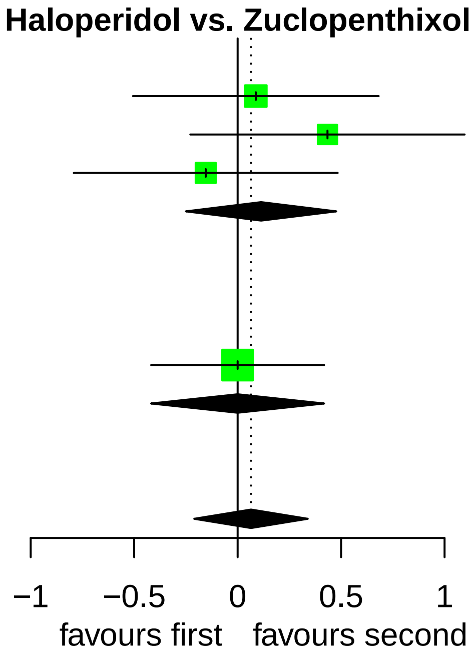

Study or Subgroup	first drug Mean	SD	Total	second drug Mean	SD	Total	Weight	SMD [95% CI]
**blinding = 1**								
Arvanitis 1997	−6.99	14.7899	104	−7.58	14.7899	50	13.8%	0.04 [−0.30; 0.38]
Copolov 2000	−18.70	24.0700	218	−22.10	24.1200	219	44.7%	0.14 [−0.05; 0.33]
Murasaki 2001	78.50	26.5000	97	78.30	25.7000	90	19.1%	0.01 [−0.28; 0.29]
**Total (95% CI)**			**419**			**359**	**77.6%**	**0.09 [−0.05; 0.23]**
Heterogeneity: $Tau^2 = 0$; $Chi^2 = 0.68$, df = 2 (P = 0.71); $I^2 = 0\%$								
**blinding = 0**								
Atmaca 2002	75.08	5.6500	18	74.43	5.4200	17	3.6%	0.11 [−0.55; 0.78]
McCue2006	−14.20	11.8240	50	−16.40	11.8240	57	10.9%	0.18 [−0.20; 0.57]
Taneli 2003	75.20	29.3000	45	72.20	27.4000	34	7.9%	0.10 [−0.34; 0.55]
**Total (95% CI)**			**113**			**108**	**22.4%**	**0.15 [−0.12; 0.41]**
Heterogeneity: $Tau^2 = 0$; $Chi^2 = 0.08$, df = 2 (P = 0.96); $I^2 = 0\%$								
**Total (95% CI)**			**532**			**467**	**100.0%**	**0.10 [−0.02; 0.23]**

Heterogeneity: $Tau^2 = 0$; $Chi^2 = 0.89$, df = 5 (P = 0.97); $I^2 = 0\%$

Test for subgroup differences: $Chi^2 = 0.13$, df = 1 (P = 0.72)

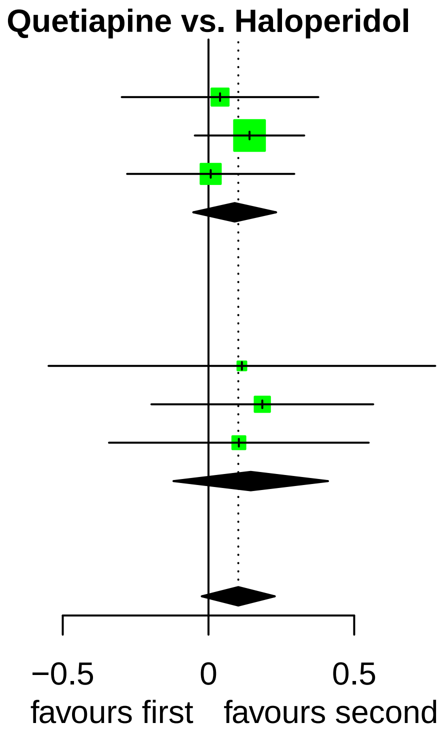

Study or Subgroup	first drug Mean	SD	Total	second drug Mean	SD	Total	Weight	SMD [95% CI]
**blinding = 1**								
Hatta 2009	−28.90	26.1940	20	−33.40	26.1940	17	3.6%	0.17 [−0.48; 0.82]
Lieberman 2005 12weeks	−3.76	13.4568	333	−6.38	13.4568	331	65.6%	0.19 [0.04; 0.35]
Mori 2004	72.90	12.7758	20	69.40	12.7758	20	3.9%	0.27 [−0.35; 0.89]
Riedel 2007	−21.50	23.3900	16	−17.88	20.7100	17	3.3%	−0.16 [−0.84; 0.52]
Sacchetti 2008	−38.15	20.4892	25	−35.83	20.4892	25	5.0%	−0.11 [−0.67; 0.44]
Svestka 2003a	−43.91	20.9400	22	−45.65	11.9600	20	4.2%	0.10 [−0.51; 0.70]
**Total (95% CI)**			**436**			**430**	**85.5%**	**0.16 [0.03; 0.29]**
Heterogeneity: $Tau^2 = 0$; $Chi^2 = 2.11$, df = 5 (P = 0.83); $I^2 = 0\%$								
**blinding = 0**								
McCue2006	−14.20	11.8240	50	−14.90	11.8240	52	10.1%	0.06 [−0.33; 0.45]
Yamashita 2004	−4.00	6.2791	28	−8.30	6.2791	20	4.4%	0.67 [0.08; 1.26]
**Total (95% CI)**			**78**			**72**	**14.5%**	**0.32 [−0.27; 0.92]**
Heterogeneity: $Tau^2 = 0.1240$; $Chi^2 = 2.91$, df = 1 (P = 0.09); $I^2 = 66\%$								
**Total (95% CI)**			**514**			**502**	**100.0%**	**0.17 [0.05; 0.30]**
Heterogeneity: $Tau^2 = 0$; $Chi^2 = 5.24$, df = 7 (P = 0.63); $I^2 = 0\%$								
Test for subgroup differences: $Chi^2 = 0.27$, df = 1 (P = 0.60)								

Quetiapine vs. Olanzapine

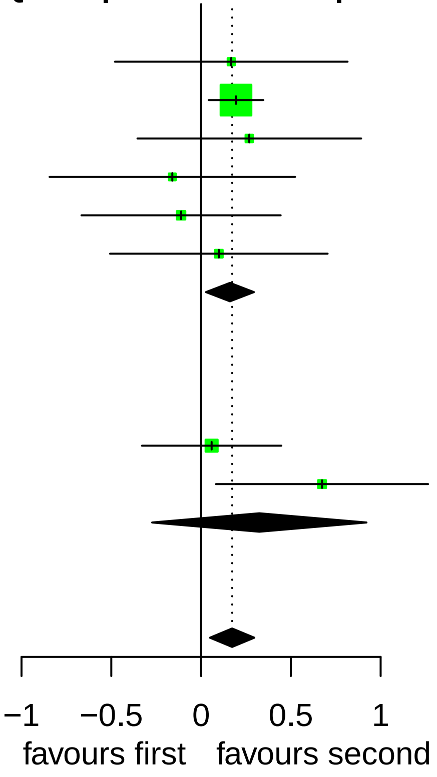

Study or Subgroup	first drug Mean	first drug SD	first drug Total	second drug Mean	second drug SD	second drug Total	Weight	SMD [95% CI]
**blinding = 1**								
Hatta 2009	−28.90	26.1940	20	−24.70	26.1940	20	3.1%	−0.16 [−0.78; 0.46]
Li 2012	−31.90	17.5000	60	−33.30	17.3000	59	8.6%	0.08 [−0.28; 0.44]
Lieberman 2005 12weeks	−3.76	13.4568	333	−3.10	13.4568	339	30.9%	−0.05 [−0.20; 0.10]
Moosavi 2015	−30.20	4.8700	45	−32.30	4.0700	45	6.5%	0.46 [0.04; 0.88]
Mori 2004	72.90	12.7758	20	71.50	12.7758	19	3.0%	0.11 [−0.52; 0.74]
Sacchetti 2008	−38.15	20.4892	25	−32.74	20.4892	25	3.8%	−0.26 [−0.82; 0.30]
Zhong 2006	−15.10	25.3600	328	−18.10	25.0000	318	30.2%	0.12 [−0.04; 0.27]
**Total (95% CI)**			**831**			**825**	**86.1%**	**0.06 [−0.07; 0.18]**
Heterogeneity: $Tau^2 = 0.0066$; $Chi^2 = 7.84$, df = 6 (P = 0.25); $I^2 = 23\%$								
**blinding = 0**								
Knegtering 2004	−5.40	12.3000	18	−8.40	11.2000	15	2.6%	0.25 [−0.44; 0.94]
McCue2006	−14.20	11.8240	50	−15.40	11.8240	57	7.8%	0.10 [−0.28; 0.48]
Yamashita 2004	−4.00	6.2791	28	−6.20	6.2791	20	3.6%	0.34 [−0.23; 0.92]
**Total (95% CI)**			**96**			**92**	**13.9%**	**0.19 [−0.10; 0.48]**
Heterogeneity: $Tau^2 = 0$; $Chi^2 = 0.51$, df = 2 (P = 0.77); $I^2 = 0\%$								
**Total (95% CI)**			**927**			**917**	**100.0%**	**0.07 [−0.04; 0.18]**

Heterogeneity: $Tau^2 = 0.0047$; $Chi^2 = 9.16$, df = 9 (P = 0.42); $I^2 = 2\%$

Test for subgroup differences: $Chi^2 = 0.67$, df = 1 (P = 0.41)

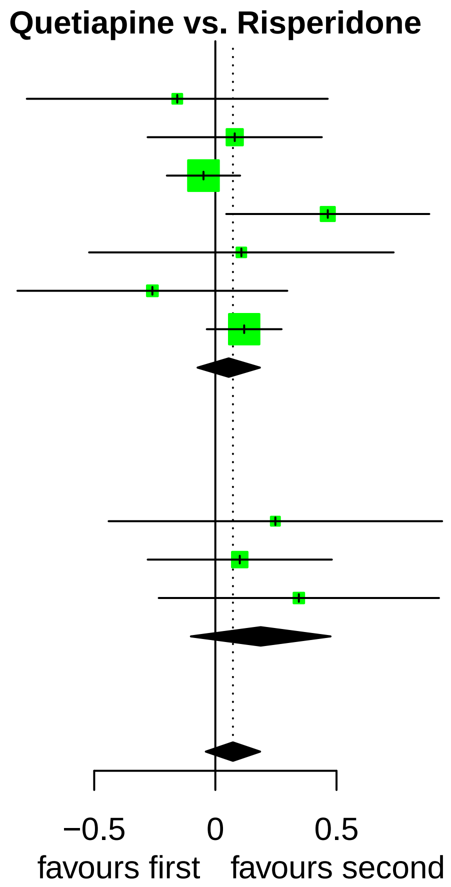

Study or Subgroup	first drug Mean	first drug SD	first drug Total	second drug Mean	second drug SD	second drug Total	Weight	SMD [95% CI]
**blinding = 1**								
Canive 2006	70.60	16.7528	4	57.80	16.7528	5	0.8%	0.68 [−0.70; 2.06]
Conley 2001	−12.80	15.9000	175	−12.90	16.1000	181	24.5%	0.01 [−0.20; 0.21]
Hatta 2009	−24.70	26.1940	20	−33.40	26.1940	17	3.6%	0.32 [−0.33; 0.98]
Hatta 2013	73.30	22.6100	20	71.09	27.3145	22	4.2%	0.09 [−0.52; 0.69]
Lieberman 2005 12weeks	−3.10	13.4568	339	−6.38	13.4568	331	35.2%	0.24 [0.09; 0.40]
Mori 2004	71.50	12.7758	19	69.40	12.7758	20	3.9%	0.16 [−0.47; 0.79]
Sacchetti 2008	−32.74	20.4892	25	−35.83	20.4892	25	4.9%	0.15 [−0.41; 0.70]
**Total (95% CI)**			**602**			**601**	**77.1%**	**0.16 [0.01; 0.31]**
Heterogeneity: $Tau^2 = 0.0067$; $Chi^2 = 4.1$, df = 6 (P = 0.66); $I^2 = 0\%$								
**blinding = 0**								
Chen 2018	79.20	19.0194	25	67.11	19.0194	28	4.9%	0.63 [0.07; 1.18]
McCue2006	−15.40	11.8240	57	−14.90	11.8240	52	9.9%	−0.04 [−0.42; 0.33]
van Bruggen 2003	−15.00	12.7400	24	−15.10	23.7600	18	4.1%	0.01 [−0.61; 0.62]
Yamashita 2004	−6.20	6.2791	20	−8.30	6.2791	20	3.9%	0.33 [−0.30; 0.95]
**Total (95% CI)**			**126**			**118**	**22.9%**	**0.20 [−0.13; 0.52]**
Heterogeneity: $Tau^2 = 0.0371$; $Chi^2 = 4.36$, df = 3 (P = 0.23); $I^2 = 31\%$								
**Total (95% CI)**			**728**			**719**	**100.0%**	**0.16 [0.03; 0.29]**
Heterogeneity: $Tau^2 = 0.0060$; $Chi^2 = 8.46$, df = 10 (P = 0.58); $I^2 = 0\%$								
Test for subgroup differences: $Chi^2 = 0.04$, df = 1 (P = 0.83)								

Risperidone vs. Olanzapine

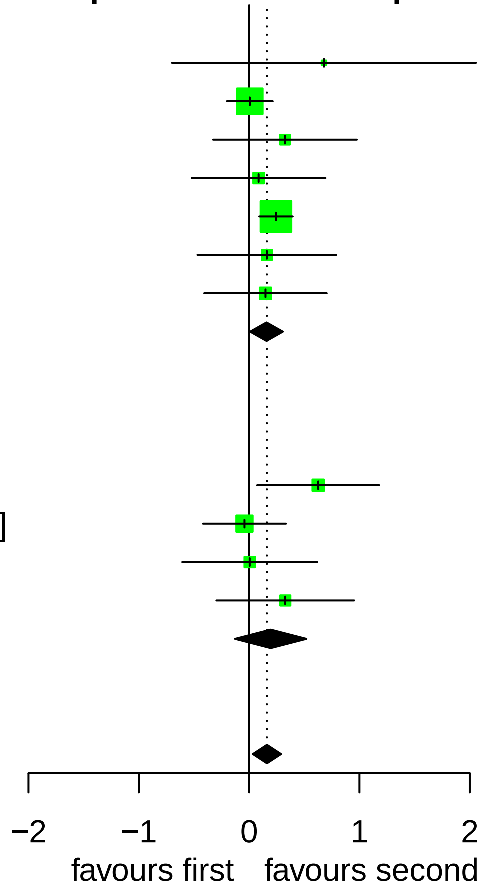

Study or Subgroup	first drug Mean	SD	Total	second drug Mean	SD	Total	Weight	SMD [95% CI]
**blinding = 0**								
McCue2006	−14.20	11.8240	50	−12.90	11.8240	53	34.1%	−0.11 [−0.50; 0.28]
**Total (95% CI)**			**50**			**53**	**34.1%**	**−0.11 [−0.50; 0.28]**
Heterogeneity: not applicable								
**blinding = 1**								
Zimbroff 2007	−21.60	18.7000	121	−24.60	19.1000	126	65.9%	0.16 [−0.09; 0.41]
**Total (95% CI)**			**121**			**126**	**65.9%**	**0.16 [−0.09; 0.41]**
Heterogeneity: not applicable								
**Total (95% CI)**			**171**			**179**	**100.0%**	**0.07 [−0.18; 0.32]**

Heterogeneity: $Tau^2 = 0.0081$; $Chi^2 = 1.30$, df = 1 (P = 0.26); $I^2 = 23\%$

Test for subgroup differences: $Chi^2 = 1.30$, df = 1 (P = 0.26)

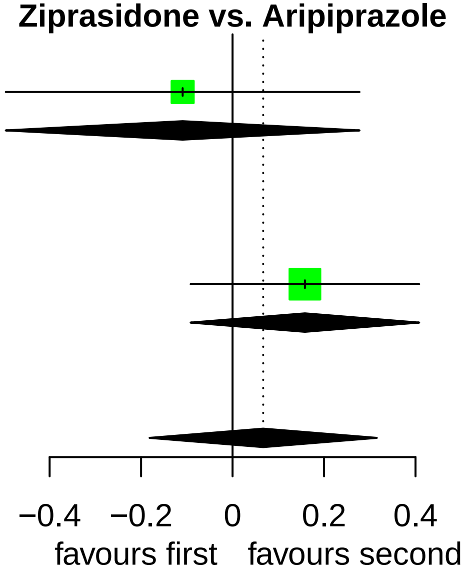

Study or Subgroup	first drug Mean	SD	Total	second drug Mean	SD	Total	Weight	SMD [95% CI]
**blinding = 1**								
Brook 2005	−14.99	19.2800	429	−15.79	15.9900	138	27.9%	0.04 [−0.15; 0.23]
Corripio 2005	61.10	16.7528	10	58.00	16.7528	10	5.3%	0.18 [−0.70; 1.06]
Goff 1998	−11.90	15.0000	20	−11.60	15.1000	17	8.8%	−0.02 [−0.67; 0.63]
Schennach 2018	66.23	23.0100	54	74.99	20.3800	58	17.5%	−0.40 [−0.78; −0.03]
Study 115 2000	−8.44	22.9324	158	−15.20	22.9324	82	23.2%	0.29 [0.03; 0.56]
**Total (95% CI)**			**671**			**305**	**82.8%**	**0.02 [−0.25; 0.29]**
Heterogeneity: $Tau^2 = 0.0498$; $Chi^2 = 8.88$, df = 4 (P = 0.06); $I^2 = 55\%$								
**blinding = 0**								
McCue2006	−14.20	11.8240	50	−16.40	11.8240	57	17.2%	0.18 [−0.20; 0.57]
**Total (95% CI)**			**50**			**57**	**17.2%**	**0.18 [−0.20; 0.57]**
Heterogeneity: not applicable								
**Total (95% CI)**			**721**			**362**	**100.0%**	**0.05 [−0.17; 0.27]**

Heterogeneity: $Tau^2 = 0.0357$; $Chi^2 = 9.31$, df = 5 (P = 0.10); $I^2 = 46\%$

Test for subgroup differences: $Chi^2 = 0.49$, df = 1 (P = 0.48)

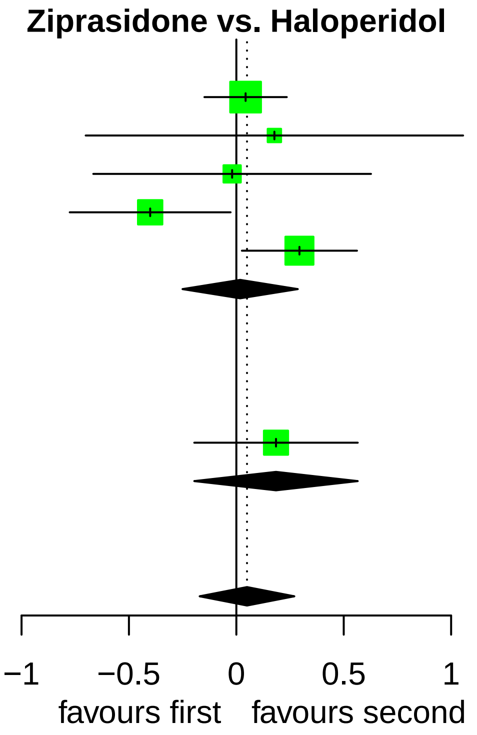

Study or	first drug			second drug				
Subgroup	Mean	SD	Total	Mean	SD	Total	Weight	SMD [95% CI]
**blinding = 1**								
Grootens 2009	−14.86	12.9900	34	−17.15	10.2500	33	8.4%	0.19 [−0.29; 0.67]
Lieberman 2005 12weeks	−2.74	13.4568	183	−6.38	13.4568	331	45.1%	0.27 [0.09; 0.45]
Simpson 2004	−11.45	13.5300	125	−11.95	13.5300	126	27.8%	0.04 [−0.21; 0.28]
Svestka 2005	60.29	18.3300	24	51.92	18.3300	24	6.0%	0.45 [−0.12; 1.02]
**Total (95% CI)**			**366**			**514**	**87.4%**	**0.20 [0.04; 0.36]**
Heterogeneity: Tau^2 = 0.0053; Chi^2 = 2.97, df = 3 (P = 0.40); I^2 = 0%								
**blinding = 0**								
McCue2006	−14.20	11.8240	50	−14.90	11.8240	52	12.6%	0.06 [−0.33; 0.45]
**Total (95% CI)**			**50**			**52**	**12.6%**	**0.06 [−0.33; 0.45]**
Heterogeneity: not applicable								
**Total (95% CI)**			**416**			**566**	**100.0%**	**0.18 [0.04; 0.33]**
Heterogeneity: Tau^2 = 0.0033; Chi^2 = 3.44, df = 4 (P = 0.49); I^2 = 0%								
Test for subgroup differences: Chi^2 = 0.43, df = 1 (P = 0.51)								

Ziprasidone vs. Olanzapine

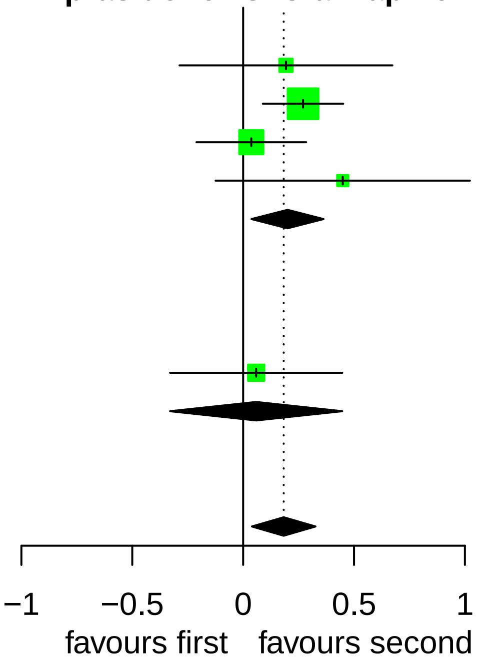

Study or Subgroup	first drug Mean	SD	Total	second drug Mean	SD	Total	Weight	SMD [95% CI]
**blinding = 1**								
Lieberman 2005 12weeks	−2.74	13.4568	183	−3.76	13.4568	333	82.5%	0.08 [−0.10; 0.26]
**Total (95% CI)**			**183**			**333**	**82.5%**	**0.08 [−0.10; 0.26]**
Heterogeneity: not applicable								
**blinding = 0**								
McCue2006	−14.20	11.8240	50	−14.20	11.8240	50	17.5%	0.00 [−0.39; 0.39]
**Total (95% CI)**			**50**			**50**	**17.5%**	**0.00 [−0.39; 0.39]**
Heterogeneity: not applicable								
**Total (95% CI)**			**233**			**383**	**100.0%**	**0.06 [−0.10; 0.23]**

Heterogeneity: $Tau^2 = 0$; $Chi^2 = 0.12$, df = 1 (P = 0.73); $I^2 = 0\%$

Test for subgroup differences: $Chi^2 = 0.12$, df = 1 (P = 0.73)

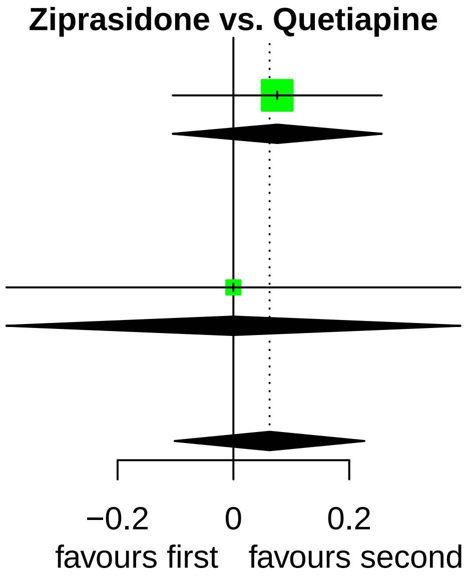

Study or Subgroup	first drug Mean	SD	Total	second drug Mean	SD	Total	Weight	SMD [95% CI]
**blinding = 1**								
Addington 2004	−25.80	20.4892	125	−27.30	20.4892	132	24.1%	0.07 [−0.17; 0.32]
Lieberman 2005 12weeks	−2.74	13.4568	183	−3.10	13.4568	339	44.7%	0.03 [−0.15; 0.21]
Zhang 2011	−35.60	14.9969	96	−37.10	14.7822	111	19.3%	0.10 [−0.17; 0.37]
**Total (95% CI)**			**404**			**582**	**88.1%**	**0.06 [−0.07; 0.18]**
Heterogeneity: $Tau^2 = 0$; $Chi^2 = 0.22$, df = 2 (P = 0.90); $I^2 = 0\%$								
**blinding = 0**								
McCue2006	−14.20	11.8240	50	−15.40	11.8240	57	10.0%	0.10 [−0.28; 0.48]
Sonmez 2009	64.30	18.0600	10	62.00	23.1400	10	1.9%	0.11 [−0.77; 0.98]
**Total (95% CI)**			**60**			**67**	**11.9%**	**0.10 [−0.25; 0.45]**
Heterogeneity: $Tau^2 = 0$; $Chi^2 = 0$, df = 1 (P = 0.99); $I^2 = 0\%$								
**Total (95% CI)**			**464**			**649**	**100.0%**	**0.06 [−0.06; 0.18]**

Heterogeneity: $Tau^2 = 0$; $Chi^2 = 0.28$, df = 4 (P = 0.99); $I^2 = 0\%$

Test for subgroup differences: $Chi^2 = 0.06$, df = 1 (P = 0.81)

Ziprasidone vs. Risperidone

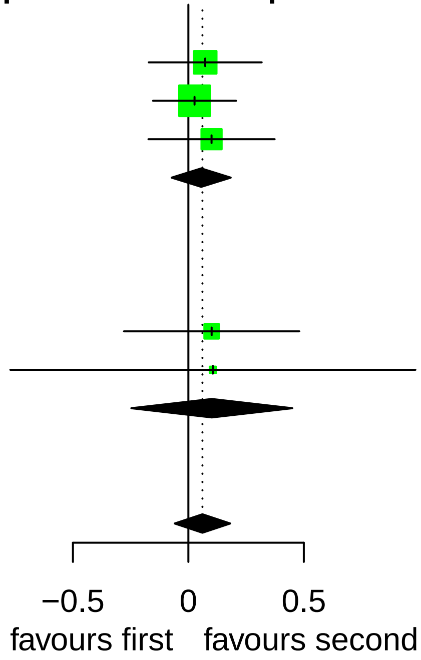

eFigure 3

Sensitivity analyses of the primary outcome

Fixed effects model

eFigure 3a overall symptoms sensitivity analysis fixed effects model individual comparisons

Study or Subgroup	first drug Mean	first drug SD	first drug Total	second drug Mean	second drug SD	second drug Total	Weight	SMD [95% CI]
**blinding = 0**								
de Oliviera 2009	69.70	22.9000	66	66.40	15.4000	31	13.1%	0.16 [−0.27; 0.58]
McCue2006	−12.90	11.8240	53	−16.40	11.8240	57	17.0%	0.29 [−0.08; 0.67]
**Total (95% CI)**			**119**			**88**	**30.1%**	**0.23 [−0.05; 0.52]**
Heterogeneity: $Tau^2 = 0$; $Chi^2 = 0.22$, df = 1 (P = 0.64); $I^2 = 0\%$								
**blinding = 1**								
Kane 2002	−13.44	24.4583	204	−13.80	24.4583	104	43.0%	0.01 [−0.22; 0.25]
Study 93202 2002	−11.10	15.7265	33	−15.80	15.7265	33	10.2%	0.30 [−0.19; 0.78]
Study 94202 2002	−15.90	25.2612	54	−10.40	25.2612	54	16.7%	−0.22 [−0.59; 0.16]
**Total (95% CI)**			**291**			**191**	**69.9%**	**0.00 [−0.18; 0.19]**
Heterogeneity: $Tau^2 = < 0.0001$; $Chi^2 = 2.69$, df = 2 (P = 0.26); $I^2 = 26\%$								
**Total (95% CI)**			**410**			**279**	**100.0%**	**0.07 [−0.08; 0.23]**

Heterogeneity: $Tau^2 = 0.0040$; $Chi^2 = 4.76$, df = 4 (P = 0.31); $I^2 = 16\%$

Test for subgroup differences: $Chi^2 = 1.85$, df = 1 (P = 0.17)

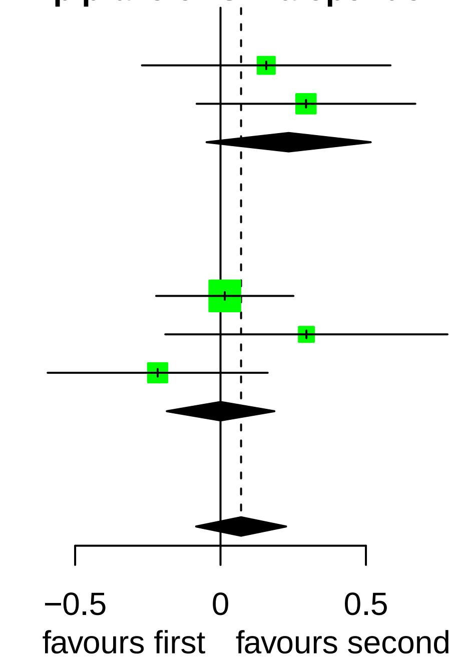

Study or Subgroup	first drug Mean	SD	Total	second drug Mean	SD	Total	Weight	SMD [95% CI]
**blinding = 1**								
Fleischhacker 2009_6weeks	−24.60	20.4892	347	−29.50	20.4892	344	41.9%	0.24 [0.09; 0.39]
Hatta 2009	−18.40	26.1940	21	−33.40	26.1940	17	2.2%	0.56 [−0.09; 1.21]
Jindal 2013	−45.31	11.9400	26	−40.93	5.4000	27	3.1%	−0.47 [−1.02; 0.08]
Kane 2009 8 weeks	−22.20	22.3000	285	−26.80	21.1000	281	34.4%	0.21 [0.05; 0.38]
McQuade 2004_6weeks	−28.21	20.4892	95	−30.71	20.4892	101	11.9%	0.12 [−0.16; 0.40]
**Total (95% CI)**			**774**			**770**	**93.6%**	**0.20 [0.10; 0.30]**
Heterogeneity: Tau^2 = < 0.0001; Chi^2 = 7.5, df = 4 (P = 0.11); I^2 = 47%								
**blinding = 0**								
McCue2006	−12.90	11.8240	53	−14.90	11.8240	52	6.4%	0.17 [−0.22; 0.55]
**Total (95% CI)**			**53**			**52**	**6.4%**	**0.17 [−0.22; 0.55]**
Heterogeneity: not applicable								
**Total (95% CI)**			**827**			**822**	**100.0%**	**0.20 [0.10; 0.29]**

Heterogeneity: Tau^2 < 0.0001; Chi^2 = 7.52, df = 5 (P = 0.18); I^2 = 34%

Test for subgroup differences: Chi^2 = 0.02, df = 1 (P = 0.88)

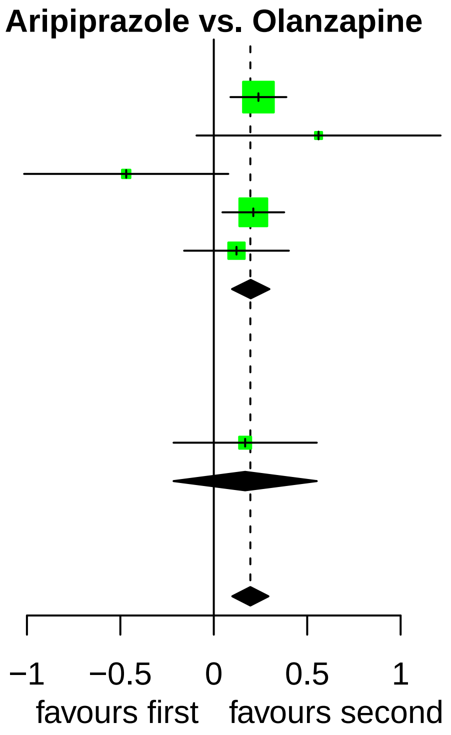

Study or Subgroup	first drug Mean	first drug SD	first drug Total	second drug Mean	second drug SD	second drug Total	Weight	SMD [95% CI]
**blinding = 1**								
Hatta 2009	−18.40	26.1940	21	−28.90	26.1940	20	28.1%	0.39 [−0.23; 1.01]
**Total (95% CI)**			**21**			**20**	**28.1%**	**0.39 [−0.23; 1.01]**
Heterogeneity: not applicable								
**blinding = 0**								
McCue2006	−12.90	11.8240	53	−14.20	11.8240	50	71.9%	0.11 [−0.28; 0.50]
**Total (95% CI)**			**53**			**50**	**71.9%**	**0.11 [−0.28; 0.50]**
Heterogeneity: not applicable								
**Total (95% CI)**			**74**			**70**	**100.0%**	**0.19 [−0.14; 0.52]**

Heterogeneity: $Tau^2 = 0$; $Chi^2 = 0.58$, df = 1 (P = 0.45); $I^2 = 0\%$

Test for subgroup differences: $Chi^2 = 0.58$, df = 1 (P = 0.45)

Aripiprazole vs. Quetiapine

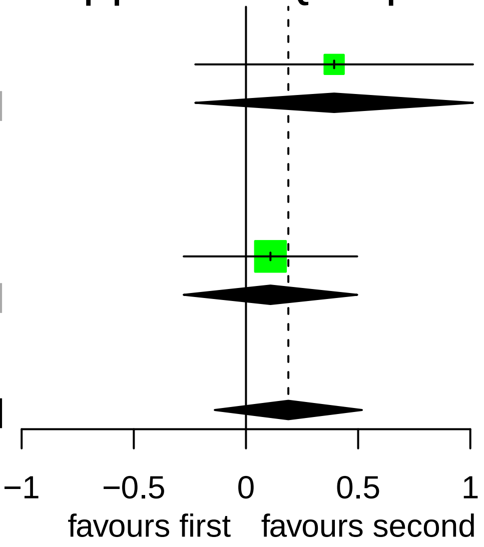

Study or Subgroup	first drug Mean	SD	Total	second drug Mean	SD	Total	Weight	SMD [95% CI]
**blinding = 1**								
Chan 2007	−19.60	18.1000	49	−21.10	17.1000	34	14.4%	0.08 [−0.35; 0.52]
Hatta 2009	−18.40	26.1940	21	−24.70	26.1940	20	7.3%	0.24 [−0.38; 0.85]
Potkin 2003	−14.20	21.1274	194	−15.70	21.1274	95	45.7%	0.07 [−0.17; 0.32]
**Total (95% CI)**			**264**			**149**	**67.4%**	**0.09 [−0.11; 0.29]**
Heterogeneity: $Tau^2 = 0$; $Chi^2 = 0.24$, df = 2 (P = 0.89); $I^2 = 0\%$								
**blinding = 0**								
de Boer 2011	−8.10	17.2000	17	−2.90	11.6000	15	5.6%	−0.34 [−1.04; 0.36]
Maat 2014	−17.24	15.8900	17	−12.85	17.5800	27	7.4%	−0.25 [−0.86; 0.36]
McCue2006	−12.90	11.8240	53	−15.40	11.8240	57	19.6%	0.21 [−0.17; 0.59]
**Total (95% CI)**			**87**			**99**	**32.6%**	**0.01 [−0.28; 0.30]**
Heterogeneity: $Tau^2 = 0.0401$; $Chi^2 = 2.78$, df = 2 (P = 0.25); $I^2 = 28\%$								
**Total (95% CI)**			**351**			**248**	**100.0%**	**0.06 [−0.10; 0.23]**
Heterogeneity: $Tau^2 = 0$; $Chi^2 = 3.23$, df = 5 (P = 0.66); $I^2 = 0\%$								
Test for subgroup differences: $Chi^2 = 0.21$, df = 1 (P = 0.65)								

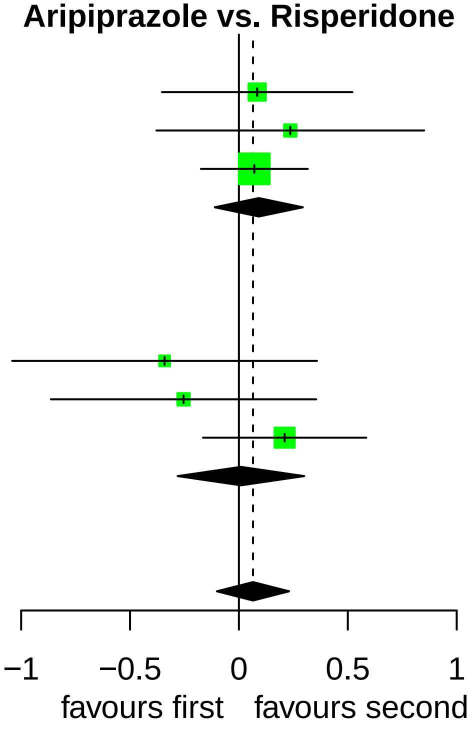

Study or Subgroup	first drug Mean	first drug SD	first drug Total	second drug Mean	second drug SD	second drug Total	Weight	SMD [95% CI]
**blinding = 0**								
Citrome 2015	−22.90	13.6000	64	−19.40	13.7900	33	40.2%	−0.25 [−0.68; 0.17]
**Total (95% CI)**			**64**			**33**	**40.2%**	**−0.25 [−0.68; 0.17]**
Heterogeneity: not applicable								
**blinding = 1**								
NCT00905307	−16.19	19.8009	90	−17.98	19.8009	50	59.8%	0.09 [−0.26; 0.44]
**Total (95% CI)**			**90**			**50**	**59.8%**	**0.09 [−0.26; 0.44]**
Heterogeneity: not applicable								
**Total (95% CI)**			**154**			**83**	**100.0%**	**−0.05 [−0.32; 0.22]**

Heterogeneity: $Tau^2 = 0.0205$; $Chi^2 = 1.53$, df = 1 (P = 0.22); $I^2 = 35\%$

Test for subgroup differences: $Chi^2 = 1.53$, df = 1 (P = 0.22)

Brexpiprazole vs. Aripiprazole

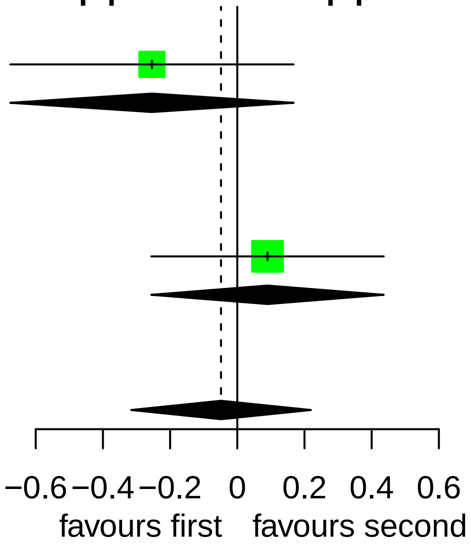

Study or Subgroup	first drug Mean	first drug SD	first drug Total	second drug Mean	second drug SD	second drug Total	Weight	SMD [95% CI]
**blinding = 1**								
Allan 1998	85.30	14.6000	10	83.40	26.2000	13	0.8%	0.08 [−0.74; 0.91]
Beasley 1996b	−12.90	15.7977	68	−13.93	15.7977	127	6.5%	0.06 [−0.23; 0.36]
Beasley 1997	−20.00	25.9000	79	−24.72	26.6400	168	7.9%	0.18 [−0.09; 0.45]
Bernardo 2001	62.70	20.7000	13	68.00	23.3000	14	1.0%	−0.23 [−0.99; 0.53]
Boulay 2007	56.10	12.9700	10	62.00	12.8400	14	0.8%	−0.44 [−1.26; 0.38]
Ishigooka 2001	−7.94	21.8500	78	−11.84	17.4200	80	5.8%	0.20 [−0.12; 0.51]
Lahti 2009	33.20	8.1000	12	33.80	6.9000	17	1.0%	−0.08 [−0.82; 0.66]
Rosenheck 2003w6	79.00	15.8000	115	79.90	15.5000	121	8.7%	−0.06 [−0.31; 0.20]
Tollefson 1997	−13.40	20.6000	636	−17.70	21.8000	1312	62.8%	0.20 [0.11; 0.30]
**Total (95% CI)**			**1021**			**1866**	**95.3%**	**0.15 [0.07; 0.23]**
Heterogeneity: $Tau^2 = 0.0044$; $Chi^2 = 7.44$, df = 8 (P = 0.49); $I^2 = 0\%$								
**blinding = 0**								
Avasthi 2001	12.57	13.3900	7	9.50	7.0600	16	0.7%	0.32 [−0.58; 1.21]
McCue2006	−16.40	11.8240	57	−14.90	11.8240	52	4.0%	−0.13 [−0.50; 0.25]
**Total (95% CI)**			**64**			**68**	**4.7%**	**−0.06 [−0.41; 0.29]**
Heterogeneity: $Tau^2 = 0$; $Chi^2 = 0.8$, df = 1 (P = 0.37); $I^2 = 0\%$								
**Total (95% CI)**			**1085**			**1934**	**100.0%**	**0.14 [0.07; 0.22]**

Heterogeneity: $Tau^2 = 0.0061$; $Chi^2 = 9.59$, df = 10 (P = 0.48); $I^2 = 0\%$

Test for subgroup differences: $Chi^2 = 1.35$, df = 1 (P = 0.24)

Haloperidol vs. Olanzapine

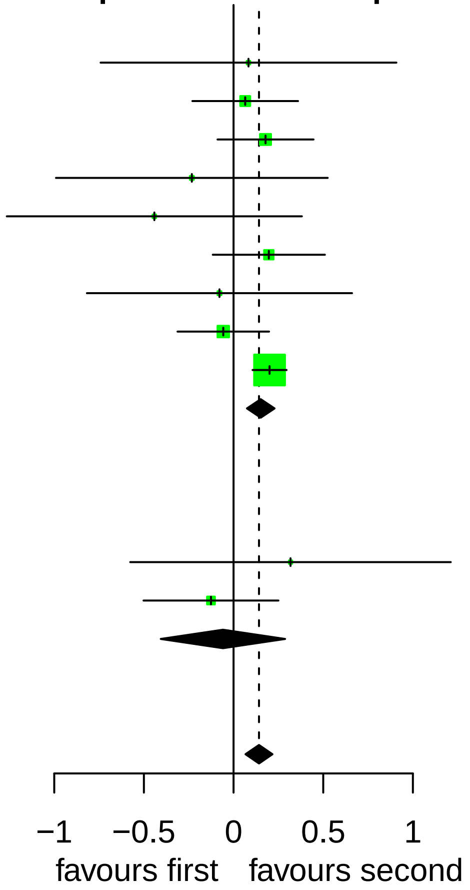

Study or Subgroup	first drug Mean	SD	Total	second drug Mean	SD	Total	Weight	SMD [95% CI]
**blinding = 1**								
Abdolahian 2008	86.10	17.0000	30	71.30	17.0000	35	3.5%	0.86 [0.35; 1.37]
Blin 1996	−26.60	27.1611	20	−44.70	27.1611	21	2.3%	0.65 [0.02; 1.28]
Borison 1992	−9.00	16.5414	53	−11.60	16.5414	53	6.3%	0.16 [−0.23; 0.54]
Ceskova 1993	28.58	6.4600	31	32.48	10.2400	31	3.6%	−0.45 [−0.95; 0.05]
Chouinard 1993	−9.30	25.5028	21	−25.70	25.5028	22	2.4%	0.63 [0.02; 1.25]
Claus 1992	74.30	20.1600	21	76.90	20.1600	21	2.5%	−0.13 [−0.73; 0.48]
Ghaleiha 2011	−37.87	29.3861	16	−43.62	29.3861	16	1.9%	0.19 [−0.50; 0.89]
Janicak 2001	−14.00	27.7400	32	−16.00	21.4200	30	3.7%	0.08 [−0.42; 0.58]
Liu 2000	−31.60	20.6000	19	−24.70	15.7000	19	2.2%	−0.37 [−1.01; 0.27]
Marder 1994	−4.10	20.3186	64	−16.10	20.3186	63	7.2%	0.59 [0.23; 0.94]
Min 1993	−21.90	9.3300	19	−17.10	11.6700	16	2.0%	−0.45 [−1.12; 0.23]
Murasaki 1993	20.30	12.2000	95	21.20	13.9000	97	11.4%	−0.07 [−0.35; 0.21]
Peuskens 1995	−15.00	21.8000	223	−18.25	23.5080	455	35.4%	0.14 [−0.02; 0.30]
See 1999	67.55	11.4500	10	59.95	9.6400	10	1.1%	0.69 [−0.22; 1.60]
Svestka 1990	20.70	11.3431	18	24.70	11.3431	18	2.1%	−0.34 [−1.00; 0.31]
Yen 2004	−24.80	18.5000	20	−29.80	15.7000	21	2.4%	0.29 [−0.33; 0.90]
**Total (95% CI)**			**692**			**928**	**89.8%**	**0.15 [0.05; 0.25]**
Heterogeneity: $Tau^2 = 0.0906$; $Chi^2 = 35.9$, df = 15 ($P < 0.01$); $I^2 = 58\%$								
**blinding = 0**								
Fakra 2008	57.27	12.9200	14	54.70	9.5000	11	1.4%	0.22 [−0.58; 1.01]
McCue2006	−16.40	11.8240	57	−15.40	11.8240	57	6.7%	−0.08 [−0.45; 0.28]
Tamrakar 2006	−43.17	12.6400	18	−52.11	12.2000	18	2.0%	0.70 [0.03; 1.38]
**Total (95% CI)**			**89**			**86**	**10.2%**	**0.11 [−0.19; 0.41]**
Heterogeneity: $Tau^2 = 0.0976$; $Chi^2 = 4.11$, df = 2 ($P = 0.13$); $I^2 = 51\%$								
**Total (95% CI)**			**781**			**1014**	**100.0%**	**0.14 [0.05; 0.24]**

Heterogeneity: $Tau^2 = 0.0804$; $Chi^2 = 40.05$, df = 18 ($P < 0.01$); $I^2 = 55\%$
Test for subgroup differences: $Chi^2 = 0.04$, df = 1 ($P = 0.83$)

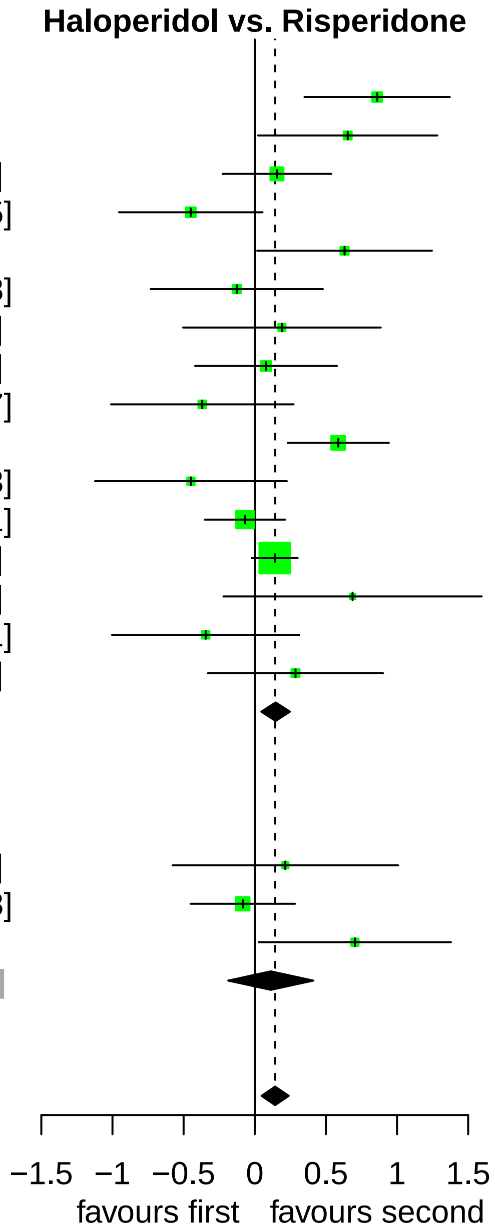

Study or Subgroup	first drug Mean	SD	Total	second drug Mean	SD	Total	Weight	SMD [95% CI]
blinding = 1								
Brook 1998	−5.50	6.0300	20	−6.04	6.0300	24	21.3%	0.09 [−0.51; 0.68]
Heikkila 1981	26.64	8.2555	19	22.98	8.2555	17	17.1%	0.43 [−0.23; 1.10]
Heikkilae 1992	10.30	7.9341	18	11.60	8.5329	20	18.5%	−0.15 [−0.79; 0.48]
**Total (95% CI)**			**57**			**61**	**56.9%**	**0.11 [−0.25; 0.48]**
Heterogeneity: $Tau^2 = 0$; $Chi^2 = 1.58$, df = 2 (P = 0.45); $I^2 = 0\%$								
blinding = 0								
Lamure 2003	21.70	13.6100	45	21.70	13.6100	43	43.1%	0.00 [−0.42; 0.42]
**Total (95% CI)**			**45**			**43**	**43.1%**	**0.00 [−0.42; 0.42]**
Heterogeneity: not applicable								
**Total (95% CI)**			**102**			**104**	**100.0%**	**0.06 [−0.21; 0.34]**

Heterogeneity: $Tau^2 = 0$; $Chi^2 = 1.74$, df = 3 (P = 0.63); $I^2 = 0\%$

Test for subgroup differences: $Chi^2 = 0.16$, df = 1 (P = 0.69)

Haloperidol vs. Zuclopenthixol

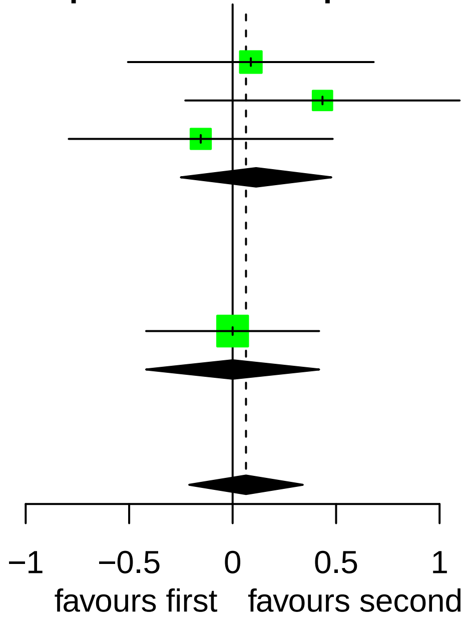

Study or Subgroup	first drug Mean	SD	Total	second drug Mean	SD	Total	Weight	SMD [95% CI]
**blinding = 1**								
Arvanitis 1997	−6.99	14.7899	104	−7.58	14.7899	50	13.8%	0.04 [−0.30; 0.38]
Copolov 2000	−18.70	24.0700	218	−22.10	24.1200	219	44.7%	0.14 [−0.05; 0.33]
Murasaki 2001	78.50	26.5000	97	78.30	25.7000	90	19.1%	0.01 [−0.28; 0.29]
**Total (95% CI)**			**419**			**359**	**77.6%**	**0.09 [−0.05; 0.23]**
Heterogeneity: $Tau^2 = 0$; $Chi^2 = 0.68$, df = 2 (P = 0.71); $I^2 = 0\%$								
**blinding = 0**								
Atmaca 2002	75.08	5.6500	18	74.43	5.4200	17	3.6%	0.11 [−0.55; 0.78]
McCue2006	−14.20	11.8240	50	−16.40	11.8240	57	10.9%	0.18 [−0.20; 0.57]
Taneli 2003	75.20	29.3000	45	72.20	27.4000	34	7.9%	0.10 [−0.34; 0.55]
**Total (95% CI)**			**113**			**108**	**22.4%**	**0.15 [−0.12; 0.41]**
Heterogeneity: $Tau^2 = 0$; $Chi^2 = 0.08$, df = 2 (P = 0.96); $I^2 = 0\%$								
**Total (95% CI)**			**532**			**467**	**100.0%**	**0.10 [−0.02; 0.23]**

Heterogeneity: $Tau^2 = 0$; $Chi^2 = 0.89$, df = 5 (P = 0.97); $I^2 = 0\%$
Test for subgroup differences: $Chi^2 = 0.13$, df = 1 (P = 0.72)

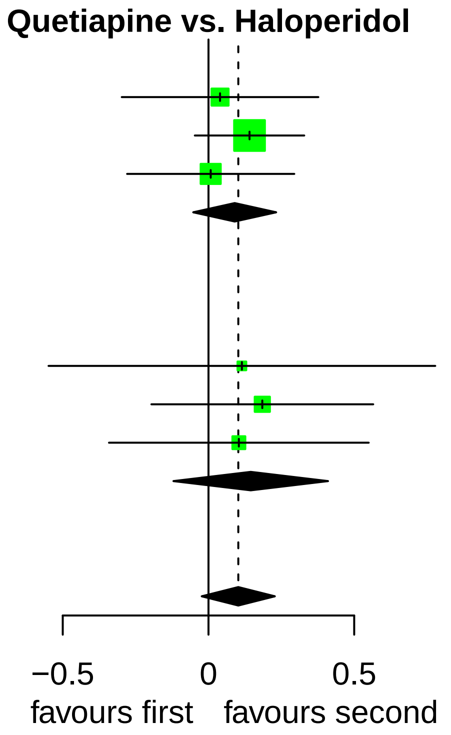

Study or Subgroup	first drug Mean	first drug SD	first drug Total	second drug Mean	second drug SD	second drug Total	Weight	SMD [95% CI]
**blinding = 1**								
Hatta 2009	−28.90	26.1940	20	−33.40	26.1940	17	3.6%	0.17 [−0.48; 0.82]
Lieberman 2005 12weeks	−3.76	13.4568	333	−6.38	13.4568	331	65.6%	0.19 [0.04; 0.35]
Mori 2004	72.90	12.7758	20	69.40	12.7758	20	3.9%	0.27 [−0.35; 0.89]
Riedel 2007	−21.50	23.3900	16	−17.88	20.7100	17	3.3%	−0.16 [−0.84; 0.52]
Sacchetti 2008	−38.15	20.4892	25	−35.83	20.4892	25	5.0%	−0.11 [−0.67; 0.44]
Svestka 2003a	−43.91	20.9400	22	−45.65	11.9600	20	4.2%	0.10 [−0.51; 0.70]
**Total (95% CI)**			**436**			**430**	**85.5%**	**0.16 [0.03; 0.29]**
Heterogeneity: $Tau^2 = 0$; $Chi^2 = 2.11$, df = 5 (P = 0.83); $I^2 = 0\%$								
**blinding = 0**								
McCue2006	−14.20	11.8240	50	−14.90	11.8240	52	10.1%	0.06 [−0.33; 0.45]
Yamashita 2004	−4.00	6.2791	28	−8.30	6.2791	20	4.4%	0.67 [0.08; 1.26]
**Total (95% CI)**			**78**			**72**	**14.5%**	**0.24 [−0.08; 0.57]**
Heterogeneity: $Tau^2 = 0.1240$; $Chi^2 = 2.91$, df = 1 (P = 0.09); $I^2 = 66\%$								
**Total (95% CI)**			**514**			**502**	**100.0%**	**0.17 [0.05; 0.30]**
Heterogeneity: $Tau^2 = 0$; $Chi^2 = 5.24$, df = 7 (P = 0.63); $I^2 = 0\%$								
Test for subgroup differences: $Chi^2 = 0.22$, df = 1 (P = 0.64)								

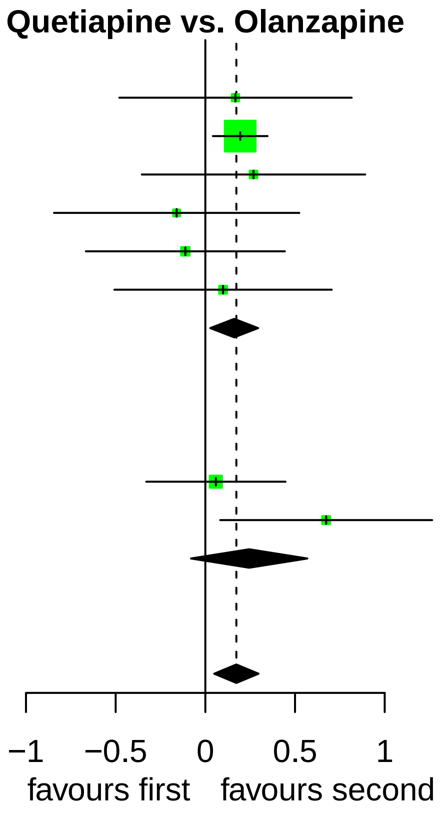

Study or Subgroup	first drug Mean	SD	Total	second drug Mean	SD	Total	Weight	SMD [95% CI]
**blinding = 1**								
Hatta 2009	−28.90	26.1940	20	−24.70	26.1940	20	2.2%	−0.16 [−0.78; 0.46]
Li 2012	−31.90	17.5000	60	−33.30	17.3000	59	6.5%	0.08 [−0.28; 0.44]
Lieberman 2005 12weeks	−3.76	13.4568	333	−3.10	13.4568	339	36.6%	−0.05 [−0.20; 0.10]
Moosavi 2015	−30.20	4.8700	45	−32.30	4.0700	45	4.8%	0.46 [0.04; 0.88]
Mori 2004	72.90	12.7758	20	71.50	12.7758	19	2.1%	0.11 [−0.52; 0.74]
Sacchetti 2008	−38.15	20.4892	25	−32.74	20.4892	25	2.7%	−0.26 [−0.82; 0.30]
Zhong 2006	−15.10	25.3600	328	−18.10	25.0000	318	35.1%	0.12 [−0.04; 0.27]
**Total (95% CI)**			**831**			**825**	**89.9%**	**0.05 [−0.05; 0.14]**
Heterogeneity: $Tau^2 = 0.0066$; $Chi^2 = 7.84$, df = 6 (P = 0.25); $I^2 = 23\%$								
**blinding = 0**								
Knegtering 2004	−5.40	12.3000	18	−8.40	11.2000	15	1.8%	0.25 [−0.44; 0.94]
McCue2006	−14.20	11.8240	50	−15.40	11.8240	57	5.8%	0.10 [−0.28; 0.48]
Yamashita 2004	−4.00	6.2791	28	−6.20	6.2791	20	2.5%	0.34 [−0.23; 0.92]
**Total (95% CI)**			**96**			**92**	**10.1%**	**0.19 [−0.10; 0.48]**
Heterogeneity: $Tau^2 = 0$; $Chi^2 = 0.51$, df = 2 (P = 0.77); $I^2 = 0\%$								
**Total (95% CI)**			**927**			**917**	**100.0%**	**0.06 [−0.03; 0.15]**

Heterogeneity: $Tau^2 = 0.0047$; $Chi^2 = 9.16$, df = 9 (P = 0.42); $I^2 = 2\%$

Test for subgroup differences: $Chi^2 = 0.81$, df = 1 (P = 0.37)

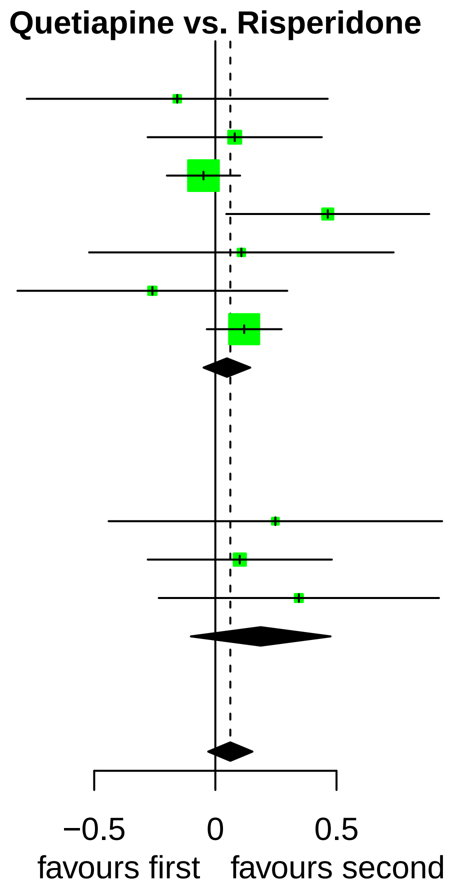

Study or Subgroup	first drug Mean	SD	Total	second drug Mean	SD	Total	Weight	SMD [95% CI]
**blinding = 1**								
Canive 2006	70.60	16.7528	4	57.80	16.7528	5	0.6%	0.68 [−0.70; 2.06]
Conley 2001	−12.80	15.9000	175	−12.90	16.1000	181	24.8%	0.01 [−0.20; 0.21]
Hatta 2009	−24.70	26.1940	20	−33.40	26.1940	17	2.5%	0.32 [−0.33; 0.98]
Hatta 2013	73.30	22.6100	20	71.09	27.3145	22	2.9%	0.09 [−0.52; 0.69]
Lieberman 2005 12weeks	−3.10	13.4568	339	−6.38	13.4568	331	46.3%	0.24 [0.09; 0.40]
Mori 2004	71.50	12.7758	19	69.40	12.7758	20	2.7%	0.16 [−0.47; 0.79]
Sacchetti 2008	−32.74	20.4892	25	−35.83	20.4892	25	3.5%	0.15 [−0.41; 0.70]
**Total (95% CI)**			**602**			**601**	**83.3%**	**0.17 [0.05; 0.28]**
Heterogeneity: Tau^2 = 0.0067; Chi^2 = 4.1, df = 6 (P = 0.66); I^2 = 0%								
**blinding = 0**								
Chen 2018	79.20	19.0194	25	67.11	19.0194	28	3.5%	0.63 [0.07; 1.18]
McCue2006	−15.40	11.8240	57	−14.90	11.8240	52	7.6%	−0.04 [−0.42; 0.33]
van Bruggen 2003	−15.00	12.7400	24	−15.10	23.7600	18	2.9%	0.01 [−0.61; 0.62]
Yamashita 2004	−6.20	6.2791	20	−8.30	6.2791	20	2.7%	0.33 [−0.30; 0.95]
**Total (95% CI)**			**126**			**118**	**16.7%**	**0.17 [−0.09; 0.42]**
Heterogeneity: Tau^2 = 0.0371; Chi^2 = 4.36, df = 3 (P = 0.23); I^2 = 31%								
**Total (95% CI)**			**728**			**719**	**100.0%**	**0.17 [0.06; 0.27]**

Heterogeneity: Tau^2 = 0.0060; Chi^2 = 8.46, df = 10 (P = 0.58); I^2 = 0%

Test for subgroup differences: Chi^2 = 0.00, df = 1 (P = 0.99)

Risperidone vs. Olanzapine

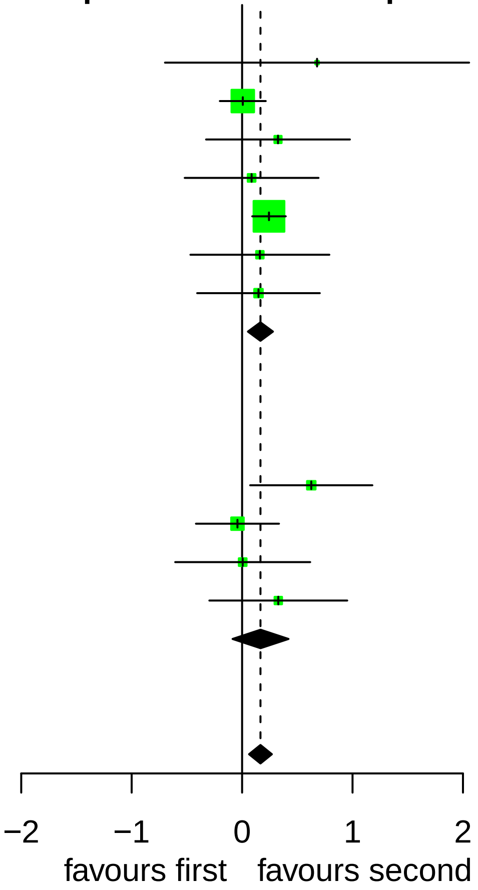

Study or Subgroup	first drug Mean	SD	Total	second drug Mean	SD	Total	Weight	SMD [95% CI]
**blinding = 0**								
McCue2006	−14.20	11.8240	50	−12.90	11.8240	53	29.5%	−0.11 [−0.50; 0.28]
**Total (95% CI)**			**50**			**53**	**29.5%**	**−0.11 [−0.50; 0.28]**
Heterogeneity: not applicable								
**blinding = 1**								
Zimbroff 2007	−21.60	18.7000	121	−24.60	19.1000	126	70.5%	0.16 [−0.09; 0.41]
**Total (95% CI)**			**121**			**126**	**70.5%**	**0.16 [−0.09; 0.41]**
Heterogeneity: not applicable								
**Total (95% CI)**			**171**			**179**	**100.0%**	**0.08 [−0.13; 0.29]**

Heterogeneity: $Tau^2 = 0.0081$; $Chi^2 = 1.30$, df = 1 (P = 0.26); $I^2 = 23\%$

Test for subgroup differences: $Chi^2 = 1.30$, df = 1 (P = 0.26)

Ziprasidone vs. Aripiprazole

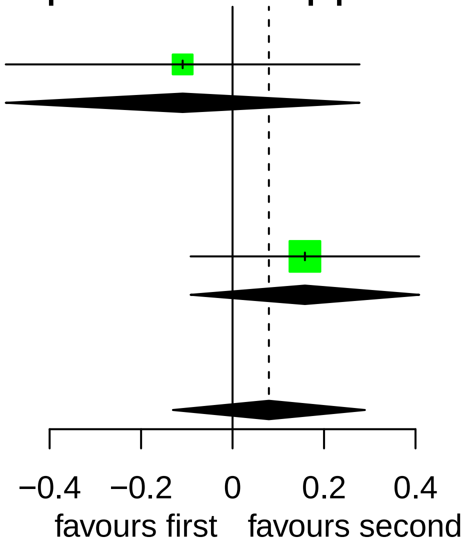

Study or Subgroup	first drug Mean	first drug SD	first drug Total	second drug Mean	second drug SD	second drug Total	Weight	SMD [95% CI]
**blinding = 1**								
Brook 2005	−14.99	19.2800	429	−15.79	15.9900	138	46.2%	0.04 [−0.15; 0.23]
Corripio 2005	61.10	16.7528	10	58.00	16.7528	10	2.2%	0.18 [−0.70; 1.06]
Goff 1998	−11.90	15.0000	20	−11.60	15.1000	17	4.1%	−0.02 [−0.67; 0.63]
Schennach 2018	66.23	23.0100	54	74.99	20.3800	58	12.1%	−0.40 [−0.78; −0.03]
Study 115 2000	−8.44	22.9324	158	−15.20	22.9324	82	23.7%	0.29 [0.03; 0.56]
**Total (95% CI)**			**671**			**305**	**88.3%**	**0.05 [−0.09; 0.19]**
Heterogeneity: $Tau^2 = 0.0498$; $Chi^2 = 8.88$, df = 4 (P = 0.06); $I^2 = 55\%$								
**blinding = 0**								
McCue2006	−14.20	11.8240	50	−16.40	11.8240	57	11.7%	0.18 [−0.20; 0.57]
**Total (95% CI)**			**50**			**57**	**11.7%**	**0.18 [−0.20; 0.57]**
Heterogeneity: not applicable								
**Total (95% CI)**			**721**			**362**	**100.0%**	**0.07 [−0.06; 0.20]**

Heterogeneity: $Tau^2 = 0.0357$; $Chi^2 = 9.31$, df = 5 (P = 0.10); $I^2 = 46\%$
Test for subgroup differences: $Chi^2 = 0.43$, df = 1 (P = 0.51)

Ziprasidone vs. Haloperidol

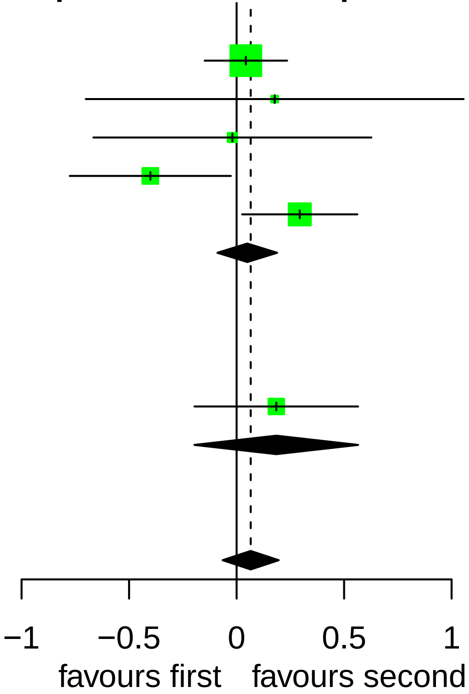

Study or Subgroup	first drug Mean	SD	Total	second drug Mean	SD	Total	Weight	SMD [95% CI]
**blinding = 1**								
Grootens 2009	−14.86	12.9900	34	−17.15	10.2500	33	7.1%	0.19 [−0.29; 0.67]
Lieberman 2005 12weeks	−2.74	13.4568	183	−6.38	13.4568	331	50.1%	0.27 [0.09; 0.45]
Simpson 2004	−11.45	13.5300	125	−11.95	13.5300	126	26.9%	0.04 [−0.21; 0.28]
Svestka 2005	60.29	18.3300	24	51.92	18.3300	24	5.0%	0.45 [−0.12; 1.02]
**Total (95% CI)**			**366**			**514**	**89.1%**	**0.20 [0.07; 0.34]**
Heterogeneity: $Tau^2 = 0.0053$; $Chi^2 = 2.97$, df = 3 (P = 0.40); $I^2 = 0\%$								
**blinding = 0**								
McCue2006	−14.20	11.8240	50	−14.90	11.8240	52	10.9%	0.06 [−0.33; 0.45]
**Total (95% CI)**			**50**			**52**	**10.9%**	**0.06 [−0.33; 0.45]**
Heterogeneity: not applicable								
**Total (95% CI)**			**416**			**566**	**100.0%**	**0.19 [0.06; 0.32]**

Heterogeneity: $Tau^2 = 0.0033$; $Chi^2 = 3.44$, df = 4 (P = 0.49); $I^2 = 0\%$
Test for subgroup differences: $Chi^2 = 0.48$, df = 1 (P = 0.49)

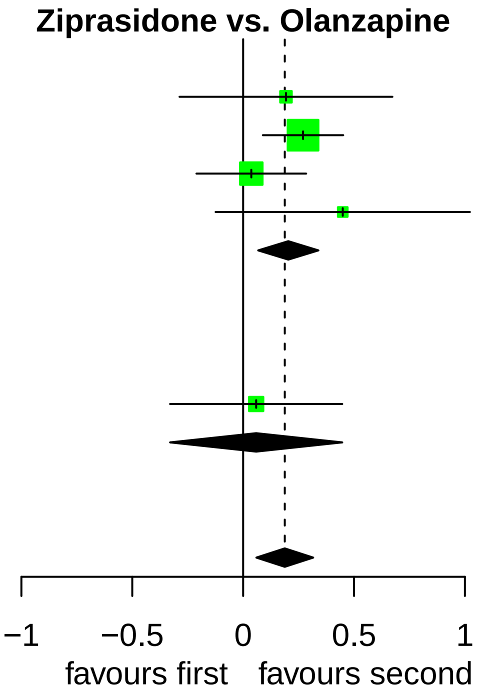

Study or Subgroup	first drug Mean	SD	Total	second drug Mean	SD	Total	Weight	SMD [95% CI]
**blinding = 1**								
Lieberman 2005 12weeks	−2.74	13.4568	183	−3.76	13.4568	333	82.5%	0.08 [−0.10; 0.26]
**Total (95% CI)**			**183**			**333**	**82.5%**	**0.08 [−0.10; 0.26]**
Heterogeneity: not applicable								
**blinding = 0**								
McCue2006	−14.20	11.8240	50	−14.20	11.8240	50	17.5%	0.00 [−0.39; 0.39]
**Total (95% CI)**			**50**			**50**	**17.5%**	**0.00 [−0.39; 0.39]**
Heterogeneity: not applicable								
**Total (95% CI)**			**233**			**383**	**100.0%**	**0.06 [−0.10; 0.23]**

Heterogeneity: $Tau^2 = 0$; $Chi^2 = 0.12$, df = 1 (P = 0.73); $I^2 = 0\%$

Test for subgroup differences: $Chi^2 = 0.12$, df = 1 (P = 0.73)

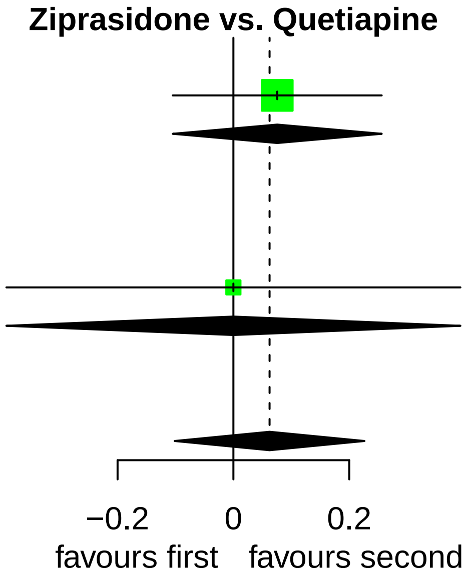

Study or Subgroup	first drug Mean	first drug SD	first drug Total	second drug Mean	second drug SD	second drug Total	Weight	SMD [95% CI]
**blinding = 1**								
Addington 2004	−25.80	20.4892	125	−27.30	20.4892	132	24.1%	0.07 [−0.17; 0.32]
Lieberman 2005 12weeks	−2.74	13.4568	183	−3.10	13.4568	339	44.7%	0.03 [−0.15; 0.21]
Zhang 2011	−35.60	14.9969	96	−37.10	14.7822	111	19.3%	0.10 [−0.17; 0.37]
**Total (95% CI)**			**404**			**582**	**88.1%**	**0.06 [−0.07; 0.18]**
Heterogeneity: Tau^2 = 0; Chi^2 = 0.22, df = 2 (P = 0.90); I^2 = 0%								
**blinding = 0**								
McCue2006	−14.20	11.8240	50	−15.40	11.8240	57	10.0%	0.10 [−0.28; 0.48]
Sonmez 2009	64.30	18.0600	10	62.00	23.1400	10	1.9%	0.11 [−0.77; 0.98]
**Total (95% CI)**			**60**			**67**	**11.9%**	**0.10 [−0.25; 0.45]**
Heterogeneity: Tau^2 = 0; Chi^2 = 0, df = 1 (P = 0.99); I^2 = 0%								
**Total (95% CI)**			**464**			**649**	**100.0%**	**0.06 [−0.06; 0.18]**
Heterogeneity: Tau^2 = 0; Chi^2 = 0.28, df = 4 (P = 0.99); I^2 = 0%								
Test for subgroup differences: Chi^2 = 0.06, df = 1 (P = 0.81)								

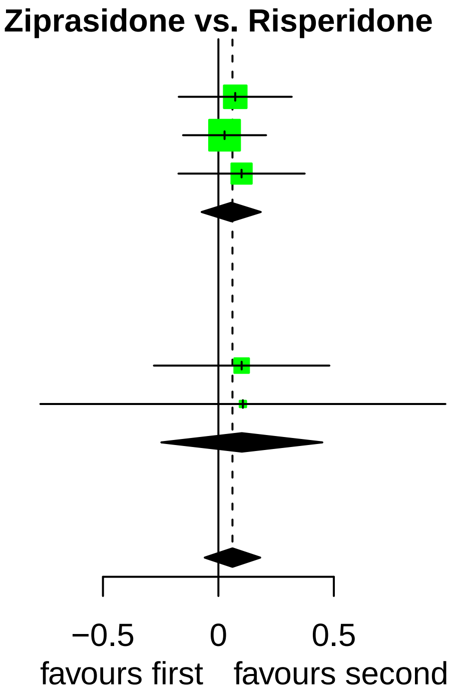

Figure 3b overall symptoms sensitivity analysis fixed-effects model difference between blinded and open trials, more recent drug listed first

Study	SMD	SE	Weight	SMD [95% CI]
Aripiprazole vs. Ziprasidone, (nbl=247, nop=103)	−0.2673	0.2349	3.9%	−0.27 [−0.73; 0.19]
Ziprasidone vs. Quetiapine, (nbl=516, nop=100)	0.0757	0.2202	4.4%	0.08 [−0.36; 0.51]
Quetiapine vs. Risperidone, (nbl=1656, nop=188)	−0.1394	0.1551	8.9%	−0.14 [−0.44; 0.16]
Quetiapine vs. Olanzapine, (nbl=866, nop=150)	−0.0834	0.1790	6.7%	−0.08 [−0.43; 0.27]
Aripiprazole vs. Quetiapine, (nbl=41, nop=103)	0.2840	0.3723	1.5%	0.28 [−0.45; 1.01]
Ziprasidone vs. Olanzapine, (nbl=880, nop=102)	0.1449	0.2099	4.8%	0.14 [−0.27; 0.56]
Aripiprazole vs. Olanzapine, (nbl=1544, nop=105)	0.0298	0.2021	5.2%	0.03 [−0.37; 0.43]
Aripiprazole vs. Haloperidol, (nbl=482, nop=207)	−0.2340	0.1723	7.2%	−0.23 [−0.57; 0.10]
Brexpiprazole vs. Aripiprazole, (nbl=140, nop=97)	0.3440	0.2782	2.8%	0.34 [−0.20; 0.89]
Aripiprazole vs. Risperidone, (nbl=413, nop=186)	0.0821	0.1806	6.5%	0.08 [−0.27; 0.44]
Olanzapine vs. Risperidone, (nbl=1203, nop=244)	0.0009	0.1416	10.7%	0.00 [−0.28; 0.28]
Ziprasidone vs. Haloperidol, (nbl=976, nop=107)	−0.1350	0.2067	5.0%	−0.13 [−0.54; 0.27]
Zuclopenthixol vs. Haloperidol, (nbl=118, nop=88)	−0.1134	0.2826	2.7%	−0.11 [−0.67; 0.44]
Quetiapine vs. Haloperidol, (nbl=778, nop=221)	−0.0550	0.1536	9.0%	−0.06 [−0.36; 0.25]
Olanzapine vs. Haloperidol, (nbl=2887, nop=132)	−0.2109	0.1812	6.5%	−0.21 [−0.57; 0.14]
Ziprasidone vs. Risperidone, (nbl=986, nop=127)	−0.0461	0.1895	5.9%	−0.05 [−0.42; 0.33]
Risperidone vs. Haloperidol, (nbl=1620, nop=175)	−0.0341	0.1609	8.2%	−0.03 [−0.35; 0.28]
**Total (95% CI)**			**100.0%**	**−0.05 [−0.14; 0.04]**

Heterogeneity: $Tau^2 = 0$; $Chi^2 = 8.20$, df = 16 (P = 0.94); $I^2 = 0\%$

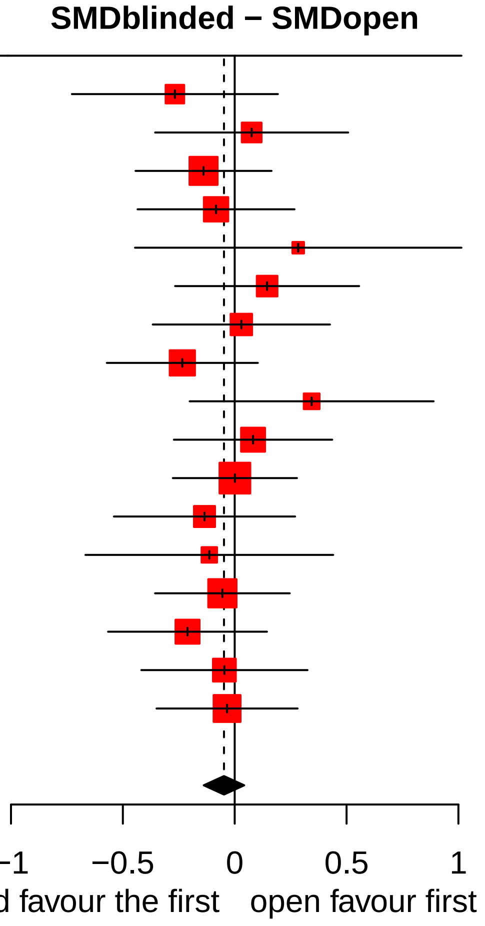

Figure 3c overall symptoms sensitivity analysis fixed effects model difference between blinded and open trials, less efficacious drug listed first

Study	SMD	SE	Weight	SMD [95% CI]
Ziprasidone vs. Aripiprazole, (nbl=247, nop=103)	0.2673	0.2349	3.9%	0.27 [−0.19; 0.73]
Ziprasidone vs. Quetiapine, (nbl=516, nop=100)	0.0757	0.2202	4.4%	0.08 [−0.36; 0.51]
Quetiapine vs. Risperidone, (nbl=1656, nop=188)	−0.1394	0.1551	8.9%	−0.14 [−0.44; 0.16]
Quetiapine vs. Olanzapine, (nbl=866, nop=150)	−0.0834	0.1790	6.7%	−0.08 [−0.43; 0.27]
Aripiprazole vs. Quetiapine, (nbl=41, nop=103)	0.2840	0.3723	1.5%	0.28 [−0.45; 1.01]
Ziprasidone vs. Olanzapine, (nbl=880, nop=102)	0.1449	0.2099	4.8%	0.14 [−0.27; 0.56]
Aripiprazole vs. Olanzapine, (nbl=1544, nop=105)	0.0298	0.2021	5.2%	0.03 [−0.37; 0.43]
Aripiprazole vs. Haloperidol, (nbl=482, nop=207)	−0.2340	0.1723	7.2%	−0.23 [−0.57; 0.10]
Brexpiprazole vs. Aripiprazole, (nbl=140, nop=97)	0.3440	0.2782	2.8%	0.34 [−0.20; 0.89]
Aripiprazole vs. Risperidone, (nbl=413, nop=186)	0.0821	0.1806	6.5%	0.08 [−0.27; 0.44]
Risperidone vs. Olanzapine, (nbl=1203, nop=244)	−0.0009	0.1416	10.7%	−0.00 [−0.28; 0.28]
Ziprasidone vs. Haloperidol, (nbl=976, nop=107)	−0.1350	0.2067	5.0%	−0.13 [−0.54; 0.27]
Haloperidol vs. Zuclopenthixol, (nbl=118, nop=88)	0.1134	0.2826	2.7%	0.11 [−0.44; 0.67]
Quetiapine vs. Haloperidol, (nbl=778, nop=221)	−0.0550	0.1536	9.0%	−0.06 [−0.36; 0.25]
Haloperidol vs. Olanzapine, (nbl=2887, nop=132)	0.2109	0.1812	6.5%	0.21 [−0.14; 0.57]
Ziprasidone vs. Risperidone, (nbl=986, nop=127)	−0.0461	0.1895	5.9%	−0.05 [−0.42; 0.33]
Haloperidol vs. Risperidone, (nbl=1620, nop=175)	0.0341	0.1609	8.2%	0.03 [−0.28; 0.35]
**Total (95% CI)**			**100.0%**	**0.01 [−0.08; 0.10]**

Heterogeneity: $Tau^2 = 0$; $Chi^2 = 9.21$, df = 16 (P = 0.90); $I^2 = 0\%$

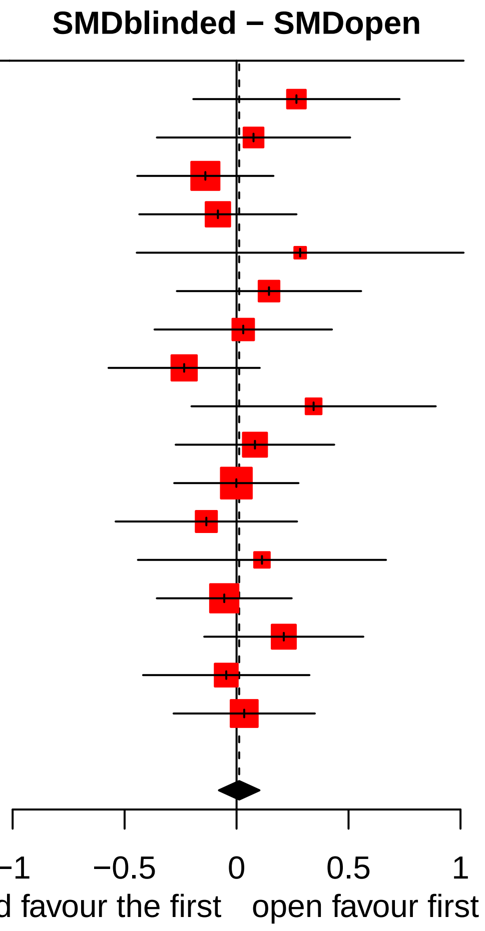

Figure 3d overall symptoms sensitivity analysis fixed-effects model sponsored versus non sponsored drugs in blinded and open trials

Study or Subgroup	SMD	SE	Weight	SMD [95% CI]
**blinding = 1**				
Addington 2004	−0.0732	0.1248	1.2%	−0.07 [−0.32; 0.17]
Arvanitis 1997	−0.0398	0.1721	0.6%	−0.04 [−0.38; 0.30]
Azorin 2006	0.1509	0.1529	0.8%	0.15 [−0.15; 0.45]
Beasley 1996b	0.0677	0.1503	0.8%	0.07 [−0.23; 0.36]
Beasley 1997	0.1787	0.1367	1.0%	0.18 [−0.09; 0.45]
Bernardo 2001	−0.2399	0.3865	0.1%	−0.24 [−1.00; 0.52]
Blin 1996	0.7467	0.2766	0.2%	0.75 [0.20; 1.29]
Boulay 2007	−0.4576	0.4193	0.1%	−0.46 [−1.28; 0.36]
Brook 1998	0.0896	0.3029	0.2%	0.09 [−0.50; 0.68]
Brook 2005	−0.0432	0.0979	2.0%	−0.04 [−0.23; 0.15]
Bueno 1979	−0.7661	0.3361	0.2%	−0.77 [−1.42; −0.11]
Canive 2006	0.7641	0.6946	0.0%	0.76 [−0.60; 2.13]
Chan 2007	−0.0847	0.2233	0.4%	−0.08 [−0.52; 0.35]
Chouinard 1993	0.5716	0.3112	0.2%	0.57 [−0.04; 1.18]
Clark 1970a	−0.2153	0.3727	0.1%	−0.22 [−0.95; 0.52]
Clark 1972	0.2689	0.3397	0.2%	0.27 [−0.40; 0.93]
Clark 1975	−0.3932	0.4042	0.1%	−0.39 [−1.19; 0.40]
Claus 1992	−0.1290	0.3089	0.2%	−0.13 [−0.73; 0.48]
Conley 2001	−0.0062	0.1060	1.7%	−0.01 [−0.21; 0.20]
Cooper 2000a	0.7297	0.2006	0.5%	0.73 [0.34; 1.12]
Copolov 2000	−0.1411	0.0958	2.1%	−0.14 [−0.33; 0.05]
Corripio 2005	−0.1850	0.4482	0.1%	−0.19 [−1.06; 0.69]
Costa e Silva 1989	0.3346	0.3357	0.2%	0.33 [−0.32; 0.99]
Cutler 2008	−0.0156	0.1024	1.8%	−0.02 [−0.22; 0.18]
Davidson 2007	−0.0855	0.1268	1.2%	−0.09 [−0.33; 0.16]
Dieterle 1991	−0.1620	0.3167	0.2%	−0.16 [−0.78; 0.46]
Durgam 2014	−0.3458	0.0985	2.0%	−0.35 [−0.54; −0.15]
Fleischhacker 1989	−0.5656	0.3225	0.2%	−0.57 [−1.20; 0.07]
Fleischhacker 2009_6weeks	−0.2391	0.0764	3.3%	−0.24 [−0.39; −0.09]
Freeman 1969	0.3107	0.3268	0.2%	0.31 [−0.33; 0.95]
Gattaz 2004	−0.1018	0.4006	0.1%	−0.10 [−0.89; 0.68]
Gelenberg 1979	1.0579	0.5524	0.1%	1.06 [−0.02; 2.14]
Goff 1998	0.0199	0.3299	0.2%	0.02 [−0.63; 0.67]
Grootens 2009	−0.1954	0.2449	0.3%	−0.20 [−0.68; 0.28]
Hale 2000	−0.0316	0.1108	1.5%	−0.03 [−0.25; 0.19]
Hera 041−021	−0.1670	0.1207	1.3%	−0.17 [−0.40; 0.07]
Hera 041−022	−0.2205	0.1539	0.8%	−0.22 [−0.52; 0.08]
Honigfeld 1984a	1.0456	0.4193	0.1%	1.05 [0.22; 1.87]
Honigfeld 1984c	0.7645	0.2331	0.3%	0.76 [0.31; 1.22]
Hoyberg 1993	0.1619	0.1993	0.5%	0.16 [−0.23; 0.55]
Huttunen 1995	0.1464	0.2023	0.5%	0.15 [−0.25; 0.54]
Hwang 2001	−0.2536	0.2400	0.3%	−0.25 [−0.72; 0.22]
Hwang 2003	−0.2380	0.2934	0.2%	−0.24 [−0.81; 0.34]
Ishigooka 2001	0.1977	0.1595	0.7%	0.20 [−0.11; 0.51]
Itoh 1977	0.2502	0.2145	0.4%	0.25 [−0.17; 0.67]
Janicak 2001	0.0804	0.2542	0.3%	0.08 [−0.42; 0.58]
Janssen CR012625	−0.0444	0.1709	0.6%	−0.04 [−0.38; 0.29]
Kane 2002	−0.0146	0.1205	1.3%	−0.01 [−0.25; 0.22]
Kane 2007b	−0.0180	0.1024	1.8%	−0.02 [−0.22; 0.18]
Kane 2009 8 weeks	0.2119	0.0843	2.7%	0.21 [0.05; 0.38]
Kane 2010a	0.0226	0.1166	1.4%	0.02 [−0.21; 0.25]
Kingstone 1970	0.1225	0.3127	0.2%	0.12 [−0.49; 0.74]
Kluge 2007	−0.2327	0.3664	0.1%	−0.23 [−0.95; 0.49]
Kramer 1978	0.2081	0.3927	0.1%	0.21 [−0.56; 0.98]
Li 2012	−0.0805	0.1834	0.6%	−0.08 [−0.44; 0.28]
Lin 2003	−0.7672	0.2743	0.3%	−0.77 [−1.30; −0.23]
Lundbeck NCT00864045	−0.2310	0.1029	1.8%	−0.23 [−0.43; −0.03]
Marder 1994	0.6217	0.1817	0.6%	0.62 [0.27; 0.98]
Marder 2007c	−0.0921	0.1185	1.4%	−0.09 [−0.32; 0.14]
Martin 2002	0.0764	0.1037	1.8%	0.08 [−0.13; 0.28]
McQuade 2004_6weeks	−0.1220	0.1431	0.9%	−0.12 [−0.40; 0.16]
Meyer−Lindenberg 1997	−0.0781	0.2830	0.2%	−0.08 [−0.63; 0.48]
Min 1993	−0.4589	0.3437	0.2%	−0.46 [−1.13; 0.21]
Möller 1997	0.1967	0.1462	0.9%	0.20 [−0.09; 0.48]
Moyano 1975	0.4905	0.3063	0.2%	0.49 [−0.11; 1.09]
Murasaki 2001	−0.0077	0.1464	0.9%	−0.01 [−0.29; 0.28]
NCT00350467	−0.1015	0.1184	1.4%	−0.10 [−0.33; 0.13]
NCT00882518	−0.1432	0.1140	1.5%	−0.14 [−0.37; 0.08]
NCT01104766	0.0228	0.0997	1.9%	0.02 [−0.17; 0.22]
NCT01617187	0.0061	0.1767	0.6%	0.01 [−0.34; 0.35]
NCT01810380	−0.2177	0.1158	1.4%	−0.22 [−0.44; 0.01]
Petit 1996	0.2263	0.1802	0.6%	0.23 [−0.13; 0.58]
Peuskens 1995	0.1414	0.0818	2.8%	0.14 [−0.02; 0.30]
Peuskens 1997	0.0503	0.1429	0.9%	0.05 [−0.23; 0.33]
Peuskens 1999	0.1735	0.1327	1.1%	0.17 [−0.09; 0.43]
Potkin 2003	−0.0710	0.1253	1.2%	−0.07 [−0.32; 0.17]
Potkin 2007c	0.1460	0.1876	0.5%	0.15 [−0.22; 0.51]
Puech 1998	0.3969	0.1575	0.8%	0.40 [0.09; 0.71]
Riedel 2007	−0.1642	0.3489	0.2%	−0.16 [−0.85; 0.52]
Rüther 1988	0.6034	0.4093	0.1%	0.60 [−0.20; 1.41]
Sacchetti 2008	0.1896	0.2454	0.3%	0.19 [−0.29; 0.67]
Schennach 2018	0.4039	0.1910	0.5%	0.40 [0.03; 0.78]
Selman 1976	−0.1307	0.2726	0.3%	−0.13 [−0.67; 0.40]
Shah 2011	0.0123	0.1394	1.0%	0.01 [−0.26; 0.29]
Simpson 2004	−0.0370	0.1263	1.2%	−0.04 [−0.28; 0.21]
Study 049	−0.2786	0.1466	0.9%	−0.28 [−0.57; 0.01]
Study 231	−0.1855	0.1120	1.5%	−0.19 [−0.41; 0.03]
Study 233	−0.1751	0.1128	1.5%	−0.18 [−0.40; 0.05]
Study 3000	−0.1906	0.1325	1.1%	−0.19 [−0.45; 0.07]
Study 3001	−0.0244	0.0951	2.1%	−0.02 [−0.21; 0.16]
Study 3002	−0.0879	0.0984	2.0%	−0.09 [−0.28; 0.11]
Study 3003	−0.0390	0.1046	1.7%	−0.04 [−0.24; 0.17]
Study 3004	−0.1948	0.1167	1.4%	−0.19 [−0.42; 0.03]
Study 3005	−0.2966	0.0977	2.0%	−0.30 [−0.49; −0.11]
Study 93202 2002	−0.3000	0.2476	0.3%	−0.30 [−0.79; 0.19]
Study 94202 2002	0.2199	0.1930	0.5%	0.22 [−0.16; 0.60]
Tollefson 1997	0.2008	0.0484	8.1%	0.20 [0.11; 0.30]
Tuason 1984	−0.1802	0.2430	0.3%	−0.18 [−0.66; 0.30]
van der Velde 1975	0.6060	0.2869	0.2%	0.61 [0.04; 1.17]
Wagner 2005	−0.5230	0.3390	0.2%	−0.52 [−1.19; 0.14]
Wetzel 1991	0.7551	0.3556	0.2%	0.76 [0.06; 1.45]
Wetzel 1998	0.4862	0.1802	0.6%	0.49 [0.13; 0.84]
Zborowski 1995	−0.2712	0.1342	1.1%	−0.27 [−0.53; −0.01]
Zhang 2011	−0.1008	0.1395	1.0%	−0.10 [−0.37; 0.17]
Zhong 2006	−0.1191	0.0788	3.1%	−0.12 [−0.27; 0.04]
Ziegler 1989	0.2113	0.3213	0.2%	0.21 [−0.42; 0.84]
Zimbroff 1997	−0.0807	0.1222	1.3%	−0.08 [−0.32; 0.16]
Zimbroff 2007	−0.1587	0.1275	1.2%	−0.16 [−0.41; 0.09]
**Total (95% CI)**			**97.9%**	**−0.01 [−0.04; 0.01]**
Heterogeneity: Tau^2 = 0.0232; Chi^2 = 238.18, df = 107 (P < 0.01); I^2 = 55%				
**blinding = 0**				
Avasthi 2001	0.3295	0.4558	0.1%	0.33 [−0.56; 1.22]
de Boer 2011	0.3501	0.3569	0.1%	0.35 [−0.35; 1.05]
de Oliviera 2009	−0.1585	0.2180	0.4%	−0.16 [−0.59; 0.27]
Dossenbach 2007	1.2281	0.2144	0.4%	1.23 [0.81; 1.65]
Knegtering 2004	−0.2539	0.3510	0.2%	−0.25 [−0.94; 0.43]
Loza 1999	0.7428	0.3394	0.2%	0.74 [0.08; 1.41]
Maat 2014	0.2589	0.3108	0.2%	0.26 [−0.35; 0.87]
Taneli 2003	−0.1053	0.2274	0.4%	−0.11 [−0.55; 0.34]
van Bruggen 2003	0.0055	0.3118	0.2%	0.01 [−0.61; 0.62]
**Total (95% CI)**			**2.1%**	**0.29 [0.11; 0.48]**
Heterogeneity: Tau^2 = 0.1916; Chi^2 = 31.45, df = 8 (P < 0.01); I^2 = 75%				
**Total (95% CI)**			**100.0%**	**−0.01 [−0.03; 0.02]**

Heterogeneity: Tau^2 = 0.0330; Chi^2 = 279.86, df = 116 (P < 0.01); I^2 = 59%

Test for subgroup differences: Chi^2 = 10.22, df = 1 (P < 0.01)

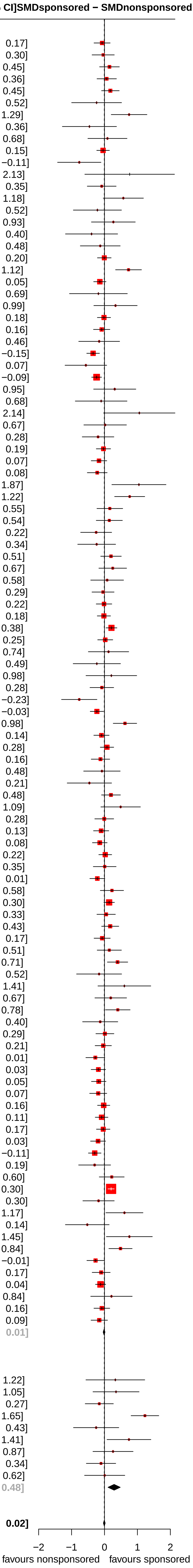

eFigure 4

Sensitivity analyses of the primary outcome

Single-blind studies excluded

eFigure 4a overall symptoms sensitivity analysis single-blind studies excluded individual comparisons

Study or Subgroup	first drug Mean	SD	Total	second drug Mean	SD	Total	Weight	SMD [95% CI]
**blinding = 0**								
de Oliviera 2009	69.70	22.9000	66	66.40	15.4000	31	14.1%	0.16 [−0.27; 0.58]
McCue2006	−12.90	11.8240	53	−16.40	11.8240	57	17.8%	0.29 [−0.08; 0.67]
**Total (95% CI)**			**119**			**88**	**31.9%**	**0.23 [−0.05; 0.52]**
Heterogeneity: Tau^2 = 0; Chi^2 = 0.22, df = 1 (P = 0.64); I^2 = 0%								
**blinding = 1**								
Kane 2002	−13.44	24.4583	204	−13.80	24.4583	104	39.3%	0.01 [−0.22; 0.25]
Study 93202 2002	−11.10	15.7265	33	−15.80	15.7265	33	11.1%	0.30 [−0.19; 0.78]
Study 94202 2002	−15.90	25.2612	54	−10.40	25.2612	54	17.6%	−0.22 [−0.59; 0.16]
**Total (95% CI)**			**291**			**191**	**68.1%**	**0.00 [−0.18; 0.19]**
Heterogeneity: Tau^2 = < 0.0001; Chi^2 = 2.69, df = 2 (P = 0.26); I^2 = 26%								
**Total (95% CI)**			**410**			**279**	**100.0%**	**0.08 [−0.09; 0.24]**
Heterogeneity: Tau^2 = 0.0040; Chi^2 = 4.76, df = 4 (P = 0.31); I^2 = 16%								
Test for subgroup differences: Chi^2 = 1.85, df = 1 (P = 0.17)								

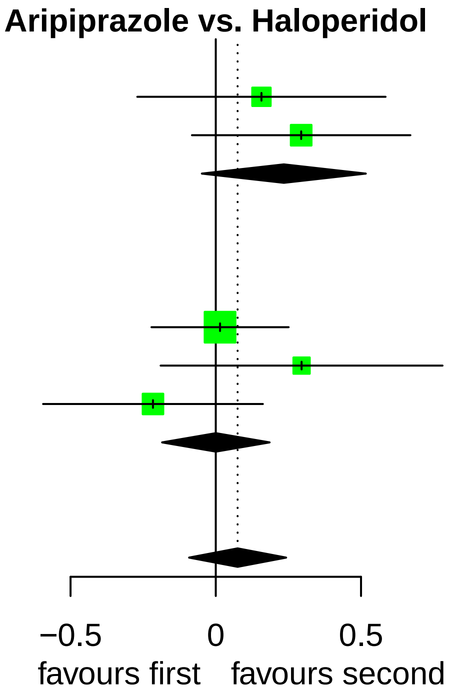

Study or Subgroup	first drug Mean	first drug SD	first drug Total	second drug Mean	second drug SD	second drug Total	Weight	SMD [95% CI]
**blinding = 1**								
Fleischhacker 2009_6weeks	−24.60	20.4892	347	−29.50	20.4892	344	42.9%	0.24 [0.09; 0.39]
Jindal 2013	−45.31	11.9400	26	−40.93	5.4000	27	3.2%	−0.47 [−1.02; 0.08]
Kane 2009 8 weeks	−22.20	22.3000	285	−26.80	21.1000	281	35.2%	0.21 [0.05; 0.38]
McQuade 2004_6weeks	−28.21	20.4892	95	−30.71	20.4892	101	12.2%	0.12 [−0.16; 0.40]
**Total (95% CI)**			**753**			**753**	**93.5%**	**0.19 [0.09; 0.29]**
Heterogeneity: Tau^2 = < 0.0001; Chi^2 = 6.29, df = 3 (P = 0.10); I^2 = 52%								
**blinding = 0**								
McCue2006	−12.90	11.8240	53	−14.90	11.8240	52	6.5%	0.17 [−0.22; 0.55]
**Total (95% CI)**			**53**			**52**	**6.5%**	**0.17 [−0.22; 0.55]**
Heterogeneity: not applicable								
**Total (95% CI)**			**806**			**805**	**100.0%**	**0.19 [0.09; 0.29]**

Heterogeneity: Tau^2 < 0.0001; Chi^2 = 6.30, df = 4 (P = 0.18); I^2 = 36%

Test for subgroup differences: Chi^2 = 0.01, df = 1 (P = 0.92)

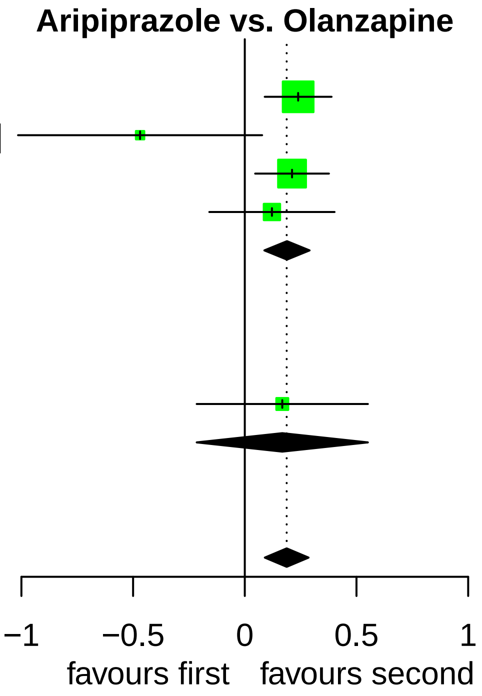

Study or Subgroup	first drug Mean	SD	Total	second drug Mean	SD	Total	Weight	SMD [95% CI]
**blinding = 1**								
Chan 2007	−19.60	18.1000	49	−21.10	17.1000	34	15.5%	0.08 [−0.35; 0.52]
Potkin 2003	−14.20	21.1274	194	−15.70	21.1274	95	49.3%	0.07 [−0.17; 0.32]
**Total (95% CI)**			**243**			**129**	**64.8%**	**0.07 [−0.14; 0.29]**
Heterogeneity: $Tau^2 = 0$; $Chi^2 = 0$, df = 1 (P = 0.96); $I^2 = 0\%$								
**blinding = 0**								
de Boer 2011	−8.10	17.2000	17	−2.90	11.6000	15	6.1%	−0.34 [−1.04; 0.36]
Maat 2014	−17.24	15.8900	17	−12.85	17.5800	27	8.0%	−0.25 [−0.86; 0.36]
McCue2006	−12.90	11.8240	53	−15.40	11.8240	57	21.1%	0.21 [−0.17; 0.59]
**Total (95% CI)**			**87**			**99**	**35.2%**	**−0.04 [−0.43; 0.34]**
Heterogeneity: $Tau^2 = 0.0401$; $Chi^2 = 2.78$, df = 2 (P = 0.25); $I^2 = 28\%$								
**Total (95% CI)**			**330**			**228**	**100.0%**	**0.05 [−0.12; 0.22]**
Heterogeneity: $Tau^2 = 0$; $Chi^2 = 2.91$, df = 4 (P = 0.57); $I^2 = 0\%$								
Test for subgroup differences: $Chi^2 = 0.28$, df = 1 (P = 0.60)								

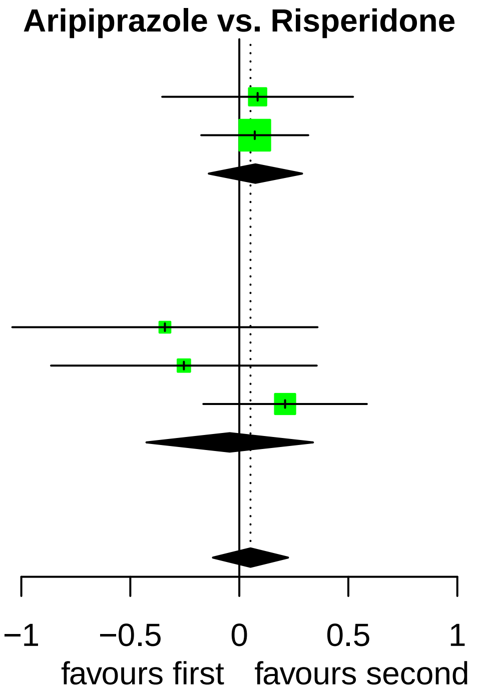

Study or Subgroup	first drug Mean	SD	Total	second drug Mean	SD	Total	Weight	SMD [95% CI]
**blinding = 0**								
Citrome 2015	−22.90	13.6000	64	−19.40	13.7900	33	43.6%	−0.25 [−0.68; 0.17]
**Total (95% CI)**			**64**			**33**	**43.6%**	**−0.25 [−0.68; 0.17]**
Heterogeneity: not applicable								
**blinding = 1**								
NCT00905307	−16.19	19.8009	90	−17.98	19.8009	50	56.4%	0.09 [−0.26; 0.44]
**Total (95% CI)**			**90**			**50**	**56.4%**	**0.09 [−0.26; 0.44]**
Heterogeneity: not applicable								
**Total (95% CI)**			**154**			**83**	**100.0%**	**−0.06 [−0.39; 0.27]**

Heterogeneity: $Tau^2 = 0.0205$; $Chi^2 = 1.53$, df = 1 (P = 0.22); $I^2 = 35\%$
Test for subgroup differences: $Chi^2 = 1.53$, df = 1 (P = 0.22)

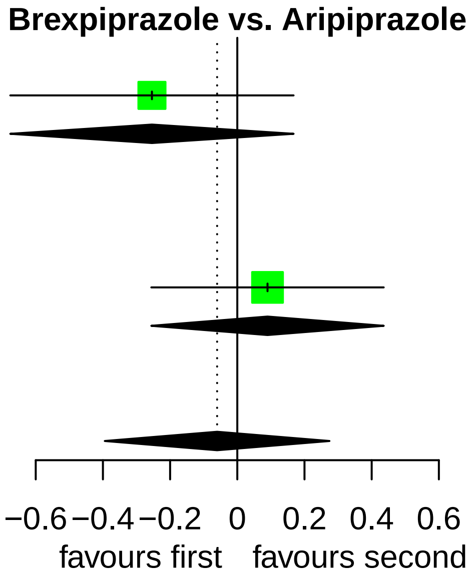

Study or Subgroup	first drug Mean	first drug SD	first drug Total	second drug Mean	second drug SD	second drug Total	Weight	SMD [95% CI]
**blinding = 1**								
Allan 1998	85.30	14.6000	10	83.40	26.2000	13	1.7%	0.08 [−0.74; 0.91]
Beasley 1996b	−12.90	15.7977	68	−13.93	15.7977	127	10.9%	0.06 [−0.23; 0.36]
Beasley 1997	−20.00	25.9000	79	−24.72	26.6400	168	12.6%	0.18 [−0.09; 0.45]
Bernardo 2001	62.70	20.7000	13	68.00	23.3000	14	2.0%	−0.23 [−0.99; 0.53]
Boulay 2007	56.10	12.9700	10	62.00	12.8400	14	1.7%	−0.44 [−1.26; 0.38]
Ishigooka 2001	−7.94	21.8500	78	−11.84	17.4200	80	9.9%	0.20 [−0.12; 0.51]
Lahti 2009	33.20	8.1000	12	33.80	6.9000	17	2.1%	−0.08 [−0.82; 0.66]
Rosenheck 2003w6	79.00	15.8000	115	79.90	15.5000	121	13.5%	−0.06 [−0.31; 0.20]
Tollefson 1997	−13.40	20.6000	636	−17.70	21.8000	1312	36.9%	0.20 [0.11; 0.30]
**Total (95% CI)**			**1021**			**1866**	**91.3%**	**0.12 [0.01; 0.23]**
Heterogeneity: $Tau^2 = 0.0044$; $Chi^2 = 7.44$, df = 8 (P = 0.49); $I^2 = 0\%$								
**blinding = 0**								
Avasthi 2001	12.57	13.3900	7	9.50	7.0600	16	1.5%	0.32 [−0.58; 1.21]
McCue2006	−16.40	11.8240	57	−14.90	11.8240	52	7.3%	−0.13 [−0.50; 0.25]
**Total (95% CI)**			**64**			**68**	**8.7%**	**−0.06 [−0.41; 0.29]**
Heterogeneity: $Tau^2 = 0$; $Chi^2 = 0.8$, df = 1 (P = 0.37); $I^2 = 0\%$								
**Total (95% CI)**			**1085**			**1934**	**100.0%**	**0.10 [−0.01; 0.21]**

Heterogeneity: $Tau^2 = 0.0061$; $Chi^2 = 9.59$, df = 10 (P = 0.48); $I^2 = 0\%$
Test for subgroup differences: $Chi^2 = 0.93$, df = 1 (P = 0.34)

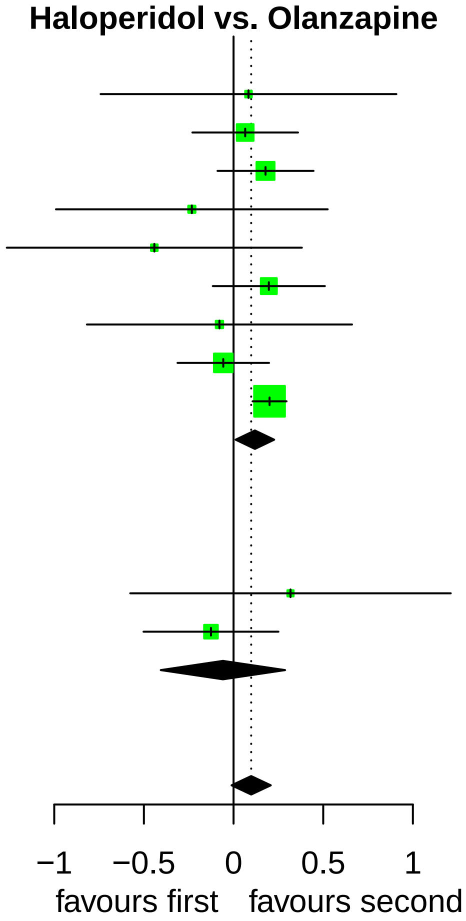

Study or Subgroup	first drug Mean	first drug SD	first drug Total	second drug Mean	second drug SD	second drug Total	Weight	SMD [95% CI]
**blinding = 1**								
Abdolahian 2008	86.10	17.0000	30	71.30	17.0000	35	5.8%	0.86 [0.35; 1.37]
Blin 1996	−26.60	27.1611	20	−44.70	27.1611	21	4.7%	0.65 [0.02; 1.28]
Borison 1992	−9.00	16.5414	53	−11.60	16.5414	53	7.1%	0.16 [−0.23; 0.54]
Ceskova 1993	28.58	6.4600	31	32.48	10.2400	31	5.8%	−0.45 [−0.95; 0.05]
Chouinard 1993	−9.30	25.5028	21	−25.70	25.5028	22	4.8%	0.63 [0.02; 1.25]
Claus 1992	74.30	20.1600	21	76.90	20.1600	21	4.9%	−0.13 [−0.73; 0.48]
Ghaleiha 2011	−37.87	29.3861	16	−43.62	29.3861	16	4.2%	0.19 [−0.50; 0.89]
Janicak 2001	−14.00	27.7400	32	−16.00	21.4200	30	5.9%	0.08 [−0.42; 0.58]
Liu 2000	−31.60	20.6000	19	−24.70	15.7000	19	4.6%	−0.37 [−1.01; 0.27]
Marder 1994	−4.10	20.3186	64	−16.10	20.3186	63	7.4%	0.59 [0.23; 0.94]
Min 1993	−21.90	9.3300	19	−17.10	11.6700	16	4.4%	−0.45 [−1.12; 0.23]
Murasaki 1993	20.30	12.2000	95	21.20	13.9000	97	8.2%	−0.07 [−0.35; 0.21]
Peuskens 1995	−15.00	21.8000	223	−18.25	23.5080	455	9.5%	0.14 [−0.02; 0.30]
See 1999	67.55	11.4500	10	59.95	9.6400	10	3.0%	0.69 [−0.22; 1.60]
Svestka 1990	20.70	11.3431	18	24.70	11.3431	18	4.5%	−0.34 [−1.00; 0.31]
**Total (95% CI)**			**672**			**907**	**84.8%**	**0.14 [−0.07; 0.35]**
Heterogeneity: $Tau^2 = 0.1008$; $Chi^2 = 35.7$, df = 14 ($P < 0.01$); $I^2 = 61\%$								
**blinding = 0**								
Fakra 2008	57.27	12.9200	14	54.70	9.5000	11	3.6%	0.22 [−0.58; 1.01]
McCue2006	−16.40	11.8240	57	−15.40	11.8240	57	7.3%	−0.08 [−0.45; 0.28]
Tamrakar 2006	−43.17	12.6400	18	−52.11	12.2000	18	4.3%	0.70 [0.03; 1.38]
**Total (95% CI)**			**89**			**86**	**15.2%**	**0.22 [−0.27; 0.71]**
Heterogeneity: $Tau^2 = 0.0976$; $Chi^2 = 4.11$, df = 2 ($P = 0.13$); $I^2 = 51\%$								
**Total (95% CI)**			**761**			**993**	**100.0%**	**0.15 [−0.03; 0.34]**
Heterogeneity: $Tau^2 = 0.0882$; $Chi^2 = 39.84$, df = 17 ($P < 0.01$); $I^2 = 57\%$								
Test for subgroup differences: $Chi^2 = 0.07$, df = 1 ($P = 0.79$)								

Haloperidol vs. Risperidone

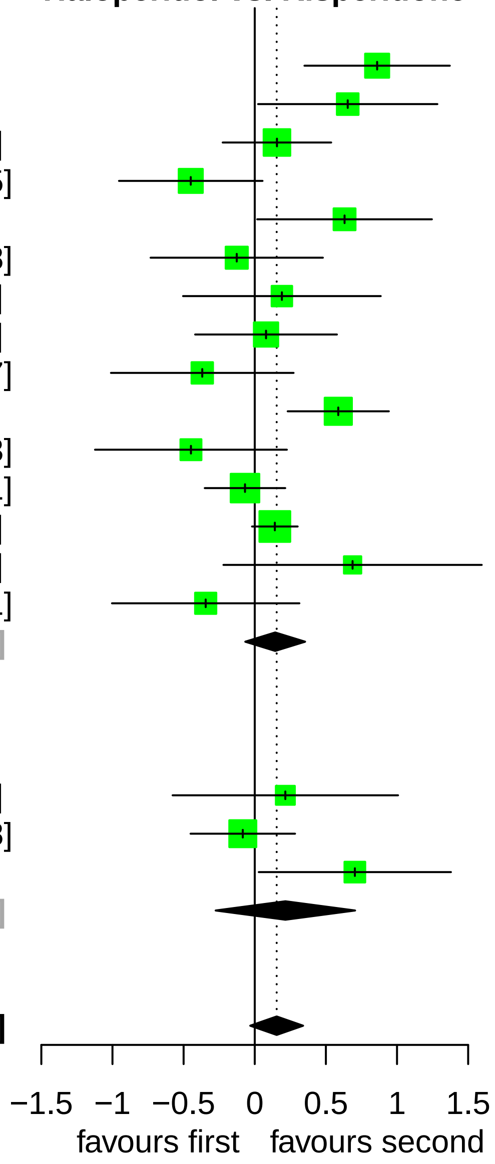

Study or Subgroup	first drug Mean	SD	Total	second drug Mean	SD	Total	Weight	SMD [95% CI]
**blinding = 1**								
Brook 1998	−5.50	6.0300	20	−6.04	6.0300	24	21.3%	0.09 [−0.51; 0.68]
Heikkila 1981	26.64	8.2555	19	22.98	8.2555	17	17.1%	0.43 [−0.23; 1.10]
Heikkilae 1992	10.30	7.9341	18	11.60	8.5329	20	18.5%	−0.15 [−0.79; 0.48]
**Total (95% CI)**			**57**			**61**	**56.9%**	**0.11 [−0.25; 0.48]**
Heterogeneity: $Tau^2 = 0$; $Chi^2 = 1.58$, df = 2 (P = 0.45); $I^2 = 0\%$								
**blinding = 0**								
Lamure 2003	21.70	13.6100	45	21.70	13.6100	43	43.1%	0.00 [−0.42; 0.42]
**Total (95% CI)**			**45**			**43**	**43.1%**	**0.00 [−0.42; 0.42]**
Heterogeneity: not applicable								
**Total (95% CI)**			**102**			**104**	**100.0%**	**0.06 [−0.21; 0.34]**

Heterogeneity: $Tau^2 = 0$; $Chi^2 = 1.74$, df = 3 (P = 0.63); $I^2 = 0\%$
Test for subgroup differences: $Chi^2 = 0.16$, df = 1 (P = 0.69)

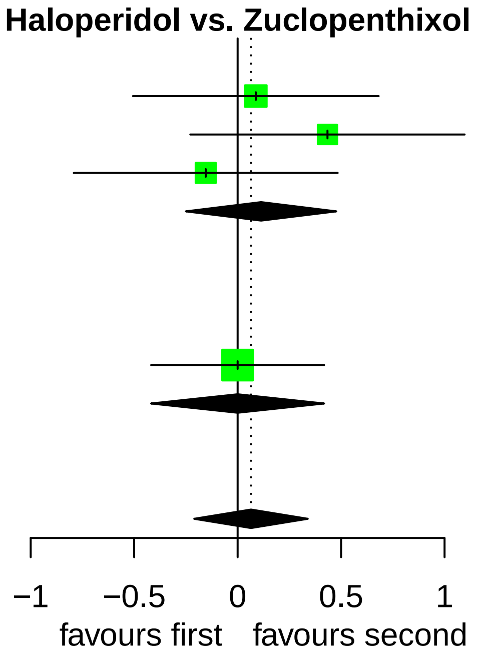

Study or Subgroup	first drug Mean	SD	Total	second drug Mean	SD	Total	Weight	SMD [95% CI]
**blinding = 1**								
Arvanitis 1997	−6.99	14.7899	104	−7.58	14.7899	50	13.8%	0.04 [−0.30; 0.38]
Copolov 2000	−18.70	24.0700	218	−22.10	24.1200	219	44.7%	0.14 [−0.05; 0.33]
Murasaki 2001	78.50	26.5000	97	78.30	25.7000	90	19.1%	0.01 [−0.28; 0.29]
**Total (95% CI)**			**419**			**359**	**77.6%**	**0.09 [−0.05; 0.23]**
Heterogeneity: $Tau^2 = 0$; $Chi^2 = 0.68$, df = 2 (P = 0.71); $I^2 = 0\%$								
**blinding = 0**								
Atmaca 2002	75.08	5.6500	18	74.43	5.4200	17	3.6%	0.11 [−0.55; 0.78]
McCue2006	−14.20	11.8240	50	−16.40	11.8240	57	10.9%	0.18 [−0.20; 0.57]
Taneli 2003	75.20	29.3000	45	72.20	27.4000	34	7.9%	0.10 [−0.34; 0.55]
**Total (95% CI)**			**113**			**108**	**22.4%**	**0.15 [−0.12; 0.41]**
Heterogeneity: $Tau^2 = 0$; $Chi^2 = 0.08$, df = 2 (P = 0.96); $I^2 = 0\%$								
**Total (95% CI)**			**532**			**467**	**100.0%**	**0.10 [−0.02; 0.23]**

Heterogeneity: $Tau^2 = 0$; $Chi^2 = 0.89$, df = 5 (P = 0.97); $I^2 = 0\%$

Test for subgroup differences: $Chi^2 = 0.13$, df = 1 (P = 0.72)

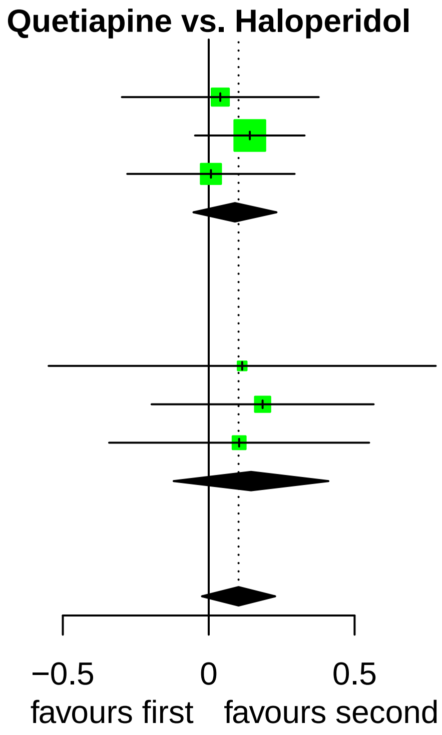

Study or Subgroup	first drug Mean	SD	Total	second drug Mean	SD	Total	Weight	SMD [95% CI]
**blinding = 1**								
Lieberman 2005 12weeks	−3.76	13.4568	333	−6.38	13.4568	331	71.7%	0.19 [0.04; 0.35]
Mori 2004	72.90	12.7758	20	69.40	12.7758	20	4.3%	0.27 [−0.35; 0.89]
Riedel 2007	−21.50	23.3900	16	−17.88	20.7100	17	3.6%	−0.16 [−0.84; 0.52]
Svestka 2003a	−43.91	20.9400	22	−45.65	11.9600	20	4.5%	0.10 [−0.51; 0.70]
**Total (95% CI)**			**391**			**388**	**84.2%**	**0.18 [0.04; 0.32]**
Heterogeneity: $Tau^2 = 0$; $Chi^2 = 1.13$, df = 3 (P = 0.77); $I^2 = 0\%$								
**blinding = 0**								
McCue2006	−14.20	11.8240	50	−14.90	11.8240	52	11.1%	0.06 [−0.33; 0.45]
Yamashita 2004	−4.00	6.2791	28	−8.30	6.2791	20	4.8%	0.67 [0.08; 1.26]
**Total (95% CI)**			**78**			**72**	**15.8%**	**0.32 [−0.27; 0.92]**
Heterogeneity: $Tau^2 = 0.1240$; $Chi^2 = 2.91$, df = 1 (P = 0.09); $I^2 = 66\%$								
**Total (95% CI)**			**469**			**460**	**100.0%**	**0.19 [0.06; 0.32]**

Heterogeneity: $Tau^2 < 0.0001$; $Chi^2 = 4.17$, df = 5 (P = 0.53); $I^2 = 0\%$

Test for subgroup differences: $Chi^2 = 0.22$, df = 1 (P = 0.64)

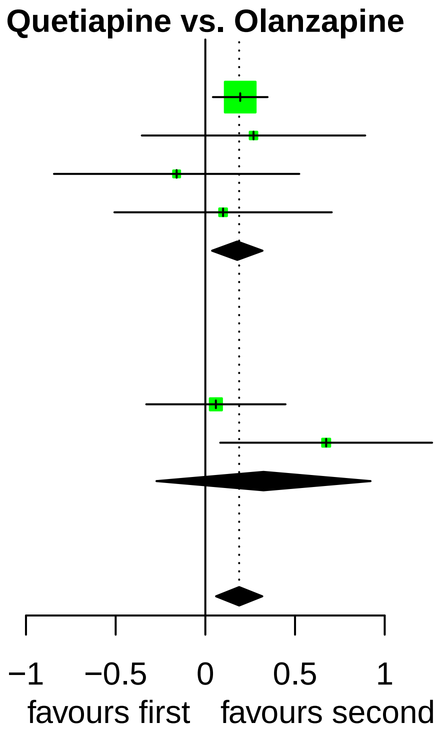

Study or Subgroup	first drug Mean	first drug SD	first drug Total	second drug Mean	second drug SD	second drug Total	Weight	SMD [95% CI]
**blinding = 0**								
Knegtering 2004	−5.40	12.3000	18	−8.40	11.2000	15	4.0%	0.25 [−0.44; 0.94]
McCue2006	−14.20	11.8240	50	−15.40	11.8240	57	11.2%	0.10 [−0.28; 0.48]
Yamashita 2004	−4.00	6.2791	28	−6.20	6.2791	20	5.5%	0.34 [−0.23; 0.92]
**Total (95% CI)**			**96**			**92**	**20.8%**	**0.19 [−0.10; 0.48]**
Heterogeneity: $Tau^2 = 0$; $Chi^2 = 0.51$, df = 2 (P = 0.77); $I^2 = 0\%$								
**blinding = 1**								
Lieberman 2005 12weeks	−3.76	13.4568	333	−3.10	13.4568	339	32.6%	−0.05 [−0.20; 0.10]
Moosavi 2015	−30.20	4.8700	45	−32.30	4.0700	45	9.6%	0.46 [0.04; 0.88]
Mori 2004	72.90	12.7758	20	71.50	12.7758	19	4.8%	0.11 [−0.52; 0.74]
Zhong 2006	−15.10	25.3600	328	−18.10	25.0000	318	32.2%	0.12 [−0.04; 0.27]
**Total (95% CI)**			**726**			**721**	**79.2%**	**0.10 [−0.09; 0.30]**
Heterogeneity: $Tau^2 = 0.0186$; $Chi^2 = 6.15$, df = 3 (P = 0.10); $I^2 = 51\%$								
**Total (95% CI)**			**822**			**813**	**100.0%**	**0.11 [−0.03; 0.26]**
Heterogeneity: $Tau^2 = 0.0106$; $Chi^2 = 7.31$, df = 6 (P = 0.29); $I^2 = 18\%$								
Test for subgroup differences: $Chi^2 = 0.22$, df = 1 (P = 0.64)								

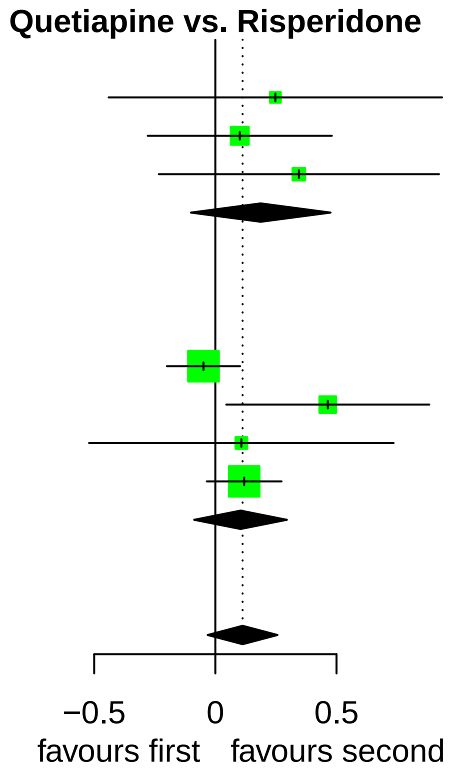

Study or Subgroup	first drug Mean	SD	Total	second drug Mean	SD	Total	Weight	SMD [95% CI]
**blinding = 1**								
Canive 2006	70.60	16.7528	4	57.80	16.7528	5	1.2%	0.68 [−0.70; 2.06]
Conley 2001	−12.80	15.9000	175	−12.90	16.1000	181	27.5%	0.01 [−0.20; 0.21]
Lieberman 2005 12weeks	−3.10	13.4568	339	−6.38	13.4568	331	36.5%	0.24 [0.09; 0.40]
Mori 2004	71.50	12.7758	19	69.40	12.7758	20	5.2%	0.16 [−0.47; 0.79]
**Total (95% CI)**			**537**			**537**	**70.3%**	**0.15 [−0.04; 0.34]**
Heterogeneity: $Tau^2 = 0.0128$; $Chi^2 = 3.8$, df = 3 (P = 0.28); $I^2 = 21\%$								
**blinding = 0**								
Chen 2018	79.20	19.0194	25	67.11	19.0194	28	6.5%	0.63 [0.07; 1.18]
McCue2006	−15.40	11.8240	57	−14.90	11.8240	52	12.5%	−0.04 [−0.42; 0.33]
van Bruggen 2003	−15.00	12.7400	24	−15.10	23.7600	18	5.4%	0.01 [−0.61; 0.62]
Yamashita 2004	−6.20	6.2791	20	−8.30	6.2791	20	5.2%	0.33 [−0.30; 0.95]
**Total (95% CI)**			**126**			**118**	**29.7%**	**0.20 [−0.13; 0.52]**
Heterogeneity: $Tau^2 = 0.0371$; $Chi^2 = 4.36$, df = 3 (P = 0.23); $I^2 = 31\%$								
**Total (95% CI)**			**663**			**655**	**100.0%**	**0.16 [0.01; 0.31]**

Heterogeneity: $Tau^2 = 0.0100$; $Chi^2 = 8.16$, df = 7 (P = 0.32); $I^2 = 14\%$

Test for subgroup differences: $Chi^2 = 0.05$, df = 1 (P = 0.82)

Risperidone vs. Olanzapine

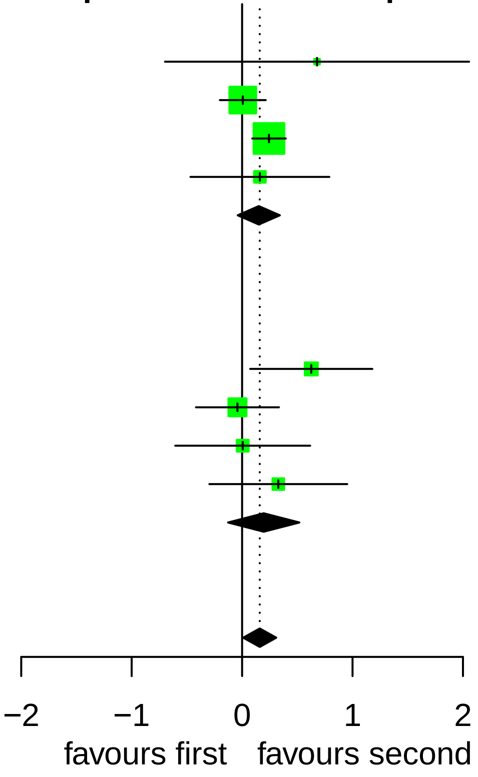

Study or Subgroup	first drug Mean	SD	Total	second drug Mean	SD	Total	Weight	SMD [95% CI]
**blinding = 0**								
McCue2006	−14.20	11.8240	50	−12.90	11.8240	53	34.1%	−0.11 [−0.50; 0.28]
**Total (95% CI)**			**50**			**53**	**34.1%**	**−0.11 [−0.50; 0.28]**
Heterogeneity: not applicable								
**blinding = 1**								
Zimbroff 2007	−21.60	18.7000	121	−24.60	19.1000	126	65.9%	0.16 [−0.09; 0.41]
**Total (95% CI)**			**121**			**126**	**65.9%**	**0.16 [−0.09; 0.41]**
Heterogeneity: not applicable								
**Total (95% CI)**			**171**			**179**	**100.0%**	**0.07 [−0.18; 0.32]**

Heterogeneity: $Tau^2 = 0.0081$; $Chi^2 = 1.30$, df = 1 (P = 0.26); $I^2 = 23\%$

Test for subgroup differences: $Chi^2 = 1.30$, df = 1 (P = 0.26)

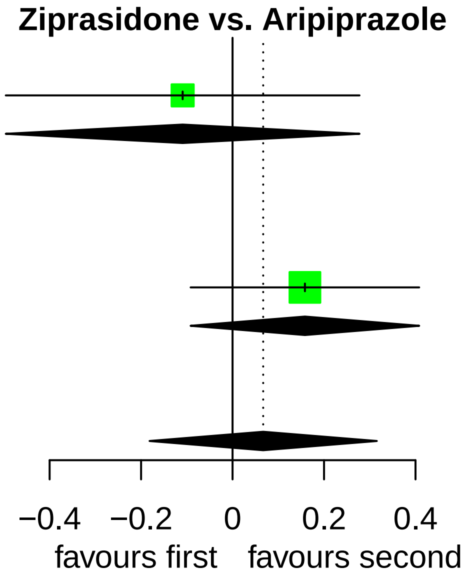

Study or Subgroup	first drug Mean	first drug SD	first drug Total	second drug Mean	second drug SD	second drug Total	Weight	SMD [95% CI]
**blinding = 1**								
Goff 1998	−11.90	15.0000	20	−11.60	15.1000	17	16.0%	−0.02 [−0.67; 0.63]
Schennach 2018	66.23	23.0100	54	74.99	20.3800	58	26.4%	−0.40 [−0.78; −0.03]
Study 115 2000	−8.44	22.9324	158	−15.20	22.9324	82	31.5%	0.29 [0.03; 0.56]
**Total (95% CI)**			**232**			**157**	**73.9%**	**−0.03 [−0.48; 0.42]**
Heterogeneity: $Tau^2 = 0.1125$; $Chi^2 = 8.8$, df = 2 (P = 0.01); $I^2 = 77\%$								
**blinding = 0**								
McCue2006	−14.20	11.8240	50	−16.40	11.8240	57	26.1%	0.18 [−0.20; 0.57]
**Total (95% CI)**			**50**			**57**	**26.1%**	**0.18 [−0.20; 0.57]**
Heterogeneity: not applicable								
**Total (95% CI)**			**282**			**214**	**100.0%**	**0.03 [−0.30; 0.37]**
Heterogeneity: $Tau^2 = 0.0741$; $Chi^2 = 9.17$, df = 3 (P = 0.03); $I^2 = 67\%$								
Test for subgroup differences: $Chi^2 = 0.50$, df = 1 (P = 0.48)								

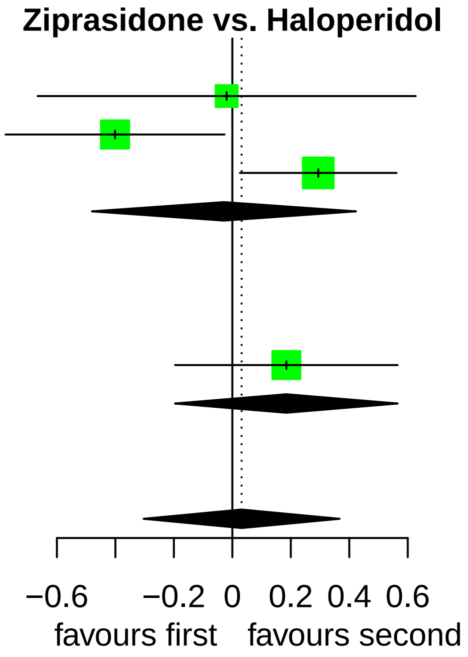

Study or Subgroup	first drug Mean	first drug SD	first drug Total	second drug Mean	second drug SD	second drug Total	Weight	SMD [95% CI]
**blinding = 1**								
Grootens 2009	−14.86	12.9900	34	−17.15	10.2500	33	8.4%	0.19 [−0.29; 0.67]
Lieberman 2005 12weeks	−2.74	13.4568	183	−6.38	13.4568	331	45.1%	0.27 [0.09; 0.45]
Simpson 2004	−11.45	13.5300	125	−11.95	13.5300	126	27.8%	0.04 [−0.21; 0.28]
Svestka 2005	60.29	18.3300	24	51.92	18.3300	24	6.0%	0.45 [−0.12; 1.02]
**Total (95% CI)**			**366**			**514**	**87.4%**	**0.20 [0.04; 0.36]**
Heterogeneity: $Tau^2 = 0.0053$; $Chi^2 = 2.97$, df = 3 (P = 0.40); $I^2 = 0\%$								
**blinding = 0**								
McCue2006	−14.20	11.8240	50	−14.90	11.8240	52	12.6%	0.06 [−0.33; 0.45]
**Total (95% CI)**			**50**			**52**	**12.6%**	**0.06 [−0.33; 0.45]**
Heterogeneity: not applicable								
**Total (95% CI)**			**416**			**566**	**100.0%**	**0.18 [0.04; 0.33]**

Heterogeneity: $Tau^2 = 0.0033$; $Chi^2 = 3.44$, df = 4 (P = 0.49); $I^2 = 0\%$

Test for subgroup differences: $Chi^2 = 0.43$, df = 1 (P = 0.51)

Ziprasidone vs. Olanzapine

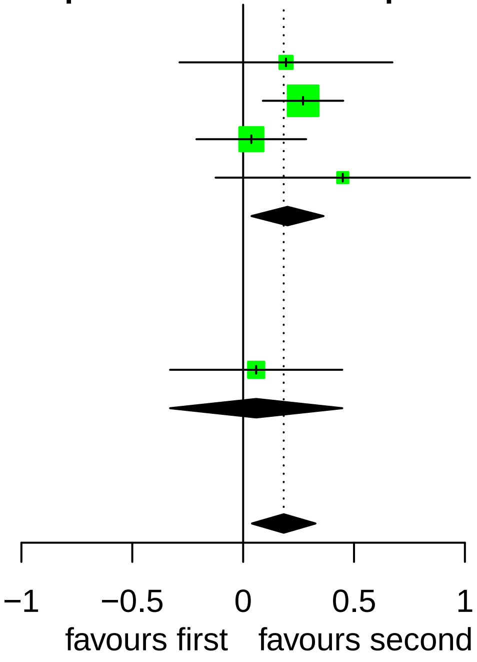

Study or Subgroup	first drug Mean	SD	Total	second drug Mean	SD	Total	Weight	SMD [95% CI]
**blinding = 1**								
Lieberman 2005 12weeks	−2.74	13.4568	183	−3.76	13.4568	333	82.5%	0.08 [−0.10; 0.26]
**Total (95% CI)**			**183**			**333**	**82.5%**	**0.08 [−0.10; 0.26]**
Heterogeneity: not applicable								
**blinding = 0**								
McCue2006	−14.20	11.8240	50	−14.20	11.8240	50	17.5%	0.00 [−0.39; 0.39]
**Total (95% CI)**			**50**			**50**	**17.5%**	**0.00 [−0.39; 0.39]**
Heterogeneity: not applicable								
**Total (95% CI)**			**233**			**383**	**100.0%**	**0.06 [−0.10; 0.23]**

Heterogeneity: $Tau^2 = 0$; $Chi^2 = 0.12$, df = 1 (P = 0.73); $I^2 = 0\%$

Test for subgroup differences: $Chi^2 = 0.12$, df = 1 (P = 0.73)

Ziprasidone vs. Quetiapine

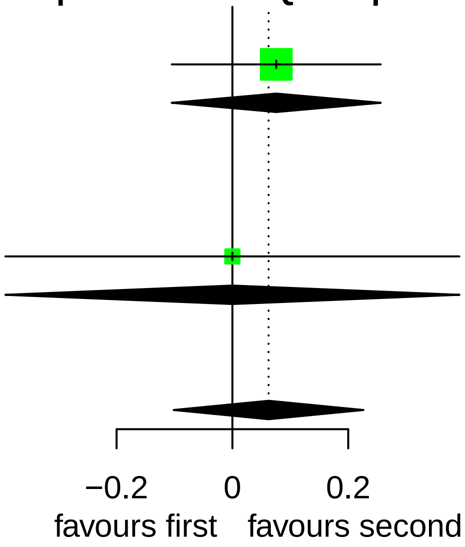

Study or Subgroup	first drug Mean	SD	Total	second drug Mean	SD	Total	Weight	SMD [95% CI]
blinding = 1								
Addington 2004	−25.80	20.4892	125	−27.30	20.4892	132	24.1%	0.07 [−0.17; 0.32]
Lieberman 2005 12weeks	−2.74	13.4568	183	−3.10	13.4568	339	44.7%	0.03 [−0.15; 0.21]
Zhang 2011	−35.60	14.9969	96	−37.10	14.7822	111	19.3%	0.10 [−0.17; 0.37]
Total (95% CI)			404			582	88.1%	0.06 [−0.07; 0.18]
Heterogeneity: Tau^2 = 0; Chi^2 = 0.22, df = 2 (P = 0.90); I^2 = 0%								
blinding = 0								
McCue2006	−14.20	11.8240	50	−15.40	11.8240	57	10.0%	0.10 [−0.28; 0.48]
Sonmez 2009	64.30	18.0600	10	62.00	23.1400	10	1.9%	0.11 [−0.77; 0.98]
Total (95% CI)			60			67	11.9%	0.10 [−0.25; 0.45]
Heterogeneity: Tau^2 = 0; Chi^2 = 0, df = 1 (P = 0.99); I^2 = 0%								
**Total (95% CI)**			**464**			**649**	**100.0%**	**0.06 [−0.06; 0.18]**
Heterogeneity: Tau^2 = 0; Chi^2 = 0.28, df = 4 (P = 0.99); I^2 = 0%								
Test for subgroup differences: Chi^2 = 0.06, df = 1 (P = 0.81)								

Ziprasidone vs. Risperidone

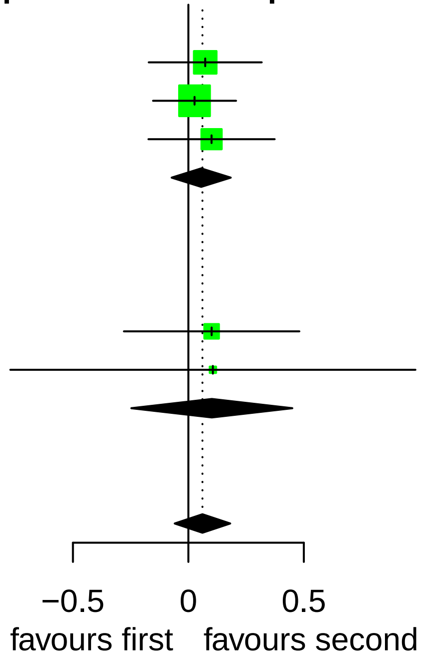

Figure 4b overall symptoms sensitivity analysis excluding single-blind RCTs difference between blinded and open trials, more recent drug listed first

Study	TE	SE	Weight	SMD [95% CI]
Aripiprazole vs. Ziprasidone, (nbl=247, nop=103)	−0.27	0.2349	5.1%	−0.27 [−0.73; 0.19]
Ziprasidone vs. Quetiapine, (nbl=516, nop=100)	0.08	0.2202	5.8%	0.08 [−0.36; 0.51]
Quetiapine vs. Olanzapine, (nbl=779, nop=150)	−0.15	0.3129	2.9%	−0.15 [−0.76; 0.47]
Quetiapine vs. Risperidone, (nbl=1447, nop=188)	−0.08	0.1766	9.0%	−0.08 [−0.43; 0.26]
Ziprasidone vs. Olanzapine, (nbl=880, nop=102)	0.14	0.2148	6.1%	0.14 [−0.28; 0.56]
Ziprasidone vs. Haloperidol, (nbl=389, nop=107)	−0.21	0.3012	3.1%	−0.21 [−0.80; 0.38]
Aripiprazole vs. Olanzapine, (nbl=1506, nop=105)	0.02	0.2023	6.8%	0.02 [−0.38; 0.42]
Aripiprazole vs. Haloperidol, (nbl=482, nop=207)	−0.23	0.1723	9.4%	−0.23 [−0.57; 0.10]
Brexpiprazole vs. Aripiprazole, (nbl=140, nop=97)	0.34	0.2782	3.6%	0.34 [−0.20; 0.89]
Aripiprazole vs. Risperidone, (nbl=372, nop=186)	0.12	0.2235	5.6%	0.12 [−0.32; 0.56]
Olanzapine vs. Risperidone, (nbl=1074, nop=244)	0.04	0.1916	7.6%	0.04 [−0.33; 0.42]
Zuclopenthixol vs. Haloperidol, (nbl=118, nop=88)	−0.11	0.2826	3.5%	−0.11 [−0.67; 0.44]
Quetiapine vs. Haloperidol, (nbl=778, nop=221)	−0.06	0.1536	11.9%	−0.06 [−0.36; 0.25]
Olanzapine vs. Haloperidol, (nbl=2887, nop=132)	−0.18	0.1852	8.2%	−0.18 [−0.54; 0.18]
Ziprasidone vs. Risperidone, (nbl=986, nop=127)	−0.05	0.1895	7.8%	−0.05 [−0.42; 0.33]
Risperidone vs. Haloperidol, (nbl=1579, nop=175)	0.07	0.2721	3.8%	0.07 [−0.46; 0.61]
**Total (95% CI)**			**100.0%**	**−0.04 [−0.15; 0.06]**

Heterogeneity: $Tau^2 = 0$; $Chi^2 = 7.19$, df = 15 (P = 0.95); $I^2 = 0\%$

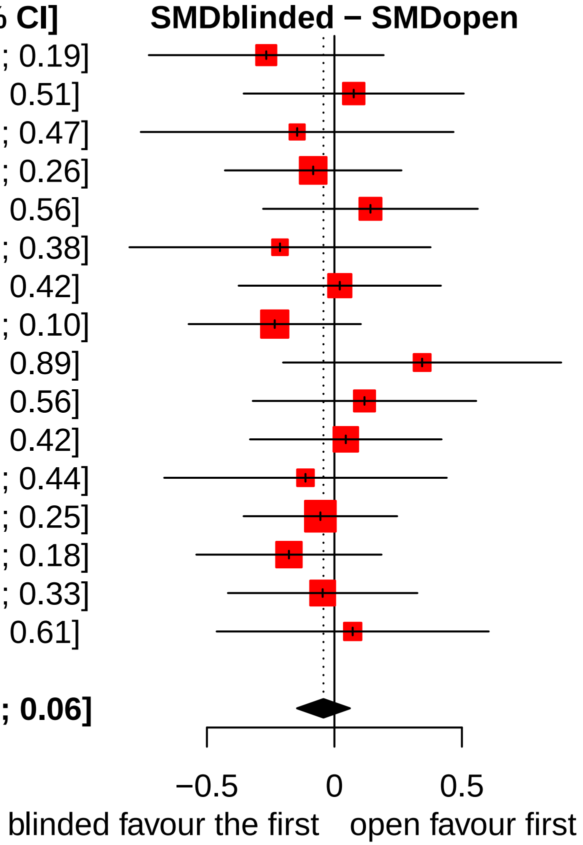

Figure 4c overall symptoms sensitivity analysis excluding single-blind RCTs difference between blinded and open trials, less efficacious drug listed first

Study	TE	SE	Weight	SMD [95% CI]
Ziprasidone vs. Aripiprazole, (nbl=247, nop=103)	0.27	0.2349	5.1%	0.27 [−0.19; 0.73]
Ziprasidone vs. Quetiapine, (nbl=516, nop=100)	0.08	0.2202	5.8%	0.08 [−0.36; 0.51]
Quetiapine vs. Olanzapine, (nbl=779, nop=150)	−0.15	0.3129	2.9%	−0.15 [−0.76; 0.47]
Quetiapine vs. Risperidone, (nbl=1447, nop=188)	−0.08	0.1766	9.0%	−0.08 [−0.43; 0.26]
Ziprasidone vs. Olanzapine, (nbl=880, nop=102)	0.14	0.2148	6.1%	0.14 [−0.28; 0.56]
Ziprasidone vs. Haloperidol, (nbl=389, nop=107)	−0.21	0.3012	3.1%	−0.21 [−0.80; 0.38]
Aripiprazole vs. Olanzapine, (nbl=1506, nop=105)	0.02	0.2023	6.8%	0.02 [−0.38; 0.42]
Aripiprazole vs. Haloperidol, (nbl=482, nop=207)	−0.23	0.1723	9.4%	−0.23 [−0.57; 0.10]
Brexpiprazole vs. Aripiprazole, (nbl=140, nop=97)	0.34	0.2782	3.6%	0.34 [−0.20; 0.89]
Aripiprazole vs. Risperidone, (nbl=372, nop=186)	0.12	0.2235	5.6%	0.12 [−0.32; 0.56]
Risperidone vs. Olanzapine, (nbl=1074, nop=244)	−0.04	0.1916	7.6%	−0.04 [−0.42; 0.33]
Haloperidol vs. Zuclopenthixol, (nbl=118, nop=88)	0.11	0.2826	3.5%	0.11 [−0.44; 0.67]
Quetiapine vs. Haloperidol, (nbl=778, nop=221)	−0.06	0.1536	11.9%	−0.06 [−0.36; 0.25]
Haloperidol vs. Olanzapine, (nbl=2887, nop=132)	0.18	0.1852	8.2%	0.18 [−0.18; 0.54]
Ziprasidone vs. Risperidone, (nbl=986, nop=127)	−0.05	0.1895	7.8%	−0.05 [−0.42; 0.33]
Haloperidol vs. Risperidone, (nbl=1579, nop=175)	−0.07	0.2721	3.8%	−0.07 [−0.61; 0.46]
**Total (95% CI)**			**100.0%**	**0.01 [−0.09; 0.11]**

Heterogeneity: $Tau^2 = 0$; $Chi^2 = 7.82$, df = 15 (P = 0.93); $I^2 = 0\%$

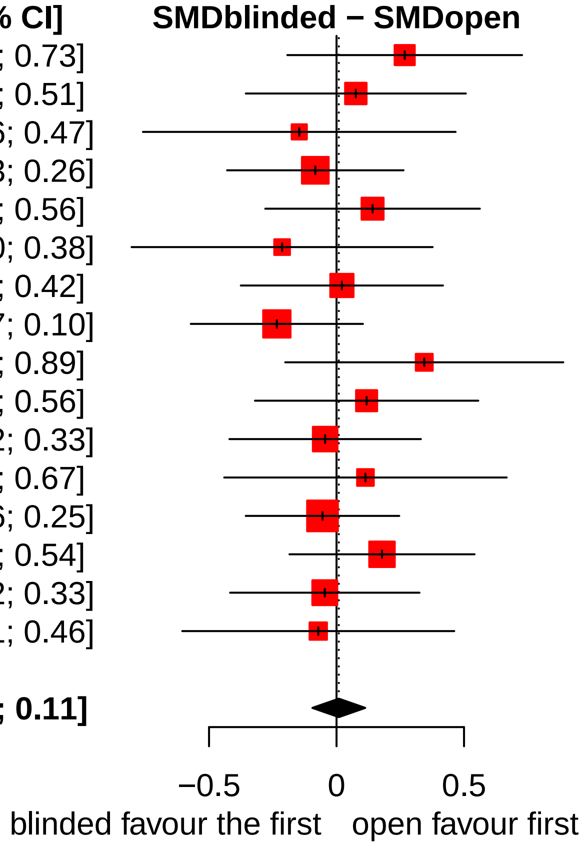

Figure 4d overall symptoms sensitivity analysis excluding single-blind studies sponsored versus non sponsored drugs in blinded and open trials

Study or Subgroup	TE	SE	Weight	SMD [95% CI]
blinding = 1				
Addington 2004	−0.07	0.1248	1.3%	−0.07 [−0.32; 0.17]
Arvanitis 1997	−0.04	0.1721	1.0%	−0.04 [−0.38; 0.30]
Azorin 2006	0.15	0.1529	1.1%	0.15 [−0.15; 0.45]
Beasley 1996b	0.07	0.1503	1.1%	0.07 [−0.23; 0.36]
Beasley 1997	0.18	0.1367	1.2%	0.18 [−0.09; 0.45]
Bernardo 2001	−0.24	0.3865	0.3%	−0.24 [−1.00; 0.52]
Blin 1996	0.75	0.2766	0.6%	0.75 [0.20; 1.29]
Boulay 2007	−0.46	0.4193	0.3%	−0.46 [−1.28; 0.36]
Brook 1998	0.09	0.3029	0.5%	0.09 [−0.50; 0.68]
Bueno 1979	−0.77	0.3361	0.4%	−0.77 [−1.42; −0.11]
Canive 2006	0.76	0.6946	0.1%	0.76 [−0.60; 2.13]
Chan 2007	−0.08	0.2233	0.7%	−0.08 [−0.52; 0.35]
Chouinard 1993	0.57	0.3112	0.5%	0.57 [−0.04; 1.18]
Clark 1970a	−0.22	0.3727	0.4%	−0.22 [−0.95; 0.52]
Clark 1972	0.27	0.3397	0.4%	0.27 [−0.40; 0.93]
Clark 1975	−0.39	0.4042	0.3%	−0.39 [−1.19; 0.40]
Claus 1992	−0.13	0.3089	0.5%	−0.13 [−0.73; 0.48]
Conley 2001	−0.01	0.1060	1.4%	−0.01 [−0.21; 0.20]
Cooper 2000a	0.73	0.2006	0.8%	0.73 [0.34; 1.12]
Copolov 2000	−0.14	0.0958	1.5%	−0.14 [−0.33; 0.05]
Costa e Silva 1989	0.33	0.3357	0.4%	0.33 [−0.32; 0.99]
Cutler 2008	−0.02	0.1024	1.4%	−0.02 [−0.22; 0.18]
Davidson 2007	−0.09	0.1268	1.3%	−0.09 [−0.33; 0.16]
Dieterle 1991	−0.16	0.3167	0.5%	−0.16 [−0.78; 0.46]
Durgam 2014	−0.35	0.0985	1.4%	−0.35 [−0.54; −0.15]
Fleischhacker 1989	−0.57	0.3225	0.5%	−0.57 [−1.20; 0.07]
Fleischhacker 2009_6weeks	−0.24	0.0764	1.6%	−0.24 [−0.39; −0.09]
Freeman 1969	0.31	0.3268	0.4%	0.31 [−0.33; 0.95]
Gattaz 2004	−0.10	0.4006	0.3%	−0.10 [−0.89; 0.68]
Gelenberg 1979	1.06	0.5524	0.2%	1.06 [−0.02; 2.14]
Goff 1998	0.02	0.3299	0.4%	0.02 [−0.63; 0.67]
Grootens 2009	−0.20	0.2449	0.7%	−0.20 [−0.68; 0.28]
Hale 2000	−0.03	0.1108	1.4%	−0.03 [−0.25; 0.19]
Hera 041−021	−0.17	0.1207	1.3%	−0.17 [−0.40; 0.07]
Hera 041−022	−0.22	0.1539	1.1%	−0.22 [−0.52; 0.08]
Honigfeld 1984a	1.05	0.4193	0.3%	1.05 [0.22; 1.87]
Honigfeld 1984c	0.76	0.2331	0.7%	0.76 [0.31; 1.22]
Hoyberg 1993	0.16	0.1993	0.8%	0.16 [−0.23; 0.55]
Huttunen 1995	0.15	0.2023	0.8%	0.15 [−0.25; 0.54]
Hwang 2001	−0.25	0.2400	0.7%	−0.25 [−0.72; 0.22]
Hwang 2003	−0.24	0.2934	0.5%	−0.24 [−0.81; 0.34]
Ishigooka 2001	0.20	0.1595	1.1%	0.20 [−0.11; 0.51]
Itoh 1977	0.25	0.2145	0.8%	0.25 [−0.17; 0.67]
Janicak 2001	0.08	0.2542	0.6%	0.08 [−0.42; 0.58]
Janssen CR012625	−0.04	0.1709	1.0%	−0.04 [−0.38; 0.29]
Kane 2002	−0.01	0.1205	1.3%	−0.01 [−0.25; 0.22]
Kane 2007b	−0.02	0.1024	1.4%	−0.02 [−0.22; 0.18]
Kane 2009 8 weeks	0.21	0.0843	1.5%	0.21 [0.05; 0.38]
Kane 2010a	0.02	0.1166	1.3%	0.02 [−0.21; 0.25]
Kingstone 1970	0.12	0.3127	0.5%	0.12 [−0.49; 0.74]
Kluge 2007	−0.23	0.3664	0.4%	−0.23 [−0.95; 0.49]
Kramer 1978	0.21	0.3927	0.3%	0.21 [−0.56; 0.98]
Lundbeck NCT00864045	−0.23	0.1029	1.4%	−0.23 [−0.43; −0.03]
Marder 1994	0.62	0.1817	0.9%	0.62 [0.27; 0.98]
Marder 2007c	−0.09	0.1185	1.3%	−0.09 [−0.32; 0.14]
Martin 2002	0.08	0.1037	1.4%	0.08 [−0.13; 0.28]
McQuade 2004_6weeks	−0.12	0.1431	1.1%	−0.12 [−0.40; 0.16]
Meyer−Lindenberg 1997	−0.08	0.2830	0.5%	−0.08 [−0.63; 0.48]
Min 1993	−0.46	0.3437	0.4%	−0.46 [−1.13; 0.21]
Möller 1997	0.20	0.1462	1.1%	0.20 [−0.09; 0.48]
Moyano 1975	0.49	0.3063	0.5%	0.49 [−0.11; 1.09]
Murasaki 2001	−0.01	0.1464	1.1%	−0.01 [−0.29; 0.28]
NCT00350467	−0.10	0.1184	1.3%	−0.10 [−0.33; 0.13]
NCT00882518	−0.14	0.1140	1.3%	−0.14 [−0.37; 0.08]
NCT01104766	0.02	0.0997	1.4%	0.02 [−0.17; 0.22]
NCT01617187	0.01	0.1767	1.0%	0.01 [−0.34; 0.35]
NCT01810380	−0.22	0.1158	1.3%	−0.22 [−0.44; 0.01]
Petit 1996	0.23	0.1802	0.9%	0.23 [−0.13; 0.58]
Peuskens 1995	0.14	0.0818	1.5%	0.14 [−0.02; 0.30]
Peuskens 1997	0.05	0.1429	1.2%	0.05 [−0.23; 0.33]
Peuskens 1999	0.17	0.1327	1.2%	0.17 [−0.09; 0.43]
Potkin 2003	−0.07	0.1253	1.3%	−0.07 [−0.32; 0.17]
Potkin 2007c	0.15	0.1876	0.9%	0.15 [−0.22; 0.51]
Puech 1998	0.40	0.1575	1.1%	0.40 [0.09; 0.71]
Riedel 2007	−0.16	0.3489	0.4%	−0.16 [−0.85; 0.52]
Rüther 1988	0.60	0.4093	0.3%	0.60 [−0.20; 1.41]
Schennach 2018	0.40	0.1910	0.9%	0.40 [0.03; 0.78]
Selman 1976	−0.13	0.2726	0.6%	−0.13 [−0.67; 0.40]
Shah 2011	0.01	0.1394	1.2%	0.01 [−0.26; 0.29]
Simpson 2004	−0.04	0.1263	1.3%	−0.04 [−0.28; 0.21]
Study 049	−0.28	0.1466	1.1%	−0.28 [−0.57; 0.01]
Study 231	−0.19	0.1120	1.3%	−0.19 [−0.41; 0.03]
Study 233	−0.18	0.1128	1.3%	−0.18 [−0.40; 0.05]
Study 3000	−0.19	0.1325	1.2%	−0.19 [−0.45; 0.07]
Study 3001	−0.02	0.0951	1.5%	−0.02 [−0.21; 0.16]
Study 3002	−0.09	0.0984	1.4%	−0.09 [−0.28; 0.11]
Study 3003	−0.04	0.1046	1.4%	−0.04 [−0.24; 0.17]
Study 3004	−0.19	0.1167	1.3%	−0.19 [−0.42; 0.03]
Study 3005	−0.30	0.0977	1.4%	−0.30 [−0.49; −0.11]
Study 93202 2002	−0.30	0.2476	0.7%	−0.30 [−0.79; 0.19]
Study 94202 2002	0.22	0.1930	0.9%	0.22 [−0.16; 0.60]
Tollefson 1997	0.20	0.0484	1.7%	0.20 [0.11; 0.30]
Tuason 1984	−0.18	0.2430	0.7%	−0.18 [−0.66; 0.30]
van der Velde 1975	0.61	0.2869	0.5%	0.61 [0.04; 1.17]
Wagner 2005	−0.52	0.3390	0.4%	−0.52 [−1.19; 0.14]
Wetzel 1991	0.76	0.3556	0.4%	0.76 [0.06; 1.45]
Wetzel 1998	0.49	0.1802	0.9%	0.49 [0.13; 0.84]
Zborowski 1995	−0.27	0.1342	1.2%	−0.27 [−0.53; −0.01]
Zhang 2011	−0.10	0.1395	1.2%	−0.10 [−0.37; 0.17]
Zhong 2006	−0.12	0.0788	1.6%	−0.12 [−0.27; 0.04]
Ziegler 1989	0.21	0.3213	0.5%	0.21 [−0.42; 0.84]
Zimbroff 1997	−0.08	0.1222	1.3%	−0.08 [−0.32; 0.16]
Zimbroff 2007	−0.16	0.1275	1.2%	−0.16 [−0.41; 0.09]
Total (95% CI)			95.3%	0.00 [−0.04; 0.05]
Heterogeneity: Tau^2 = 0.0236; Chi^2 = 229.52, df = 102 (P < 0.01); I^2 = 56%				
blinding = 0				
Avasthi 2001	0.33	0.4558	0.3%	0.33 [−0.56; 1.22]
de Boer 2011	0.35	0.3569	0.4%	0.35 [−0.35; 1.05]
de Oliviera 2009	−0.16	0.2180	0.8%	−0.16 [−0.59; 0.27]
Dossenbach 2007	1.23	0.2144	0.8%	1.23 [0.81; 1.65]
Knegtering 2004	−0.25	0.3510	0.4%	−0.25 [−0.94; 0.43]
Loza 1999	0.74	0.3394	0.4%	0.74 [0.08; 1.41]
Maat 2014	0.26	0.3108	0.5%	0.26 [−0.35; 0.87]
Taneli 2003	−0.11	0.2274	0.7%	−0.11 [−0.55; 0.34]
van Bruggen 2003	0.01	0.3118	0.5%	0.01 [−0.61; 0.62]
Total (95% CI)			4.7%	0.27 [−0.08; 0.62]
Heterogeneity: Tau^2 = 0.1916; Chi^2 = 31.45, df = 8 (P < 0.01); I^2 = 75%				
**Total (95% CI)**			**100.0%**	**0.02 [−0.03; 0.07]**

Heterogeneity: Tau^2 = 0.0335; Chi^2 = 271.02, df = 111 (P < 0.01); I^2 = 59%
Test for subgroup differences: Chi^2 = 2.26, df = 1 (P = 0.13)

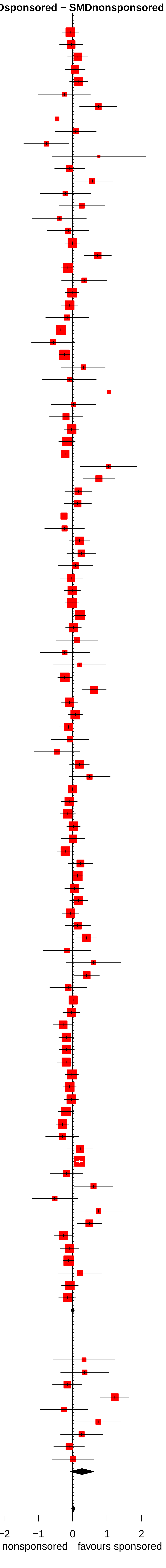

eFigure 5

Positive symptoms

1. Results of individual comparisons
2. Difference between blinded and open RCTs by recency
3. Difference between blinded and open RCTs by efficacy
4. Differences between blinded and open RCTs sponsored versus non-sponsored drugs

eFigure 5a positive symptoms individual comparisons

Study or Subgroup	first drug Mean	first drug SD	first drug Total	second drug Mean	second drug SD	second drug Total	Weight	SMD [95% CI]
**blinding = 0**								
de Oliviera 2009	15.20	8.0000	66	13.00	4.8000	31	17.9%	0.31 [−0.12; 0.73]
**Total (95% CI)**			**66**			**31**	**17.9%**	**0.31 [−0.12; 0.73]**
Heterogeneity: not applicable								
**blinding = 1**								
Kane 2002	−4.00	7.7572	204	−4.40	7.7572	104	59.0%	0.05 [−0.18; 0.29]
Study 94202 2002	−3.30	4.6313	54	−3.80	4.6313	54	23.1%	0.11 [−0.27; 0.48]
**Total (95% CI)**			**258**			**158**	**82.1%**	**0.07 [−0.13; 0.27]**
Heterogeneity: $Tau^2 = 0$; $Chi^2 = 0.06$, df = 1 (P = 0.81); $I^2 = 0\%$								
**Total (95% CI)**			**324**			**189**	**100.0%**	**0.11 [−0.07; 0.29]**

Heterogeneity: $Tau^2 = 0$; $Chi^2 = 1.03$, df = 2 (P = 0.60); $I^2 = 0\%$

Test for subgroup differences: $Chi^2 = 0.97$, df = 1 (P = 0.32)

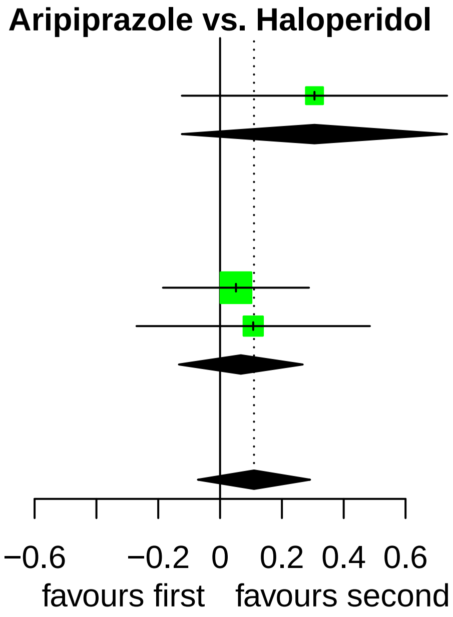

Study or Subgroup	first drug Mean	SD	Total	second drug Mean	SD	Total	Weight	SMD [95% CI]
**blinding = 1**								
Chan 2007	−5.80	6.9000	49	−8.10	5.8000	34	17.7%	0.35 [−0.09; 0.79]
Hatta 2009	−6.50	9.5172	21	−10.80	9.5172	20	8.9%	0.44 [−0.18; 1.06]
Potkin 2003	−4.41	6.6503	194	−5.20	6.6503	95	57.0%	0.12 [−0.13; 0.36]
**Total (95% CI)**			**264**			**149**	**83.6%**	**0.20 [0.00; 0.41]**
Heterogeneity: $Tau^2 = 0$; $Chi^2 = 1.47$, df = 2 (P = 0.48); $I^2 = 0\%$								
**blinding = 0**								
de Boer 2011	−2.17	14.9000	17	−1.62	12.7000	15	7.1%	−0.04 [−0.73; 0.66]
Maat 2014	−7.00	5.4000	17	−6.00	4.9100	27	9.3%	−0.19 [−0.80; 0.42]
**Total (95% CI)**			**34**			**42**	**16.4%**	**−0.13 [−0.58; 0.33]**
Heterogeneity: $Tau^2 = 0$; $Chi^2 = 0.11$, df = 1 (P = 0.74); $I^2 = 0\%$								
**Total (95% CI)**			**298**			**191**	**100.0%**	**0.15 [−0.04; 0.33]**
Heterogeneity: $Tau^2 = 0$; $Chi^2 = 3.23$, df = 4 (P = 0.52); $I^2 = 0\%$								
Test for subgroup differences: $Chi^2 = 1.65$, df = 1 (P = 0.20)								

Aripiprazole vs. Risperidone

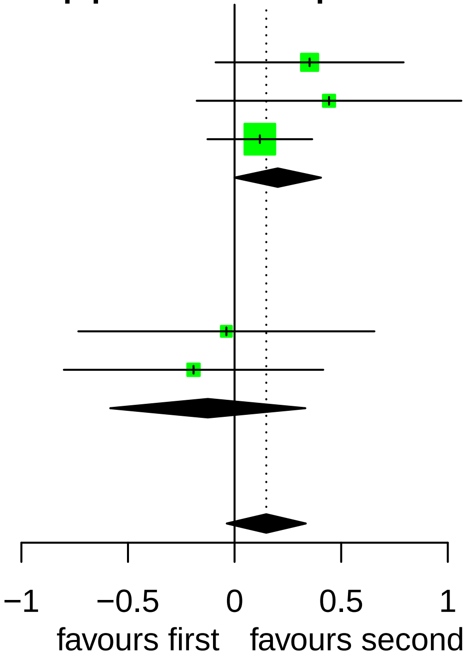

Study or Subgroup	first drug Mean	first drug SD	first drug Total	second drug Mean	second drug SD	second drug Total	Weight	SMD [95% CI]
**blinding = 1**								
Allan 1998	18.90	6.0000	10	19.00	8.0000	13	1.0%	−0.01 [−0.84; 0.81]
Beasley 1996b	−4.60	5.5254	68	−4.55	5.5254	128	7.6%	−0.01 [−0.30; 0.29]
Beasley 1997	−6.50	8.6000	79	−7.21	8.0300	168	9.2%	0.09 [−0.18; 0.35]
Bernardo 2001	16.40	7.1000	13	18.80	7.6000	14	1.1%	−0.32 [−1.08; 0.44]
Ishigooka 2001	−1.29	6.2600	78	−2.44	5.3700	80	6.8%	0.20 [−0.12; 0.51]
Tollefson 1997	−3.80	6.3000	636	−4.70	6.8000	1312	73.4%	0.14 [0.04; 0.23]
**Total (95% CI)**			**884**			**1715**	**99.2%**	**0.12 [0.04; 0.20]**
Heterogeneity: $Tau^2 = 0$; $Chi^2 = 2.49$, df = 5 (P = 0.78); $I^2 = 0\%$								
**blinding = 0**								
Avasthi 2001	10.86	8.4900	7	11.44	4.1100	16	0.8%	−0.10 [−0.99; 0.79]
**Total (95% CI)**			**7**			**16**	**0.8%**	**−0.10 [−0.99; 0.79]**
Heterogeneity: not applicable								
**Total (95% CI)**			**891**			**1731**	**100.0%**	**0.12 [0.03; 0.20]**

Heterogeneity: $Tau^2 = 0$; $Chi^2 = 2.71$, df = 6 (P = 0.84); $I^2 = 0\%$

Test for subgroup differences: $Chi^2 = 0.22$, df = 1 (P = 0.64)

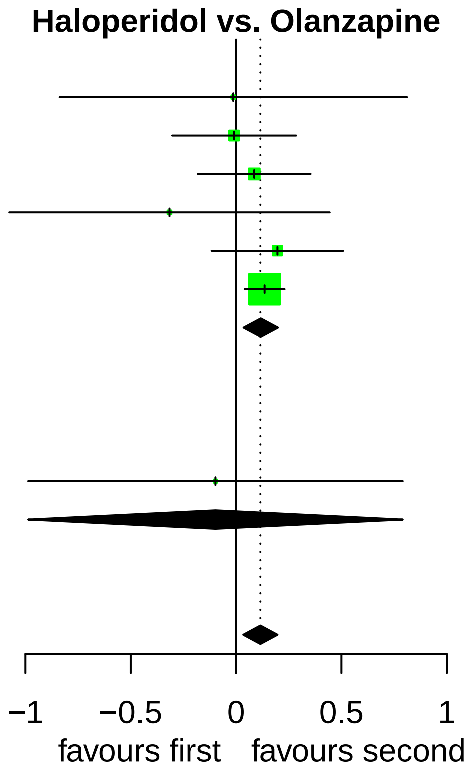

Study or Subgroup	first drug Mean	SD	Total	second drug Mean	SD	Total	Weight	SMD [95% CI]
**blinding = 1**								
Abdolahian 2008	21.30	3.5700	30	18.20	3.5700	35	7.9%	0.86 [0.35; 1.37]
Blin 1996	−9.10	9.5116	20	−14.70	9.5116	21	5.8%	0.58 [−0.05; 1.20]
Chouinard 1993	−3.90	8.0380	21	−7.50	8.0380	22	6.1%	0.44 [−0.17; 1.05]
Claus 1992	15.40	5.9600	21	15.90	6.4200	21	6.1%	−0.08 [−0.68; 0.53]
Ghaleiha 2011	16.03	13.5408	16	13.62	13.5408	16	4.9%	0.17 [−0.52; 0.87]
Janicak 2001	−3.40	6.1000	32	−4.20	5.8900	30	8.2%	0.13 [−0.37; 0.63]
Liu 2000	−9.70	7.3000	19	−8.80	7.4000	19	5.6%	−0.12 [−0.76; 0.52]
Marder 1994	−2.40	6.5676	64	−4.70	6.5676	63	12.9%	0.35 [0.00; 0.70]
Min 1993	−3.30	3.6700	19	−4.30	2.1700	16	5.2%	0.32 [−0.35; 0.99]
Peuskens 1995	−3.90	6.4200	223	−4.35	6.8600	455	23.1%	0.07 [−0.09; 0.23]
See 1999	13.05	2.9400	10	10.90	2.5000	10	3.0%	0.75 [−0.16; 1.67]
Yen 2004	−9.00	5.5000	20	−7.80	4.7000	21	5.9%	−0.23 [−0.85; 0.38]
**Total (95% CI)**			**495**			**729**	**94.6%**	**0.24 [0.06; 0.42]**
Heterogeneity: $Tau^2 = 0.0302$; $Chi^2 = 16.79$, df = 11 (P = 0.11); $I^2 = 34\%$								
**blinding = 0**								
Tamrakar 2006	−12.18	3.7100	18	−12.39	6.8100	18	5.4%	0.04 [−0.62; 0.69]
**Total (95% CI)**			**18**			**18**	**5.4%**	**0.04 [−0.62; 0.69]**
Heterogeneity: not applicable								
**Total (95% CI)**			**513**			**747**	**100.0%**	**0.22 [0.06; 0.39]**
Heterogeneity: $Tau^2 = 0.0252$; $Chi^2 = 16.95$, df = 12 (P = 0.15); $I^2 = 29\%$								
Test for subgroup differences: $Chi^2 = 0.33$, df = 1 (P = 0.56)								

Haloperidol vs. Risperidone

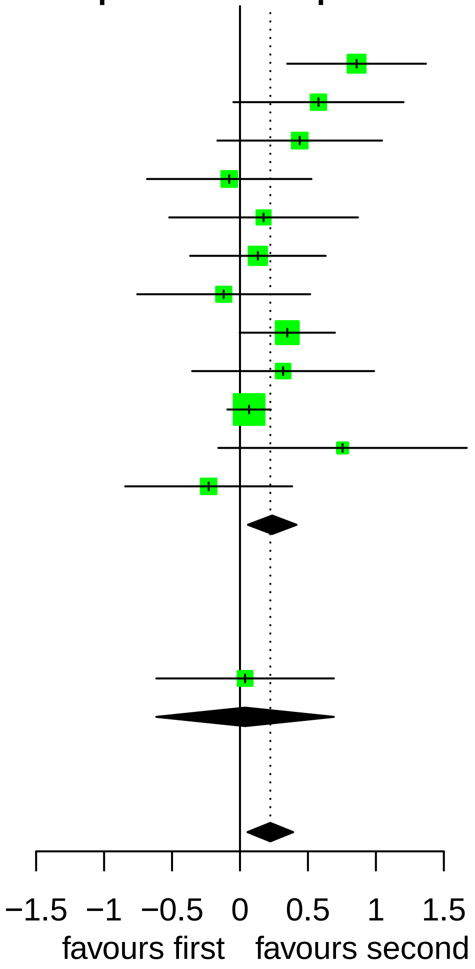

Study or Subgroup	first drug Mean	SD	Total	second drug Mean	SD	Total	Weight	SMD [95% CI]
**blinding = 1**								
Arvanitis 1997	−0.66	1.2349	104	−0.74	1.2349	50	18.5%	0.06 [−0.27; 0.40]
Copolov 2000	−5.60	8.1000	218	−8.00	8.0000	219	46.1%	0.30 [0.11; 0.49]
Murasaki 2001	15.90	8.2000	97	15.50	7.2000	90	24.3%	0.05 [−0.24; 0.34]
**Total (95% CI)**			**419**			**359**	**88.8%**	**0.17 [−0.01; 0.36]**
Heterogeneity: Tau^2 = 0.0086; Chi^2 = 2.67, df = 2 (P = 0.26); I^2 = 25%								
**blinding = 0**								
Taneli 2003	17.80	9.0000	45	16.10	8.1000	34	11.2%	0.20 [−0.25; 0.64]
**Total (95% CI)**			**45**			**34**	**11.2%**	**0.20 [−0.25; 0.64]**
Heterogeneity: not applicable								
**Total (95% CI)**			**464**			**393**	**100.0%**	**0.18 [0.03; 0.34]**

Heterogeneity: Tau^2 = 0.0044; Chi^2 = 2.67, df = 3 (P = 0.44); I^2 = 0%

Test for subgroup differences: Chi^2 = 0.01, df = 1 (P = 0.93)

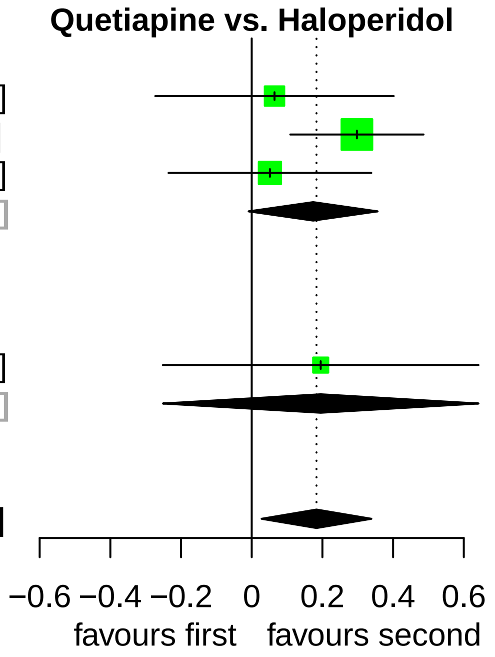

Study or Subgroup	first drug Mean	SD	Total	second drug Mean	SD	Total	Weight	SMD [95% CI]
**blinding = 1**								
Canive 2006	15.74	7.0425	4	14.35	7.0425	5	1.7%	0.18 [−1.14; 1.49]
Conley 2001	−4.80	6.8000	175	−4.30	6.3000	181	28.5%	−0.08 [−0.28; 0.13]
Hatta 2009	−10.80	9.5172	20	−12.60	9.5172	17	6.2%	0.19 [−0.46; 0.83]
Hatta 2013	17.85	6.5900	20	16.91	6.9669	22	7.0%	0.14 [−0.47; 0.74]
Lieberman 2005 12weeks	−1.16	4.7105	339	−2.15	4.7105	331	35.2%	0.21 [0.06; 0.36]
Mori 2004	10.80	3.3301	19	11.60	3.3301	20	6.5%	−0.24 [−0.87; 0.39]
Sacchetti 2008	−12.61	6.7027	25	−12.87	6.7027	25	8.1%	0.04 [−0.52; 0.59]
**Total (95% CI)**			**602**			**601**	**93.2%**	**0.07 [−0.10; 0.24]**
Heterogeneity: $Tau^2 = 0.0134$; $Chi^2 = 6$, df = 6 (P = 0.42); $I^2 = 0\%$								
**blinding = 0**								
van Bruggen 2003	−5.50	5.8800	24	−3.20	5.5100	18	6.8%	−0.39 [−1.01; 0.22]
**Total (95% CI)**			**24**			**18**	**6.8%**	**−0.39 [−1.01; 0.22]**
Heterogeneity: not applicable								
**Total (95% CI)**			**626**			**619**	**100.0%**	**0.04 [−0.14; 0.21]**

Heterogeneity: $Tau^2 = 0.0163$; $Chi^2 = 8.38$, df = 7 (P = 0.30); $I^2 = 16\%$

Test for subgroup differences: $Chi^2 = 2.02$, df = 1 (P = 0.16)

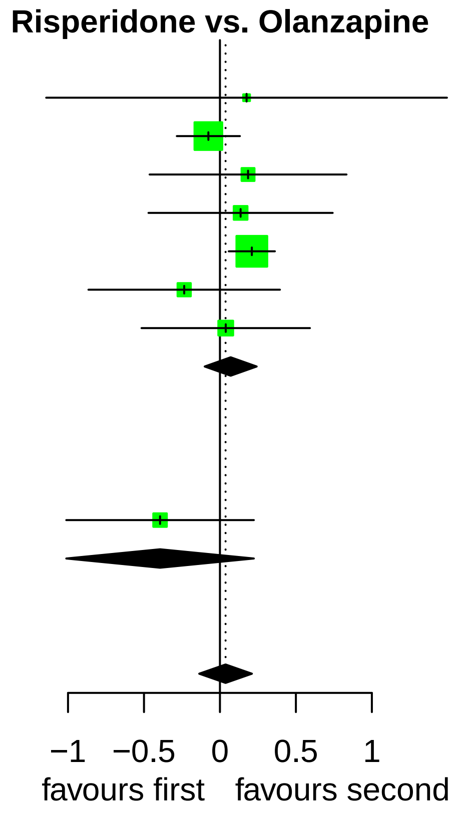

Study or Subgroup	first drug Mean	SD	Total	second drug Mean	SD	Total	Weight	SMD [95% CI]
**blinding = 0**								
Ehrlich 2012	13.63	2.1000	8	14.21	3.1700	14	3.6%	−0.20 [−1.07; 0.67]
**Total (95% CI)**			**8**			**14**	**3.6%**	**−0.20 [−1.07; 0.67]**
Heterogeneity: not applicable								
**blinding = 1**								
Grootens 2009	−5.62	4.9700	34	−6.70	4.5400	33	12.0%	0.22 [−0.26; 0.70]
Lieberman 2005 12weeks	−0.88	4.7105	184	−2.15	4.7105	331	84.4%	0.27 [0.09; 0.45]
**Total (95% CI)**			**218**			**364**	**96.4%**	**0.26 [0.09; 0.43]**
Heterogeneity: $Tau^2 = 0$; $Chi^2 = 0.03$, df = 1 (P = 0.86); $I^2 = 0\%$								
**Total (95% CI)**			**226**			**378**	**100.0%**	**0.25 [0.08; 0.41]**

Heterogeneity: $Tau^2 = 0$; $Chi^2 = 1.06$, df = 2 (P = 0.59); $I^2 = 0\%$

Test for subgroup differences: $Chi^2 = 1.03$, df = 1 (P = 0.31)

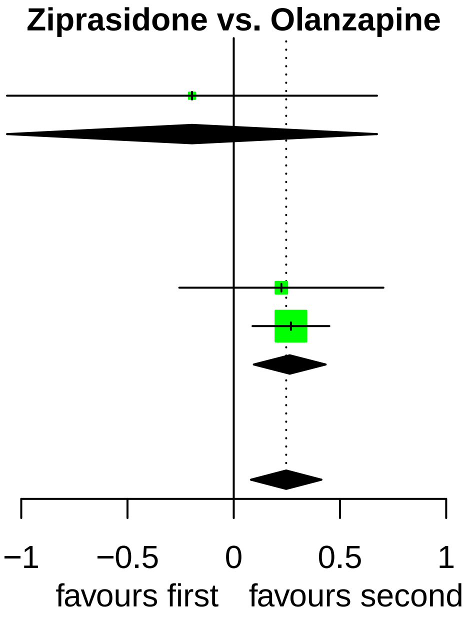

Study or Subgroup	first drug Mean	first drug SD	first drug Total	second drug Mean	second drug SD	second drug Total	Weight	SMD [95% CI]
**blinding = 1**								
Addington 2004	−5.50	4.4282	125	−6.00	4.4282	132	25.9%	0.11 [−0.13; 0.36]
Lieberman 2005 12weeks	−0.88	4.7105	184	−1.16	4.7105	339	48.1%	0.06 [−0.12; 0.24]
Zhang 2011	−10.20	6.3004	118	−11.50	6.2700	121	24.0%	0.21 [−0.05; 0.46]
**Total (95% CI)**			**427**			**592**	**98.0%**	**0.11 [−0.02; 0.24]**
Heterogeneity: $Tau^2 = 0$; $Chi^2 = 0.86$, df = 2 (P = 0.65); $I^2 = 0\%$								
**blinding = 0**								
Sonmez 2009	12.30	3.8000	10	12.60	5.6800	10	2.0%	−0.06 [−0.94; 0.82]
**Total (95% CI)**			**10**			**10**	**2.0%**	**−0.06 [−0.94; 0.82]**
Heterogeneity: not applicable								
**Total (95% CI)**			**437**			**602**	**100.0%**	**0.11 [−0.02; 0.23]**

Heterogeneity: $Tau^2 = 0$; $Chi^2 = 1.00$, df = 3 (P = 0.80); $I^2 = 0\%$

Test for subgroup differences: $Chi^2 = 0.14$, df = 1 (P = 0.71)

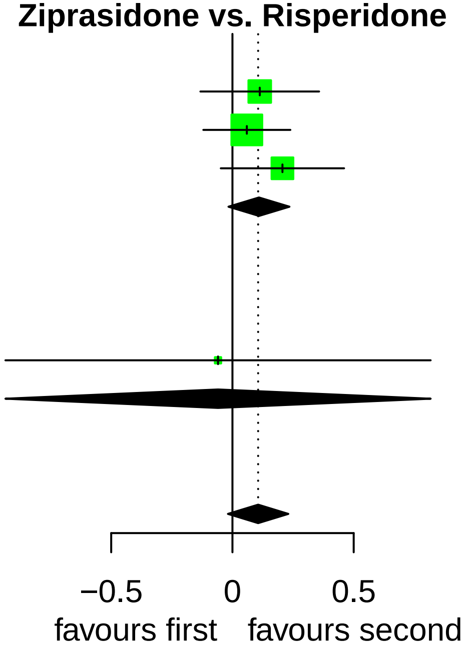

eFigure 5b positive symptoms difference between blinded and open trials, more recent drug listed first

Study	TE	SE	Weight	SMD [95% CI]
Ziprasidone vs. Olanzapine, (nbl=582, nop=22)	0.46	0.4527	6.0%	0.46 [−0.43; 1.35]
Aripiprazole vs. Haloperidol, (nbl=416, nop=97)	−0.24	0.2415	21.1%	−0.24 [−0.71; 0.24]
Aripiprazole vs. Risperidone, (nbl=413, nop=76)	0.33	0.2554	18.9%	0.33 [−0.17; 0.83]
Olanzapine vs. Risperidone, (nbl=1203, nop=42)	−0.46	0.3269	11.5%	−0.46 [−1.11; 0.18]
Quetiapine vs. Haloperidol, (nbl=778, nop=79)	−0.02	0.2461	20.3%	−0.02 [−0.50; 0.46]
Olanzapine vs. Haloperidol, (nbl=2599, nop=23)	−0.22	0.4553	5.9%	−0.22 [−1.11; 0.68]
Ziprasidone vs. Risperidone, (nbl=1019, nop=20)	0.17	0.4519	6.0%	0.17 [−0.72; 1.05]
Risperidone vs. Haloperidol, (nbl=1224, nop=36)	−0.20	0.3457	10.3%	−0.20 [−0.88; 0.48]
**Total (95% CI)**			**100.0%**	**−0.04 [−0.26; 0.18]**

Heterogeneity: $Tau^2 = 0.0002$; $Chi^2 = 6.24$, df = 7 (P = 0.51); $I^2 = 0\%$

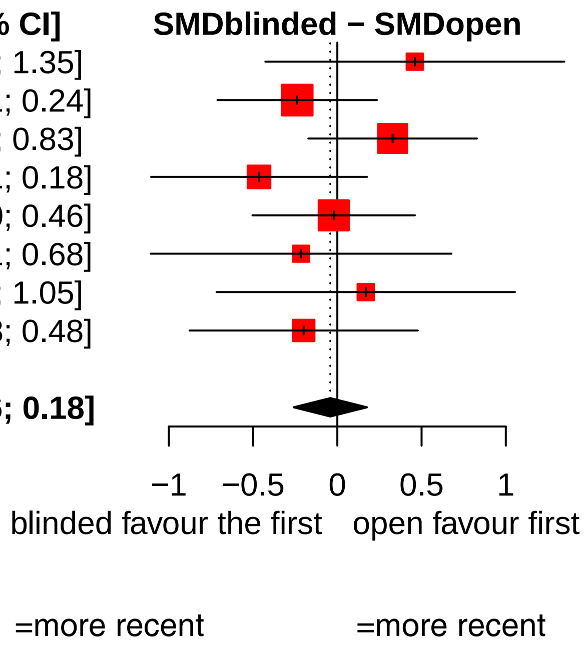

eFigure 5c positive symptoms difference between blinded and open trials, less efficacious drug according to Huhn et al. 2019 listed first

Study	TE	SE	Weight	SMD [95% CI]
Ziprasidone vs. Olanzapine, (nbl=582, nop=22)	0.46	0.4527	6.0%	0.46 [−0.43; 1.35]
Aripiprazole vs. Haloperidol, (nbl=416, nop=97)	−0.24	0.2415	21.1%	−0.24 [−0.71; 0.24]
Aripiprazole vs. Risperidone, (nbl=413, nop=76)	0.33	0.2554	18.9%	0.33 [−0.17; 0.83]
Risperidone vs. Olanzapine, (nbl=1203, nop=42)	0.46	0.3269	11.5%	0.46 [−0.18; 1.11]
Quetiapine vs. Haloperidol, (nbl=778, nop=79)	−0.02	0.2461	20.3%	−0.02 [−0.50; 0.46]
Haloperidol vs. Olanzapine, (nbl=2599, nop=23)	0.22	0.4553	5.9%	0.22 [−0.68; 1.11]
Ziprasidone vs. Risperidone, (nbl=1019, nop=20)	0.17	0.4519	6.0%	0.17 [−0.72; 1.05]
Haloperidol vs. Risperidone, (nbl=1224, nop=36)	0.20	0.3457	10.3%	0.20 [−0.48; 0.88]
**Total (95% CI)**			**100.0%**	**0.13 [−0.09; 0.35]**

Heterogeneity: $Tau^2 = 0$; $Chi^2 = 4.97$, df = 7 (P = 0.66); $I^2 = 0\%$

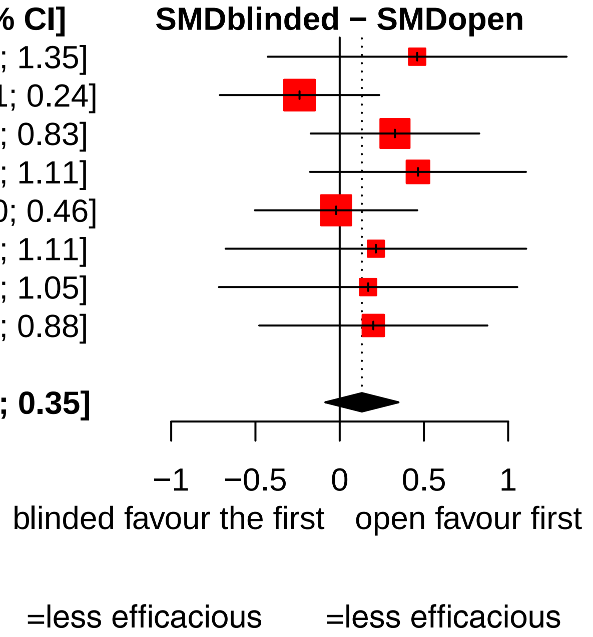

eFigure 5d positive symptoms sponsored vs non sponsored drugs and blinded versus open trials

Study or Subgroup	TE	SE	Weight	SMD [95% CI]
**blinding = 1**				
Addington 2004	−0.11	0.1249	1.7%	−0.11 [−0.36; 0.13]
Arvanitis 1997	−0.06	0.1721	1.3%	−0.06 [−0.40; 0.27]
Azorin 2006	0.11	0.1528	1.4%	0.11 [−0.19; 0.41]
Beasley 1996b	−0.01	0.1501	1.5%	−0.01 [−0.30; 0.28]
Beasley 1997	0.09	0.1365	1.6%	0.09 [−0.18; 0.35]
Bernardo 2001	−0.33	0.3877	0.4%	−0.33 [−1.09; 0.43]
Blin 1996	0.65	0.2746	0.7%	0.65 [0.11; 1.19]
Canive 2006	0.20	0.6724	0.2%	0.20 [−1.12; 1.52]
Chan 2007	−0.36	0.2249	0.9%	−0.36 [−0.80; 0.09]
Chouinard 1993	0.40	0.3082	0.6%	0.40 [−0.20; 1.01]
Claus 1992	−0.08	0.3087	0.6%	−0.08 [−0.69; 0.52]
Conley 2001	0.08	0.1061	1.9%	0.08 [−0.13; 0.28]
Copolov 2000	−0.30	0.0962	2.0%	−0.30 [−0.49; −0.11]
Cutler 2008	−0.00	0.1024	1.9%	−0.00 [−0.20; 0.20]
Davidson 2007	−0.01	0.1268	1.7%	−0.01 [−0.26; 0.23]
Durgam 2014	−0.39	0.0987	2.0%	−0.39 [−0.59; −0.20]
Gattaz 2004	−0.11	0.4006	0.4%	−0.11 [−0.89; 0.68]
Goff 1998	0.09	0.3301	0.5%	0.09 [−0.56; 0.74]
Grootens 2009	−0.23	0.2451	0.8%	−0.23 [−0.71; 0.25]
Hale 2000	−0.16	0.1110	1.9%	−0.16 [−0.38; 0.05]
Hoyberg 1993	0.39	0.2009	1.1%	0.39 [−0.01; 0.78]
Huttunen 1995	0.18	0.2025	1.1%	0.18 [−0.21; 0.58]
Hwang 2001	−0.29	0.2403	0.9%	−0.29 [−0.76; 0.19]
Hwang 2003	−0.25	0.2934	0.6%	−0.25 [−0.82; 0.33]
Ishigooka 2001	0.20	0.1595	1.4%	0.20 [−0.12; 0.51]
Janicak 2001	0.13	0.2544	0.8%	0.13 [−0.37; 0.63]
Janssen CR012625	0.04	0.1709	1.3%	0.04 [−0.30; 0.37]
Kane 2002	−0.05	0.1205	1.8%	−0.05 [−0.29; 0.18]
Kane 2007b	0.13	0.1025	1.9%	0.13 [−0.07; 0.33]
Kane 2009 8 weeks	0.18	0.0842	2.1%	0.18 [0.01; 0.34]
Kane 2010a	0.03	0.1166	1.8%	0.03 [−0.20; 0.26]
Kluge 2007	−0.39	0.3686	0.4%	−0.39 [−1.11; 0.33]
Li 2012	−0.18	0.1837	1.2%	−0.18 [−0.54; 0.18]
Lundbeck NCT00864045	0.04	0.1014	2.0%	0.04 [−0.16; 0.24]
Marder 1994	0.35	0.1788	1.2%	0.35 [0.00; 0.70]
Marder 2007c	−0.11	0.1185	1.8%	−0.11 [−0.34; 0.12]
Martin 2002	0.12	0.1038	1.9%	0.12 [−0.08; 0.33]
Min 1993	0.32	0.3415	0.5%	0.32 [−0.34; 0.99]
Möller 1997	0.11	0.1460	1.5%	0.11 [−0.17; 0.40]
Murasaki 2001	−0.05	0.1464	1.5%	−0.05 [−0.34; 0.24]
NCT00882518	−0.17	0.1022	1.9%	−0.17 [−0.37; 0.03]
NCT01104766	−0.01	0.0997	2.0%	−0.01 [−0.20; 0.19]
NCT01617187	−0.05	0.1767	1.2%	−0.05 [−0.40; 0.30]
NCT01810380	−0.18	0.1157	1.8%	−0.18 [−0.41; 0.05]
Peuskens 1995	0.07	0.0818	2.2%	0.07 [−0.09; 0.23]
Peuskens 1999	0.13	0.1326	1.6%	0.13 [−0.13; 0.39]
Potkin 2003	−0.12	0.1253	1.7%	−0.12 [−0.36; 0.13]
Potkin 2007c	0.05	0.1874	1.2%	0.05 [−0.32; 0.42]
Puech 1998	0.34	0.1572	1.4%	0.34 [0.04; 0.65]
Sacchetti 2008	−0.08	0.2450	0.8%	−0.08 [−0.56; 0.40]
Schennach 2018	0.68	0.1945	1.1%	0.68 [0.30; 1.06]
Shah 2011	0.15	0.1396	1.6%	0.15 [−0.12; 0.43]
Study 049	−0.37	0.1471	1.5%	−0.37 [−0.66; −0.08]
Study 231	−0.22	0.1121	1.8%	−0.22 [−0.44; 0.00]
Study 233	−0.20	0.1129	1.8%	−0.20 [−0.42; 0.03]
Study 3000	−0.20	0.1325	1.6%	−0.20 [−0.46; 0.06]
Study 3004	−0.20	0.1167	1.8%	−0.20 [−0.43; 0.03]
Study 3005	−0.34	0.0978	2.0%	−0.34 [−0.53; −0.15]
Study 94202 2002	−0.11	0.1926	1.1%	−0.11 [−0.49; 0.27]
Tollefson 1997	0.14	0.0484	2.5%	0.14 [0.04; 0.23]
Wagner 2005	−0.31	0.3353	0.5%	−0.31 [−0.97; 0.35]
Wetzel 1998	0.39	0.1793	1.2%	0.39 [0.04; 0.74]
Zborowski 1995	−0.36	0.1347	1.6%	−0.36 [−0.63; −0.10]
Zhang 2011	−0.21	0.1297	1.7%	−0.21 [−0.46; 0.05]
Zhong 2006	−0.15	0.0788	2.2%	−0.15 [−0.31; 0.00]
Zimbroff 1997	−0.18	0.1224	1.7%	−0.18 [−0.42; 0.06]
Zimbroff 2007	−0.27	0.1279	1.7%	−0.27 [−0.52; −0.02]
**Total (95% CI)**			**94.3%**	**−0.03 [−0.09; 0.02]**
Heterogeneity: $Tau^2 = 0.0227$; $Chi^2 = 152.87$, df = 66 ($P < 0.01$); $I^2 = 57\%$				
**blinding = 0**				
Avasthi 2001	−0.10	0.4534	0.3%	−0.10 [−0.99; 0.79]
de Boer 2011	0.04	0.3543	0.5%	0.04 [−0.65; 0.73]
de Oliviera 2009	−0.31	0.2189	1.0%	−0.31 [−0.74; 0.12]
Dossenbach 2007	0.76	0.2050	1.1%	0.76 [0.36; 1.16]
Ehrlich 2012	0.20	0.4443	0.3%	0.20 [−0.67; 1.07]
Loza 1999	0.59	0.3358	0.5%	0.59 [−0.06; 1.25]
Maat 2014	0.20	0.3103	0.6%	0.20 [−0.41; 0.80]
Taneli 2003	−0.20	0.2278	0.9%	−0.20 [−0.64; 0.25]
van Bruggen 2003	−0.40	0.3149	0.6%	−0.40 [−1.02; 0.22]
**Total (95% CI)**			**5.7%**	**0.09 [−0.21; 0.39]**
Heterogeneity: $Tau^2 = 0.1169$; $Chi^2 = 20.7$, df = 8 ($P < 0.01$); $I^2 = 61\%$				
**Total (95% CI)**			**100.0%**	**−0.03 [−0.08; 0.03]**

Heterogeneity: $Tau^2 = 0.0267$; $Chi^2 = 175.70$, df = 75 ($P < 0.01$); $I^2 = 57\%$
Test for subgroup differences: $Chi^2 = 0.66$, df = 1 ($P = 0.42$)

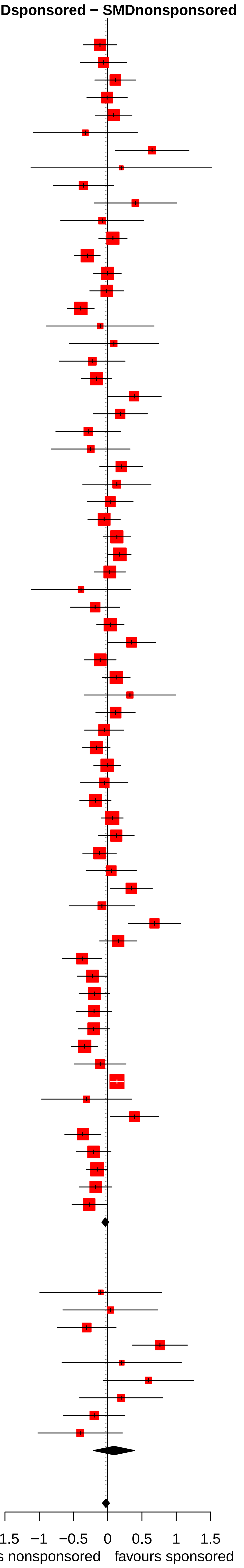

eFigure 6

Negative symptoms

1. Results of individual comparisons
2. Difference between blinded and open RCTs by recency
3. Difference between blinded and open RCTs by efficacy
4. Differences between blinded and open RCTs sponsored versus non-sponsored drugs

eFigure 6a Negative symptoms individual comparisons

Study or Subgroup	first drug Mean	SD	Total	second drug Mean	SD	Total	Weight	SMD [95% CI]
**blinding = 1**								
Chan 2007	−4.80	5.1000	49	−4.00	5.7000	34	17.8%	−0.15 [−0.59; 0.29]
Hatta 2009	−3.80	6.7496	21	−3.30	6.7496	20	9.1%	−0.07 [−0.69; 0.54]
Potkin 2003	−3.40	6.2937	194	−3.10	6.2937	95	56.7%	−0.05 [−0.29; 0.20]
**Total (95% CI)**			**264**			**149**	**83.7%**	**−0.07 [−0.27; 0.13]**
Heterogeneity: $Tau^2 = 0$; $Chi^2 = 0.15$, df = 2 (P = 0.93); $I^2 = 0\%$								
**blinding = 0**								
de Boer 2011	−1.30	14.9000	17	0.40	15.1000	15	7.1%	−0.11 [−0.81; 0.58]
Maat 2014	−2.35	5.5300	17	−1.26	7.2000	27	9.3%	−0.16 [−0.77; 0.45]
**Total (95% CI)**			**34**			**42**	**16.3%**	**−0.14 [−0.60; 0.32]**
Heterogeneity: $Tau^2 = 0$; $Chi^2 = 0.01$, df = 1 (P = 0.91); $I^2 = 0\%$								
**Total (95% CI)**			**298**			**191**	**100.0%**	**−0.08 [−0.27; 0.10]**

Heterogeneity: $Tau^2 = 0$; $Chi^2 = 0.24$, df = 4 (P = 0.99); $I^2 = 0\%$

Test for subgroup differences: $Chi^2 = 0.07$, df = 1 (P = 0.79)

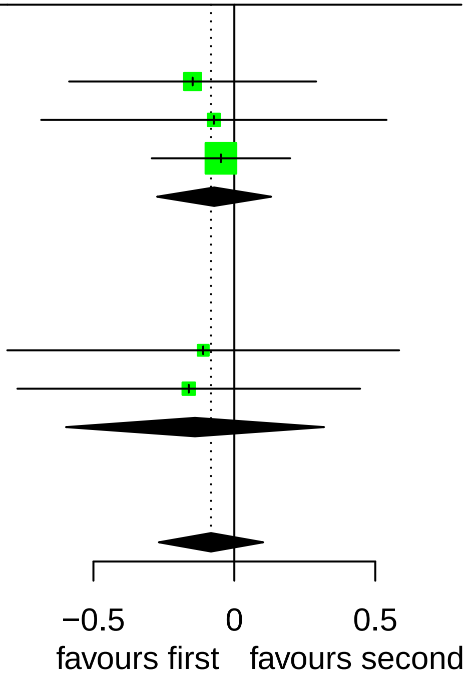

Study or Subgroup	first drug Mean	SD	Total	second drug Mean	SD	Total	Weight	SMD [95% CI]
**blinding = 0**								
de Oliviera 2009	21.00	6.2000	31	20.40	7.2000	66	15.9%	0.09 [−0.34; 0.51]
**blinding = 1**								
Kane 2002	−2.90	6.1831	104	−2.95	6.1831	199	51.5%	0.01 [−0.23; 0.25]
Study 93202 2002	−3.40	4.3459	33	−2.80	4.3459	33	12.4%	−0.14 [−0.62; 0.35]
Study 94202 2002	−2.00	6.1624	54	−3.65	6.1624	54	20.2%	0.27 [−0.11; 0.64]
**Total (95% CI)**			**191**			**286**	**84.1%**	**0.05 [−0.14; 0.23]**
Heterogeneity: Tau^2 = < 0.0001; Chi^2 = 1.94, df = 2 (P = 0.38); I^2 = 0%								
**Total (95% CI)**			**222**			**352**	**100.0%**	**0.05 [−0.12; 0.22]**

Heterogeneity: Tau^2 = 0; Chi^2 = 1.96, df = 3 (P = 0.58); I^2 = 0%

Test for subgroup differences: Chi^2 = 0.03, df = 1 (P = 0.87)

Haloperidol vs. Aripiprazole

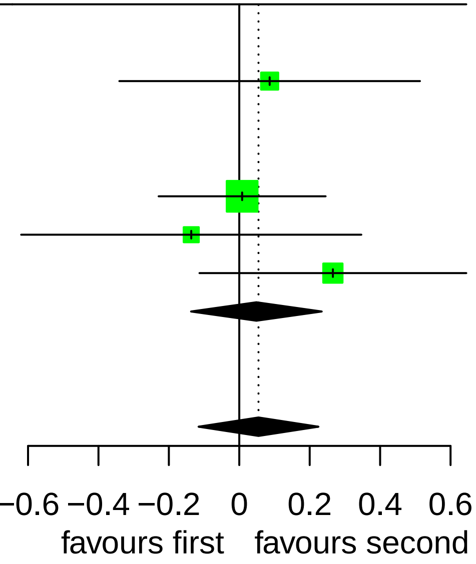

Study or Subgroup	first drug Mean	SD	Total	second drug Mean	SD	Total	Weight	SMD [95% CI]
**blinding = 1**								
Allan 1998	23.80	6.6000	10	22.90	7.8000	13	1.0%	0.12 [−0.71; 0.94]
Beasley 1996b	−6.60	16.9035	68	−9.83	16.9035	127	7.6%	0.19 [−0.10; 0.49]
Beasley 1997	−4.80	6.3000	79	−6.01	7.4900	168	9.2%	0.17 [−0.10; 0.44]
Bernardo 2001	18.00	5.6000	13	17.80	5.5000	14	1.2%	0.03 [−0.72; 0.79]
Ishigooka 2001	−2.94	5.6500	78	−3.76	4.6500	80	6.8%	0.16 [−0.15; 0.47]
Tollefson 1997	−3.20	6.1000	636	−4.50	6.3000	1312	73.4%	0.21 [0.11; 0.30]
**Total (95% CI)**			**884**			**1714**	**99.2%**	**0.20 [0.12; 0.28]**
Heterogeneity: $Tau^2 = 0$; $Chi^2 = 0.37$, df = 5 (P = 1.00); $I^2 = 0\%$								
**blinding = 0**								
Avasthi 2001	16.86	8.7100	7	15.62	7.9300	16	0.8%	0.15 [−0.74; 1.04]
**Total (95% CI)**			**891**			**1730**	**100.0%**	**0.20 [0.12; 0.28]**

Heterogeneity: $Tau^2 = 0$; $Chi^2 = 0.38$, df = 6 (P = 1.00); $I^2 = 0\%$

Test for subgroup differences: $Chi^2 = 0.01$, df = 1 (P = 0.91)

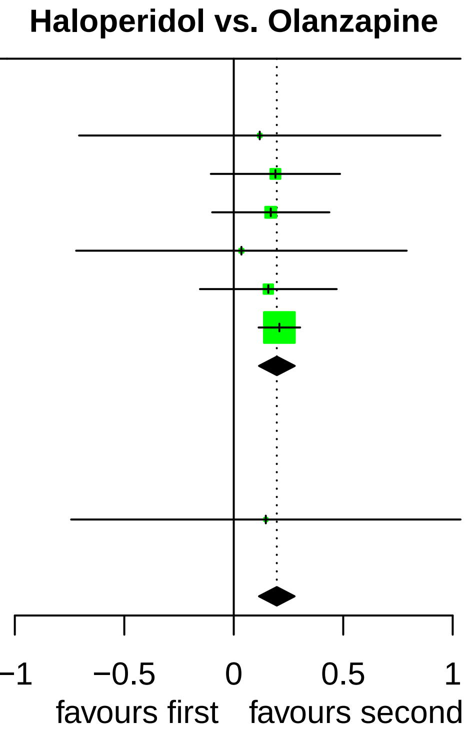

Study or Subgroup	first drug Mean	SD	Total	second drug Mean	SD	Total	Weight	SMD [95% CI]
**blinding = 1**								
Arvanitis 1997	−1.83	3.6945	50	−0.74	3.6945	104	16.1%	−0.29 [−0.63; 0.05]
Copolov 2000	−5.30	7.7000	219	−4.90	7.5000	216	52.2%	−0.05 [−0.24; 0.14]
Murasaki 2001	22.60	7.8000	90	23.00	8.2000	97	22.4%	−0.05 [−0.34; 0.24]
**Total (95% CI)**			**359**			**417**	**90.7%**	**−0.09 [−0.24; 0.05]**
Heterogeneity: $Tau^2 = 0$; $Chi^2 = 1.61$, df = 2 (P = 0.45); $I^2 = 0\%$								
**blinding = 0**								
Taneli 2003	19.40	8.7000	34	20.40	9.0000	45	9.3%	−0.11 [−0.56; 0.33]
**Total (95% CI)**			**393**			**462**	**100.0%**	**−0.10 [−0.23; 0.04]**

Heterogeneity: $Tau^2 = 0$; $Chi^2 = 1.62$, df = 3 (P = 0.66); $I^2 = 0\%$

Test for subgroup differences: $Chi^2 = 0.01$, df = 1 (P = 0.94)

Haloperidol vs. Quetiapine

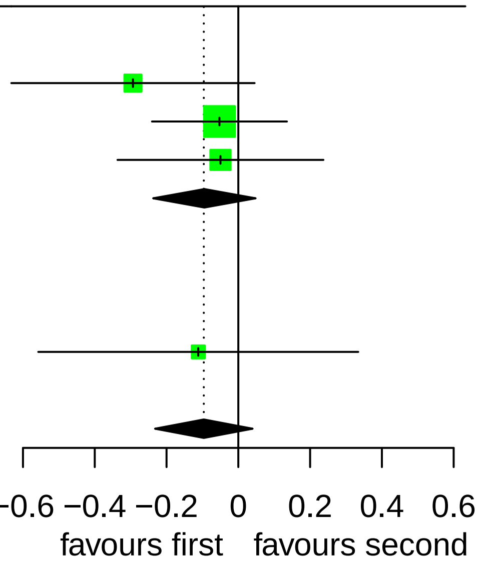

Study or Subgroup	first drug Mean	first drug SD	first drug Total	second drug Mean	second drug SD	second drug Total	Weight	SMD [95% CI]
**blinding = 1**								
Abdolahian 2008	22.10	3.3400	30	19.20	3.3400	35	6.5%	0.86 [0.35; 1.37]
Blin 1996	−3.30	8.3748	20	−6.80	8.3748	21	5.1%	0.41 [−0.21; 1.03]
Borison 1992	−8.50	15.4700	53	−9.80	15.4700	53	8.8%	0.08 [−0.30; 0.46]
Ceskova 1993	1.76	0.5568	31	1.93	0.6125	31	6.6%	−0.29 [−0.79; 0.21]
Chouinard 1993	−2.00	7.0434	21	−5.80	7.0434	22	5.2%	0.53 [−0.08; 1.14]
Claus 1992	22.00	7.7900	21	25.20	8.2500	21	5.2%	−0.39 [−1.00; 0.22]
Ghaleiha 2011	13.37	10.9496	16	12.81	10.9496	16	4.3%	0.05 [−0.64; 0.74]
Janicak 2001	−2.20	5.8000	32	−3.50	5.8900	30	6.6%	0.22 [−0.28; 0.72]
Liu 2000	−5.40	8.0000	19	−5.40	8.0000	19	4.9%	0.00 [−0.64; 0.64]
Marder 1994	−0.30	6.7053	64	−3.30	6.7053	63	9.3%	0.44 [0.09; 0.80]
Min 1993	−7.40	6.4846	19	−4.50	6.4846	16	4.5%	−0.44 [−1.11; 0.24]
Peuskens 1995	−4.80	6.8700	223	−5.35	7.5500	455	13.8%	0.07 [−0.09; 0.24]
See 1999	21.90	5.8500	10	19.20	4.9600	10	2.9%	0.48 [−0.41; 1.37]
Yen 2004	−4.10	3.4000	20	−6.70	4.0000	21	4.9%	0.69 [0.05; 1.32]
**Total (95% CI)**			**579**			**813**	**88.6%**	**0.19 [−0.00; 0.38]**
Heterogeneity: $Tau^2 = 0.0619$; $Chi^2 = 25.69$, df = 13 (P = 0.02); $I^2 = 49\%$								
**blinding = 0**								
Mirabzadeh 2014	7.38	3.2000	29	6.17	2.9000	37	6.8%	0.39 [−0.10; 0.88]
Tamrakar 2006	−13.00	9.1100	18	−17.06	14.1700	18	4.7%	0.33 [−0.33; 0.99]
**Total (95% CI)**			**47**			**55**	**11.4%**	**0.37 [−0.02; 0.77]**
Heterogeneity: $Tau^2 = 0$; $Chi^2 = 0.02$, df = 1 (P = 0.88); $I^2 = 0\%$								
**Total (95% CI)**			**626**			**868**	**100.0%**	**0.21 [0.04; 0.38]**

Heterogeneity: $Tau^2 = 0.0476$; $Chi^2 = 26.80$, df = 15 (P = 0.03); $I^2 = 44\%$

Test for subgroup differences: $Chi^2 = 0.66$, df = 1 (P = 0.42)

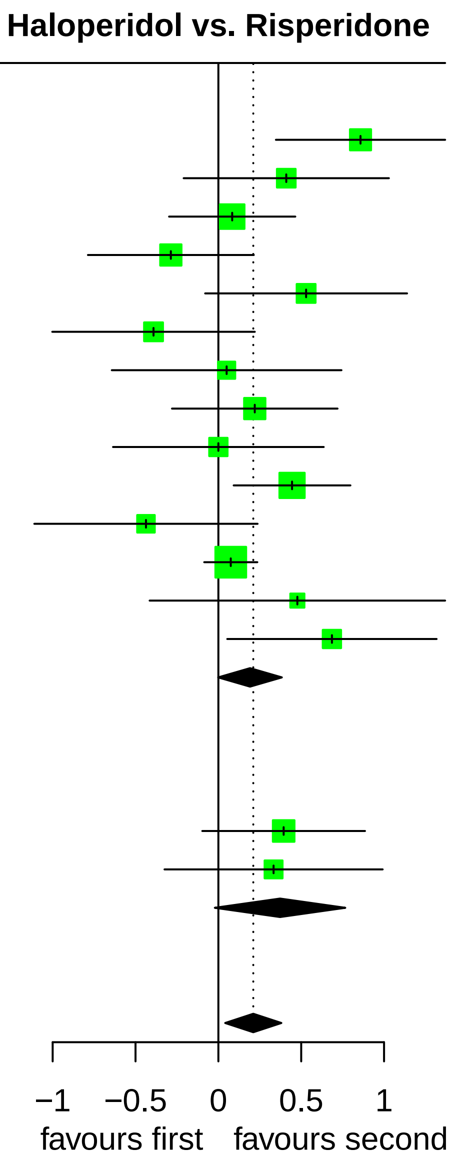

Study or Subgroup	first drug Mean	SD	Total	second drug Mean	SD	Total	Weight	SMD [95% CI]
**blinding = 1**								
Canive 2006	21.25	7.2365	4	15.90	7.2365	5	0.7%	0.66 [−0.72; 2.03]
Conley 2001	−2.90	5.9000	175	−2.90	6.0000	181	29.0%	0.00 [−0.21; 0.21]
Hatta 2009	−3.30	6.7496	20	−5.60	6.7496	17	3.0%	0.33 [−0.32; 0.98]
Hatta 2013	17.20	6.9109	20	17.82	8.8350	22	3.5%	−0.08 [−0.68; 0.53]
Lieberman 2005 12weeks	−0.50	4.6598	339	−1.26	4.6598	331	53.1%	0.16 [0.01; 0.31]
Mori 2004	25.60	4.2755	19	22.80	4.2755	20	3.1%	0.64 [−0.00; 1.29]
Sacchetti 2008	−5.62	5.7578	25	−5.72	5.7578	25	4.2%	0.02 [−0.54; 0.57]
**Total (95% CI)**			**602**			**601**	**96.6%**	**0.12 [0.00; 0.24]**
Heterogeneity: $Tau^2 = 0.0012$; $Chi^2 = 5.63$, df = 6 (P = 0.47); $I^2 = 0\%$								
**blinding = 0**								
van Bruggen 2003	−2.40	6.8600	24	−4.30	9.3300	18	3.4%	0.23 [−0.38; 0.85]
**Total (95% CI)**			**626**			**619**	**100.0%**	**0.13 [0.01; 0.24]**
Heterogeneity: $Tau^2 = 0.0003$; $Chi^2 = 5.75$, df = 7 (P = 0.57); $I^2 = 0\%$								
Test for subgroup differences: $Chi^2 = 0.12$, df = 1 (P = 0.73)								

Risperidone vs. Olanzapine

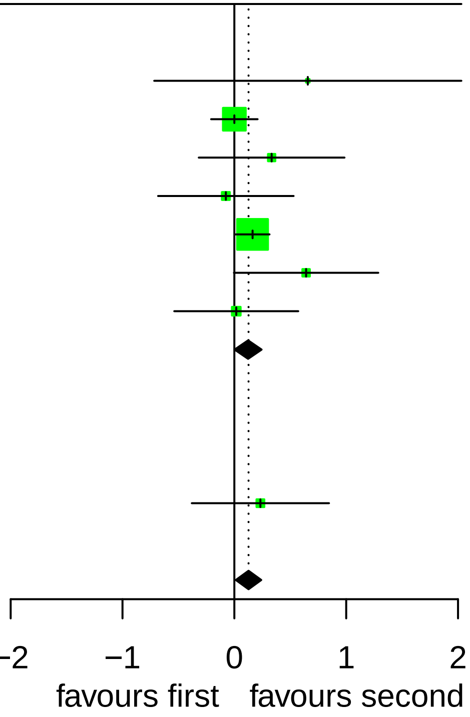

Study or	first drug			second drug					Ziprasidone vs. Olanzapine
Subgroup	Mean	SD	Total	Mean	SD	Total	Weight	SMD [95% CI]	
**blinding = 0**									
Ehrlich 2012	18.25	3.1500	8	18.14	5.3000	14	3.6%	0.02 [−0.85; 0.89]	
**blinding = 1**									
Grootens 2009	−2.38	4.7900	34	−2.76	4.1500	33	11.9%	0.08 [−0.40; 0.56]	
Lieberman 2005 12weeks	−1.20	4.6598	184	−1.26	4.6598	331	84.4%	0.01 [−0.17; 0.19]	
**Total (95% CI)**			**218**			**364**	**96.4%**	**0.02 [−0.15; 0.19]**	
Heterogeneity: $Tau^2 = 0$; $Chi^2 = 0.07$, df = 1 (P = 0.79); $I^2 = 0\%$									
**Total (95% CI)**			**226**			**378**	**100.0%**	**0.02 [−0.14; 0.19]**	

Heterogeneity: $Tau^2 = 0$; $Chi^2 = 0.07$, df = 2 (P = 0.96); $I^2 = 0\%$

Test for subgroup differences: $Chi^2 = 0.00$, df = 1 (P = 1.00)

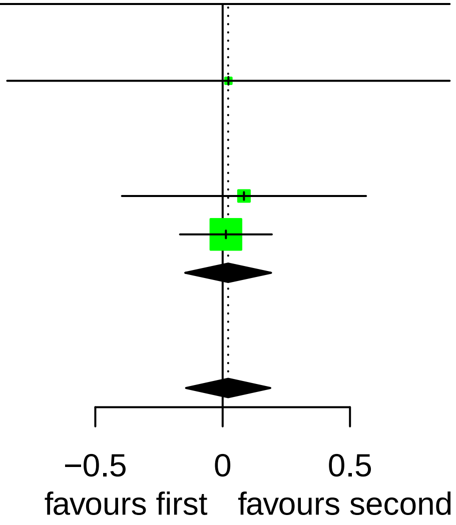

Study or Subgroup	first drug Mean	first drug SD	first drug Total	second drug Mean	second drug SD	second drug Total	Weight	SMD [95% CI]
**blinding = 1**								
Addington 2004	−6.40	5.7578	123	−6.40	5.7578	132	28.5%	0.00 [−0.25; 0.25]
Lieberman 2005 12weeks	−1.20	4.6598	184	−0.50	4.6598	339	40.8%	−0.15 [−0.33; 0.03]
Zhang 2011	−7.00	5.7573	118	−7.80	5.7200	121	27.3%	0.14 [−0.11; 0.39]
**Total (95% CI)**			**425**			**592**	**96.7%**	**−0.02 [−0.19; 0.15]**
Heterogeneity: $Tau^2 = 0.0098$; $Chi^2 = 3.45$, df = 2 (P = 0.18); $I^2 = 42\%$								
**blinding = 0**								
Sonmez 2009	18.00	6.1500	10	17.80	7.5500	10	3.3%	0.03 [−0.85; 0.90]
**Total (95% CI)**			**435**			**602**	**100.0%**	**−0.02 [−0.19; 0.14]**

Heterogeneity: $Tau^2 = 0.0086$; $Chi^2 = 3.47$, df = 3 (P = 0.32); $I^2 = 14\%$

Test for subgroup differences: $Chi^2 = 0.01$, df = 1 (P = 0.91)

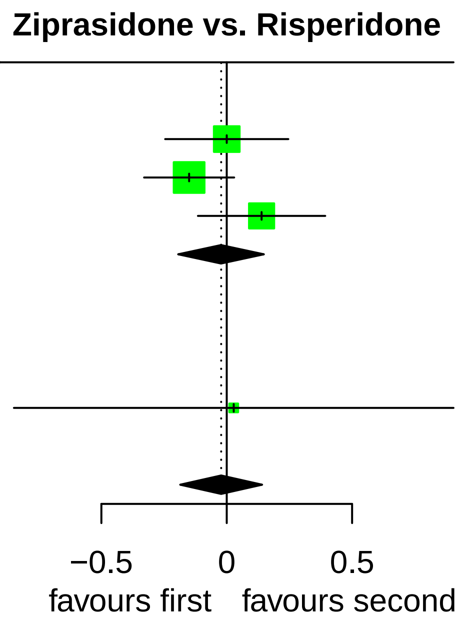

eFigure 6b negative symptoms difference between blinded and open trials, more recent drug first

Study	SMD	SE	Weight	SMD [95% CI]
Ziprasidone vs. Olanzapine, (nbl=582, nop=22)	−0.0011	0.4515	5.2%	−0.00 [−0.89; 0.88]
Haloperidol vs. Aripiprazole, (nbl=477, nop=97)	−0.0377	0.2375	18.7%	−0.04 [−0.50; 0.43]
Aripiprazole vs. Risperidone, (nbl=413, nop=76)	0.0679	0.2552	16.2%	0.07 [−0.43; 0.57]
Risperidone vs. Olanzapine, (nbl=1203, nop=42)	−0.1099	0.3189	10.3%	−0.11 [−0.74; 0.52]
Haloperidol vs. Quetiapine, (nbl=776, nop=79)	0.0171	0.2388	18.5%	0.02 [−0.45; 0.49]
Haloperidol vs. Olanzapine, (nbl=2598, nop=23)	0.0506	0.4557	5.1%	0.05 [−0.84; 0.94]
Ziprasidone vs. Risperidone, (nbl=1017, nop=20)	−0.0506	0.4557	5.1%	−0.05 [−0.94; 0.84]
Haloperidol vs. Risperidone, (nbl=1392, nop=102)	−0.1811	0.2235	21.1%	−0.18 [−0.62; 0.26]
**Total (95% CI)**			**100.0%**	**−0.04 [−0.24; 0.16]**

Heterogeneity: $Tau^2 = 0$; $Chi^2 = 0.73$, df = 7 (P = 1.00); $I^2 = 0\%$

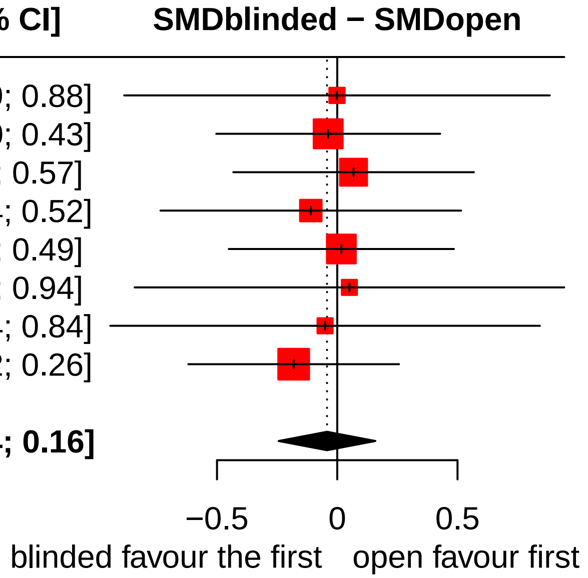

Figure 6c negative symptoms - difference between blinded and open RCTs less efficacious drug listed first

Study	SMD	SE	Weight	SMD [95% CI]
Ziprasidone vs. Olanzapine, (nbl=582, nop=22)	−0.0011	0.4515	5.2%	−0.00 [−0.89; 0.88]
Aripiprazole vs. Haloperidol, (nbl=477, nop=97)	0.0377	0.2375	18.7%	0.04 [−0.43; 0.50]
Aripiprazole vs. Risperidone, (nbl=413, nop=76)	0.0679	0.2552	16.2%	0.07 [−0.43; 0.57]
Olanzapine vs. Risperidone, (nbl=1203, nop=42)	0.1099	0.3189	10.3%	0.11 [−0.52; 0.74]
Quetiapine vs. Haloperidol, (nbl=776, nop=79)	−0.0171	0.2388	18.5%	−0.02 [−0.49; 0.45]
Olanzapine vs. Haloperidol, (nbl=2598, nop=23)	−0.0506	0.4557	5.1%	−0.05 [−0.94; 0.84]
Ziprasidone vs. Risperidone, (nbl=1017, nop=20)	−0.0506	0.4557	5.1%	−0.05 [−0.94; 0.84]
Risperidone vs. Haloperidol, (nbl=1392, nop=102)	0.1811	0.2235	21.1%	0.18 [−0.26; 0.62]
**Total (95% CI)**			**100.0%**	**0.06 [−0.14; 0.26]**

Heterogeneity: $Tau^2 = 0$; $Chi^2 = 0.57$, df = 7 (P = 1.00); $I^2 = 0\%$

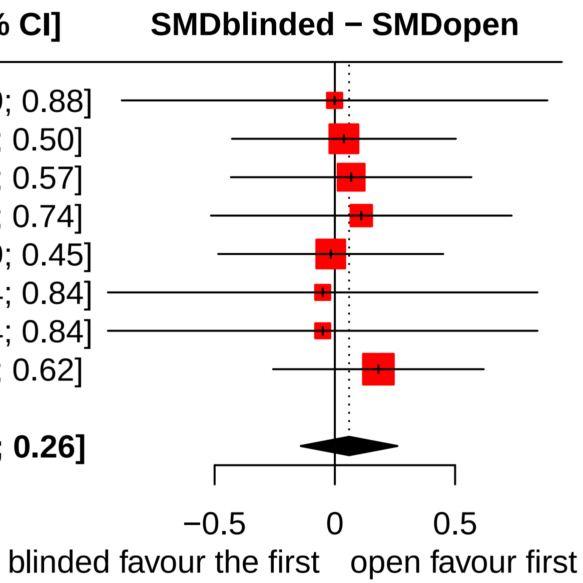

Figure 6d negative symptoms - sponsored versus non-sponsored drugs and blinded versus open trials

Study or Subgroup	SMD	SE	Weight	SMD [95% CI]
**blinding = 1**				
Addington 2004	0.0000	0.1253	1.6%	0.00 [−0.25; 0.25]
Arvanitis 1997	−0.2936	0.1729	1.1%	−0.29 [−0.63; 0.05]
Azorin 2006	0.2134	0.1531	1.3%	0.21 [−0.09; 0.51]
Beasley 1996b	0.1934	0.1506	1.3%	0.19 [−0.10; 0.49]
Beasley 1997	0.1696	0.1366	1.5%	0.17 [−0.10; 0.44]
Bernardo 2001	0.0360	0.3852	0.3%	0.04 [−0.72; 0.79]
Blin 1996	0.4276	0.2711	0.5%	0.43 [−0.10; 0.96]
Brook 2005	0.2565	0.0982	2.1%	0.26 [0.06; 0.45]
Canive 2006	0.7393	0.6931	0.1%	0.74 [−0.62; 2.10]
Chan 2007	0.1495	0.2235	0.7%	0.15 [−0.29; 0.59]
Chouinard 1993	0.4902	0.3096	0.4%	0.49 [−0.12; 1.10]
Claus 1992	−0.3988	0.3117	0.4%	−0.40 [−1.01; 0.21]
Conley 2001	0.0000	0.1060	2.0%	0.00 [−0.21; 0.21]
Cooper 2000a	0.4281	0.1965	0.9%	0.43 [0.04; 0.81]
Copolov 2000	−0.0526	0.0959	2.1%	−0.05 [−0.24; 0.14]
Costa e Silva 1989	0.7242	0.3491	0.3%	0.72 [0.04; 1.41]
Cutler 2008	−0.0220	0.1024	2.0%	−0.02 [−0.22; 0.18]
Davidson 2007	−0.0530	0.1268	1.6%	−0.05 [−0.30; 0.20]
Durgam 2014	−0.1533	0.0981	2.1%	−0.15 [−0.35; 0.04]
Gattaz 2004	0.2413	0.4018	0.3%	0.24 [−0.55; 1.03]
Gelenberg 1979	−0.1047	0.2505	0.6%	−0.10 [−0.60; 0.39]
Grootens 2009	−0.0847	0.2445	0.6%	−0.08 [−0.56; 0.39]
Guirguis 1977	−0.2331	0.3405	0.3%	−0.23 [−0.90; 0.43]
Hale 2000	0.1270	0.1109	1.9%	0.13 [−0.09; 0.34]
Honigfeld 1984a	0.5351	0.4003	0.3%	0.54 [−0.25; 1.32]
Honigfeld 1984c	0.8405	0.2348	0.7%	0.84 [0.38; 1.30]
Hoyberg 1993	0.1435	0.1993	0.9%	0.14 [−0.25; 0.53]
Huttunen 1995	0.1919	0.2025	0.8%	0.19 [−0.21; 0.59]
Hwang 2001	−0.1657	0.2395	0.6%	−0.17 [−0.64; 0.30]
Hwang 2003	−0.1716	0.2929	0.5%	−0.17 [−0.75; 0.40]
Ishigooka 2001	0.1587	0.1594	1.2%	0.16 [−0.15; 0.47]
Janicak 2001	0.2225	0.2549	0.6%	0.22 [−0.28; 0.72]
Janssen CR012625	−0.0822	0.1709	1.1%	−0.08 [−0.42; 0.25]
Kane 2002	0.0080	0.1210	1.7%	0.01 [−0.23; 0.25]
Kane 2007b	−0.1449	0.1025	2.0%	−0.14 [−0.35; 0.06]
Kane 2009 8 weeks	0.2064	0.0843	2.4%	0.21 [0.04; 0.37]
Kane 2010a	0.0764	0.1167	1.8%	0.08 [−0.15; 0.31]
Kluge 2007	−0.2300	0.3664	0.3%	−0.23 [−0.95; 0.49]
Li 2012	0.1299	0.1835	1.0%	0.13 [−0.23; 0.49]
Lundbeck NCT00864045	−0.0324	0.1014	2.0%	−0.03 [−0.23; 0.17]
Marder 1994	0.4935	0.1802	1.0%	0.49 [0.14; 0.85]
Marder 2007c	−0.0343	0.1184	1.7%	−0.03 [−0.27; 0.20]
Martin 2002	−0.0348	0.1037	2.0%	−0.03 [−0.24; 0.17]
Min 1993	−0.4472	0.3435	0.3%	−0.45 [−1.12; 0.23]
Möller 1997	0.2927	0.1466	1.3%	0.29 [0.01; 0.58]
Murasaki 2001	−0.0499	0.1464	1.3%	−0.05 [−0.34; 0.24]
NCT00882518	−0.1177	0.1022	2.0%	−0.12 [−0.32; 0.08]
NCT01104766	0.0770	0.0998	2.1%	0.08 [−0.12; 0.27]
NCT01617187	0.1016	0.1768	1.0%	0.10 [−0.24; 0.45]
NCT01810380	−0.1633	0.1157	1.8%	−0.16 [−0.39; 0.06]
Petit 1996	0.3619	0.1818	1.0%	0.36 [0.01; 0.72]
Peuskens 1995	0.0750	0.0818	2.4%	0.07 [−0.09; 0.24]
Peuskens 1997	0.1314	0.1430	1.4%	0.13 [−0.15; 0.41]
Peuskens 1999	0.2264	0.1329	1.5%	0.23 [−0.03; 0.49]
Potkin 2003	0.0488	0.1252	1.6%	0.05 [−0.20; 0.29]
Potkin 2007c	0.3921	0.1891	0.9%	0.39 [0.02; 0.76]
Puech 1998	0.1946	0.1565	1.2%	0.19 [−0.11; 0.50]
Sacchetti 2008	0.4739	0.2480	0.6%	0.47 [−0.01; 0.96]
Schennach 2018	0.0801	0.1892	0.9%	0.08 [−0.29; 0.45]
Shah 2011	−0.0299	0.1394	1.4%	−0.03 [−0.30; 0.24]
Study 049	−0.1071	0.1460	1.4%	−0.11 [−0.39; 0.18]
Study 231	−0.1028	0.1119	1.8%	−0.10 [−0.32; 0.12]
Study 233	−0.0224	0.1126	1.8%	−0.02 [−0.24; 0.20]
Study 3000	−0.1291	0.1323	1.5%	−0.13 [−0.39; 0.13]
Study 3004	−0.1152	0.1165	1.8%	−0.12 [−0.34; 0.11]
Study 3005	−0.1956	0.0974	2.1%	−0.20 [−0.39; −0.00]
Study 93202 2002	−0.1381	0.2465	0.6%	−0.14 [−0.62; 0.34]
Study 94202 2002	0.2757	0.1934	0.9%	0.28 [−0.10; 0.65]
Tollefson 1997	0.2085	0.0484	3.2%	0.21 [0.11; 0.30]
Wagner 2005	−0.2972	0.3502	0.3%	−0.30 [−0.98; 0.39]
Wetzel 1998	0.4393	0.1797	1.0%	0.44 [0.09; 0.79]
Zborowski 1995	0.0000	0.1336	1.5%	0.00 [−0.26; 0.26]
Zhang 2011	−0.1394	0.1295	1.6%	−0.14 [−0.39; 0.11]
Zhong 2006	−0.0557	0.0787	2.5%	−0.06 [−0.21; 0.10]
Ziegler 1989	0.1397	0.3166	0.4%	0.14 [−0.48; 0.76]
Zimbroff 1997	0.0802	0.1273	1.6%	0.08 [−0.17; 0.33]
**Total (95% CI)**			**95.8%**	**0.05 [0.01; 0.10]**
Heterogeneity: $Tau^2 = 0.0109$; $Chi^2 = 127.44$, df = 75 ($P < 0.01$); $I^2 = 41\%$				
**blinding = 0**				
Avasthi 2001	0.1520	0.4537	0.2%	0.15 [−0.74; 1.04]
de Boer 2011	0.1134	0.3545	0.3%	0.11 [−0.58; 0.81]
de Oliviera 2009	0.0870	0.2178	0.8%	0.09 [−0.34; 0.51]
Dossenbach 2007	0.5316	0.2018	0.8%	0.53 [0.14; 0.93]
Ehrlich 2012	−0.0236	0.4432	0.2%	−0.02 [−0.89; 0.85]
Loza 1999	0.6202	0.3364	0.4%	0.62 [−0.04; 1.28]
Maat 2014	0.1648	0.3101	0.4%	0.16 [−0.44; 0.77]
Taneli 2003	−0.1127	0.2274	0.7%	−0.11 [−0.56; 0.33]
van Bruggen 2003	0.2374	0.3129	0.4%	0.24 [−0.38; 0.85]
**Total (95% CI)**			**4.2%**	**0.21 [0.01; 0.41]**
Heterogeneity: $Tau^2 = 0.0110$; $Chi^2 = 6.76$, df = 8 ($P = 0.56$); $I^2 = 0\%$				
**Total (95% CI)**			**100.0%**	**0.06 [0.02; 0.10]**

Heterogeneity: $Tau^2 = 0.0114$; $Chi^2 = 136.97$, df = 84 ($P < 0.01$); $I^2 = 39\%$
Test for subgroup differences: $Chi^2 = 2.25$, df = 1 ($P = 0.13$)

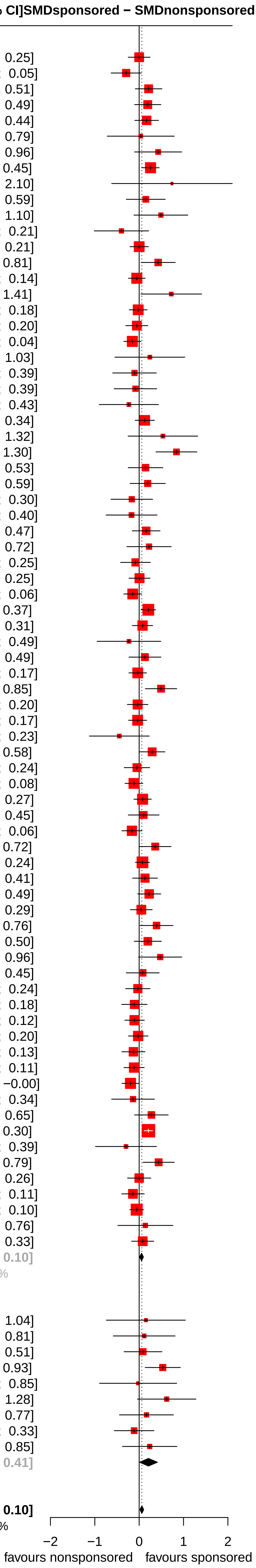

eFigure 7

All-cause discontinuation

1. Results of individual comparisons
2. Difference between blinded and open RCTs by recency
3. Difference between blinded and open RCTs by efficacy
4. Differences between blinded and open RCTs sponsored versus non-sponsored drugs

eFigure 7a all-cause discontinuation individual comparisons

Study or Subgroup	first drug Events	first drug Total	second drug Events	second drug Total	Weight	OR [95% CI]
**blinding = 0**						
de Oliviera 2009	13	66	12	33	12.2%	0.43 [0.17; 1.09]
McCue2006	14	63	9	61	12.4%	1.65 [0.66; 4.16]
**Total (95% CI)**		**129**		**94**	**24.6%**	**0.84 [0.23; 3.16]**
Heterogeneity: $Tau^2 = 0.6827$; $Chi^2 = 4.04$, df = 1 (P = 0.04); $I^2 = 75\%$						
**blinding = 1**						
Kane 2002	76	204	42	104	44.4%	0.88 [0.54; 1.42]
Study 93202 2002	13	34	14	34	11.2%	0.88 [0.33; 2.34]
Study 94202 2002	20	61	29	63	19.8%	0.57 [0.28; 1.19]
**Total (95% CI)**		**299**		**201**	**75.4%**	**0.79 [0.54; 1.14]**
Heterogeneity: $Tau^2 = 0$; $Chi^2 = 0.98$, df = 2 (P = 0.61); $I^2 = 0\%$						
**Total (95% CI)**		**428**		**295**	**100.0%**	**0.80 [0.58; 1.11]**

Heterogeneity: $Tau^2 = 0.0017$; $Chi^2 = 5.06$, df = 4 (P = 0.28); $I^2 = 21\%$

Test for subgroup differences: $Chi^2 = 0.01$, df = 1 (P = 0.92)

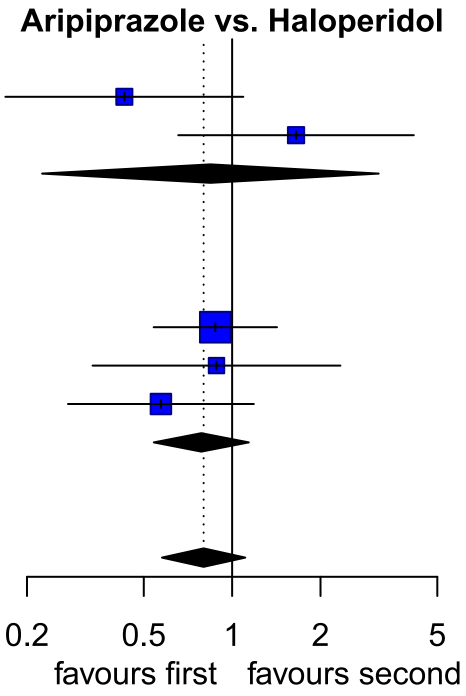

Aripiprazole vs. Olanzapine

Study or Subgroup	first drug Events	first drug Total	second drug Events	second drug Total	Weight	OR [95% CI]
**blinding = 1**						
Fleischhacker 2009_6weeks	104	355	77	348	56.2%	1.46 [1.04; 2.05]
Hatta 2009	12	22	2	17	2.3%	9.00 [1.65; 49.14]
Jindal 2013	4	30	3	30	2.6%	1.38 [0.28; 6.80]
McQuade 2004_6weeks	61	156	60	161	31.8%	1.08 [0.69; 1.70]
**Total (95% CI)**		**563**		**556**	**92.8%**	**1.38 [1.03; 1.84]**
Heterogeneity: $Tau^2 = 0.0069$; $Chi^2 = 5.9$, df = 3 (P = 0.12); $I^2 = 49\%$						
**blinding = 0**						
McCue2006	14	63	8	58	7.2%	1.79 [0.69; 4.64]
**Total (95% CI)**		**63**		**58**	**7.2%**	**1.79 [0.69; 4.64]**
Heterogeneity: not applicable						
**Total (95% CI)**		**626**		**614**	**100.0%**	**1.40 [1.08; 1.81]**

Heterogeneity: $Tau^2 < 0.0001$; $Chi^2 = 6.17$, df = 4 (P = 0.19); $I^2 = 35\%$

Test for subgroup differences: $Chi^2 = 0.26$, df = 1 (P = 0.61)

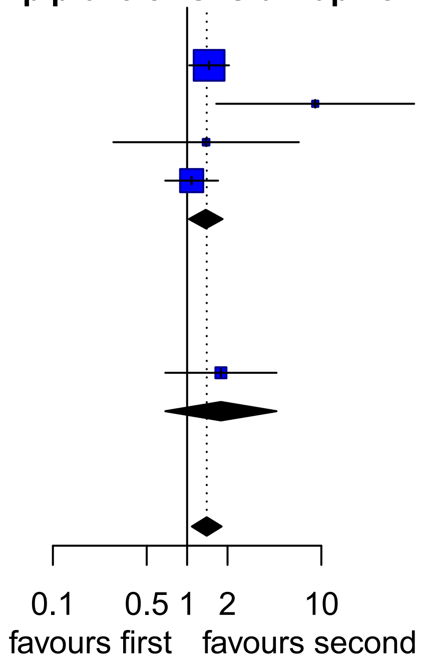

Study or Subgroup	first drug Events	first drug Total	second drug Events	second drug Total	Weight	OR [95% CI]
**blinding = 1**						
Hatta 2009	12	22	11	20	33.6%	0.98 [0.29; 3.31]
**Total (95% CI)**		**22**		**20**	**33.6%**	**0.98 [0.29; 3.31]**
Heterogeneity: not applicable						
**blinding = 0**						
McCue2006	14	63	12	62	66.4%	1.19 [0.50; 2.83]
**Total (95% CI)**		**63**		**62**	**66.4%**	**1.19 [0.50; 2.83]**
Heterogeneity: not applicable						
**Total (95% CI)**		**85**		**82**	**100.0%**	**1.12 [0.55; 2.26]**

Heterogeneity: $Tau^2 = 0$; $Chi^2 = 0.06$, df = 1 (P = 0.80); $I^2 = 0\%$

Test for subgroup differences: $Chi^2 = 0.06$, df = 1 (P = 0.80)

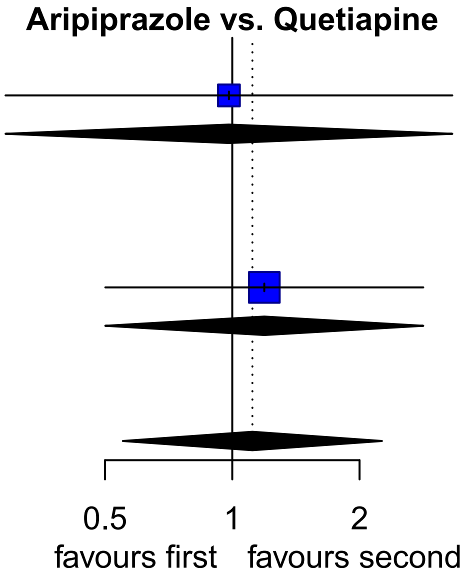

Study or Subgroup	first drug Events	first drug Total	second drug Events	second drug Total	Weight	OR [95% CI]
**blinding = 1**						
Chan 2007	11	49	10	34	12.2%	0.69 [0.26; 1.88]
Hatta 2009	12	22	6	21	7.8%	3.00 [0.85; 10.63]
Potkin 2003	74	202	37	99	40.4%	0.97 [0.59; 1.59]
**Total (95% CI)**		**273**		**154**	**60.4%**	**1.06 [0.65; 1.72]**
Heterogeneity: $Tau^2 = 0.0315$; $Chi^2 = 3.4$, df = 2 (P = 0.18); $I^2 = 41\%$						
**blinding = 0**						
de Boer 2011	5	23	3	21	5.1%	1.67 [0.35; 8.04]
Liemburg 2011	5	20	3	20	5.0%	1.89 [0.38; 9.27]
Maat 2014	18	38	14	42	14.6%	1.80 [0.73; 4.44]
McCue2006	14	63	10	65	14.8%	1.57 [0.64; 3.86]
**Total (95% CI)**		**144**		**148**	**39.6%**	**1.70 [0.98; 2.96]**
Heterogeneity: $Tau^2 = 0$; $Chi^2 = 0.06$, df = 3 (P = 1.00); $I^2 = 0\%$						
**Total (95% CI)**		**417**		**302**	**100.0%**	**1.27 [0.89; 1.82]**
Heterogeneity: $Tau^2 = 0.0197$; $Chi^2 = 5.44$, df = 6 (P = 0.49); $I^2 = 0\%$						
Test for subgroup differences: $Chi^2 = 1.61$, df = 1 (P = 0.20)						

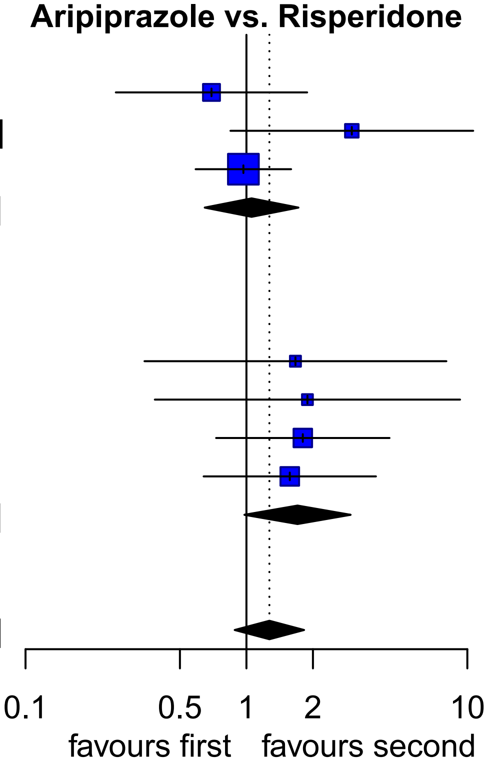

Study or Subgroup	first drug Events	first drug Total	second drug Events	second drug Total	Weight	OR [95% CI]
blinding = 0						
Citrome 2015	24	64	12	33	41.1%	1.05 [0.44; 2.51]
**Total (95% CI)**		**64**		**33**	**41.1%**	**1.05 [0.44; 2.51]**
Heterogeneity: not applicable						
blinding = 1						
NCT00905307	37	90	16	50	58.9%	1.48 [0.72; 3.07]
**Total (95% CI)**		**90**		**50**	**58.9%**	**1.48 [0.72; 3.07]**
Heterogeneity: not applicable						
**Total (95% CI)**		**154**		**83**	**100.0%**	**1.29 [0.74; 2.25]**

Heterogeneity: $Tau^2 = 0$; $Chi^2 = 0.36$, df = 1 (P = 0.55); $I^2 = 0\%$

Test for subgroup differences: $Chi^2 = 0.36$, df = 1 (P = 0.55)

Brexpiprazole vs. Aripiprazole

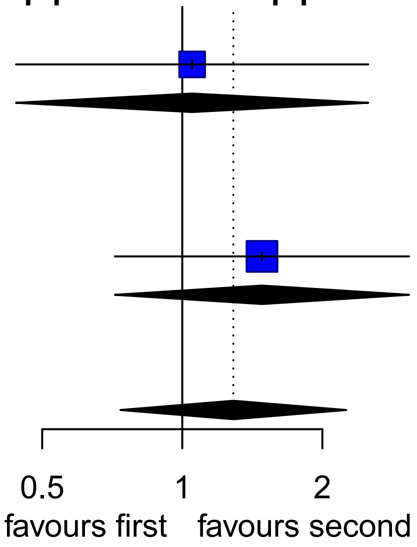

Study or Subgroup	first drug Events	first drug Total	second drug Events	second drug Total	Weight	OR [95% CI]
**blinding = 0**						
Avasthi 2001	3	10	1	17	1.2%	6.86 [0.60; 77.98]
Duggan 2005	16	51	13	53	7.5%	1.41 [0.59; 3.33]
McCue2006	9	61	8	58	5.6%	1.08 [0.39; 3.03]
**Total (95% CI)**		**122**		**128**	**14.3%**	**1.42 [0.75; 2.68]**
Heterogeneity: Tau^2 = < 0.0001; Chi^2 = 1.88, df = 2 (P = 0.39); I^2 = 0%						
**blinding = 1**						
Beasley 1996b	39	69	73	133	12.8%	1.07 [0.59; 1.92]
Beasley 1997	38	81	67	175	14.3%	1.42 [0.84; 2.43]
Boulay 2007	5	13	0	14	0.8%	18.76 [0.92; 383.10]
Ishigooka 2001	30	89	18	93	10.6%	2.12 [1.08; 4.17]
Lahti 2009	2	14	1	18	1.1%	2.83 [0.23; 34.92]
Rosenheck 2003w6	35	150	29	159	13.7%	1.36 [0.79; 2.37]
Sergi 2007	5	20	13	40	4.3%	0.69 [0.21; 2.32]
Tollefson 1997	351	660	448	1336	28.1%	2.25 [1.86; 2.72]
**Total (95% CI)**		**1096**		**1968**	**85.7%**	**1.64 [1.21; 2.22]**
Heterogeneity: Tau^2 = 0.0677; Chi^2 = 14.3, df = 7 (P = 0.05); I^2 = 51%						
**Total (95% CI)**		**1218**		**2096**	**100.0%**	**1.62 [1.24; 2.11]**

Heterogeneity: Tau^2 = 0.0572; Chi^2 = 17.05, df = 10 (P = 0.07); I^2 = 41%

Test for subgroup differences: Chi^2 = 0.16, df = 1 (P = 0.69)

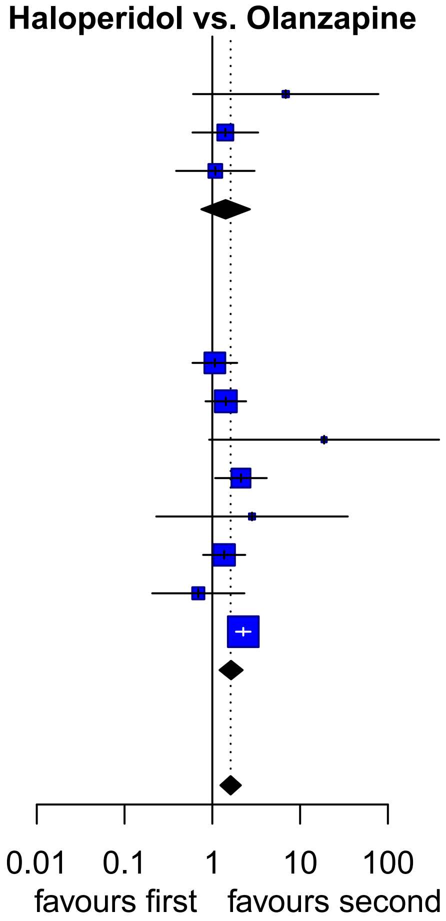

Study or Subgroup	first drug Events	first drug Total	second drug Events	second drug Total	Weight	OR [95% CI]
blinding = 1						
Blin 1996	6	20	4	21	2.3%	1.82 [0.43; 7.76]
Borison 1992	31	53	26	53	8.3%	1.46 [0.68; 3.15]
Cavallaro 2001	7	16	6	17	2.5%	1.43 [0.35; 5.79]
Ceskova 1993	3	31	0	31	0.5%	7.74 [0.38; 156.36]
Chouinard 1993	13	21	5	22	2.8%	5.53 [1.46; 20.90]
Claus 1992	6	22	2	22	1.6%	3.75 [0.66; 21.15]
Ghaleiha 2011	1	17	1	17	0.6%	1.00 [0.06; 17.41]
Janicak 2001	20	32	17	30	4.7%	1.27 [0.46; 3.52]
Liu 2000	9	28	7	28	3.6%	1.42 [0.44; 4.56]
Marder 1994	38	66	29	64	10.2%	1.64 [0.82; 3.28]
Mesotten 1991	3	32	6	28	2.2%	0.38 [0.09; 1.69]
Min 1993	0	19	3	16	0.5%	0.10 [0.00; 2.07]
Murasaki 1993	14	95	14	97	7.6%	1.02 [0.46; 2.28]
Peuskens 1995	63	226	101	457	36.6%	1.36 [0.95; 1.96]
Sergi 2007	5	20	12	40	3.3%	0.78 [0.23; 2.63]
Svestka 1990	1	18	2	18	0.8%	0.47 [0.04; 5.71]
Yen 2004	7	20	7	21	2.9%	1.08 [0.30; 3.92]
**Total (95% CI)**		**736**		**982**	**91.1%**	**1.36 [1.08; 1.71]**
Heterogeneity: Tau^2 = < 0.0001; Chi^2 = 15.18, df = 16 (P = 0.51); I^2 = 0%						
blinding = 0						
Fakra 2008	1	15	4	15	0.9%	0.20 [0.02; 2.02]
McCue2006	9	61	10	65	5.1%	0.95 [0.36; 2.53]
Mirabzadeh 2014	0	29	0	37	0.0%	
Nam 2004	6	20	10	20	2.9%	0.43 [0.12; 1.57]
**Total (95% CI)**		**125**		**137**	**8.9%**	**0.61 [0.28; 1.34]**
Heterogeneity: Tau^2 = 0.0355; Chi^2 = 1.99, df = 2 (P = 0.37); I^2 = 0%						
**Total (95% CI)**		**861**		**1119**	**100.0%**	**1.27 [1.02; 1.58]**

Heterogeneity: Tau^2 < 0.0001; Chi^2 = 20.99, df = 19 (P = 0.34); I^2 = 9%

Test for subgroup differences: Chi^2 = 3.66, df = 1 (P = 0.06)

Haloperidol vs. Risperidone

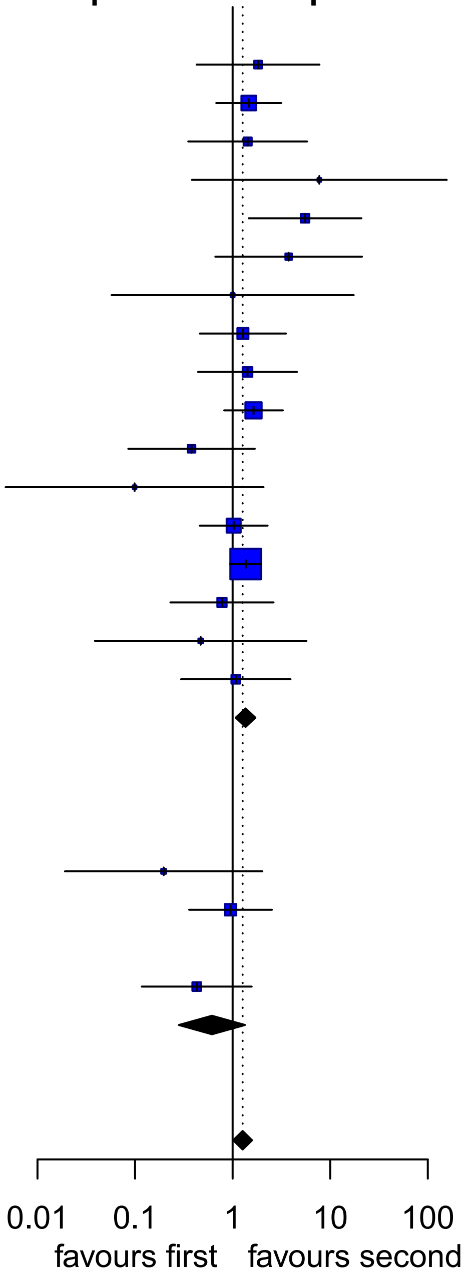

Study or Subgroup	first drug Events	first drug Total	second drug Events	second drug Total	Weight	OR [95% CI]
**blinding = 1**						
Arvanitis 1997	52	105	34	52	15.7%	0.52 [0.26; 1.03]
Copolov 2000	69	221	80	227	47.8%	0.83 [0.56; 1.24]
Murasaki 2001	34	100	43	97	22.3%	0.65 [0.36; 1.15]
**Total (95% CI)**		**426**		**376**	**85.8%**	**0.72 [0.53; 0.96]**
Heterogeneity: Tau^2 = 0; Chi^2 = 1.53, df = 2 (P = 0.46); I^2 = 0%						
**blinding = 0**						
Atmaca 2002	0	18	0	17	0.0%	
McCue2006	12	62	9	61	8.3%	1.39 [0.54; 3.58]
Taneli 2003	11	45	6	34	6.0%	1.51 [0.50; 4.60]
**Total (95% CI)**		**125**		**112**	**14.2%**	**1.44 [0.70; 2.96]**
Heterogeneity: Tau^2 = 0; Chi^2 = 0.01, df = 1 (P = 0.91); I^2 = 0%						
**Total (95% CI)**		**551**		**488**	**100.0%**	**0.79 [0.60; 1.04]**

Heterogeneity: Tau^2 < 0.0001; Chi^2 = 4.62, df = 4 (P = 0.33); I^2 = 13%

Test for subgroup differences: Chi^2 = 3.07, df = 1 (P = 0.08)

Quetiapine vs. Haloperidol

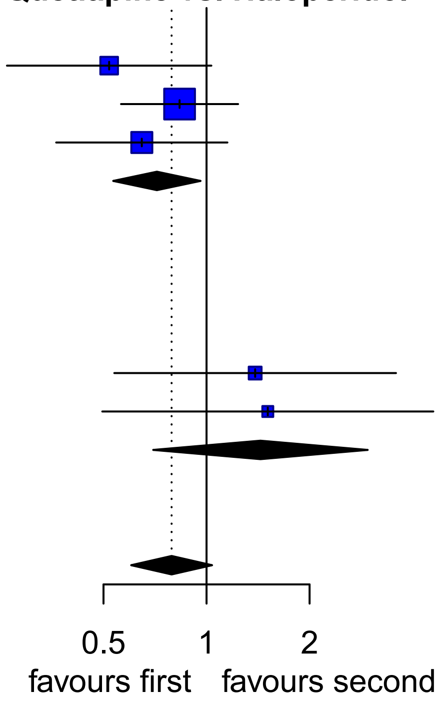

Study or Subgroup	first drug Events	first drug Total	second drug Events	second drug Total	Weight	OR [95% CI]
**blinding = 1**						
Hatta 2009	11	20	2	17	2.7%	9.17 [1.64; 51.11]
Lieberman 2005 12weeks	134	337	102	336	77.5%	1.51 [1.10; 2.08]
Ozguven 2004	4	19	0	15	0.9%	9.00 [0.45; 181.74]
Riedel 2007	17	26	15	26	6.3%	1.39 [0.45; 4.25]
Sacchetti 2008	4	25	5	25	3.7%	0.76 [0.18; 3.25]
**Total (95% CI)**		**427**		**419**	**91.0%**	**1.57 [1.17; 2.11]**
Heterogeneity: Tau^2 = < 0.0001; Chi^2 = 6.4, df = 4 (P = 0.17); I^2 = 37%						
**blinding = 0**						
McCue2006	12	62	8	58	8.3%	1.50 [0.56; 3.98]
Yamashita 2004	1	28	0	20	0.7%	2.24 [0.09; 57.75]
**Total (95% CI)**		**90**		**78**	**9.0%**	**1.55 [0.61; 3.95]**
Heterogeneity: Tau^2 = 0; Chi^2 = 0.05, df = 1 (P = 0.82); I^2 = 0%						
**Total (95% CI)**		**517**		**497**	**100.0%**	**1.57 [1.18; 2.08]**
Heterogeneity: Tau^2 = 0; Chi^2 = 6.45, df = 6 (P = 0.37); I^2 = 7%						
Test for subgroup differences: Chi^2 = 0.00, df = 1 (P = 0.98)						

Quetiapine vs. Olanzapine

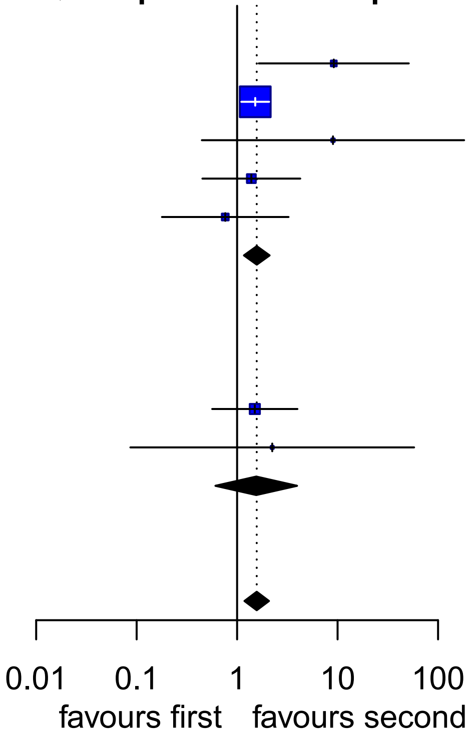

Study or Subgroup	first drug Events	first drug Total	second drug Events	second drug Total	Weight	OR [95% CI]
**blinding = 1**						
Hatta 2009	11	20	6	21	2.4%	3.06 [0.84; 11.14]
Li 2012	19	60	11	59	5.5%	2.02 [0.86; 4.74]
Lieberman 2005 12weeks	134	337	129	341	41.7%	1.08 [0.80; 1.48]
Sacchetti 2008	4	25	5	25	1.9%	0.76 [0.18; 3.25]
Zhong 2006	184	338	167	335	43.4%	1.20 [0.89; 1.63]
**Total (95% CI)**		**780**		**781**	**94.9%**	**1.20 [0.98; 1.47]**
Heterogeneity: Tau^2 = < 0.0001; Chi^2 = 4.24, df = 4 (P = 0.38); I^2 = 6%						
**blinding = 0**						
Knegtering 2004	0	25	0	26	0.0%	
McCue2006	12	62	10	65	4.7%	1.32 [0.52; 3.32]
Yamashita 2004	1	28	0	20	0.4%	2.24 [0.09; 57.75]
**Total (95% CI)**		**115**		**111**	**5.1%**	**1.37 [0.57; 3.33]**
Heterogeneity: Tau^2 = 0; Chi^2 = 0.09, df = 1 (P = 0.76); I^2 = 0%						
**Total (95% CI)**		**895**		**892**	**100.0%**	**1.21 [0.99; 1.48]**
Heterogeneity: Tau^2 = 0; Chi^2 = 4.41, df = 6 (P = 0.62); I^2 = 0%						
Test for subgroup differences: Chi^2 = 0.08, df = 1 (P = 0.77)						

Quetiapine vs. Risperidone

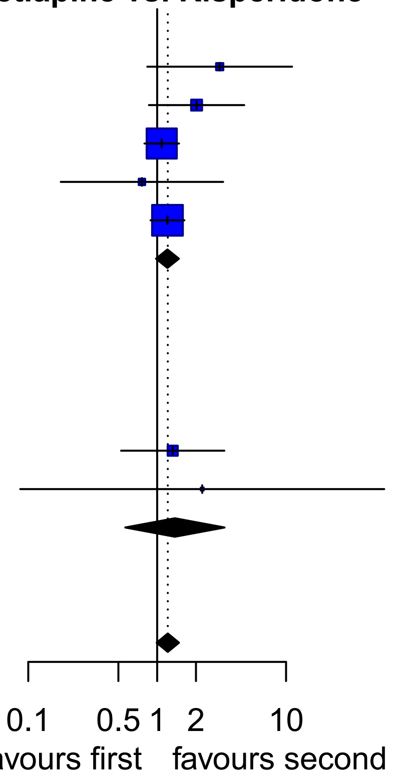

Study or Subgroup	first drug Events	first drug Total	second drug Events	second drug Total	Weight	OR [95% CI]
**blinding = 0**						
Chen 2018	3	28	25	53	5.7%	0.13 [0.04; 0.50]
Chung 2012	7	30	16	60	8.7%	0.84 [0.30; 2.32]
Ingole 2009	0	30	0	30	0.0%	
Knegtering 2006	0	21	0	25	0.0%	
McCue2006	10	65	8	58	8.9%	1.14 [0.42; 3.11]
Yamashita 2004	0	20	0	20	0.0%	
**Total (95% CI)**		**194**		**246**	**23.3%**	**0.54 [0.15; 1.86]**
Heterogeneity: $Tau^2 = 0.8840$; $Chi^2 = 6.93$, df = 2 (P = 0.03); $I^2 = 71\%$						
**blinding = 1**						
Conley 2001	53	188	43	189	23.2%	1.33 [0.84; 2.12]
Hatta 2009	6	21	2	17	3.4%	3.00 [0.52; 17.32]
Hatta 2013	5	20	5	22	5.0%	1.13 [0.27; 4.69]
Lieberman 2005 12weeks	129	341	102	336	30.1%	1.40 [1.01; 1.92]
Sacchetti 2008	5	25	5	25	5.2%	1.00 [0.25; 4.00]
Sergi 2007	12	40	13	40	9.8%	0.89 [0.35; 2.29]
**Total (95% CI)**		**635**		**629**	**76.7%**	**1.34 [1.05; 1.70]**
Heterogeneity: $Tau^2 = 0$; $Chi^2 = 1.82$, df = 5 (P = 0.87); $I^2 = 0\%$						
**Total (95% CI)**		**829**		**875**	**100.0%**	**1.08 [0.77; 1.52]**
Heterogeneity: $Tau^2 = 0.0727$; $Chi^2 = 13.72$, df = 8 (P = 0.09); $I^2 = 42\%$						
Test for subgroup differences: $Chi^2 = 1.99$, df = 1 (P = 0.16)						

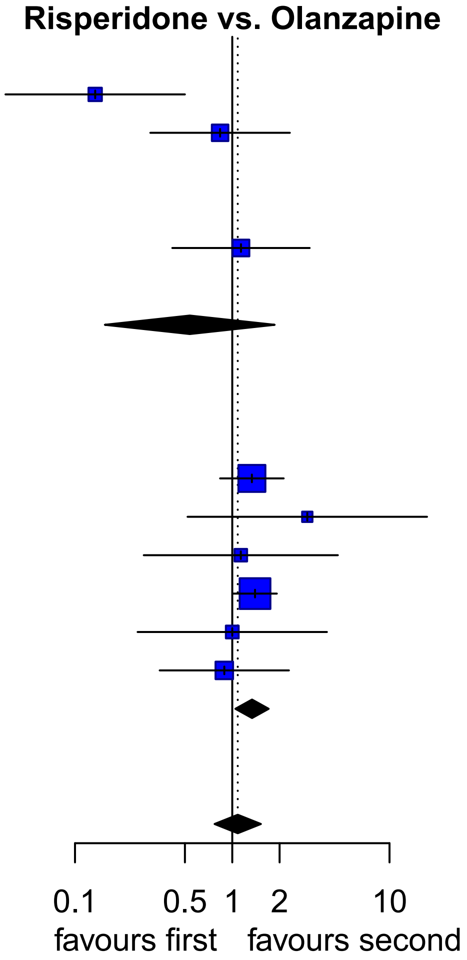

Study or Subgroup	first drug Events	first drug Total	second drug Events	second drug Total	Weight	OR [95% CI]
**blinding = 0**						
McCue2006	14	59	14	63	27.9%	1.09 [0.47; 2.53]
**Total (95% CI)**		**59**		**63**	**27.9%**	**1.09 [0.47; 2.53]**
Heterogeneity: not applicable						
**blinding = 1**						
Zimbroff 2007	42	127	40	129	72.1%	1.10 [0.65; 1.86]
**Total (95% CI)**		**127**		**129**	**72.1%**	**1.10 [0.65; 1.86]**
Heterogeneity: not applicable						
**Total (95% CI)**		**186**		**192**	**100.0%**	**1.10 [0.70; 1.71]**

Heterogeneity: $Tau^2 = 0$; $Chi^2 = 0.00$, df = 1 (P = 0.98); $I^2 = 0\%$

Test for subgroup differences: $Chi^2 = 0.00$, df = 1 (P = 0.98)

Ziprasidone vs. Aripiprazole

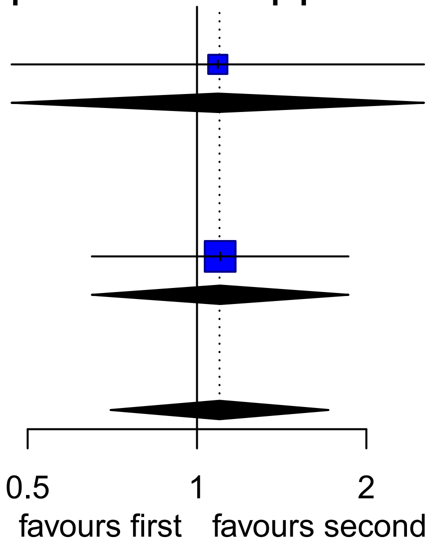

Study or Subgroup	first drug Events	first drug Total	second drug Events	second drug Total	Weight	OR [95% CI]
**blinding = 1**						
Brook 2005	137	429	47	138	46.5%	0.91 [0.61; 1.36]
Corripio 2005	0	10	0	10	0.0%	
Goff 1998	8	20	8	17	4.5%	0.75 [0.20; 2.77]
Schennach 2018	17	54	21	58	12.5%	0.81 [0.37; 1.78]
Study 115 2000	77	164	37	85	27.6%	1.15 [0.68; 1.95]
**Total (95% CI)**		**677**		**308**	**91.1%**	**0.95 [0.71; 1.27]**
Heterogeneity: $Tau^2 = 0$; $Chi^2 = 0.83$, df = 3 (P = 0.84); $I^2 = 0\%$						
**blinding = 0**						
McCue2006	14	59	9	61	8.9%	1.80 [0.71; 4.54]
**Total (95% CI)**		**59**		**61**	**8.9%**	**1.80 [0.71; 4.54]**
Heterogeneity: not applicable						
**Total (95% CI)**		**736**		**369**	**100.0%**	**1.01 [0.76; 1.33]**

Heterogeneity: $Tau^2 = 0$; $Chi^2 = 2.48$, df = 4 (P = 0.65); $I^2 = 0\%$

Test for subgroup differences: $Chi^2 = 1.65$, df = 1 (P = 0.20)

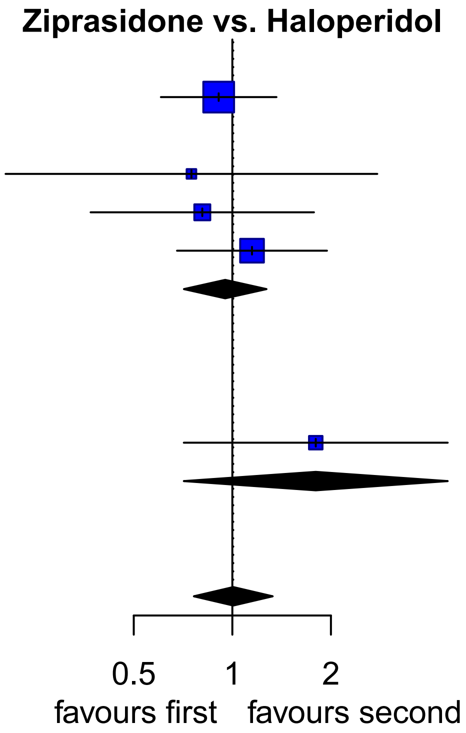

Study or Subgroup	first drug Events	first drug Total	second drug Events	second drug Total	Weight	OR [95% CI]
**blinding = 0**						
Ehrlich 2012	8	16	7	21	4.0%	2.00 [0.53; 7.60]
McCue2006	14	59	8	58	7.8%	1.94 [0.75; 5.07]
**Total (95% CI)**		**75**		**79**	**11.8%**	**1.96 [0.90; 4.27]**
Heterogeneity: Tau^2 = 0; Chi^2 = 0, df = 1 (P = 0.97); I^2 = 0%						
**blinding = 1**						
Grootens 2009	11	39	6	35	5.7%	1.90 [0.62; 5.83]
Lieberman 2005 12weeks	91	185	102	336	52.3%	2.22 [1.53; 3.22]
Simpson 2004	66	136	49	133	30.2%	1.62 [0.99; 2.63]
**Total (95% CI)**		**360**		**504**	**88.2%**	**1.97 [1.48; 2.62]**
Heterogeneity: Tau^2 = 0; Chi^2 = 1.04, df = 2 (P = 0.59); I^2 = 0%						
**Total (95% CI)**		**435**		**583**	**100.0%**	**1.97 [1.51; 2.58]**

Heterogeneity: Tau^2 = 0; Chi^2 = 1.04, df = 4 (P = 0.90); I^2 = 0%

Test for subgroup differences: Chi^2 = 0.00, df = 1 (P = 0.99)

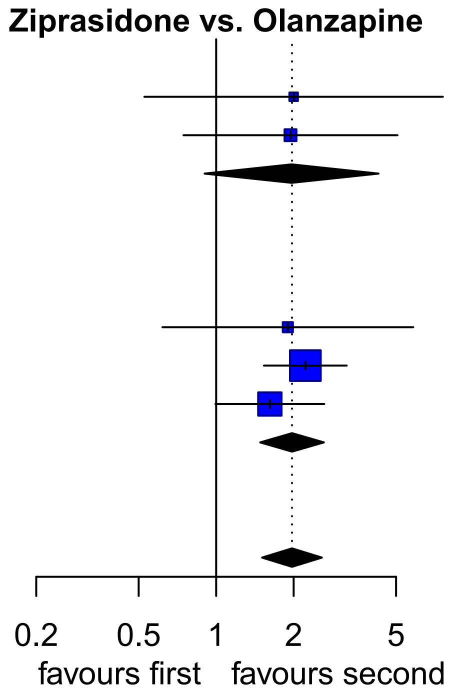

Study or Subgroup	first drug Events	first drug Total	second drug Events	second drug Total	Weight	OR [95% CI]
**blinding = 1**						
Lieberman 2005 12weeks	91	185	134	337	85.3%	1.47 [1.02; 2.11]
**Total (95% CI)**		**185**		**337**	**85.3%**	**1.47 [1.02; 2.11]**
Heterogeneity: not applicable						
**blinding = 0**						
McCue2006	14	59	12	62	14.7%	1.30 [0.54; 3.09]
**Total (95% CI)**		**59**		**62**	**14.7%**	**1.30 [0.54; 3.09]**
Heterogeneity: not applicable						
**Total (95% CI)**		**244**		**399**	**100.0%**	**1.44 [1.03; 2.01]**

Heterogeneity: Tau^2 = 0; Chi^2 = 0.07, df = 1 (P = 0.80); I^2 = 0%

Test for subgroup differences: Chi^2 = 0.07, df = 1 (P = 0.80)

Ziprasidone vs. Quetiapine

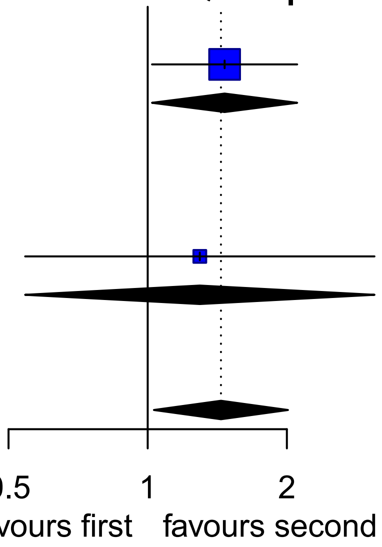

Study or Subgroup	first drug Events	first drug Total	second drug Events	second drug Total	Weight	OR [95% CI]
**blinding = 1**						
Addington 2004	55	149	43	147	28.8%	1.42 [0.87; 2.30]
Lieberman 2005 12weeks	91	185	129	341	52.1%	1.59 [1.11; 2.28]
Zhang 2011	21	118	10	121	10.6%	2.40 [1.08; 5.35]
**Total (95% CI)**		**452**		**609**	**91.6%**	**1.61 [1.22; 2.11]**
Heterogeneity: $Tau^2 = 0$; $Chi^2 = 1.23$, df = 2 (P = 0.54); $I^2 = 0\%$						
**blinding = 0**						
McCue2006	14	59	10	65	8.4%	1.71 [0.69; 4.22]
**Total (95% CI)**		**59**		**65**	**8.4%**	**1.71 [0.69; 4.22]**
Heterogeneity: not applicable						
**Total (95% CI)**		**511**		**674**	**100.0%**	**1.62 [1.25; 2.10]**

Heterogeneity: $Tau^2 = 0$; $Chi^2 = 1.25$, df = 3 (P = 0.74); $I^2 = 0\%$

Test for subgroup differences: $Chi^2 = 0.02$, df = 1 (P = 0.90)

Ziprasidone vs. Risperidone

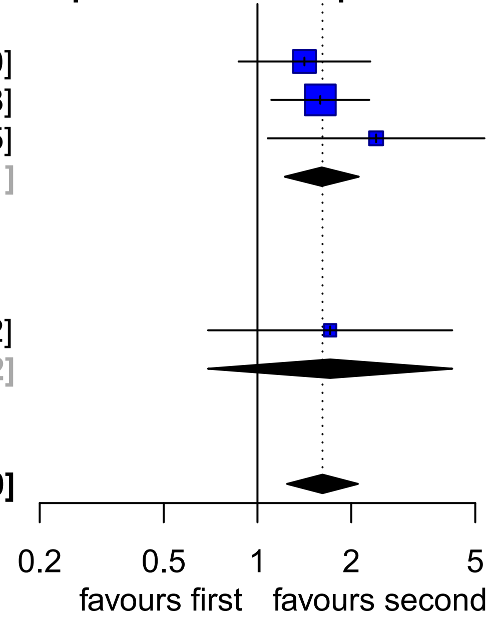

eFigure 7b all cause discontinuation ratio of blinded and open trials, more recent drug listed first

Study	TE	SE	Weight	OR [95% CI]
Aripiprazole vs. Ziprasidone, (nbl=256, nop=122)	−0.01	0.5072	5.5%	0.99 [0.37; 2.68]
Ziprasidone vs. Quetiapine, (nbl=522, nop=121)	0.12	0.4806	6.1%	1.13 [0.44; 2.90]
Quetiapine vs. Risperidone, (nbl=1561, nop=226)	−0.13	0.4647	6.5%	0.87 [0.35; 2.18]
Quetiapine vs. Olanzapine, (nbl=846, nop=168)	0.01	0.5003	5.6%	1.01 [0.38; 2.70]
Aripiprazole vs. Quetiapine, (nbl=42, nop=125)	−0.19	0.7619	2.4%	0.82 [0.19; 3.67]
Aripiprazole vs. Olanzapine, (nbl=1119, nop=121)	−0.26	0.5084	5.5%	0.77 [0.28; 2.09]
Ziprasidone vs. Olanzapine, (nbl=864, nop=154)	0.00	0.4228	7.9%	1.00 [0.44; 2.30]
Aripiprazole vs. Haloperidol, (nbl=500, nop=223)	−0.07	0.6998	2.9%	0.93 [0.24; 3.67]
Brexpiprazole vs. Aripiprazole, (nbl=140, nop=97)	0.35	0.5792	4.2%	1.41 [0.45; 4.40]
Olanzapine vs. Risperidone, (nbl=1264, nop=440)	−0.91	0.6461	3.4%	0.40 [0.11; 1.43]
Aripiprazole vs. Risperidone, (nbl=427, nop=292)	−0.48	0.3767	10.0%	0.62 [0.30; 1.30]
Ziprasidone vs. Haloperidol, (nbl=985, nop=120)	−0.64	0.4959	5.7%	0.53 [0.20; 1.40]
Risperidone vs. Haloperidol, (nbl=1718, nop=262)	−0.79	0.4149	8.2%	0.45 [0.20; 1.02]
Olanzapine vs. Haloperidol, (nbl=3064, nop=250)	−0.14	0.3600	10.9%	0.87 [0.43; 1.76]
Quetiapine vs. Haloperidol, (nbl=802, nop=237)	−0.70	0.3975	8.9%	0.50 [0.23; 1.09]
Ziprasidone vs. Risperidone, (nbl=1061, nop=124)	−0.06	0.4809	6.1%	0.94 [0.37; 2.41]
**Total (95% CI)**			**100.0%**	**0.76 [0.61; 0.96]**

Heterogeneity: $Tau^2 = 0$; $Chi^2 = 7.88$, df = 15 (P = 0.93); $I^2 = 0\%$

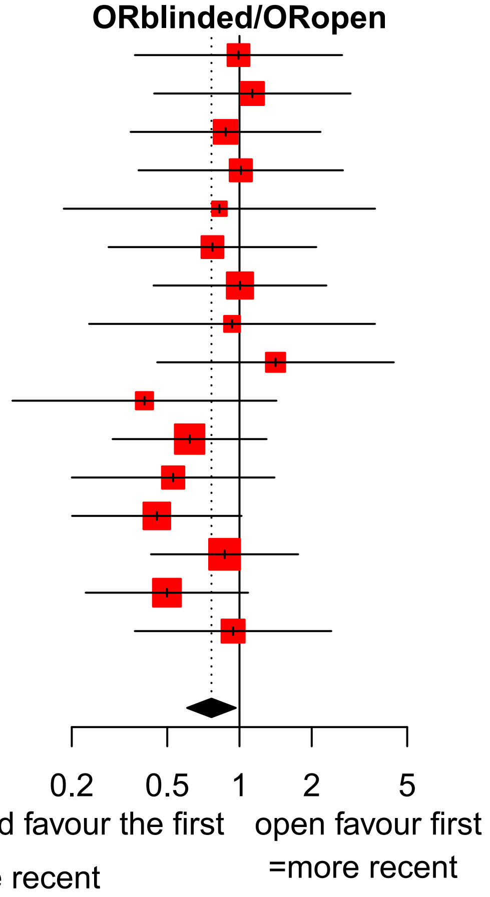

eFigure 7c all cause discontinuation ratio of blinded and open drug with more drop-out according to Huhn et al. 2019 listed first

Study	logOR	SE	Weight	OR [95% CI]
Ziprasidone vs. Aripiprazole, (nbl=256, nop=122)	0.0096	0.5072	5.5%	1.01 [0.37; 2.73]
Ziprasidone vs. Quetiapine, (nbl=522, nop=121)	0.1234	0.4806	6.1%	1.13 [0.44; 2.90]
Quetiapine vs. Risperidone, (nbl=1561, nop=226)	−0.1336	0.4647	6.5%	0.87 [0.35; 2.18]
Quetiapine vs. Olanzapine, (nbl=846, nop=168)	0.0119	0.5003	5.6%	1.01 [0.38; 2.70]
Quetiapine vs. Aripiprazole, (nbl=42, nop=125)	0.1927	0.7619	2.4%	1.21 [0.27; 5.40]
Aripiprazole vs. Olanzapine, (nbl=1119, nop=121)	−0.2595	0.5084	5.5%	0.77 [0.28; 2.09]
Ziprasidone vs. Olanzapine, (nbl=864, nop=154)	0.0044	0.4228	7.9%	1.00 [0.44; 2.30]
Haloperidol vs. Aripiprazole, (nbl=500, nop=223)	0.0714	0.6998	2.9%	1.07 [0.27; 4.23]
Brexpiprazole vs. Aripiprazole, (nbl=140, nop=97)	0.3456	0.5792	4.2%	1.41 [0.45; 4.40]
Risperidone vs. Olanzapine, (nbl=1264, nop=440)	0.9119	0.6461	3.4%	2.49 [0.70; 8.83]
Aripiprazole vs. Risperidone, (nbl=427, nop=292)	−0.4787	0.3767	10.0%	0.62 [0.30; 1.30]
Haloperidol vs. Ziprasidone, (nbl=985, nop=120)	0.6366	0.4959	5.7%	1.89 [0.72; 5.00]
Haloperidol vs. Risperidone, (nbl=1718, nop=262)	0.7934	0.4149	8.2%	2.21 [0.98; 4.99]
Haloperidol vs. Olanzapine, (nbl=3064, nop=250)	0.1431	0.3600	10.9%	1.15 [0.57; 2.34]
Haloperidol vs. Quetiapine, (nbl=802, nop=237)	0.6967	0.3975	8.9%	2.01 [0.92; 4.37]
Ziprasidone vs. Risperidone, (nbl=1061, nop=124)	−0.0617	0.4809	6.1%	0.94 [0.37; 2.41]
**Total (95% CI)**			**100.0%**	**1.18 [0.94; 1.49]**

Heterogeneity: $Tau^2 = 0$; $Chi^2 = 11.04$, df = 15 (P = 0.75); $I^2 = 0\%$

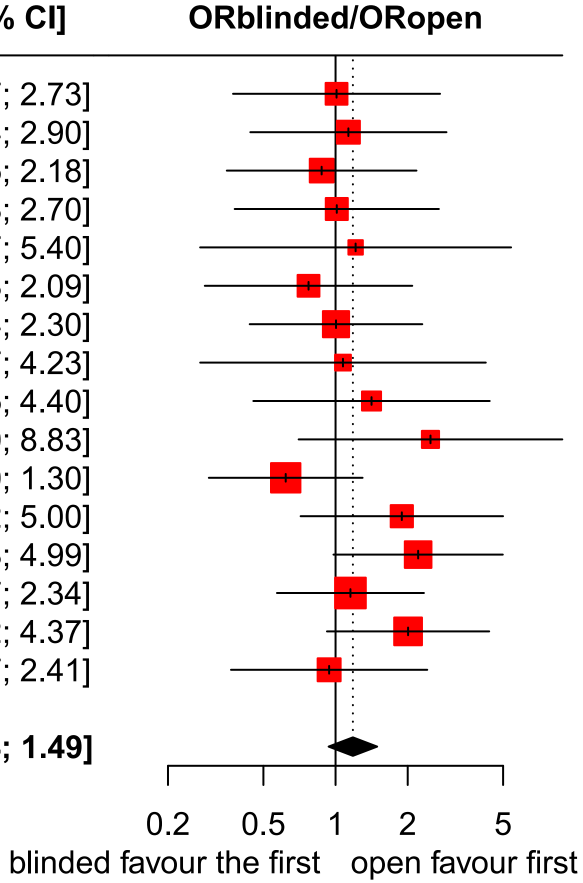

eFigure 7d all cause discontinuation sponsored vs non sponsored drugs and blinded versus open trials

Study or Subgroup	TE	SE	Weight	OR [95% CI]
blinding = 1				
Addington 2004	−0.35	0.2484	1.4%	0.71 [0.43; 1.15]
Arvanitis 1997	0.66	0.3508	1.0%	1.93 [0.97; 3.83]
Azorin 2006	−0.32	0.3084	1.1%	0.72 [0.40; 1.33]
Beasley 1996b	0.07	0.2989	1.2%	1.07 [0.59; 1.92]
Beasley 1997	0.35	0.2716	1.3%	1.42 [0.84; 2.43]
Blin 1996	−0.22	0.7010	0.3%	0.80 [0.20; 3.17]
Boulay 2007	2.93	1.5390	0.1%	18.76 [0.92; 383.10]
Brook 2005	0.10	0.2073	1.6%	1.10 [0.73; 1.65]
Bueno 1979	0.00	1.4510	0.1%	1.00 [0.06; 17.18]
Cavallaro 2001	0.35	0.7152	0.3%	1.43 [0.35; 5.79]
Chan 2007	0.36	0.5088	0.6%	1.44 [0.53; 3.90]
Chiu 1976	0.89	0.5163	0.6%	2.43 [0.88; 6.68]
Chouinard 1993	1.71	0.6788	0.4%	5.53 [1.46; 20.90]
Clark 1970a	1.75	1.5933	0.1%	5.74 [0.25; 130.37]
Clark 1972	0.69	1.2719	0.1%	2.00 [0.17; 24.19]
Clark 1975	0.49	0.9968	0.2%	1.62 [0.23; 11.46]
Claus 1992	1.32	0.8827	0.2%	3.75 [0.66; 21.15]
Conley 2001	−0.29	0.2374	1.5%	0.75 [0.47; 1.19]
Cooper 2000a	0.47	0.3972	0.8%	1.60 [0.73; 3.48]
Copolov 2000	0.18	0.2009	1.7%	1.20 [0.81; 1.78]
Corripio 2005	.	.	0.0%	
Costa e Silva 1989	0.00	1.0541	0.2%	1.00 [0.13; 7.89]
Cutler 2008	−0.05	0.2082	1.6%	0.95 [0.63; 1.43]
Davidson 2007	−0.28	0.2654	1.3%	0.75 [0.45; 1.27]
Dieterle 1991	0.81	0.6455	0.4%	2.25 [0.63; 7.97]
Durgam 2014	−0.34	0.2134	1.6%	0.71 [0.47; 1.08]
Fleischhacker 2009_6weeks	−0.38	0.1740	1.8%	0.69 [0.49; 0.96]
Freeman 1969	1.71	1.5820	0.1%	5.54 [0.25; 123.08]
Gattaz 2004	−0.80	0.8433	0.2%	0.45 [0.09; 2.35]
Gelenberg 1979	0.22	1.0567	0.2%	1.25 [0.16; 9.92]
Goff 1998	0.29	0.6667	0.4%	1.33 [0.36; 4.93]
Gowardman 1973	.	.	0.0%	
Grootens 2009	−0.64	0.5725	0.5%	0.53 [0.17; 1.62]
Guirguis 1977	0.23	0.6268	0.4%	1.26 [0.37; 4.32]
Hale 2000	0.08	0.2244	1.5%	1.09 [0.70; 1.69]
Harnryd 1984	−0.21	0.6463	0.4%	0.81 [0.23; 2.88]
Hera 041−021	−0.12	0.2424	1.4%	0.89 [0.55; 1.43]
Hera 041−022	−0.00	0.2958	1.2%	1.00 [0.56; 1.78]
Honigfeld 1984a	1.42	0.7472	0.3%	4.13 [0.95; 17.85]
Honigfeld 1984c	0.84	0.5137	0.6%	2.33 [0.85; 6.36]
Honigfeld 1984d	−0.03	0.4491	0.7%	0.97 [0.40; 2.34]
Hoyberg 1993	0.17	0.4353	0.7%	1.19 [0.51; 2.79]
Huttunen 1995	0.44	0.4142	0.8%	1.55 [0.69; 3.50]
Hwang 2001	−0.47	0.5644	0.5%	0.62 [0.21; 1.89]
Hwang 2003	−2.16	1.5407	0.1%	0.11 [0.01; 2.35]
Ishigooka 2001	0.75	0.3452	1.0%	2.12 [1.08; 4.17]
Itil 1971	0.50	0.5799	0.5%	1.64 [0.53; 5.12]
Itoh 1977	−2.15	1.5064	0.1%	0.12 [0.01; 2.23]
Janicak 2001	0.24	0.5187	0.6%	1.27 [0.46; 3.52]
Janssen CR012625	0.04	0.3526	1.0%	1.04 [0.52; 2.07]
Kane 2002	0.13	0.2468	1.4%	1.14 [0.70; 1.85]
Kane 2007b	0.04	0.2246	1.5%	1.04 [0.67; 1.62]
Kane 2010a	0.26	0.2341	1.5%	1.29 [0.82; 2.05]
Kingstone 1970	1.50	1.1668	0.1%	4.47 [0.45; 44.01]
Kluge 2007	1.25	1.2199	0.1%	3.50 [0.32; 38.23]
Kramer 1978	−0.72	0.5437	0.5%	0.49 [0.17; 1.41]
Li 2012	−0.70	0.4345	0.7%	0.49 [0.21; 1.16]
Lin 2003	−2.56	1.4864	0.1%	0.08 [0.00; 1.43]
Lundbeck NCT00864045	−0.84	0.2444	1.4%	0.43 [0.27; 0.70]
Marder 1994	0.49	0.3537	1.0%	1.64 [0.82; 3.28]
Marder 2007c	0.06	0.2337	1.5%	1.06 [0.67; 1.67]
Martin 2002	0.10	0.2509	1.4%	1.11 [0.68; 1.81]
McQuade 2004_6weeks	−0.08	0.2313	1.5%	0.93 [0.59; 1.46]
Mesotten 1991	−0.97	0.7615	0.3%	0.38 [0.09; 1.69]
Meyer−Lindenberg 1997	−0.54	0.6042	0.4%	0.58 [0.18; 1.91]
Min 1993	−2.31	1.5528	0.1%	0.10 [0.00; 2.07]
Möller 1997	0.65	0.3122	1.1%	1.92 [1.04; 3.53]
Murasaki 2001	0.44	0.2938	1.2%	1.55 [0.87; 2.75]
NCT00882518	0.21	0.2493	1.4%	1.24 [0.76; 2.01]
NCT01104766	−0.50	0.2215	1.6%	0.60 [0.39; 0.93]
NCT01617187	−0.78	0.3952	0.8%	0.46 [0.21; 0.99]
NCT01810380	−0.26	0.2733	1.3%	0.77 [0.45; 1.32]
Petit 1996	0.32	0.3605	0.9%	1.38 [0.68; 2.80]
Peuskens 1995	0.31	0.1863	1.8%	1.36 [0.95; 1.96]
Peuskens 1997	0.24	0.2999	1.2%	1.27 [0.71; 2.29]
Peuskens 1999	−0.18	0.2889	1.2%	0.83 [0.47; 1.47]
Potkin 2003	0.03	0.2539	1.4%	1.03 [0.63; 1.70]
Potkin 2007c	0.00	0.3684	0.9%	1.00 [0.49; 2.06]
Puech 1998	0.76	0.3494	1.0%	2.14 [1.08; 4.24]
Riedel 2007	0.33	0.5723	0.5%	1.39 [0.45; 4.25]
Rifkin 1984	−0.24	0.5481	0.5%	0.79 [0.27; 2.31]
Rüther 1988	−0.49	0.9968	0.2%	0.62 [0.09; 4.34]
Sacchetti 2008	−0.54	0.7210	0.3%	0.58 [0.14; 2.40]
Sarai 1987	−0.38	0.5430	0.5%	0.68 [0.24; 1.98]
Schennach 2018	0.21	0.4006	0.8%	1.24 [0.56; 2.71]
Selman 1976	0.47	0.5649	0.5%	1.60 [0.53; 4.85]
Sergi 2007	−0.72	0.4858	0.6%	0.49 [0.19; 1.26]
Shah 2011	0.26	0.3799	0.9%	1.30 [0.62; 2.73]
Simpson 2004	−0.48	0.2485	1.4%	0.62 [0.38; 1.01]
Study 049	0.10	0.2926	1.2%	1.11 [0.62; 1.96]
Study 231	−0.37	0.2344	1.5%	0.69 [0.44; 1.09]
Study 233	−0.39	0.2737	1.3%	0.67 [0.39; 1.15]
Study 3000	0.31	0.2621	1.3%	1.36 [0.81; 2.27]
Study 3001	0.31	0.2142	1.6%	1.36 [0.89; 2.07]
Study 3002	0.29	0.2810	1.3%	1.34 [0.77; 2.32]
Study 3003	0.47	0.2964	1.2%	1.59 [0.89; 2.85]
Study 3004	−0.07	0.2308	1.5%	0.93 [0.59; 1.47]
Study 3005	−0.02	0.2089	1.6%	0.98 [0.65; 1.48]
Study 93202 2002	0.12	0.4960	0.6%	1.13 [0.43; 2.99]
Study 94202 2002	0.56	0.3719	0.9%	1.75 [0.84; 3.62]
Tollefson 1997	0.81	0.0972	2.3%	2.25 [1.86; 2.72]
Tuason 1984	0.00	0.5323	0.5%	1.00 [0.35; 2.84]
van der Velde 1975	−0.08	0.8661	0.2%	0.92 [0.17; 5.02]
Wagner 2005	−0.31	0.5563	0.5%	0.73 [0.25; 2.19]
Wetzel 1991	−0.69	0.8097	0.3%	0.50 [0.10; 2.44]
Wetzel 1998	0.60	0.3732	0.9%	1.81 [0.87; 3.77]
Zborowski 1995	−0.35	0.2658	1.3%	0.71 [0.42; 1.19]
Zhang 2011	−0.88	0.4086	0.8%	0.42 [0.19; 0.93]
Zhong 2006	−0.18	0.1545	2.0%	0.83 [0.61; 1.13]
Ziegler 1989	0.00	1.4510	0.1%	1.00 [0.06; 17.18]
Zimbroff 1997	−0.16	0.2389	1.5%	0.86 [0.54; 1.37]
Zimbroff 2007	−0.09	0.2680	1.3%	0.91 [0.54; 1.54]
**Total (95% CI)**			**96.0%**	**1.04 [0.95; 1.14]**
Heterogeneity: $Tau^2 = 0.0711$; $Chi^2 = 217.43$, df = 109 (P < 0.01); $I^2 = 50\%$				
blinding = 0				
Avasthi 2001	1.93	1.2404	0.1%	6.86 [0.60; 77.98]
de Boer 2011	−0.51	0.8028	0.3%	0.60 [0.12; 2.89]
de Oliviera 2009	0.85	0.4762	0.6%	2.33 [0.92; 5.92]
Dossenbach 2007	1.17	0.5796	0.5%	3.21 [1.03; 9.99]
Duggan 2005	0.34	0.4393	0.7%	1.41 [0.59; 3.33]
Ehrlich 2012	−0.69	0.6814	0.4%	0.50 [0.13; 1.90]
Knegtering 2004	.	.	0.0%	
Liemburg 2011	−0.64	0.8117	0.3%	0.53 [0.11; 2.60]
Loza 1999	1.81	1.6665	0.1%	6.11 [0.23; 160.18]
Maat 2014	−0.59	0.4612	0.7%	0.56 [0.22; 1.37]
Taneli 2003	−0.41	0.5681	0.5%	0.66 [0.22; 2.02]
**Total (95% CI)**			**4.0%**	**1.15 [0.68; 1.92]**
Heterogeneity: $Tau^2 = 0.2514$; $Chi^2 = 15.11$, df = 9 (P = 0.09); $I^2 = 40\%$				
**Total (95% CI)**			**100.0%**	**1.04 [0.96; 1.14]**

Heterogeneity: $Tau^2 = 0.0727$; $Chi^2 = 232.63$, df = 119 (P < 0.01); $I^2 = 49\%$
Test for subgroup differences: $Chi^2 = 0.13$, df = 1 (P = 0.72)

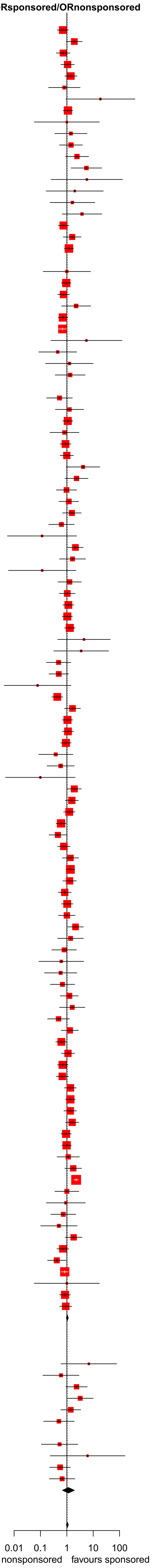

eFigure 8

Use of antiparkinson medication

1. Results of individual comparisons
2. Difference between blinded and open RCTs by recency
3. Difference between blinded and open RCTs by efficacy
4. Differences between blinded and open RCTs sponsored versus non-sponsored drugs

eFigure 8a use of antiparkinson medication individual comparisons

Study or Subgroup	first drug Events	first drug Total	second drug Events	second drug Total	Weight	OR [95% CI]
**blinding = 1**						
Kane 2002	23	204	31	104	32.0%	0.30 [0.16; 0.55]
Study 93202 2002	6	34	19	34	26.3%	0.17 [0.06; 0.51]
Study 94202 2002	21	61	28	63	30.8%	0.66 [0.32; 1.36]
**Total (95% CI)**		**299**		**201**	**89.0%**	**0.35 [0.17; 0.70]**
Heterogeneity: $Tau^2 = 0.2264$; $Chi^2 = 4.77$, df = 2 (P = 0.09); $I^2 = 58\%$						
**blinding = 0**						
McCue2006	0	63	27	61	11.0%	0.01 [0.00; 0.17]
**Total (95% CI)**		**63**		**61**	**11.0%**	**0.01 [0.00; 0.17]**
Heterogeneity: not applicable						
**Total (95% CI)**		**362**		**262**	**100.0%**	**0.23 [0.07; 0.70]**

Heterogeneity: $Tau^2 = 0.9437$; $Chi^2 = 10.85$, df = 3 (P = 0.01); $I^2 = 72\%$

Test for subgroup differences: $Chi^2 = 5.72$, df = 1 (P = 0.02)

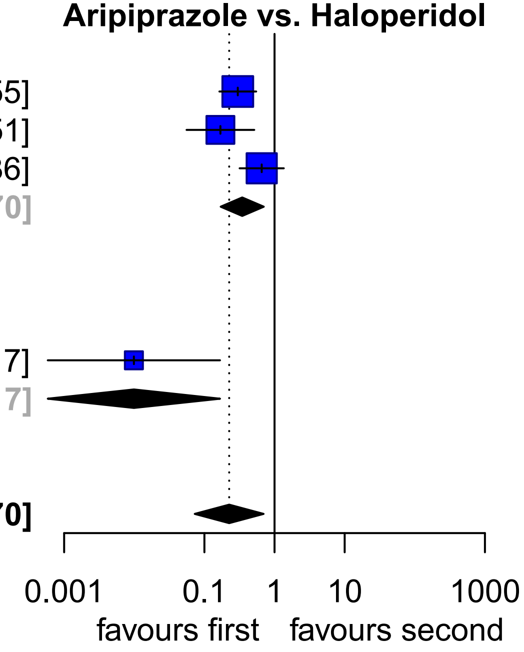

Study or Subgroup	first drug Events	first drug Total	second drug Events	second drug Total	Weight	OR [95% CI]
**blinding = 1**						
Hatta 2009	3	22	2	17	62.2%	1.18 [0.17; 8.02]
Jindal 2013	2	30	1	30	37.8%	2.07 [0.18; 24.15]
**Total (95% CI)**		**52**		**47**	**100.0%**	**1.46 [0.32; 6.62]**
Heterogeneity: Tau^2 = 0; Chi^2 = 0.12, df = 1 (P = 0.72); I^2 = 0%						
**blinding = 0**						
McCue2006	0	63	0	58	0.0%	
**Total (95% CI)**		**63**		**58**	**0.0%**	
Heterogeneity: not applicable						
**Total (95% CI)**		**115**		**105**	**100.0%**	**1.46 [0.32; 6.62]**

Heterogeneity: Tau^2 = 0; Chi^2 = 0.12, df = 1 (P = 0.72); I^2 = 0%

Test for subgroup differences: Chi^2 = 0.00, df = 0 (P = NA)

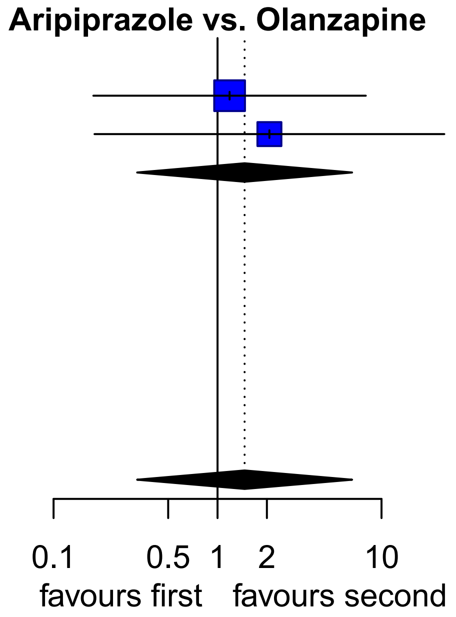

Study or Subgroup	first drug Events	first drug Total	second drug Events	second drug Total	Weight	OR [95% CI]
blinding = 1						
Hatta 2009	3	22	2	20	57.8%	1.42 [0.21; 9.52]
Total (95% CI)		22		20	57.8%	1.42 [0.21; 9.52]
Heterogeneity: not applicable						
blinding = 0						
McCue2006	0	63	5	62	42.2%	0.08 [0.00; 1.52]
Total (95% CI)		63		62	42.2%	0.08 [0.00; 1.52]
Heterogeneity: not applicable						
**Total (95% CI)**		**85**		**82**	**100.0%**	**0.43 [0.03; 6.74]**

Heterogeneity: $Tau^2 = 2.4789$; $Chi^2 = 2.57$, df = 1 (P = 0.11); $I^2 = 61\%$

Test for subgroup differences: $Chi^2 = 2.57$, df = 1 (P = 0.11)

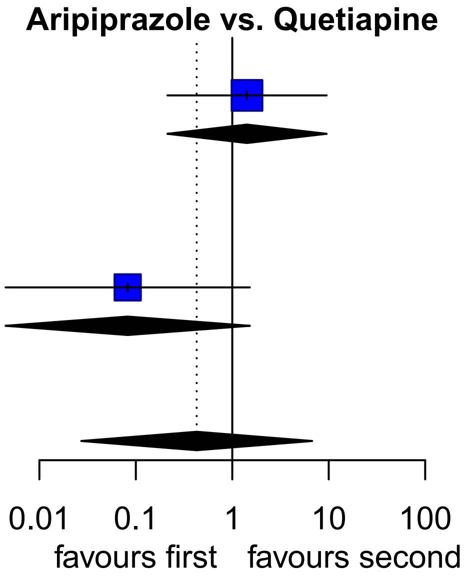

Study or Subgroup	first drug Events	first drug Total	second drug Events	second drug Total	Weight	OR [95% CI]
**blinding = 1**						
Chan 2007	12	49	14	34	52.5%	0.46 [0.18; 1.19]
Hatta 2009	3	22	7	21	33.7%	0.32 [0.07; 1.44]
**Total (95% CI)**		**71**		**55**	**86.2%**	**0.42 [0.19; 0.93]**
Heterogeneity: Tau^2 = 0; Chi^2 = 0.18, df = 1 (P = 0.67); I^2 = 0%						
**blinding = 0**						
McCue2006	0	63	17	65	13.8%	0.02 [0.00; 0.37]
**Total (95% CI)**		**63**		**65**	**13.8%**	**0.02 [0.00; 0.37]**
Heterogeneity: not applicable						
**Total (95% CI)**		**134**		**120**	**100.0%**	**0.27 [0.08; 0.85]**

Heterogeneity: Tau^2 = 0.4300; Chi^2 = 4.02, df = 2 (P = 0.13); I^2 = 50%

Test for subgroup differences: Chi^2 = 3.85, df = 1 (P = 0.05)

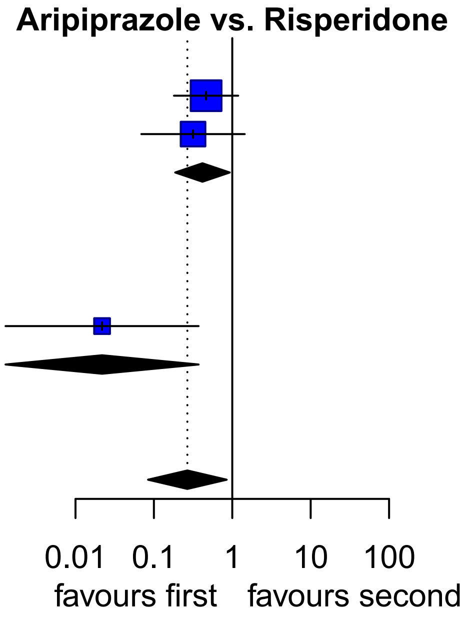

Study or Subgroup	first drug Events	first drug Total	second drug Events	second drug Total	Weight	OR [95% CI]
**blinding = 1**						
Beasley 1996b	47	69	33	133	21.5%	6.47 [3.41; 12.29]
Beasley 1997	39	81	15	175	20.9%	9.90 [4.99; 19.66]
Ishigooka 2001	48	89	36	93	22.1%	1.85 [1.03; 3.34]
Lahti 2009	1	14	1	18	4.8%	1.31 [0.07; 22.93]
Tollefson 1997	315	660	228	1336	25.8%	4.44 [3.60; 5.47]
**Total (95% CI)**		**913**		**1755**	**95.1%**	**4.46 [2.35; 8.46]**
Heterogeneity: $Tau^2 = 0.3676$; $Chi^2 = 15.72$, df = 4 ($P < 0.01$); $I^2 = 75\%$						
**blinding = 0**						
McCue2006	27	61	0	58	4.9%	93.26 [5.51; 1577.76]
**Total (95% CI)**		**61**		**58**	**4.9%**	**93.26 [5.51; 1577.76]**
Heterogeneity: not applicable						
**Total (95% CI)**		**974**		**1813**	**100.0%**	**5.13 [2.58; 10.23]**

Heterogeneity: $Tau^2 = 0.4686$; $Chi^2 = 20.15$, df = 5 ($P < 0.01$); $I^2 = 75\%$

Test for subgroup differences: $Chi^2 = 4.22$, df = 1 ($P = 0.04$)

Haloperidol vs. Olanzapine

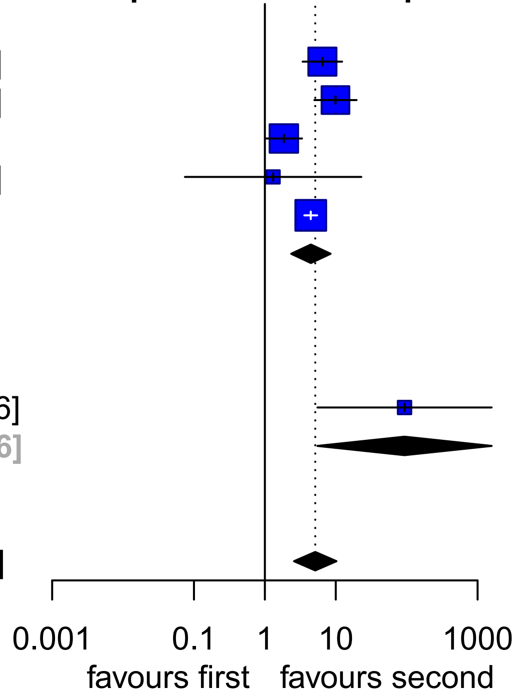

Study or Subgroup	first drug Events	first drug Total	second drug Events	second drug Total	Weight	OR [95% CI]
**blinding = 1**						
Blin 1996	7	20	5	21	2.6%	1.72 [0.44; 6.72]
Borison 1992	29	53	18	53	7.7%	2.35 [1.07; 5.15]
Ceskova 1993	16	31	8	31	4.1%	3.07 [1.05; 8.93]
Chouinard 1993	15	21	7	22	2.8%	5.36 [1.45; 19.74]
Claus 1992	6	22	4	22	2.3%	1.69 [0.40; 7.07]
Janicak 2001	22	32	12	30	4.3%	3.30 [1.16; 9.38]
Marder 1994	31	66	13	64	7.8%	3.47 [1.60; 7.56]
Mesotten 1991	12	32	9	28	4.1%	1.27 [0.44; 3.69]
Murasaki 1993	55	95	43	97	14.5%	1.73 [0.98; 3.06]
Peuskens 1995	67	226	85	457	34.5%	1.84 [1.27; 2.67]
See 1999	8	10	6	10	1.2%	2.67 [0.36; 19.71]
Yen 2004	14	20	9	21	2.8%	3.11 [0.86; 11.29]
**Total (95% CI)**		**628**		**856**	**88.8%**	**2.15 [1.70; 2.70]**
Heterogeneity: $Tau^2 = 0$; $Chi^2 = 7.2$, df = 11 (P = 0.78); $I^2 = 0\%$						
**blinding = 0**						
Fakra 2008	1	15	1	15	0.6%	1.00 [0.06; 17.62]
McCue2006	27	61	17	65	8.4%	2.24 [1.06; 4.74]
Tamrakar 2006	14	18	7	18	2.2%	5.50 [1.28; 23.69]
**Total (95% CI)**		**94**		**98**	**11.2%**	**2.57 [1.34; 4.92]**
Heterogeneity: $Tau^2 = 0$; $Chi^2 = 1.59$, df = 2 (P = 0.45); $I^2 = 0\%$						
**Total (95% CI)**		**722**		**954**	**100.0%**	**2.19 [1.76; 2.72]**
Heterogeneity: $Tau^2 = 0$; $Chi^2 = 9.05$, df = 14 (P = 0.83); $I^2 = 0\%$						
Test for subgroup differences: $Chi^2 = 0.26$, df = 1 (P = 0.61)						

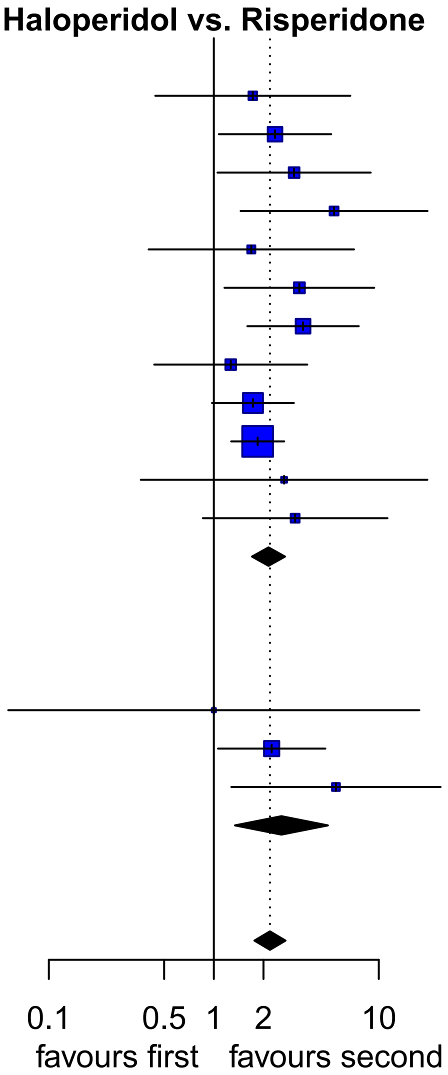

Study or Subgroup	first drug Events	first drug Total	second drug Events	second drug Total	Weight	OR [95% CI]
**blinding = 1**						
Heikkila 1981	12	33	7	30	27.7%	1.88 [0.62; 5.66]
Heikkilae 1992	10	23	8	26	24.6%	1.73 [0.54; 5.59]
Lublin 1991	0	7	0	8	0.0%	
**Total (95% CI)**		**63**		**64**	**52.4%**	**1.81 [0.81; 4.04]**
Heterogeneity: $Tau^2 = 0$; $Chi^2 = 0.01$, df = 1 (P = 0.92); $I^2 = 0\%$						
**blinding = 0**						
Lamure 2003	24	45	18	43	47.6%	1.59 [0.68; 3.69]
**Total (95% CI)**		**45**		**43**	**47.6%**	**1.59 [0.68; 3.69]**
Heterogeneity: not applicable						
**Total (95% CI)**		**108**		**107**	**100.0%**	**1.70 [0.95; 3.04]**
Heterogeneity: $Tau^2 = 0$; $Chi^2 = 0.06$, df = 2 (P = 0.97); $I^2 = 0\%$						
Test for subgroup differences: $Chi^2 = 0.05$, df = 1 (P = 0.83)						

Haloperidol vs. Zuclopenthixol

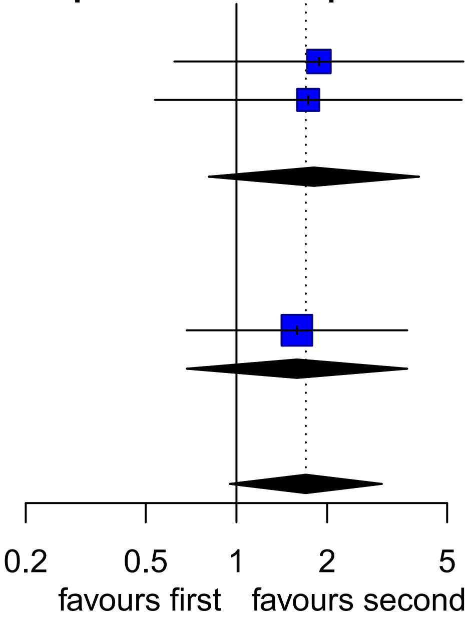

Study or Subgroup	first drug Events	first drug Total	second drug Events	second drug Total	Weight	OR [95% CI]
**blinding = 1**						
Arvanitis 1997	12	105	25	52	20.6%	0.14 [0.06; 0.31]
Copolov 2000	29	221	111	227	35.4%	0.16 [0.10; 0.25]
Murasaki 2001	30	100	55	97	29.4%	0.33 [0.18; 0.59]
**Total (95% CI)**		**426**		**376**	**85.4%**	**0.20 [0.12; 0.33]**
Heterogeneity: $Tau^2 = 0.1151$; $Chi^2 = 4.43$, df = 2 (P = 0.11); $I^2 = 55\%$						
**blinding = 0**						
McCue2006	5	62	27	61	14.6%	0.11 [0.04; 0.31]
**Total (95% CI)**		**62**		**61**	**14.6%**	**0.11 [0.04; 0.31]**
Heterogeneity: not applicable						
**Total (95% CI)**		**488**		**437**	**100.0%**	**0.18 [0.11; 0.29]**

Heterogeneity: $Tau^2 = 0.1018$; $Chi^2 = 5.48$, df = 3 (P = 0.14); $I^2 = 45\%$

Test for subgroup differences: $Chi^2 = 0.94$, df = 1 (P = 0.33)

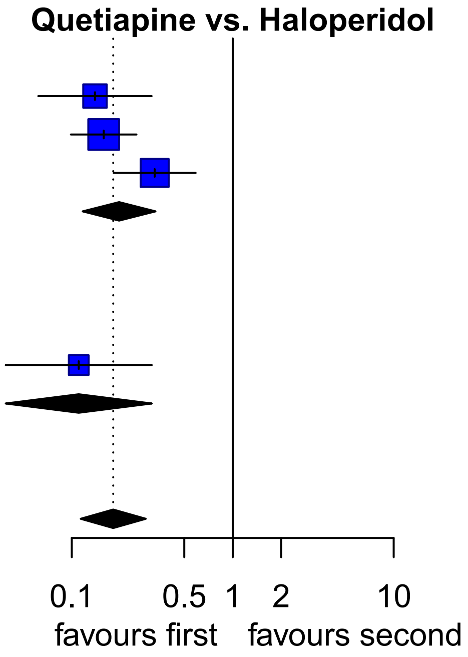

Study or Subgroup	first drug Events	first drug Total	second drug Events	second drug Total	Weight	OR [95% CI]
**blinding = 1**						
Hatta 2009	2	20	2	17	41.1%	0.83 [0.10; 6.65]
Ozguven 2004	1	19	2	15	32.6%	0.36 [0.03; 4.42]
Riedel 2007	0	26	0	26	0.0%	
**Total (95% CI)**		**65**		**58**	**73.7%**	**0.59 [0.12; 2.93]**
Heterogeneity: $Tau^2 = 0$; $Chi^2 = 0.25$, df = 1 (P = 0.61); $I^2 = 0\%$						
**blinding = 0**						
McCue2006	5	62	0	58	26.3%	11.19 [0.60; 207.06]
**Total (95% CI)**		**62**		**58**	**26.3%**	**11.19 [0.60; 207.06]**
Heterogeneity: not applicable						
**Total (95% CI)**		**127**		**116**	**100.0%**	**1.26 [0.22; 7.26]**

Heterogeneity: $Tau^2 = 0.8248$; $Chi^2 = 3.25$, df = 2 (P = 0.20); $I^2 = 38\%$

Test for subgroup differences: $Chi^2 = 3.00$, df = 1 (P = 0.08)

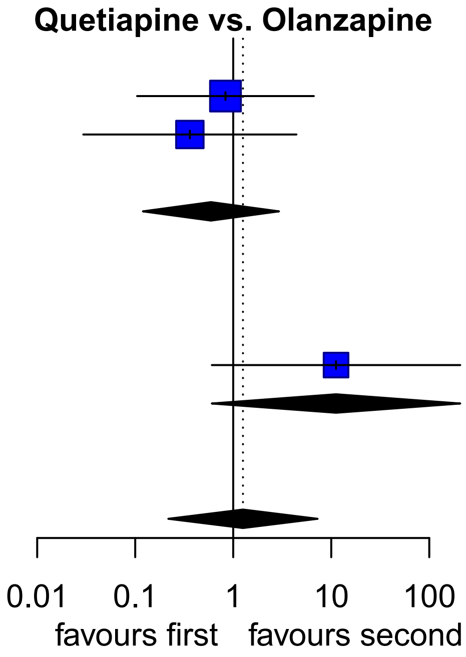

Study or Subgroup	first drug Events	first drug Total	second drug Events	second drug Total	Weight	OR [95% CI]
**blinding = 1**						
Hatta 2009	2	20	7	21	17.8%	0.22 [0.04; 1.24]
Li 2012	3	60	22	59	23.3%	0.09 [0.02; 0.32]
Zhong 2006	19	338	23	335	32.6%	0.81 [0.43; 1.51]
**Total (95% CI)**		**418**		**415**	**73.7%**	**0.28 [0.07; 1.13]**
Heterogeneity: $Tau^2 = 1.1338$; $Chi^2 = 10.17$, df = 2 (P < 0.01); $I^2 = 80\%$						
**blinding = 0**						
McCue2006	5	62	17	65	26.3%	0.25 [0.09; 0.72]
**Total (95% CI)**		**62**		**65**	**26.3%**	**0.25 [0.09; 0.72]**
Heterogeneity: not applicable						
**Total (95% CI)**		**480**		**480**	**100.0%**	**0.28 [0.10; 0.77]**

Heterogeneity: $Tau^2 = 0.7029$; $Chi^2 = 11.37$, df = 3 (P < 0.01); $I^2 = 74\%$

Test for subgroup differences: $Chi^2 = 0.02$, df = 1 (P = 0.89)

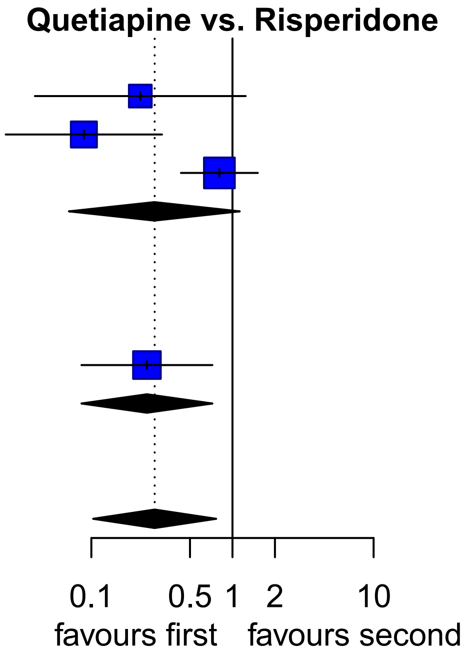

Study or Subgroup	first drug Events	first drug Total	second drug Events	second drug Total	Weight	OR [95% CI]
**blinding = 1**						
Conley 2001	61	188	53	189	34.4%	1.23 [0.79; 1.91]
Hatta 2009	7	21	2	17	18.3%	3.75 [0.66; 21.20]
Hatta 2013	9	20	2	22	18.7%	8.18 [1.50; 44.77]
**Total (95% CI)**		**229**		**228**	**71.4%**	**2.64 [0.80; 8.72]**
Heterogeneity: Tau^2 = 0.7004; Chi^2 = 5.66, df = 2 (P = 0.06); I^2 = 65%						
**blinding = 0**						
McCue2006	17	65	0	58	10.0%	42.22 [2.47; 720.24]
van Bruggen 2003	7	26	2	18	18.6%	2.95 [0.54; 16.24]
**Total (95% CI)**		**91**		**76**	**28.6%**	**8.68 [0.67; 112.45]**
Heterogeneity: Tau^2 = 2.1164; Chi^2 = 2.48, df = 1 (P = 0.12); I^2 = 60%						
**Total (95% CI)**		**320**		**304**	**100.0%**	**3.60 [1.26; 10.31]**
Heterogeneity: Tau^2 = 0.7871; Chi^2 = 11.42, df = 4 (P = 0.02); I^2 = 65%						
Test for subgroup differences: Chi^2 = 0.68, df = 1 (P = 0.41)						

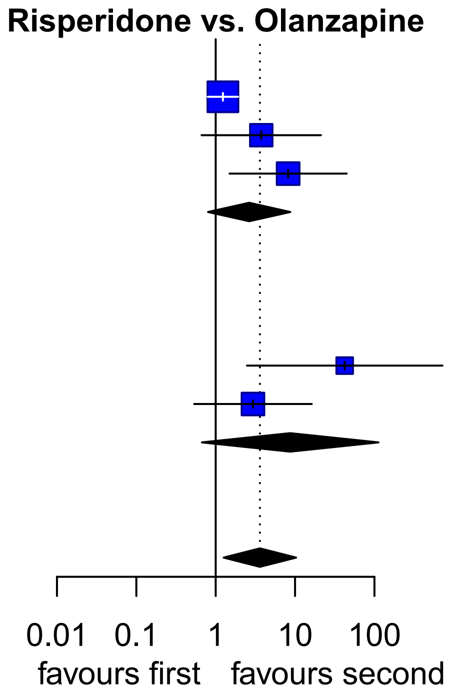

Study or Subgroup	first drug Events	first drug Total	second drug Events	second drug Total	Weight	OR [95% CI]
**blinding = 1**						
Brook 2005	94	429	84	138	36.1%	0.18 [0.12; 0.27]
Goff 1998	3	20	9	17	11.9%	0.16 [0.03; 0.74]
Study 115 2000	51	164	43	85	32.5%	0.44 [0.26; 0.76]
**Total (95% CI)**		**613**		**240**	**80.4%**	**0.25 [0.13; 0.51]**
Heterogeneity: $Tau^2 = 0.2403$; $Chi^2 = 7.01$, df = 2 (P = 0.03); $I^2 = 71\%$						
**blinding = 0**						
McCue2006	5	59	27	61	19.6%	0.12 [0.04; 0.33]
**Total (95% CI)**		**59**		**61**	**19.6%**	**0.12 [0.04; 0.33]**
Heterogeneity: not applicable						
**Total (95% CI)**		**672**		**301**	**100.0%**	**0.22 [0.12; 0.41]**

Heterogeneity: $Tau^2 = 0.2427$; $Chi^2 = 8.80$, df = 3 (P = 0.03); $I^2 = 66\%$

Test for subgroup differences: $Chi^2 = 1.46$, df = 1 (P = 0.23)

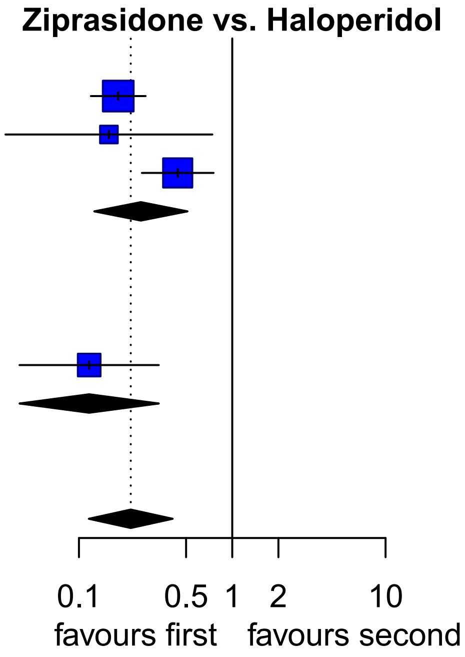

Study or Subgroup	first drug Events	first drug Total	second drug Events	second drug Total	Weight	OR [95% CI]
**blinding = 1**						
Grootens 2009	17	39	7	35	24.9%	3.09 [1.09; 8.77]
Simpson 2004	34	136	20	133	71.9%	1.88 [1.02; 3.48]
**Total (95% CI)**		**175**		**168**	**96.8%**	**2.14 [1.26; 3.63]**
Heterogeneity: $Tau^2 = 0$; $Chi^2 = 0.64$, df = 1 (P = 0.42); $I^2 = 0\%$						
**blinding = 0**						
McCue2006	5	59	0	58	3.2%	11.81 [0.64; 218.59]
**Total (95% CI)**		**59**		**58**	**3.2%**	**11.81 [0.64; 218.59]**
Heterogeneity: not applicable						
**Total (95% CI)**		**234**		**226**	**100.0%**	**2.26 [1.34; 3.80]**

Heterogeneity: $Tau^2 < 0.0001$; $Chi^2 = 1.92$, df = 2 (P = 0.38); $I^2 = 0\%$
Test for subgroup differences: $Chi^2 = 1.27$, df = 1 (P = 0.26)

Ziprasidone vs. Olanzapine

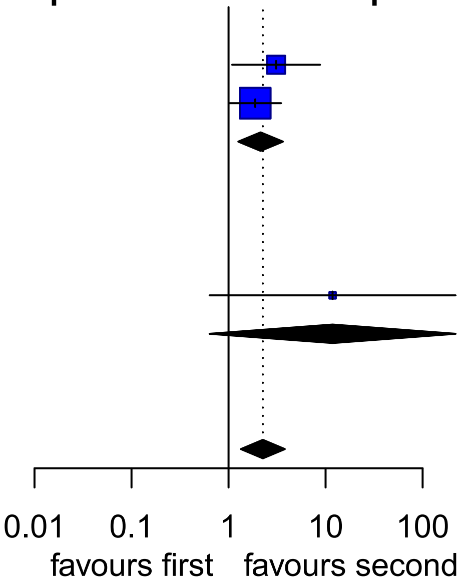

Study or Subgroup	first drug Events	first drug Total	second drug Events	second drug Total	Weight	OR [95% CI]
**blinding = 1**						
Addington 2004	33	149	49	147	32.9%	0.57 [0.34; 0.95]
Zhang 2011	62	118	56	121	33.1%	1.29 [0.77; 2.14]
**Total (95% CI)**		**267**		**268**	**66.0%**	**0.86 [0.39; 1.90]**
Heterogeneity: $Tau^2 = 0.2635$; $Chi^2 = 4.85$, df = 1 (P = 0.03); $I^2 = 79\%$						
**blinding = 0**						
McCue2006	5	59	17	65	21.6%	0.26 [0.09; 0.76]
Sonmez 2009	4	11	7	11	12.4%	0.33 [0.06; 1.86]
**Total (95% CI)**		**70**		**76**	**34.0%**	**0.28 [0.11; 0.69]**
Heterogeneity: $Tau^2 = 0$; $Chi^2 = 0.05$, df = 1 (P = 0.83); $I^2 = 0\%$						
**Total (95% CI)**		**337**		**344**	**100.0%**	**0.59 [0.28; 1.23]**

Heterogeneity: $Tau^2 = 0.3647$; $Chi^2 = 10.01$, df = 3 (P = 0.02); $I^2 = 70\%$

Test for subgroup differences: $Chi^2 = 3.31$, df = 1 (P = 0.07)

Ziprasidone vs. Risperidone

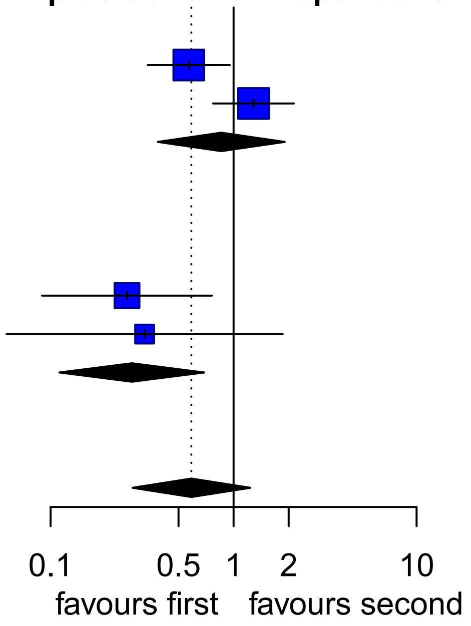

eFigure 8b use of antiparkinson medication ratio of blinded and open trials, more recent drug listed first

Study	TE	SE	Weight	OR [95% CI]
Aripiprazole vs. Haloperidol, (nbl=500, nop=124)	3.56	1.4870	2.4%	35.05 [1.90; 646.22]
Zuclopenthixol vs. Haloperidol, (nbl=127, nop=88)	−0.13	0.5941	13.0%	0.88 [0.27; 2.81]
Quetiapine vs. Risperidone, (nbl=833, nop=127)	0.12	0.8962	6.3%	1.13 [0.19; 6.54]
Quetiapine vs. Olanzapine, (nbl=123, nop=120)	−2.94	1.6975	1.8%	0.05 [0.00; 1.48]
Aripiprazole vs. Quetiapine, (nbl=42, nop=125)	2.85	1.7766	1.7%	17.26 [0.53; 561.56]
Aripiprazole vs. Olanzapine, (nbl=99, nop=121)	.	.	0.0%	
Ziprasidone vs. Olanzapine, (nbl=343, nop=117)	−1.71	1.5133	2.3%	0.18 [0.01; 3.52]
Olanzapine vs. Risperidone, (nbl=457, nop=167)	1.19	1.4420	2.5%	3.29 [0.19; 55.47]
Aripiprazole vs. Risperidone, (nbl=126, nop=128)	2.95	1.5036	2.3%	19.08 [1.00; 363.45]
Ziprasidone vs. Haloperidol, (nbl=853, nop=120)	0.78	0.6423	11.4%	2.17 [0.62; 7.66]
Risperidone vs. Haloperidol, (nbl=1484, nop=192)	0.18	0.3516	28.7%	1.20 [0.60; 2.38]
Olanzapine vs. Haloperidol, (nbl=2668, nop=119)	3.04	1.4796	2.4%	20.93 [1.15; 380.28]
Quetiapine vs. Haloperidol, (nbl=802, nop=123)	0.58	0.5954	13.0%	1.78 [0.55; 5.72]
Ziprasidone vs. Risperidone, (nbl=535, nop=146)	1.13	0.6181	12.2%	3.08 [0.92; 10.35]
**Total (95% CI)**			**100.0%**	**1.74 [1.10; 2.75]**

Heterogeneity: $Tau^2 = 0.0662$; $Chi^2 = 21.39$, df = 12 (P = 0.04); $I^2 = 44\%$

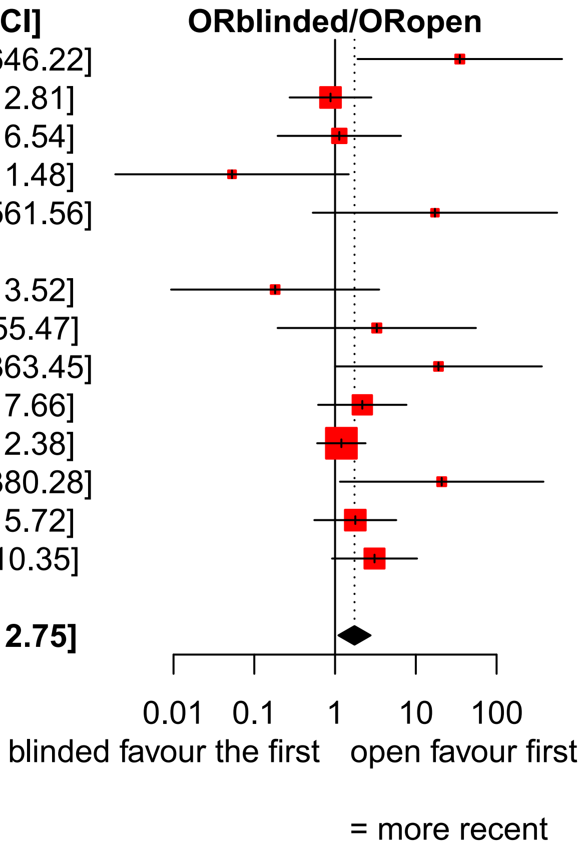

eFigure 8c use of antiparkinson medication ratio of blinded and open trials, drug with more EPS according to Huhn et al. 2019 listed first

Study	logOR	SE	Weight	OR [95% CI]
Haloperidol vs. Aripiprazole, (nbl=500, nop=124)	−3.5567	1.4870	2.7%	0.03 [0.00; 0.53]
Haloperidol vs. Zuclopenthixol, (nbl=127, nop=88)	0.1297	0.5941	13.2%	1.14 [0.36; 3.65]
Risperidone vs. Quetiapine, (nbl=833, nop=127)	−0.1208	0.8962	6.7%	0.89 [0.15; 5.13]
Quetiapine vs. Olanzapine, (nbl=123, nop=120)	−2.9381	1.6975	2.1%	0.05 [0.00; 1.48]
Aripiprazole vs. Quetiapine, (nbl=42, nop=125)	2.8485	1.7766	1.9%	17.26 [0.53; 561.56]
Aripiprazole vs. Olanzapine, (nbl=99, nop=121)	.	.	0.0%	
Ziprasidone vs. Olanzapine, (nbl=343, nop=117)	−1.7081	1.5133	2.6%	0.18 [0.01; 3.52]
Risperidone vs. Olanzapine, (nbl=457, nop=167)	−1.1895	1.4420	2.8%	0.30 [0.02; 5.14]
Risperidone vs. Aripiprazole, (nbl=126, nop=128)	−2.9487	1.5036	2.6%	0.05 [0.00; 1.00]
Haloperidol vs. Ziprasidone, (nbl=853, nop=120)	−0.7770	0.6423	11.7%	0.46 [0.13; 1.62]
Haloperidol vs. Risperidone, (nbl=1484, nop=192)	−0.1801	0.3516	25.6%	0.84 [0.42; 1.66]
Haloperidol vs. Olanzapine, (nbl=2668, nop=119)	−3.0410	1.4796	2.7%	0.05 [0.00; 0.87]
Haloperidol vs. Quetiapine, (nbl=802, nop=123)	−0.5775	0.5954	13.1%	0.56 [0.17; 1.80]
Risperidone vs. Ziprasidone, (nbl=535, nop=146)	−1.1253	0.6181	12.4%	0.32 [0.10; 1.09]
**Total (95% CI)**			**100.0%**	**0.51 [0.31; 0.82]**

Heterogeneity: $Tau^2 = 0.1193$; $Chi^2 = 19.50$, df = 12 (P = 0.08); $I^2 = 38\%$

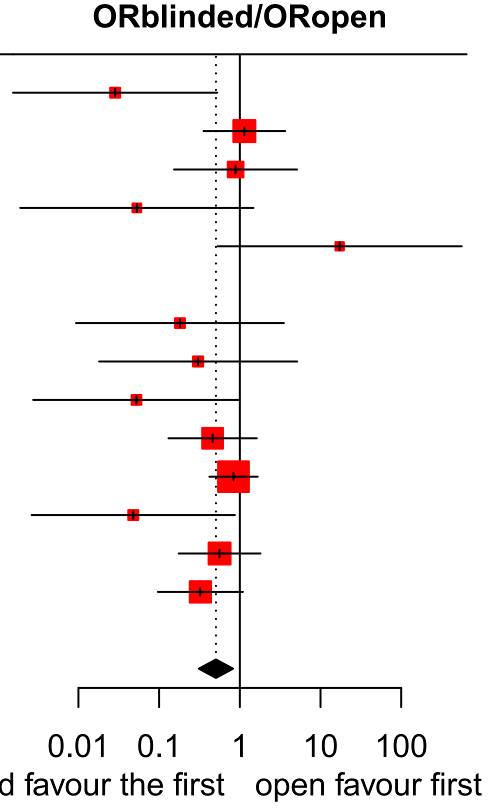

eFigure 8d use of antiparkinson medication sponsored vs non sponsored drugs n blinded versus open trials

Study or Subgroup	TE	SE	Weight	OR [95% CI]
**blinding = 1**				
Addington 2004	0.56	0.2637	1.9%	1.76 [1.05; 2.95]
Arvanitis 1997	1.97	0.4137	1.7%	7.18 [3.19; 16.14]
Beasley 1996b	1.87	0.3272	1.8%	6.47 [3.41; 12.29]
Beasley 1997	2.29	0.3498	1.8%	9.90 [4.99; 19.66]
Blin 1996	−0.82	0.7016	1.2%	0.44 [0.11; 1.74]
Brook 2005	1.71	0.2099	1.9%	5.54 [3.67; 8.36]
Chan 2007	0.77	0.4814	1.6%	2.16 [0.84; 5.55]
Chouinard 1993	1.68	0.6655	1.3%	5.36 [1.45; 19.74]
Clark 1975	0.00	0.9129	1.0%	1.00 [0.17; 5.98]
Claus 1992	0.52	0.7312	1.2%	1.69 [0.40; 7.07]
Conley 2001	−0.21	0.2247	1.9%	0.81 [0.52; 1.26]
Cooper 2000a	0.00	0.7354	1.2%	1.00 [0.24; 4.23]
Copolov 2000	1.85	0.2394	1.9%	6.34 [3.96; 10.13]
Costa e Silva 1989	2.33	1.1280	0.8%	10.23 [1.12; 93.34]
Cutler 2008	0.29	0.2878	1.8%	1.33 [0.76; 2.34]
Dieterle 1991	−0.64	0.8117	1.1%	0.53 [0.11; 2.60]
Freeman 1969	−1.35	0.8909	1.0%	0.26 [0.05; 1.49]
Gattaz 2004	2.79	1.1751	0.7%	16.33 [1.63; 163.44]
Goff 1998	1.85	0.7926	1.1%	6.37 [1.35; 30.14]
Grootens 2009	−1.13	0.5318	1.5%	0.32 [0.11; 0.92]
Hale 2000	1.70	0.2612	1.9%	5.48 [3.28; 9.14]
Harnryd 1984	0.00	0.8704	1.0%	1.00 [0.18; 5.51]
Hoyberg 1993	0.26	0.4232	1.7%	1.30 [0.57; 2.97]
Huttunen 1995	1.07	0.4308	1.6%	2.92 [1.25; 6.79]
Hwang 2003	0.59	0.6064	1.4%	1.80 [0.55; 5.89]
Ishigooka 2001	0.62	0.3009	1.8%	1.85 [1.03; 3.34]
Itil 1971	−0.44	0.9519	0.9%	0.64 [0.10; 4.15]
Itoh 1977	1.12	0.4577	1.6%	3.06 [1.25; 7.51]
Janicak 2001	1.19	0.5332	1.5%	3.30 [1.16; 9.38]
Janssen CR012625	−0.81	0.6481	1.3%	0.45 [0.13; 1.59]
Kane 2002	1.21	0.3082	1.8%	3.34 [1.83; 6.11]
Kane 2007b	−0.89	0.3568	1.8%	0.41 [0.20; 0.83]
Kane 2010a	1.24	0.2583	1.9%	3.45 [2.08; 5.72]
Kluge 2007	−1.75	1.5933	0.5%	0.17 [0.01; 3.96]
Li 2012	2.42	0.6507	1.3%	11.30 [3.16; 40.44]
Lin 2003	−2.87	1.4778	0.5%	0.06 [0.00; 1.03]
Lundbeck NCT00864045	−0.55	0.2981	1.8%	0.58 [0.32; 1.04]
Marder 1994	1.25	0.3967	1.7%	3.47 [1.60; 7.56]
Marder 2007c	−0.06	0.3578	1.7%	0.94 [0.47; 1.89]
Mesotten 1991	0.24	0.5450	1.5%	1.27 [0.44; 3.69]
Möller 1997	1.19	0.3027	1.8%	3.28 [1.81; 5.93]
Murasaki 2001	1.12	0.2993	1.8%	3.06 [1.70; 5.49]
Nishizono 1994	−0.94	0.3843	1.7%	0.39 [0.18; 0.83]
Petit 1996	1.05	0.3838	1.7%	2.85 [1.34; 6.04]
Peuskens 1995	0.61	0.1889	2.0%	1.84 [1.27; 2.67]
Peuskens 1997	0.47	0.4352	1.6%	1.61 [0.68; 3.77]
Peuskens 1999	−0.38	0.3017	1.8%	0.68 [0.38; 1.23]
Potkin 2007c	0.76	0.4465	1.6%	2.14 [0.89; 5.14]
Puech 1998	1.00	0.3361	1.8%	2.71 [1.40; 5.24]
Riedel 2007	.	.	0.0%	
Rüther 1988	−0.69	0.8466	1.1%	0.50 [0.10; 2.63]
Simpson 2004	−0.63	0.3132	1.8%	0.53 [0.29; 0.98]
Study 049	1.14	0.3167	1.8%	3.12 [1.68; 5.81]
Study 231	−0.68	0.2743	1.9%	0.51 [0.30; 0.86]
Study 233	−0.68	0.3597	1.7%	0.50 [0.25; 1.02]
Study 93202 2002	1.78	0.5672	1.4%	5.91 [1.94; 17.97]
Study 94202 2002	0.42	0.3700	1.7%	1.52 [0.74; 3.15]
Tollefson 1997	1.49	0.1066	2.0%	4.44 [3.60; 5.47]
Weston 1973	−1.15	0.8473	1.1%	0.32 [0.06; 1.67]
Wetzel 1991	−0.05	1.4500	0.5%	0.95 [0.06; 16.29]
Wetzel 1998	0.75	0.3554	1.8%	2.11 [1.05; 4.24]
Zborowski 1995	1.58	0.3283	1.8%	4.86 [2.55; 9.24]
Zhang 2011	−0.25	0.2593	1.9%	0.78 [0.47; 1.29]
Zhong 2006	0.21	0.3201	1.8%	1.24 [0.66; 2.32]
Ziegler 1989	3.15	1.1201	0.8%	23.22 [2.59; 208.61]
Zimbroff 1997	1.75	0.2955	1.8%	5.76 [3.23; 10.28]
**Total (95% CI)**			**98.4%**	**1.83 [1.43; 2.34]**
Heterogeneity: $Tau^2 = 0.7656$; $Chi^2 = 435.49$, df = 64 ($P < 0.01$); $I^2 = 85\%$				
**blinding = 0**				
Dossenbach 2007	−2.33	1.4640	0.5%	0.10 [0.01; 1.71]
Loza 1999	.	.	0.0%	
van Bruggen 2003	1.08	0.8706	1.0%	2.95 [0.54; 16.24]
**Total (95% CI)**			**1.6%**	**0.65 [0.02; 18.17]**
Heterogeneity: $Tau^2 = 4.3833$; $Chi^2 = 4.02$, df = 1 ($P = 0.04$); $I^2 = 75\%$				
**Total (95% CI)**			**100.0%**	**1.81 [1.42; 2.31]**
Heterogeneity: $Tau^2 = 0.7684$; $Chi^2 = 440.18$, df = 66 ($P < 0.01$); $I^2 = 85\%$				
Test for subgroup differences: $Chi^2 = 0.37$, df = 1 ($P = 0.55$)				

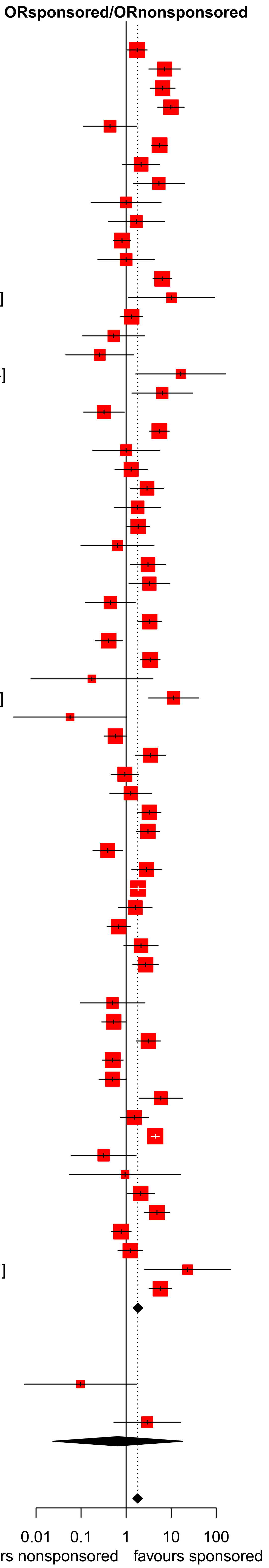

eFigure 9

Sedation

1. Results of individual comparisons
2. Difference between blinded and open RCTs by recency
3. Difference between blinded and open RCTs by efficacy
4. Differences between blinded and open RCTs sponsored versus non-sponsored drugs

eFigure 9a sedation individual comparisons

Study or Subgroup	first drug Events	first drug Total	second drug Events	second drug Total	Weight	OR [95% CI]	Brexpiprazole vs. Aripiprazole
**blinding = 0**							
Citrome 2015	0	64	2	33	32.7%	0.10 [0.00; 2.10]	
**blinding = 1**							
NCT00905307	3	90	2	50	67.3%	0.83 [0.13; 5.13]	
**Total (95% CI)**		**154**		**83**	**100.0%**	**0.41 [0.06; 2.94]**	

Heterogeneity: $Tau^2 = 0.6267$; $Chi^2 = 1.38$, df = 1 (P = 0.24); $I^2 = 27\%$
Test for subgroup differences: $Chi^2 = 1.38$, df = 1 (P = 0.24)

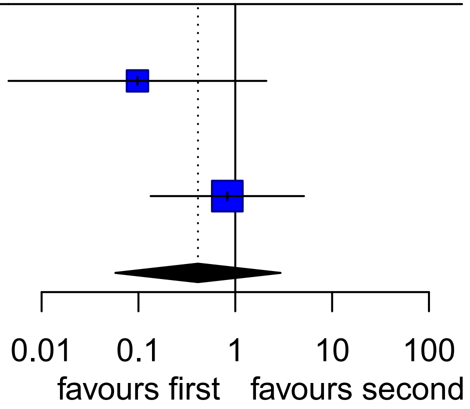

Study or Subgroup	first drug Events	first drug Total	second drug Events	second drug Total	Weight	OR [95% CI]
**blinding = 0**						
Avasthi 2001	2	10	8	17	1.6%	0.28 [0.05; 1.73]
**blinding = 1**						
Beasley 1996b	24	69	46	133	14.1%	1.01 [0.55; 1.86]
Beasley 1997	8	81	12	175	6.0%	1.49 [0.58; 3.80]
Ishigooka 2001	6	89	9	93	4.6%	0.67 [0.23; 1.98]
Tollefson 1997	88	660	216	1336	73.7%	0.80 [0.61; 1.04]
**Total (95% CI)**		**899**		**1737**	**98.4%**	**0.85 [0.67; 1.07]**
Heterogeneity: $Tau^2 = 0$; $Chi^2 = 2.07$, df = 3 (P = 0.56); $I^2 = 0\%$						
**Total (95% CI)**		**909**		**1754**	**100.0%**	**0.84 [0.66; 1.05]**

Heterogeneity: $Tau^2 < 0.0001$; $Chi^2 = 3.47$, df = 4 (P = 0.48); $I^2 = 0\%$

Test for subgroup differences: $Chi^2 = 1.40$, df = 1 (P = 0.24)

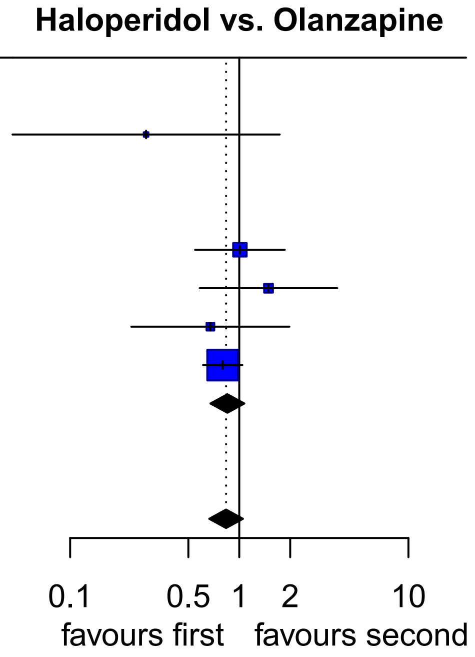

Study or Subgroup	first drug Events	first drug Total	second drug Events	second drug Total	Weight	OR [95% CI]
**blinding = 1**						
Arvanitis 1997	11	105	3	52	10.2%	1.91 [0.51; 7.17]
Copolov 2000	44	221	23	227	60.2%	2.20 [1.28; 3.79]
Murasaki 2001	19	100	11	97	27.6%	1.83 [0.82; 4.09]
**Total (95% CI)**		**426**		**376**	**98.0%**	**2.06 [1.35; 3.16]**
Heterogeneity: $Tau^2 = 0$; $Chi^2 = 0.15$, df = 2 (P = 0.93); $I^2 = 0\%$						
**blinding = 0**						
Taneli 2003	4	45	0	34	2.0%	7.48 [0.39; 143.87]
**Total (95% CI)**		**471**		**410**	**100.0%**	**2.12 [1.39; 3.23]**

Heterogeneity: $Tau^2 = 0$; $Chi^2 = 0.87$, df = 3 (P = 0.83); $I^2 = 0\%$
Test for subgroup differences: $Chi^2 = 0.71$, df = 1 (P = 0.40)

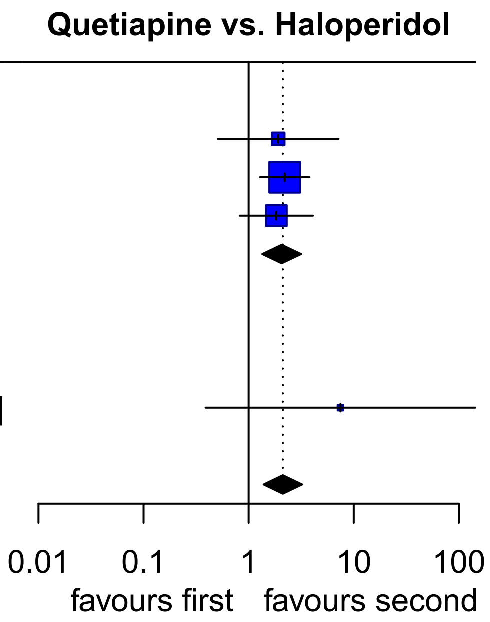

Study or Subgroup	first drug Events	first drug Total	second drug Events	second drug Total	Weight	OR [95% CI]
**blinding = 1**						
Conley 2001	69	188	73	189	40.8%	0.92 [0.61; 1.40]
Hatta 2009	0	21	0	17	0.0%	
Safa 2008	0	31	7	32	24.1%	0.05 [0.00; 0.99]
**Total (95% CI)**		**240**		**238**	**64.9%**	**0.33 [0.02; 4.75]**
Heterogeneity: $Tau^2 = 2.9009$; $Chi^2 = 3.58$, df = 1 (P = 0.06); $I^2 = 72\%$						
**blinding = 0**						
van Bruggen 2003	13	26	3	18	35.1%	5.00 [1.16; 21.50]
**Total (95% CI)**		**266**		**256**	**100.0%**	**0.84 [0.09; 7.69]**

Heterogeneity: $Tau^2 = 3.0788$; $Chi^2 = 8.69$, df = 2 (P = 0.01); $I^2 = 77\%$

Test for subgroup differences: $Chi^2 = 3.08$, df = 1 (P = 0.08)

Risperidone vs. Olanzapine

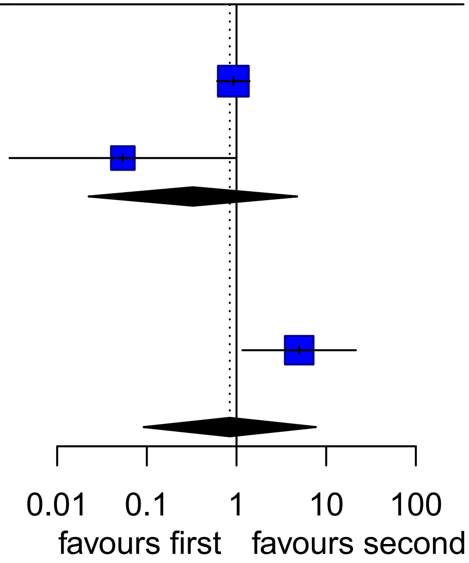

eFigure 9b sedation ratio of blinded and open trials, more recent drug listed first

Study	logOR	SE	Weight	OR [95% CI]
Olanzapine vs. Risperidone, (nbl=478, nop=44)	2.7296	1.5562	23.0%	15.33 [0.73; 323.69]
Brexpiprazole vs. Aripiprazole, (nbl=140, nop=97)	2.1369	1.8201	19.4%	8.47 [0.24; 300.15]
Olanzapine vs. Haloperidol, (nbl=2636, nop=27)	−1.1066	0.9354	34.0%	0.33 [0.05; 2.07]
Quetiapine vs. Haloperidol, (nbl=802, nop=79)	−1.2885	1.5240	23.5%	0.28 [0.01; 5.47]
**Total (95% CI)**			**100.0%**	**1.44 [0.18; 11.33]**

Heterogeneity: $Tau^2 = 2.3814$; $Chi^2 = 6.57$, df = 3 (P = 0.09); $I^2 = 54\%$

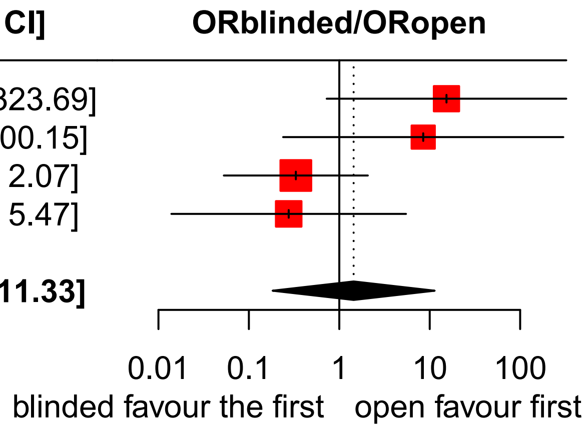

eFigure 9c sedation ratio of blinded and open trials, more sedation drug according to Huhn et al. 2019 listed first

Study	logOR	SE	Weight	OR [95% CI]
Olanzapine vs. Risperidone, (nbl=478, nop=44)	2.7296	1.5562	23.0%	15.33 [0.73; 323.69]
Brexpiprazole vs. Aripiprazole, (nbl=140, nop=97)	2.1369	1.8201	19.4%	8.47 [0.24; 300.15]
Olanzapine vs. Haloperidol, (nbl=2636, nop=27)	−1.1066	0.9354	34.0%	0.33 [0.05; 2.07]
Quetiapine vs. Haloperidol, (nbl=802, nop=79)	−1.2885	1.5240	23.5%	0.28 [0.01; 5.47]
**Total (95% CI)**			**100.0%**	**1.44 [0.18; 11.33]**

Heterogeneity: $Tau^2 = 2.3814$; $Chi^2 = 6.57$, df = 3 (P = 0.09); $I^2 = 54\%$

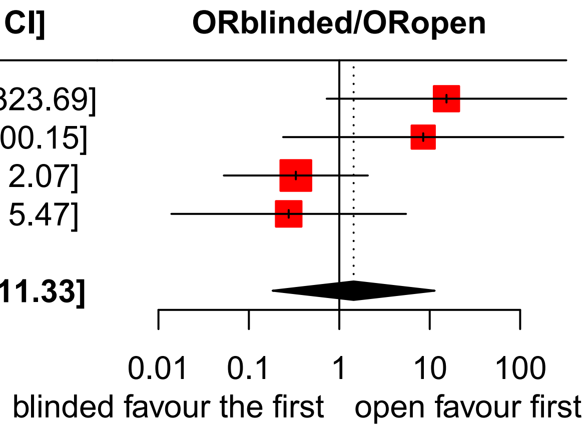

eFigure 9d sedation sponsored vs non sponsored drugs and blinded versus open trials

Study or Subgroup	logOR	SE	Weight	OR [95% CI]
**blinding = 1**				
A1281046	−0.7295	1.2541	0.3%	0.48 [0.04; 5.63]
A1281050	−0.9928	0.4157	1.5%	0.37 [0.16; 0.84]
Addington 2004	−0.2010	0.2957	1.9%	0.82 [0.46; 1.46]
Arvanitis 1997	−0.6478	0.6748	0.9%	0.52 [0.14; 1.96]
Azorin 2006	−0.0404	0.5396	1.2%	0.96 [0.33; 2.77]
Beasley 1996b	0.0087	0.3116	1.9%	1.01 [0.55; 1.86]
Beasley 1997	0.3978	0.4777	1.3%	1.49 [0.58; 3.80]
Blin 1996	−0.9878	0.5808	1.1%	0.37 [0.12; 1.16]
Brook 2005	−0.8288	0.3927	1.6%	0.44 [0.20; 0.94]
Chiu 1976	−0.1446	0.6650	0.9%	0.87 [0.24; 3.19]
Clark 1970a	2.7726	1.1573	0.4%	16.00 [1.66; 154.59]
Clark 1972	0.7138	0.7400	0.8%	2.04 [0.48; 8.71]
Clark 1975	−0.6931	0.8466	0.6%	0.50 [0.10; 2.63]
Claus 1992	−0.3944	0.6305	1.0%	0.67 [0.20; 2.32]
Conley 2001	0.0819	0.2126	2.2%	1.09 [0.72; 1.65]
Cooper 2000a	−0.0880	0.4197	1.5%	0.92 [0.40; 2.08]
Copolov 2000	−0.7907	0.2771	2.0%	0.45 [0.26; 0.78]
Costa e Silva 1989	−1.1486	1.6633	0.2%	0.32 [0.01; 8.26]
Cutler 2008	0.9552	0.2521	2.1%	2.60 [1.59; 4.26]
Davidson 2007	0.3464	0.3558	1.7%	1.41 [0.70; 2.84]
Dieterle 1991	−0.2136	0.6543	0.9%	0.81 [0.22; 2.91]
Durgam 2014	0.7152	0.3338	1.8%	2.04 [1.06; 3.93]
Freeman 1969	0.3483	0.8394	0.6%	1.42 [0.27; 7.34]
Gattaz 2004	−1.0272	1.6762	0.2%	0.36 [0.01; 9.57]
Gerlach 1975	−2.1972	1.7385	0.2%	0.11 [0.00; 3.35]
Goff 1998	−0.2803	0.9792	0.5%	0.76 [0.11; 5.15]
Grootens 2009	−0.4297	0.4684	1.3%	0.65 [0.26; 1.63]
Guirguis 1977	−0.3567	0.6094	1.0%	0.70 [0.21; 2.31]
Hale 2000	0.8568	0.4327	1.5%	2.36 [1.01; 5.50]
Harnryd 1984	0.8165	0.5796	1.1%	2.26 [0.73; 7.05]
Honigfeld 1984d	−0.9691	0.2758	2.0%	0.38 [0.22; 0.65]
Hoyberg 1993	−0.7985	0.4291	1.5%	0.45 [0.19; 1.04]
Huttunen 1995	1.1051	0.5387	1.2%	3.02 [1.05; 8.68]
Hwang 2001	−0.6061	0.5578	1.1%	0.55 [0.18; 1.63]
Ishigooka 2001	−0.3935	0.5493	1.1%	0.67 [0.23; 1.98]
Itoh 1977	−0.7436	0.4697	1.3%	0.48 [0.19; 1.19]
Janssen CR012625	2.2076	0.7011	0.8%	9.09 [2.30; 35.93]
Kane 2002	0.5878	0.3998	1.6%	1.80 [0.82; 3.94]
Kane 2007b	0.9180	0.3331	1.8%	2.50 [1.30; 4.81]
Kane 2010a	−0.5555	0.5837	1.1%	0.57 [0.18; 1.80]
Kingstone 1970	−1.1939	0.6764	0.9%	0.30 [0.08; 1.14]
Kluge 2007	−0.4855	0.9968	0.5%	0.62 [0.09; 4.34]
Kramer 1978	0.0382	0.5525	1.1%	1.04 [0.35; 3.07]
Li 2012	−1.0549	0.7039	0.8%	0.35 [0.09; 1.38]
Lundbeck NCT00864045	0.9584	0.6004	1.0%	2.61 [0.80; 8.46]
Marder 1994	0.3895	0.9302	0.6%	1.48 [0.24; 9.14]
Marder 2007c	0.8858	0.2904	1.9%	2.42 [1.37; 4.28]
Mesotten 1991	−0.1603	0.6932	0.9%	0.85 [0.22; 3.31]
Min 1993	−0.7783	0.7248	0.8%	0.46 [0.11; 1.90]
Möller 1997	1.6419	1.1053	0.4%	5.16 [0.59; 45.07]
Murasaki 2001	−0.6064	0.4093	1.5%	0.55 [0.24; 1.22]
NCT00882518	−1.3597	0.5723	1.1%	0.26 [0.08; 0.79]
NCT01617187	0.6134	0.6137	1.0%	1.85 [0.55; 6.15]
NCT01810380	0.6931	0.6235	1.0%	2.00 [0.59; 6.79]
Petit 1996	−0.7094	1.2380	0.3%	0.49 [0.04; 5.57]
Peuskens 1995	0.3048	0.1689	2.4%	1.36 [0.97; 1.89]
Peuskens 1997	0.1686	0.3966	1.6%	1.18 [0.54; 2.58]
Potkin 2003	0.2483	0.3637	1.7%	1.28 [0.63; 2.61]
Potkin 2007c	−0.2407	0.4920	1.3%	0.79 [0.30; 2.06]
Protocol 128−301	−0.0779	0.4468	1.4%	0.93 [0.39; 2.22]
Protocol ZIP−NY−97−019	0.4418	0.4990	1.3%	1.56 [0.58; 4.14]
Puech 1998	0.2324	0.3308	1.8%	1.26 [0.66; 2.41]
Rickels 1978	−0.0572	0.4531	1.4%	0.94 [0.39; 2.30]
Riedel 2007	−0.4700	0.5624	1.1%	0.62 [0.21; 1.88]
Sarai 1987	−0.5621	0.5639	1.1%	0.57 [0.19; 1.72]
Selman 1976	0.7295	1.2541	0.3%	2.07 [0.18; 24.23]
Shah 2011	−0.4914	0.7435	0.8%	0.61 [0.14; 2.63]
Simpson 2004	−0.1054	0.2987	1.9%	0.90 [0.50; 1.62]
Study 049	0.1257	0.3621	1.7%	1.13 [0.56; 2.31]
Study 231	0.2972	0.3269	1.8%	1.35 [0.71; 2.55]
Study 233	1.0143	0.3916	1.6%	2.76 [1.28; 5.94]
Tollefson 1997	−0.2260	0.1365	2.5%	0.80 [0.61; 1.04]
Tuason 1984	0.3205	0.8056	0.7%	1.38 [0.28; 6.68]
van der Velde 1975	1.6094	1.5734	0.2%	5.00 [0.23; 109.20]
Weston 1973	−0.3397	0.4553	1.4%	0.71 [0.29; 1.74]
Wetzel 1998	0.3660	0.3503	1.7%	1.44 [0.73; 2.86]
Zhang 2011	−1.8110	0.7802	0.7%	0.16 [0.04; 0.75]
Zhong 2006	−0.3762	0.1847	2.3%	0.69 [0.48; 0.99]
Zimbroff 1997	0.2680	0.3235	1.8%	1.31 [0.69; 2.46]
Zimbroff 2007	−0.8385	0.3297	1.8%	0.43 [0.23; 0.83]
**Total (95% CI)**			**97.5%**	**0.98 [0.84; 1.14]**
Heterogeneity: $Tau^2 = 0.2200$; $Chi^2 = 175.52$, df = 79 ($P < 0.01$); $I^2 = 55\%$				
**blinding = 0**				
Avasthi 2001	−1.2685	0.9280	0.6%	0.28 [0.05; 1.73]
Loza 1999	0.5306	0.6667	0.9%	1.70 [0.46; 6.28]
Taneli 2003	−2.0125	1.5084	0.2%	0.13 [0.01; 2.57]
van Bruggen 2003	1.6094	0.7442	0.8%	5.00 [1.16; 21.50]
**Total (95% CI)**			**2.5%**	**0.99 [0.22; 4.52]**
Heterogeneity: $Tau^2 = 1.5386$; $Chi^2 = 8.42$, df = 3 ($P = 0.04$); $I^2 = 64\%$				
**Total (95% CI)**			**100.0%**	**0.98 [0.84; 1.14]**

Heterogeneity: $Tau^2 = 0.2272$; $Chi^2 = 184.53$, df = 83 ($P < 0.01$); $I^2 = 55\%$
Test for subgroup differences: $Chi^2 = 0.00$, df = 1 ($P = 0.99$)

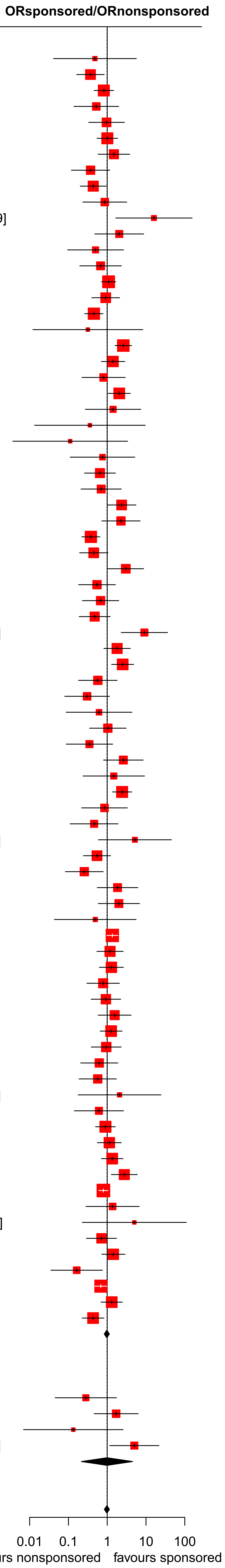

eFigure 10

Weight gain

1. Results of individual comparisons
2. Difference between blinded and open RCTs by recency
3. Difference between blinded and open RCTs by efficacy
4. Differences between blinded and open RCTs sponsored versus non-sponsored drugs

eFigure 10a weight gain individual comparisons

Study or Subgroup	first drug Mean	SD	Total	second drug Mean	SD	Total	Weight	MD [95% CI]
**blinding = 0**								
de Oliviera 2009	−0.40	3.6197	66	0.45	3.6197	33	29.7%	−0.85 [−2.36; 0.66]
**Total (95% CI)**			**66**			**33**	**29.7%**	**−0.85 [−2.36; 0.66]**
Heterogeneity: not applicable								
**blinding = 1**								
Kane 2002	0.65	3.6197	204	0.50	3.6197	104	70.3%	0.15 [−0.70; 1.00]
**Total (95% CI)**			**204**			**104**	**70.3%**	**0.15 [−0.70; 1.00]**
Heterogeneity: not applicable								
**Total (95% CI)**			**270**			**137**	**100.0%**	**−0.15 [−1.04; 0.75]**

Heterogeneity: $Tau^2 = 0.1071$; $Chi^2 = 1.27$, df = 1 (P = 0.26); $I^2 = 21\%$

Test for subgroup differences: $Chi^2 = 1.27$, df = 1 (P = 0.26)

Aripiprazole vs. Haloperidol

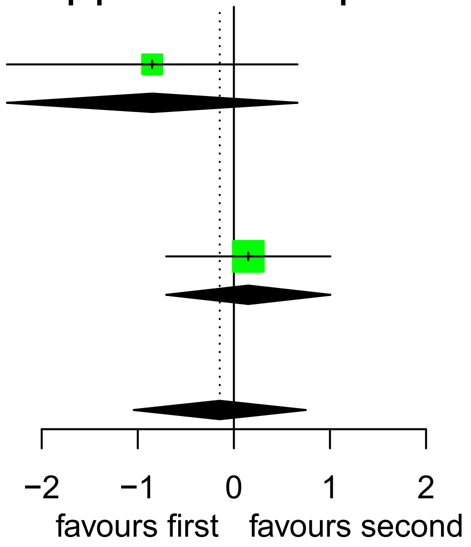

Study or Subgroup	first drug Mean	SD	Total	second drug Mean	SD	Total	Weight	MD [95% CI]
blinding = 0								
Citrome 2015	4.30	4.3000	64	3.80	5.5000	33	22.6%	0.50 [−1.65; 2.65]
Total (95% CI)			64			33	22.6%	0.50 [−1.65; 2.65]
Heterogeneity: not applicable								
blinding = 1								
NCT00905307	1.40	4.3000	90	0.30	2.7000	50	77.4%	1.10 [−0.06; 2.26]
Total (95% CI)			90			50	77.4%	1.10 [−0.06; 2.26]
Heterogeneity: not applicable								
**Total (95% CI)**			**154**			**83**	**100.0%**	**0.96 [−0.06; 1.99]**

Heterogeneity: $Tau^2 = 0$; $Chi^2 = 0.23$, df = 1 (P = 0.63); $I^2 = 0\%$

Test for subgroup differences: $Chi^2 = 0.23$, df = 1 (P = 0.63)

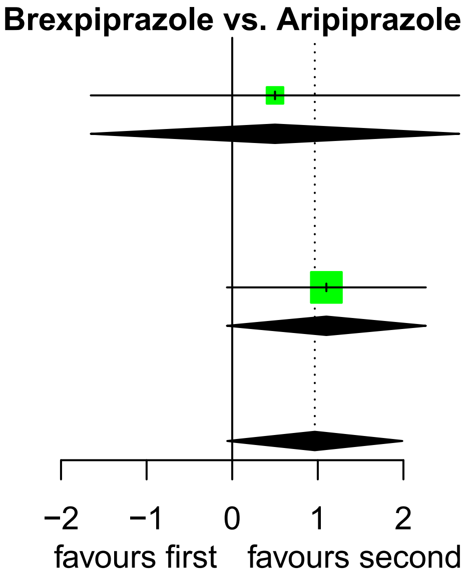

Study or Subgroup	first drug Mean	first drug SD	first drug Total	second drug Mean	second drug SD	second drug Total	Weight	MD [95% CI]
**blinding = 1**								
Beasley 1996b	0.91	3.4800	67	3.55	3.9300	125	10.7%	−2.64 [−3.72; −1.56]
Beasley 1997	−0.37	2.6700	77	2.14	3.2800	167	18.4%	−2.51 [−3.29; −1.73]
Ishigooka 2001	−0.71	3.0000	84	0.96	3.0000	90	14.8%	−1.67 [−2.56; −0.78]
Tollefson 1997	0.02	2.7900	633	1.88	3.5400	1303	55.4%	−1.86 [−2.15; −1.57]
**Total (95% CI)**			**861**			**1685**	**99.3%**	**−2.04 [−2.44; −1.65]**
Heterogeneity: $Tau^2 = 0.0537$; $Chi^2 = 4.31$, df = 3 (P = 0.23); $I^2 = 30\%$								
**blinding = 0**								
Duggan 2005	60.07	10.9000	39	59.88	10.8300	51	0.7%	0.19 [−4.34; 4.72]
**Total (95% CI)**			**39**			**51**	**0.7%**	**0.19 [−4.34; 4.72]**
Heterogeneity: not applicable								
**Total (95% CI)**			**900**			**1736**	**100.0%**	**−2.02 [−2.40; −1.64]**

Heterogeneity: $Tau^2 = 0.0455$; $Chi^2 = 5.17$, df = 4 (P = 0.27); $I^2 = 23\%$

Test for subgroup differences: $Chi^2 = 0.93$, df = 1 (P = 0.34)

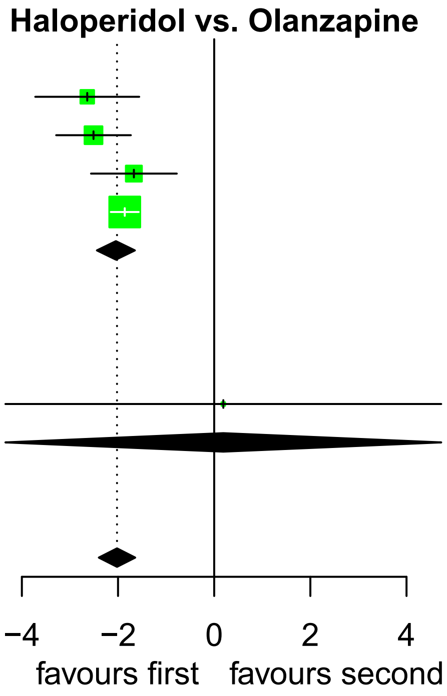

Study or Subgroup	first drug Mean	SD	Total	second drug Mean	SD	Total	Weight	MD [95% CI]
blinding = 1								
Borison 1992	0.80	5.0000	53	1.90	6.0200	53	17.2%	−1.10 [−3.21; 1.01]
Claus 1992	73.90	13.1300	22	73.20	12.2000	22	1.8%	0.70 [−6.79; 8.19]
Min 1993	−0.80	3.6197	19	0.20	3.6197	16	14.1%	−1.00 [−3.41; 1.41]
Peuskens 1995	0.00	3.6197	223	1.05	3.6197	455	52.3%	−1.05 [−1.63; −0.47]
Total (95% CI)			317			546	85.5%	−1.04 [−1.58; −0.50]
Heterogeneity: $Tau^2 = 0$; $Chi^2 = 0.21$, df = 3 (P = 0.98); $I^2 = 0\%$								
blinding = 0								
Nam 2004	2.35	3.2000	20	0.61	4.3300	20	14.5%	1.74 [−0.62; 4.10]
Total (95% CI)			20			20	14.5%	1.74 [−0.62; 4.10]
Heterogeneity: not applicable								
**Total (95% CI)**			**337**			**566**	**100.0%**	**−0.61 [−1.64; 0.41]**

Heterogeneity: $Tau^2 = 0.4367$; $Chi^2 = 5.28$, df = 4 (P = 0.26); $I^2 = 24\%$

Test for subgroup differences: $Chi^2 = 5.07$, df = 1 (P = 0.02)

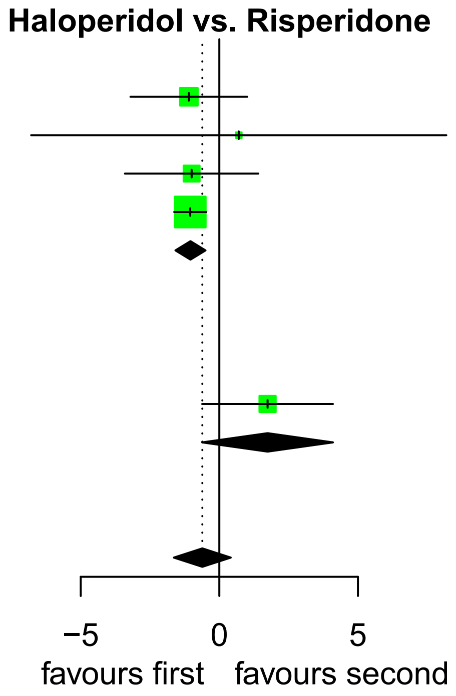

Risperidone vs. Olanzapine

Study or Subgroup	first drug Mean	first drug SD	first drug Total	second drug Mean	second drug SD	second drug Total	Weight	MD [95% CI]
**blinding = 1**								
Conley 2001	1.54	3.5400	155	3.26	5.0800	161	28.6%	−1.72 [−2.68; −0.76]
Hatta 2009	−0.80	2.5000	20	1.10	3.8000	17	6.5%	−1.90 [−4.01; 0.21]
Hatta 2013	0.48	3.5970	16	3.59	4.0890	20	4.7%	−3.11 [−5.63; −0.60]
**Total (95% CI)**			**191**			**198**	**39.8%**	**−1.90 [−2.73; −1.07]**
Heterogeneity: $Tau^2 = 0$; $Chi^2 = 1.03$, df = 2 (P = 0.60); $I^2 = 0\%$								
**blinding = 0**								
Ingole 2009	2.20	1.3500	30	4.67	1.0600	30	60.2%	−2.47 [−3.08; −1.86]
**Total (95% CI)**			**30**			**30**	**60.2%**	**−2.47 [−3.08; −1.86]**
Heterogeneity: not applicable								
**Total (95% CI)**			**221**			**228**	**100.0%**	**−2.25 [−2.80; −1.70]**

Heterogeneity: $Tau^2 = 0.0313$; $Chi^2 = 2.21$, df = 3 (P = 0.53); $I^2 = 0\%$

Test for subgroup differences: $Chi^2 = 1.18$, df = 1 (P = 0.28)

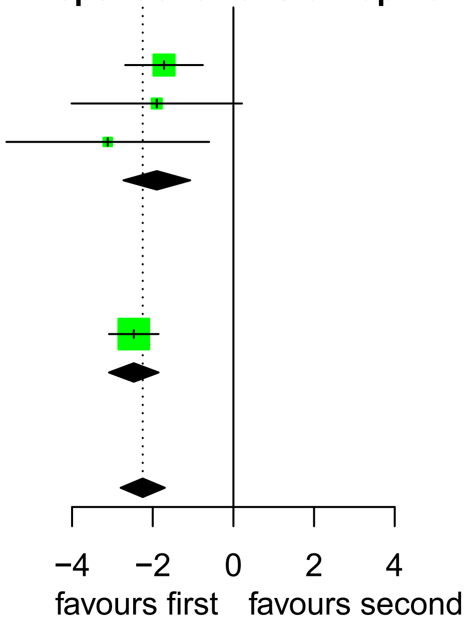

Study or Subgroup	first drug Mean	first drug SD	first drug Total	second drug Mean	second drug SD	second drug Total	Weight	MD [95% CI]
**blinding = 1**								
Grootens 2009	0.10	3.6000	30	6.80	4.1000	31	23.1%	−6.70 [−8.63; −4.77]
Simpson 2004	0.95	5.0100	116	3.54	5.0100	122	27.5%	−2.59 [−3.86; −1.32]
Svestka 2005	−1.00	5.0000	24	2.00	3.0000	24	20.5%	−3.00 [−5.33; −0.67]
**Total (95% CI)**			**170**			**177**	**71.2%**	**−4.07 [−6.64; −1.49]**
Heterogeneity: $Tau^2 = 4.2851$; $Chi^2 = 12.5$, df = 2 ($P < 0.01$); $I^2 = 84\%$								
**blinding = 0**								
Tybura 2014	−0.51	2.3500	59	3.92	3.8100	72	28.8%	−4.43 [−5.49; −3.37]
**Total (95% CI)**			**59**			**72**	**28.8%**	**−4.43 [−5.49; −3.37]**
Heterogeneity: not applicable								
**Total (95% CI)**			**229**			**249**	**100.0%**	**−4.16 [−5.91; −2.40]**

Heterogeneity: $Tau^2 = 2.4749$; $Chi^2 = 13.52$, df = 3 ($P < 0.01$); $I^2 = 78\%$

Test for subgroup differences: $Chi^2 = 0.07$, df = 1 ($P = 0.80$)

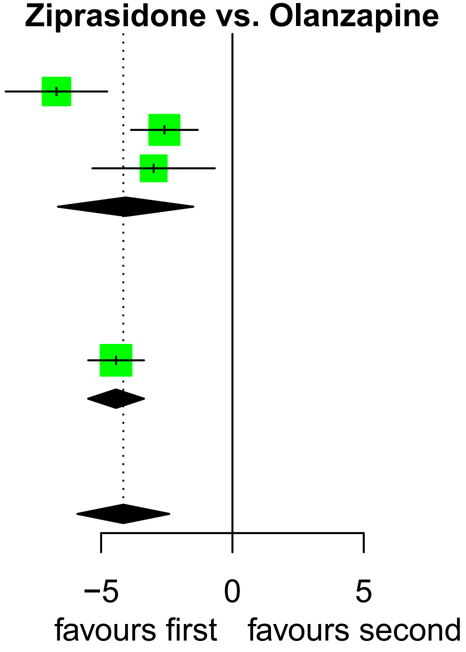

eFigure 10b weight gain difference between blinded and open trials, more recent drug listed first

Study	TE	SE	Weight	MD [95% CI]
Ziprasidone vs. Olanzapine, (nbl=347, nop=131)	0.36	1.4232	12.1%	0.36 [−2.43; 3.15]
Aripiprazole vs. Haloperidol, (nbl=308, nop=99)	1.00	0.8864	21.5%	1.00 [−0.74; 2.74]
Olanzapine vs. Risperidone, (nbl=389, nop=60)	−0.57	0.5256	31.8%	−0.57 [−1.60; 0.46]
Brexpiprazole vs. Aripiprazole, (nbl=140, nop=97)	0.60	1.2477	14.5%	0.60 [−1.85; 3.05]
Risperidone vs. Haloperidol, (nbl=863, nop=40)	2.78	1.2354	14.6%	2.78 [0.36; 5.20]
Olanzapine vs. Haloperidol, (nbl=2546, nop=90)	2.23	2.3210	5.5%	2.23 [−2.31; 6.78]
**Total (95% CI)**			**100.0%**	**0.69 [−0.45; 1.84]**

Heterogeneity: $Tau^2 = 0.7902$; $Chi^2 = 8.16$, df = 5 (P = 0.15); $I^2 = 39\%$

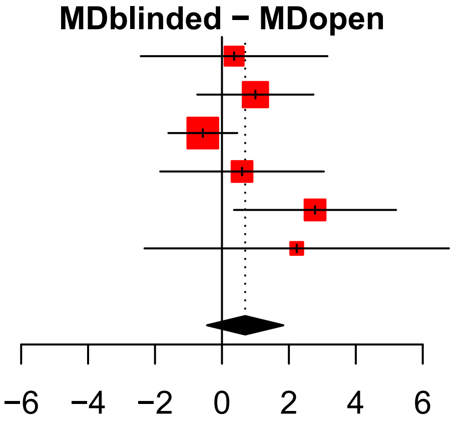

eFigure 10c weight gain difference between blinded and open trials, more weight gain drug according to Huhn et al. 2019 listed first

Study	MD	SE	Weight	MD [95% CI]
Olanzapine vs. Ziprasidone, (nbl=347, nop=131)	−0.3635	1.4232	12.5%	−0.36 [−3.15; 2.43]
Haloperidol vs. Aripiprazole, (nbl=308, nop=99)	−1.0000	0.8864	21.4%	−1.00 [−2.74; 0.74]
Olanzapine vs. Risperidone, (nbl=389, nop=60)	−0.5714	0.5256	30.5%	−0.57 [−1.60; 0.46]
Brexpiprazole vs. Aripiprazole, (nbl=140, nop=97)	0.6000	1.2477	14.8%	0.60 [−1.85; 3.05]
Risperidone vs. Haloperidol, (nbl=863, nop=40)	2.7816	1.2354	15.0%	2.78 [0.36; 5.20]
Olanzapine vs. Haloperidol, (nbl=2546, nop=90)	2.2344	2.3210	5.8%	2.23 [−2.31; 6.78]
**Total (95% CI)**			**100.0%**	**0.20 [−0.99; 1.39]**

Heterogeneity: $Tau^2 = 0.9322$; $Chi^2 = 8.63$, df = 5 (P = 0.12); $I^2 = 42\%$

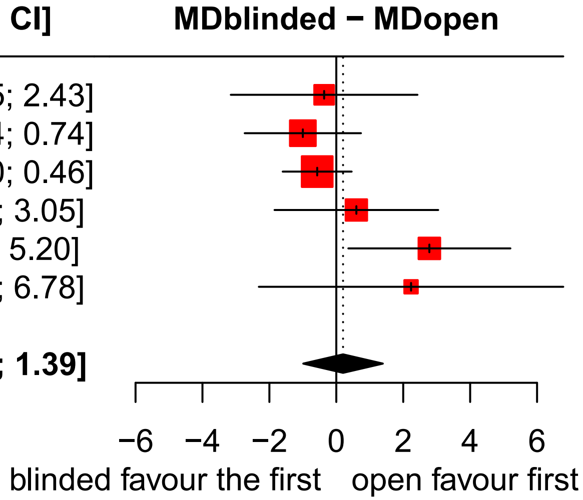

eFigure 10d weight gain sponsored vs non sponsored drugs and blinded versus open trials

Study or Subgroup	TE	SE	Weight	MD [95% CI]
**blinding = 1**				
Addington 2004	1.00	0.5813	1.7%	1.00 [−0.14; 2.14]
Arvanitis 1997	−2.15	0.6138	1.6%	−2.15 [−3.35; −0.95]
Azorin 2006	−0.60	0.5573	1.7%	−0.60 [−1.69; 0.49]
Beasley 1996b	−2.64	0.5516	1.7%	−2.64 [−3.72; −1.56]
Beasley 1997	−2.51	0.3962	1.8%	−2.51 [−3.29; −1.73]
Brook 2005	−0.40	0.4693	1.7%	−0.40 [−1.32; 0.52]
Chan 2007	0.60	0.5316	1.7%	0.60 [−0.44; 1.64]
Clark 1972	4.00	2.1693	0.7%	4.00 [−0.25; 8.25]
Claus 1992	0.70	3.8212	0.3%	0.70 [−6.79; 8.19]
Conley 2001	1.72	0.4911	1.7%	1.72 [0.76; 2.68]
Cooper 2000a	−1.00	0.7032	1.6%	−1.00 [−2.38; 0.38]
Copolov 2000	−1.60	0.4564	1.7%	−1.60 [−2.49; −0.71]
Cutler 2008	−1.68	0.3620	1.8%	−1.68 [−2.39; −0.97]
Davidson 2007	0.70	0.4675	1.7%	0.70 [−0.22; 1.62]
Durgam 2014	0.73	0.3089	1.8%	0.73 [0.13; 1.34]
Freeman 1969	1.77	1.1760	1.2%	1.77 [−0.53; 4.07]
Gattaz 2004	−3.40	0.9527	1.4%	−3.40 [−5.27; −1.53]
Grootens 2009	6.70	0.9870	1.4%	6.70 [4.77; 8.63]
Guirguis 1977	0.40	1.0313	1.3%	0.40 [−1.62; 2.42]
Hale 2000	−1.50	0.3990	1.8%	−1.50 [−2.28; −0.72]
Hwang 2001	−2.25	0.7988	1.5%	−2.25 [−3.82; −0.68]
Hwang 2003	1.50	0.8992	1.4%	1.50 [−0.26; 3.26]
Ishigooka 2001	−1.67	0.4551	1.7%	−1.67 [−2.56; −0.78]
Janssen CR012625	0.96	0.6186	1.6%	0.96 [−0.25; 2.17]
Kane 2002	−0.15	0.4361	1.8%	−0.15 [−1.00; 0.70]
Kane 2007b	0.73	0.2936	1.8%	0.73 [0.15; 1.31]
Kane 2010a	−0.40	0.4175	1.8%	−0.40 [−1.22; 0.42]
Kingstone 1970	1.72	0.7384	1.6%	1.72 [0.28; 3.17]
Kluge 2007	3.00	6.9590	0.1%	3.00 [−10.64; 16.64]
Lin 2003	−0.65	0.9086	1.4%	−0.65 [−2.43; 1.13]
Lundbeck NCT00864045	0.80	0.4677	1.7%	0.80 [−0.12; 1.72]
Marder 2007c	1.20	0.5381	1.7%	1.20 [0.15; 2.25]
Martin 2002	1.80	0.3675	1.8%	1.80 [1.08; 2.52]
McQuade 2004_6weeks	3.18	0.9404	1.4%	3.18 [1.34; 5.02]
Min 1993	−1.00	1.2282	1.2%	−1.00 [−3.41; 1.41]
NCT00350467	0.61	0.4281	1.8%	0.61 [−0.23; 1.45]
NCT01104766	0.00	0.3580	1.8%	0.00 [−0.70; 0.70]
NCT01617187	1.10	0.5747	1.7%	1.10 [−0.03; 2.23]
Petit 1996	−3.13	0.9283	1.4%	−3.13 [−4.95; −1.31]
Peuskens 1995	−1.05	0.2959	1.8%	−1.05 [−1.63; −0.47]
Peuskens 1997	−0.50	0.5106	1.7%	−0.50 [−1.50; 0.50]
Peuskens 1999	1.00	0.4464	1.7%	1.00 [0.13; 1.87]
Potkin 2003	0.50	0.6986	1.6%	0.50 [−0.87; 1.87]
Potkin 2007c	1.13	0.7507	1.5%	1.13 [−0.34; 2.60]
Puech 1998	0.81	0.4372	1.8%	0.81 [−0.05; 1.67]
Riedel 2007	−0.48	1.0389	1.3%	−0.48 [−2.52; 1.56]
Rüther 1988	−0.30	1.3217	1.1%	−0.30 [−2.89; 2.29]
Shah 2011	0.50	0.4950	1.7%	0.50 [−0.47; 1.47]
Simpson 2004	2.59	0.6497	1.6%	2.59 [1.32; 3.86]
Study 049	0.10	0.5013	1.7%	0.10 [−0.88; 1.08]
Study 231	3.10	0.4236	1.8%	3.10 [2.27; 3.93]
Study 233	1.50	0.3658	1.8%	1.50 [0.78; 2.22]
Study 3000	−2.10	0.5657	1.7%	−2.10 [−3.21; −0.99]
Study 3004	0.00	0.5823	1.7%	0.00 [−1.14; 1.14]
Study 3005	−1.00	0.3220	1.8%	−1.00 [−1.63; −0.37]
Tollefson 1997	−1.86	0.1480	1.9%	−1.86 [−2.15; −1.57]
Wetzel 1998	1.20	0.6441	1.6%	1.20 [−0.06; 2.46]
Zborowski 1995	−2.99	0.4753	1.7%	−2.99 [−3.92; −2.06]
Zhong 2006	0.48	0.5566	1.7%	0.48 [−0.61; 1.57]
Zimbroff 2007	0.00	0.4552	1.7%	0.00 [−0.89; 0.89]
**Total (95% CI)**			**94.4%**	**0.07 [−0.37; 0.51]**
Heterogeneity: $Tau^2 = 2.5130$; $Chi^2 = 628.32$, df = 59 ($P < 0.01$); $I^2 = 91\%$				
**blinding = 0**				
de Oliviera 2009	0.85	0.7717	1.5%	0.85 [−0.66; 2.36]
Dossenbach 2007	−0.60	0.5595	1.7%	−0.60 [−1.70; 0.50]
Duggan 2005	0.19	2.3122	0.6%	0.19 [−4.34; 4.72]
Tybura 2014	3.30	0.4355	1.8%	3.30 [2.44; 4.15]
**Total (95% CI)**			**5.6%**	**1.10 [−0.87; 3.06]**
Heterogeneity: $Tau^2 = 3.0763$; $Chi^2 = 31.97$, df = 3 ($P < 0.01$); $I^2 = 91\%$				
**Total (95% CI)**			**100.0%**	**0.13 [−0.31; 0.56]**

Heterogeneity: $Tau^2 = 2.5696$; $Chi^2 = 698.17$, df = 63 ($P < 0.01$); $I^2 = 91\%$

Test for subgroup differences: $Chi^2 = 1.00$, df = 1 ($P = 0.32$)

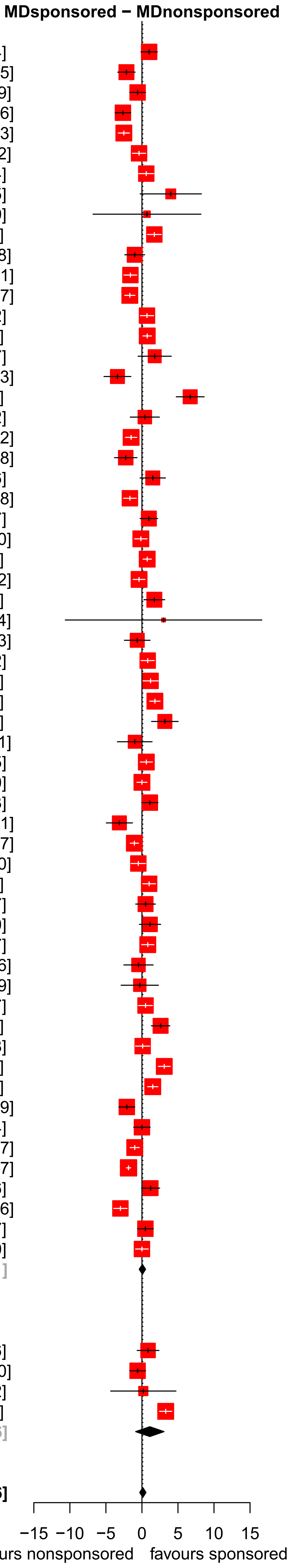

eFigure 11

Prolactin

1. Results of individual comparisons
2. Difference between blinded and open RCTs by recency
3. Difference between blinded and open RCTs by efficacy
4. Differences between blinded and open RCTs sponsored versus non-sponsored drugs

eFigure 11a prolactin individual comparisons

Study or Subgroup	first drug Mean	first drug SD	first drug Total	second drug Mean	second drug SD	second drug Total	Weight	MD [95% CI]
blinding = 0								
Knegtering 2004	13.80	17.9000	25	57.90	39.7000	26	11.2%	−44.10 [−60.90; −27.30]
blinding = 1								
Zhong 2006	−11.50	33.2500	209	35.50	30.4000	231	88.8%	−47.00 [−52.97; −41.03]
**Total (95% CI)**			**234**			**257**	**100.0%**	**−46.67 [−52.30; −41.05]**

Heterogeneity: $Tau^2 = 0$; $Chi^2 = 0.10$, df = 1 (P = 0.75); $I^2 = 0\%$
Test for subgroup differences: $Chi^2 = 0.10$, df = 1 (P = 0.75)

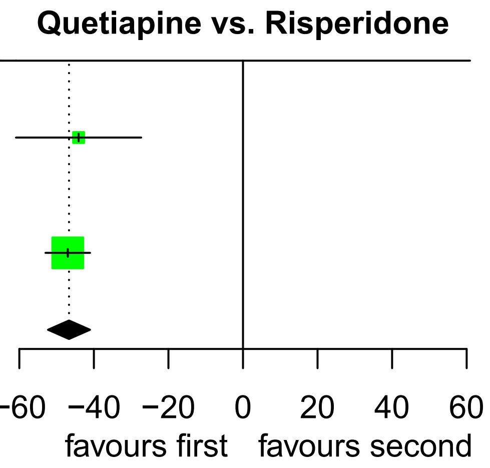

Study or Subgroup	first drug Mean	first drug SD	first drug Total	second drug Mean	second drug SD	second drug Total	Weight	MD [95% CI]
**blinding = 1**								
Addington 2004	−9.00	36.7063	110	18.00	36.7063	130	39.8%	−27.00 [−36.32; −17.68]
Zhang 2011	−3.50	47.4137	118	61.10	47.2366	121	38.5%	−64.60 [−76.60; −52.60]
**Total (95% CI)**			**228**			**251**	**78.3%**	**−45.60 [−82.45; −8.76]**
Heterogeneity: $Tau^2 = 676.8276$; $Chi^2 = 23.52$, df = 1 ($P < 0.01$); $I^2 = 96\%$								
**blinding = 0**								
Sonmez 2009	23.25	43.7222	10	76.65	43.7222	10	21.7%	−53.40 [−91.72; −15.08]
**Total (95% CI)**			**238**			**261**	**100.0%**	**−47.20 [−72.92; −21.49]**

Heterogeneity: $Tau^2 = 409.9020$; $Chi^2 = 23.90$, df = 2 ($P < 0.01$); $I^2 = 92\%$

Test for subgroup differences: $Chi^2 = 0.08$, df = 1 ($P = 0.77$)

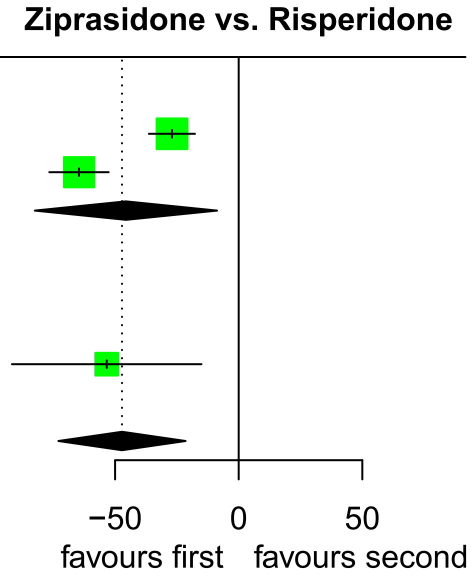

Study or Subgroup	first drug Mean	first drug SD	first drug Total	second drug Mean	second drug SD	second drug Total	Weight	MD [95% CI]
**blinding = 1**								
Chan 2007	−9.00	56.4000	38	55.40	42.3000	27	4.8%	−64.40 [−88.40; −40.40]
Hwang 2012	−9.00	36.7063	49	55.40	36.7063	34	10.7%	−64.40 [−80.46; −48.34]
Potkin 2003	−6.50	24.3000	181	47.90	24.6000	91	72.3%	−54.40 [−60.57; −48.23]
**Total (95% CI)**			**268**			**152**	**87.8%**	**−57.17 [−64.61; −49.73]**
Heterogeneity: $Tau^2 = 10.0300$; $Chi^2 = 1.78$, df = 2 (P = 0.41); $I^2 = 0\%$								
**blinding = 0**								
de Boer 2011	10.09	6.9800	18	55.71	31.7500	18	12.2%	−45.62 [−60.64; −30.60]
**Total (95% CI)**			**286**			**170**	**100.0%**	**−54.87 [−60.12; −49.63]**

Heterogeneity: $Tau^2 < 0.0001$; $Chi^2 = 3.44$, df = 3 (P = 0.33); $I^2 = 13\%$

Test for subgroup differences: $Chi^2 = 1.82$, df = 1 (P = 0.18)

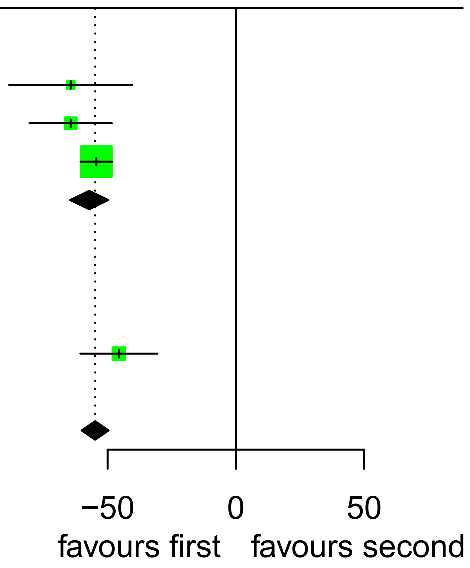

Study or Subgroup	first drug Mean	first drug SD	first drug Total	second drug Mean	second drug SD	second drug Total	Weight	MD [95% CI]
**blinding = 0**								
Citrome 2015	0.12	10.2327	64	−2.75	4.1417	33	78.8%	2.86 [−0.01; 5.74]
**blinding = 1**								
NCT00905307	−2.70	18.9900	90	−9.68	19.9000	50	21.2%	6.98 [0.21; 13.75]
**Total (95% CI)**			**154**			**83**	**100.0%**	**3.74 [0.44; 7.03]**

Heterogeneity: $Tau^2 = 1.4284$; $Chi^2 = 1.20$, df = 1 (P = 0.27); $I^2 = 17\%$

Test for subgroup differences: $Chi^2 = 1.20$, df = 1 (P = 0.27)

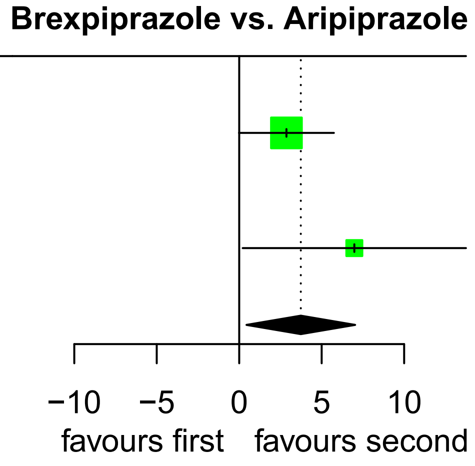

Study or Subgroup	first drug Mean	SD	Total	second drug Mean	SD	Total	Weight	MD [95% CI]
**blinding = 0**								
Chung 2000	96.11	25.5400	15	167.36	83.6300	18	7.6%	−71.25 [−111.99; −30.51]
**blinding = 1**								
Claus 1992	13.20	14.5700	16	26.37	27.8400	18	21.1%	−13.17 [−27.88; 1.54]
Marder 1994	19.00	22.0000	55	19.00	11.0000	45	26.7%	0.00 [−6.64; 6.64]
Peuskens 1995	17.00	19.0000	88	31.00	28.0000	190	27.2%	−14.00 [−19.62; −8.38]
Yen 2004	40.90	26.3000	20	42.40	37.2000	21	17.4%	−1.50 [−21.15; 18.15]
**Total (95% CI)**			**179**			**274**	**92.4%**	**−7.58 [−16.02; 0.86]**
Heterogeneity: $Tau^2 = 44.1318$; $Chi^2 = 10.83$, df = 3 (P = 0.01); $I^2 = 72\%$								
**Total (95% CI)**			**194**			**292**	**100.0%**	**−12.26 [−25.36; 0.84]**

Heterogeneity: $Tau^2 = 155.8005$; $Chi^2 = 19.92$, df = 4 (P < 0.01); $I^2 = 80\%$

Test for subgroup differences: $Chi^2 = 9.00$, df = 1 (P < 0.01)

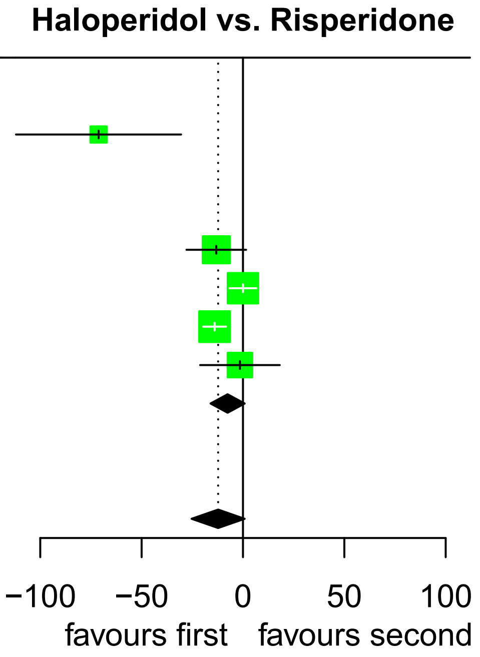

Study or Subgroup	first drug Mean	first drug SD	first drug Total	second drug Mean	second drug SD	second drug Total	Weight	MD [95% CI]
**blinding = 1**								
Arvanitis 1997	−1.34	18.8800	56	18.30	18.8800	24	19.9%	−19.64 [−28.67; −10.62]
Copolov 2000	−16.53	51.5000	179	5.90	51.4900	177	16.3%	−22.43 [−33.13; −11.73]
Murasaki 2001	8.10	8.5000	85	17.80	18.8000	82	33.4%	−9.70 [−14.15; −5.25]
**Total (95% CI)**			**320**			**283**	**69.5%**	**−16.15 [−24.38; −7.92]**
Heterogeneity: Tau^2 = 36.2355; Chi^2 = 7.18, df = 2 (P = 0.03); I^2 = 72%								
**blinding = 0**								
Atmaca 2002	15.74	4.8300	18	31.41	10.1900	17	30.5%	−15.67 [−21.00; −10.34]
**Total (95% CI)**			**338**			**300**	**100.0%**	**−15.57 [−21.07; −10.07]**

Heterogeneity: Tau^2 = 18.4316; Chi^2 = 7.84, df = 3 (P = 0.05); I^2 = 62%

Test for subgroup differences: Chi^2 = 0.01, df = 1 (P = 0.92)

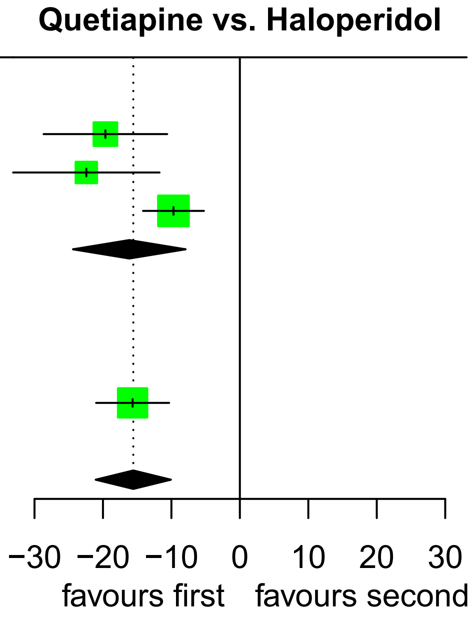

eFigure 11b prolactin incrase difference between blinded and open trials, more recent drug listed first

Study	MD	SE	Weight	MD [95% CI]
Quetiapine vs. Risperidone, (nbl=440, nop=51)	−2.9000	9.0954	15.2%	−2.90 [−20.73; 14.93]
Brexpiprazole vs. Aripiprazole, (nbl=140, nop=97)	4.1158	3.7527	33.7%	4.12 [−3.24; 11.47]
Risperidone vs. Haloperidol, (nbl=453, nop=33)	−63.6712	21.2268	3.9%	−63.67 [−105.28; −22.07]
Aripiprazole vs. Risperidone, (nbl=420, nop=36)	−11.5499	8.5515	16.5%	−11.55 [−28.31; 5.21]
Quetiapine vs. Haloperidol, (nbl=603, nop=35)	−0.4822	5.0039	28.3%	−0.48 [−10.29; 9.33]
Ziprasidone vs. Risperidone, (nbl=479, nop=20)	7.7979	27.1243	2.4%	7.80 [−45.36; 60.96]
**Total (95% CI)**			**100.0%**	**−3.36 [−11.91; 5.19]**

Heterogeneity: Tau^2 = 42.3200; Chi^2 = 12.20, df = 5 (P = 0.03); I^2 = 59%

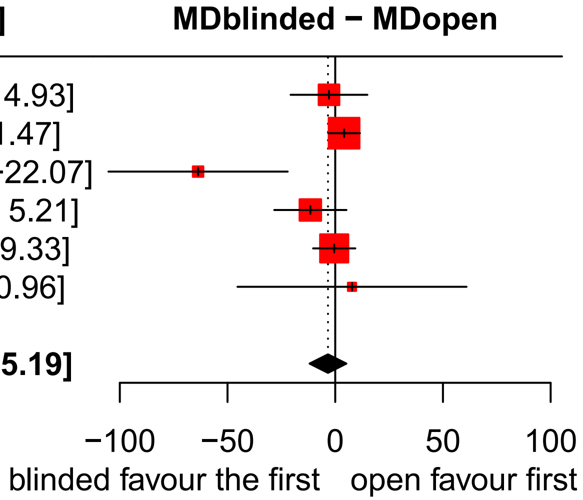

eFigure 11c positive symptoms difference between blinded and open trials, drug with more prolactin increase according to Huhn et al. 2019 listed first

Study	MD	SE	Weight	MD [95% CI]
Risperidone vs. Quetiapine, (nbl=440, nop=51)	2.9000	9.0954	8.6%	2.90 [−14.93; 20.73]
Brexpiprazole vs. Aripiprazole, (nbl=140, nop=97)	4.1158	3.7527	50.6%	4.12 [−3.24; 11.47]
Risperidone vs. Haloperidol, (nbl=453, nop=33)	−63.6712	21.2268	1.6%	−63.67 [−105.28; −22.07]
Risperidone vs. Aripiprazole, (nbl=420, nop=36)	11.5499	8.5515	9.7%	11.55 [−5.21; 28.31]
Haloperidol vs. Quetiapine, (nbl=603, nop=35)	0.4822	5.0039	28.5%	0.48 [−9.33; 10.29]
Risperidone vs. Ziprasidone, (nbl=479, nop=20)	−7.7979	27.1243	1.0%	−7.80 [−60.96; 45.36]
**Total (95% CI)**			**100.0%**	**2.51 [−2.72; 7.75]**

Heterogeneity: $Tau^2 < 0.0001$; $Chi^2 = 11.33$, df = 5 (P = 0.05); $I^2 = 56\%$

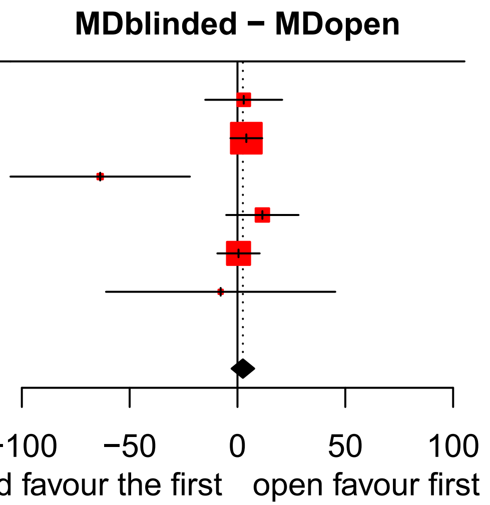

eFigure 11d prolactin increase sponsored vs non sponsored drugs and blinded versus open trials

Study or Subgroup	MD	SE	Weight	MD [95% CI]
**blinding = 1**				
A1281050	18.6000	5.9227	2.0%	18.60 [6.99; 30.21]
Addington 2004	27.0000	4.7553	2.0%	27.00 [17.68; 36.32]
Arvanitis 1997	19.6450	4.6062	2.0%	19.64 [10.62; 28.67]
Beasley 1996b	8.7600	2.5035	2.0%	8.76 [3.85; 13.67]
Beasley 1997	17.1000	3.4835	2.0%	17.10 [10.27; 23.93]
Brook 2005	12.0000	4.1298	2.0%	12.00 [3.91; 20.09]
Chan 2007	64.4000	12.2466	1.8%	64.40 [40.40; 88.40]
Claus 1992	−13.1700	7.5051	1.9%	−13.17 [−27.88; 1.54]
Copolov 2000	22.4300	5.4585	2.0%	22.43 [11.73; 33.13]
Cutler 2008	−0.7000	2.7794	2.0%	−0.70 [−6.15; 4.75]
Davidson 2007	−57.4400	5.4768	2.0%	−57.44 [−68.17; −46.71]
Durgam 2014	39.4629	3.5637	2.0%	39.46 [32.48; 46.45]
Gattaz 2004	23.2000	27.6221	1.1%	23.20 [−30.94; 77.34]
Goff 1998	18.2200	4.7380	2.0%	18.22 [8.93; 27.51]
Grootens 2009	−26.8000	19.7390	1.4%	−26.80 [−65.49; 11.89]
Hale 2000	13.4800	5.9531	2.0%	13.48 [1.81; 25.15]
Hera 041−021	2.0000	4.0170	2.0%	2.00 [−5.87; 9.87]
Hera 041−022	−5.4700	3.1293	2.0%	−5.47 [−11.60; 0.66]
Hwang 2012	64.4000	8.1930	1.9%	64.40 [48.34; 80.46]
Ishigooka 2001	43.2000	30.4465	1.0%	43.20 [−16.47; 102.87]
Janssen CR012625	−42.4900	7.4715	1.9%	−42.49 [−57.13; −27.85]
Kane 2002	29.5500	5.7967	2.0%	29.55 [18.19; 40.91]
Kane 2007b	−57.0200	5.9968	2.0%	−57.02 [−68.77; −45.27]
Kane 2010a	16.0000	6.3823	2.0%	16.00 [3.49; 28.51]
Lin 2003	−35.4400	7.5548	1.9%	−35.44 [−50.25; −20.63]
Lundbeck NCT00864045	−8.1600	3.2952	2.0%	−8.16 [−14.62; −1.70]
Marder 1994	0.0000	3.3895	2.0%	0.00 [−6.64; 6.64]
Marder 2007c	−40.8700	4.2912	2.0%	−40.87 [−49.28; −32.46]
Murasaki 2001	9.7000	2.2716	2.0%	9.70 [5.25; 14.15]
NCT01104766	−2.3349	3.7512	2.0%	−2.33 [−9.69; 5.02]
Peuskens 1995	−14.0000	2.8686	2.0%	−14.00 [−19.62; −8.38]
Peuskens 1997	10.0000	7.0687	1.9%	10.00 [−3.85; 23.85]
Potkin 2003	54.4000	3.1484	2.0%	54.40 [48.23; 60.57]
Potkin 2007c	60.6500	8.6422	1.9%	60.65 [43.71; 77.59]
Protocol 128−301	7.8100	4.1233	2.0%	7.81 [−0.27; 15.89]
Rüther 1988	−48.0000	18.9592	1.5%	−48.00 [−85.16; −10.84]
Study 049	14.0000	4.7899	2.0%	14.00 [4.61; 23.39]
Study 231	−1.4100	1.8400	2.0%	−1.41 [−5.02; 2.20]
Study 233	−7.9800	2.3888	2.0%	−7.98 [−12.66; −3.30]
Study 3000	21.3000	4.2339	2.0%	21.30 [13.00; 29.60]
Study 3004	38.8000	5.4535	2.0%	38.80 [28.11; 49.49]
Study 94202 2002	19.6000	6.5935	2.0%	19.60 [6.68; 32.52]
Tollefson 1997	15.4300	1.6928	2.1%	15.43 [12.11; 18.75]
Wetzel 1991	30.0000	7.5827	1.9%	30.00 [15.14; 44.86]
Wetzel 1998	−52.9000	15.9432	1.6%	−52.90 [−84.15; −21.65]
Zborowski 1995	5.8400	7.0397	1.9%	5.84 [−7.96; 19.64]
Zhang 2011	64.6000	6.1231	2.0%	64.60 [52.60; 76.60]
Zhong 2006	47.0000	3.0480	2.0%	47.00 [41.03; 52.97]
Zimbroff 1997	3.4100	3.4768	2.0%	3.41 [−3.40; 10.22]
Zimbroff 2007	−7.2000	5.0111	2.0%	−7.20 [−17.02; 2.62]
**Total (95% CI)**			**96.2%**	**8.51 [0.14; 16.87]**
Heterogeneity: Tau^2 = 850.6326; Chi^2 = 1499.08, df = 49 (P < 0.01); I^2 = 97%				
**blinding = 0**				
de Boer 2011	45.6200	7.6623	1.9%	45.62 [30.60; 60.64]
Knegtering 2004	44.1000	8.5694	1.9%	44.10 [27.30; 60.90]
**Total (95% CI)**			**3.8%**	**44.94 [33.75; 56.14]**
Heterogeneity: Tau^2 = 0; Chi^2 = 0.02, df = 1 (P = 0.89); I^2 = 0%				
**Total (95% CI)**			**100.0%**	**9.90 [1.63; 18.16]**
Heterogeneity: Tau^2 = 864.4804; Chi^2 = 1539.85, df = 51 (P < 0.01); I^2 = 97%				
Test for subgroup differences: Chi^2 = 26.12, df = 1 (P < 0.01)				

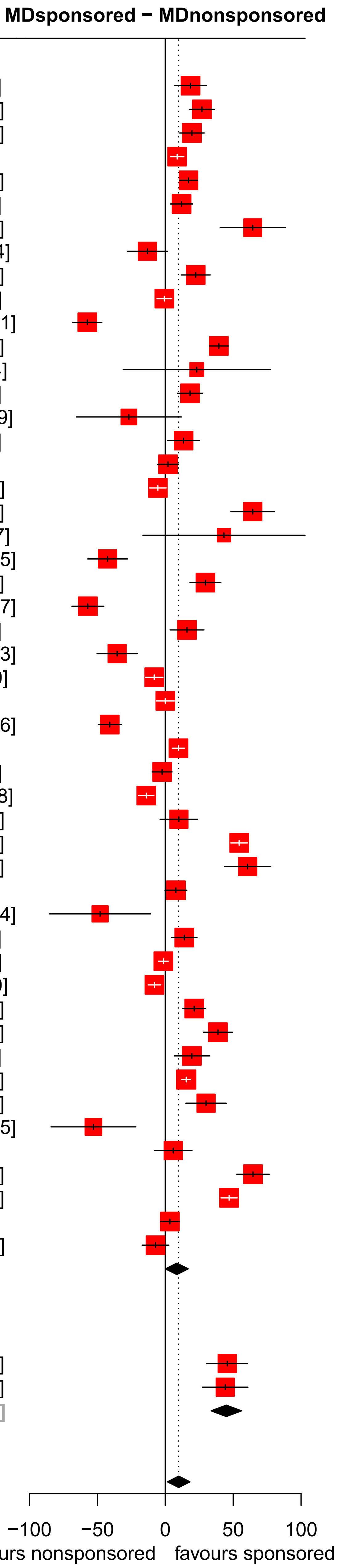

Supplement: Supplementary file 1 — eAppendix [file 41537_2024_442_MOESM1_ESM.pdf]
